# Supplementary material for: Terminal deoxynucleotidyl transferase and CD84 identify human multi-potent lymphoid progenitors
Source: Nat Commun. 2024 Jul 13;15:5910. doi: 10.1038/s41467-024-49883-w (PMC11246490; doi:10.1038/s41467-024-49883-w)
Supplement: Supplementary file 1 — Supplementary Information [file 41467_2024_49883_MOESM1_ESM.pdf]

Donor #	Experiments	Vendor	Sex	Age
1	Screen	AllCells	F	40s
2	Screen	AllCells	F	30s
3	Screen	AllCells	M	50s
4	Validation	Stemexpress	F	40s
5	inTAC	AllCells	F	20s
6	inTAC	AllCells	M	50s
7	differentiation	AllCells	F	20s
8	differentiation	AllCells	M	50s
9	cloning	AllCells	F	20s

Supplementary Table 1. Bone Marrow Donor Deidentified Information

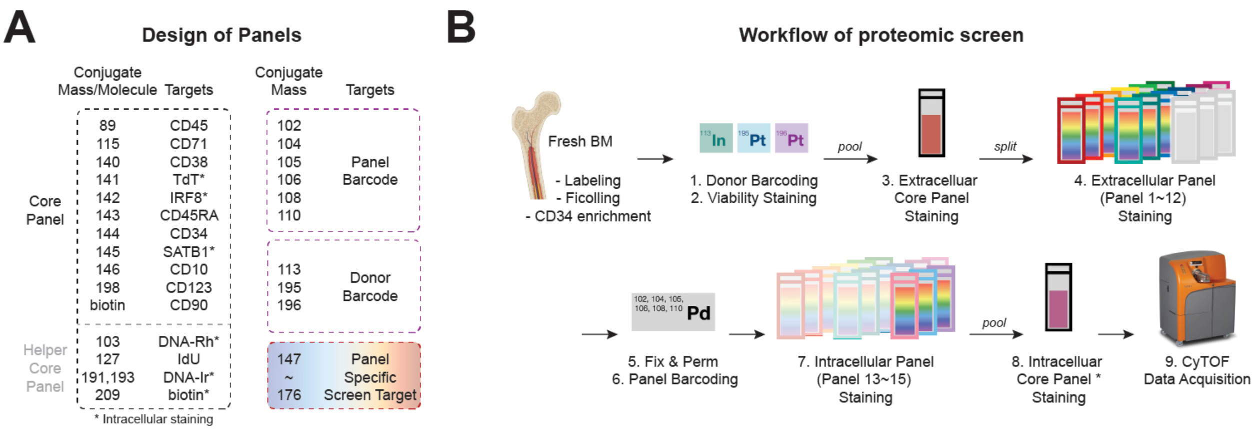

Supplementary Figure 1. Design of the CyTOF proteomics screen

(A) Design of CyTOF screen panels.

(B) Workflow of CyTOF screen. Created with BioRender.com, released under a Creative Commons Attribution-NonCommercial-NoDerivs 4.0 International license.

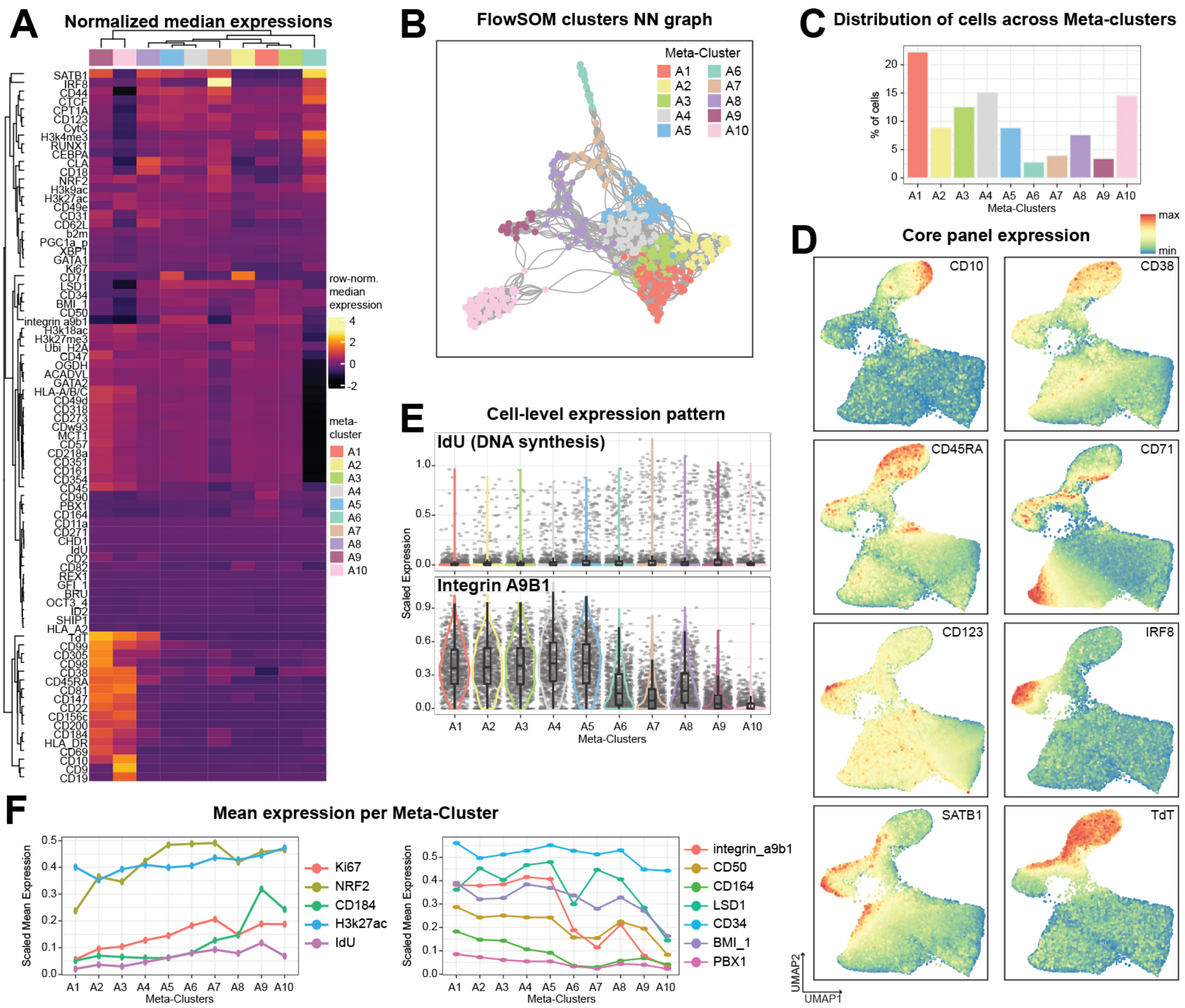

Supplementary Figure 2. Meta-Cluster level analysis of the proteomic screen

(A) Row-normalized heatmap of scaled median expression of molecules by meta-clusters.

(B) Nearest-Neighbor graph of FlowSOM Clusters. 5 nearest neighbors were calculated from Euclidean distances among FlowSOM clusters and the layout was calculated using the force-directed layout by Fruchterman-Reingold layout algorithm.

(C) Distribution of number of cells in each meta-cluster.

(D) UMAP of all cells in CyTOF screen colored by core panel targets expression level. Additional UMAPs colored by all other protein markers are provided in Supplementary Data 4.

(E) Violin plots of IdU (top) and Integrin A9B1 (bottom).

(F) Line graph of mean expression per meta-cluster of protein molecules that are increasing (left) or decreasing (right) along the hematopoietic differentiation.

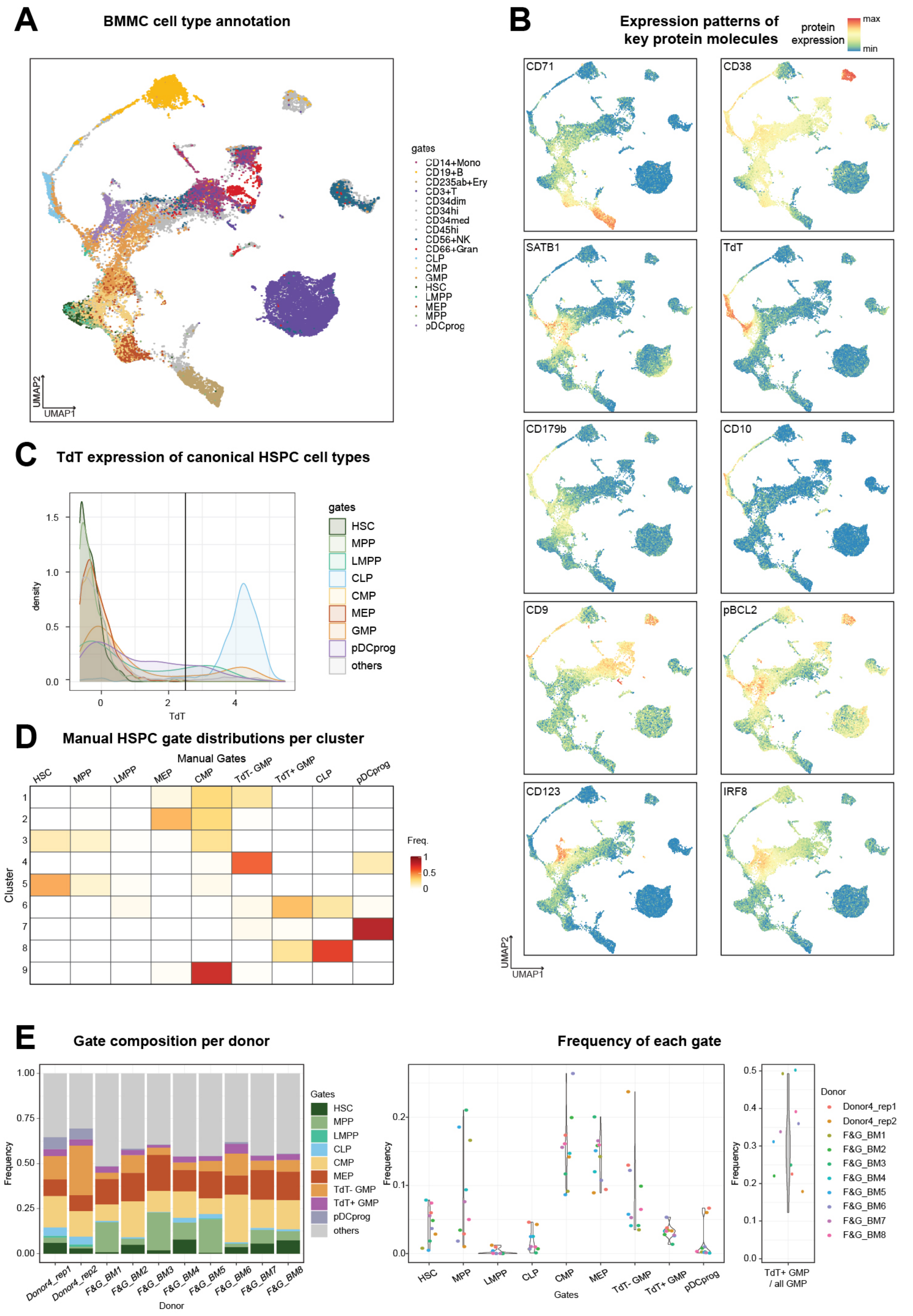

A
BMMC cell type annotation
gates
CD14+Mono
CD19+B
CD235ab+Ery
CD3+T
CD34dim
CD34hi
CD34med
CD45hi
CD56+NK
CD66+Gran
CLP
CMP
GMP
HSC
LMPP
MEP
MPP
pDCprog
UMAP2
UMAP1
B
Expression patterns of key protein molecules
protein expression
max
min
CD71
CD38
SATB1
TdT
CD179b
CD10
CD9
pBCL2
CD123
IRF8
UMAP2
UMAP1
C
TdT expression of canonical HSPC cell types
density
TdT
gates
HSC
MPP
LMPP
CLP
CMP
MEP
GMP
pDCprog
others
D
Manual HSPC gate distributions per cluster
Manual Gates
HSC
MPP
LMPP
MEP
CMP
TdT- GMP
TdT+ GMP
CLP
pDCprog
Cluster
Freq.
E
Gate composition per donor
Frequency
Donor
Donor4_rep1
Donor4_rep2
F&G_BM1
F&G_BM2
F&G_BM3
F&G_BM4
F&G_BM5
F&G_BM6
F&G_BM7
F&G_BM8
Gates
HSC
MPP
LMPP
CLP
CMP
MEP
TdT- GMP
TdT+ GMP
pDCprog
others
Frequency of each gate
Frequency
Gates
TdT+ GMP / all GMP
Donor

Supplementary Figure 3. Analysis of BMMC proteome and manual HSPC gates
(A) UMAP of finalized CyTOF panel colored by manual gates.
(B) UMAP of finalized CyTOF panel colored by protein molecule expression levels. Additional UMAPs colored by all other protein markers are provided in Supplementary Data 5.
(C) Histogram of TdT protein expression per HSPC cell type by manual gates.
(D) Confusion matrix of Leiden Clusters representing the frequency of manually gated cell types per cluster.
(E) Frequency of each HSPC cell type in samples as a stacked bar plot per donor (left) or violin plot per each cell type (right).

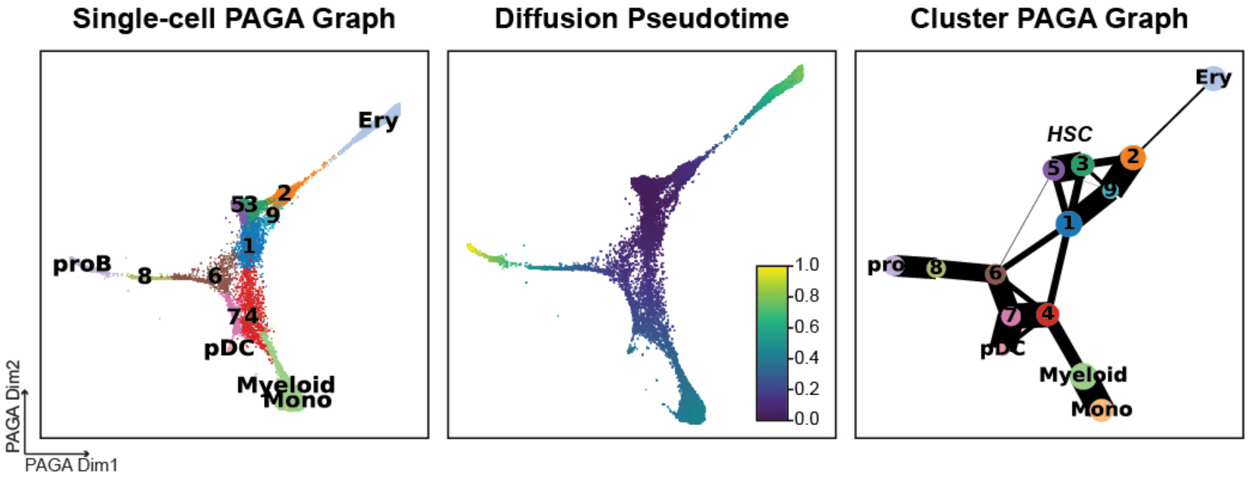

Supplementary Figure 4. Inference of the developmental trajectory among the HSPC clusters

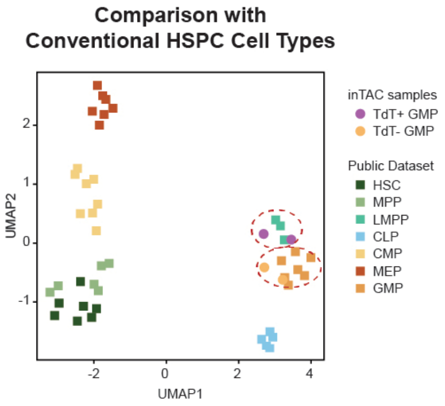

Supplementary Figure 5. UMAP of chromatin accessibility from inTAC-seq data and reference HSPC ATAC-seq data

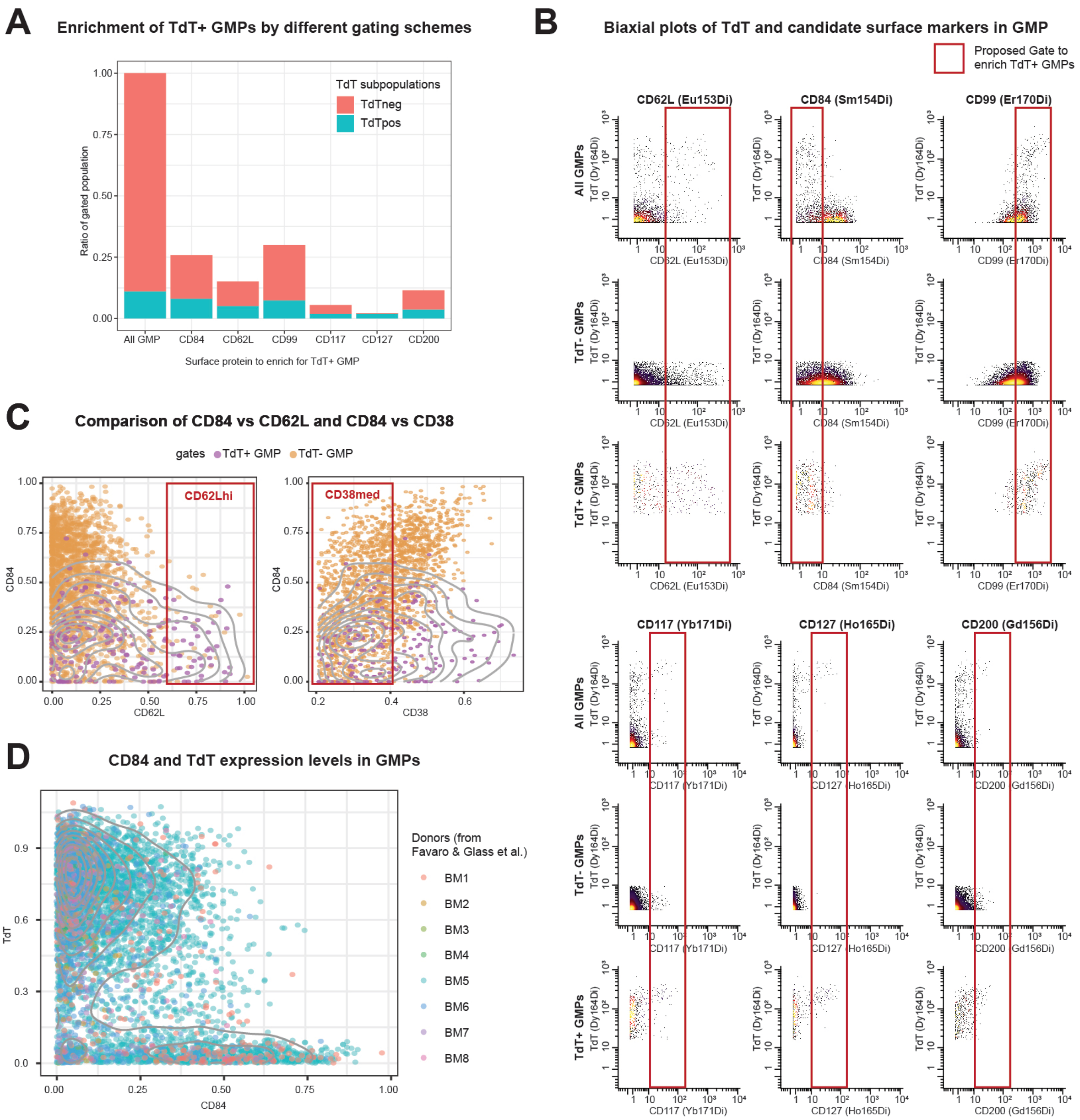

Supplementary Figure 6. Surface markers to enrich TdT+ GMP

(A) Composition of TdT+ or TdT- GMPs from gating schemes with candidate surface proteins to enrich for TdT+ GMPs.

(B) Biaxial plots of GMPs by TdT and candidate surface proteins.

(C) Biaxial plots of GMPs by CD84 and previously suggested surface proteins, CD62L (left) and CD38 (right), to distinguish lymphoid progenitors. Grey lines represent 2D density of TdT+ GMPs in each plot.

(D) Biaxial plots of GMPs by CD84 and TdT of eight different bone marrows from Favaro and Glass et al., in preparation.

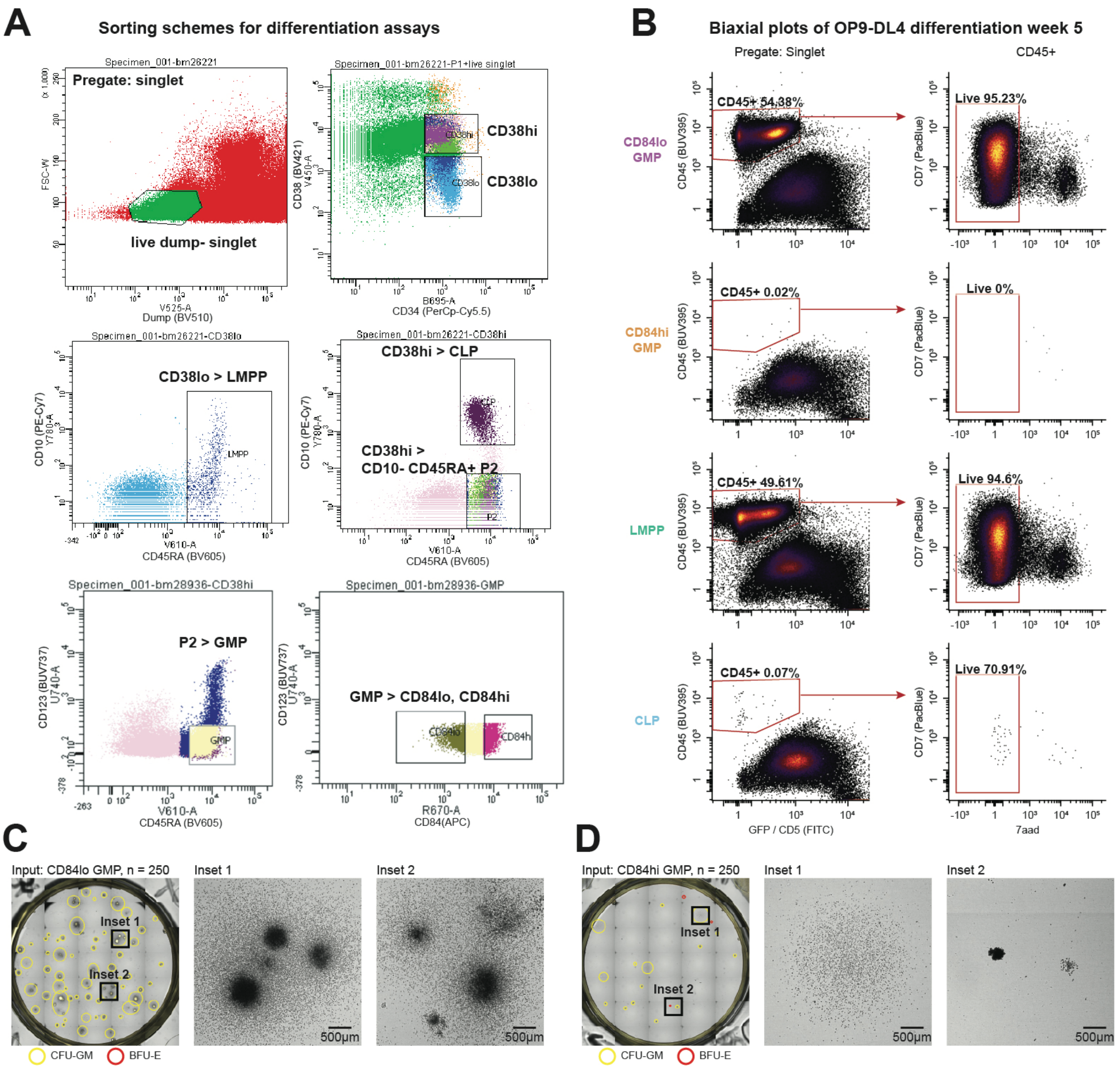

Supplementary Figure 7. Functional Differentiation Assay Results

(A) Sorting schemes for functional differentiation assays. Representative of the same sorting scheme for all functional differentiation assays.

(B) OP9-DL4 bulk co-culture differentiation results after 5 weeks as biaxial plots.

(C) Colony forming assay with CD84lo GMPs (n=250) after 2 weeks.

(D) Colony forming assay with CD84hi GMPs (n=250) after 2 weeks.
