# Supplementary material for: Terminal deoxynucleotidyl transferase and CD84 identify human multi-potent lymphoid progenitors
Source: Nat Commun. 2024 Jul 13;15:5910. doi: 10.1038/s41467-024-49883-w (PMC11246490; doi:10.1038/s41467-024-49883-w)
Supplement: Supplementary file 3 — Description of Additional Supplementary Files [file 41467_2024_49883_MOESM3_ESM.pdf]

Description of Additional Supplementary Files

Supplementary Data 1. Mass Cytometry Screen Panels

Supplementary Data 2. MetaCluster Differential Analyses Results

Supplementary Data 3. Flow Cytometry Panels

Supplementary Data 4. UMAP of all positive hits from the mass cytometry screen

Supplementary Data 5. UMAP of all markers in the finalized human bone marrow mass cytometry panel
