# Supplementary figures and images for: Terminal deoxynucleotidyl transferase and CD84 identify human multi-potent lymphoid progenitors (part 1 of 2)
Source: Nat Commun. 2024 Jul 13;15:5910. doi: 10.1038/s41467-024-49883-w (PMC11246490; doi:10.1038/s41467-024-49883-w)

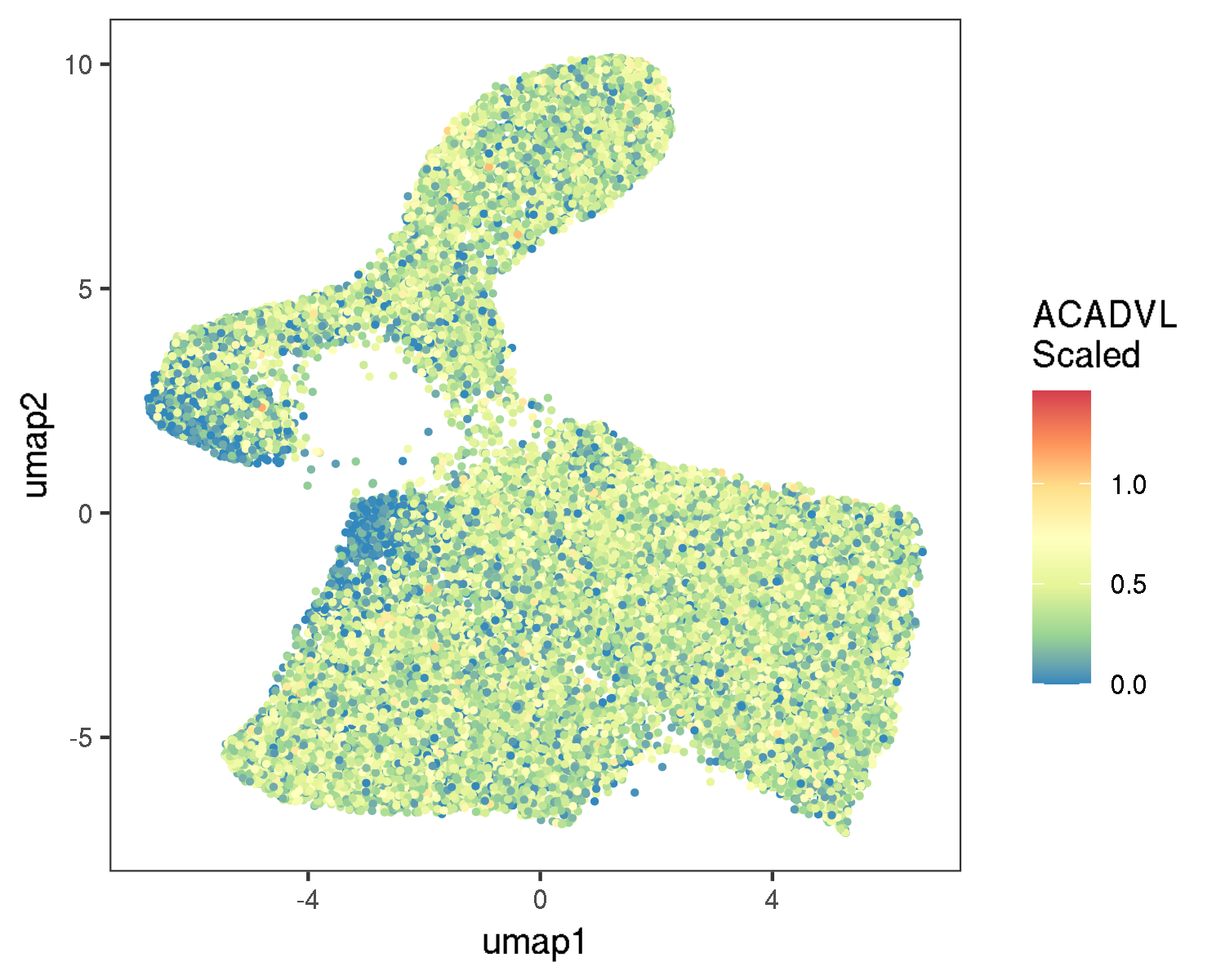

Supplement: Supplementary file 7 — Supplementary Data 4 [file 41467_2024_49883_MOESM7_ESM.zip › png/ACADVL.png]

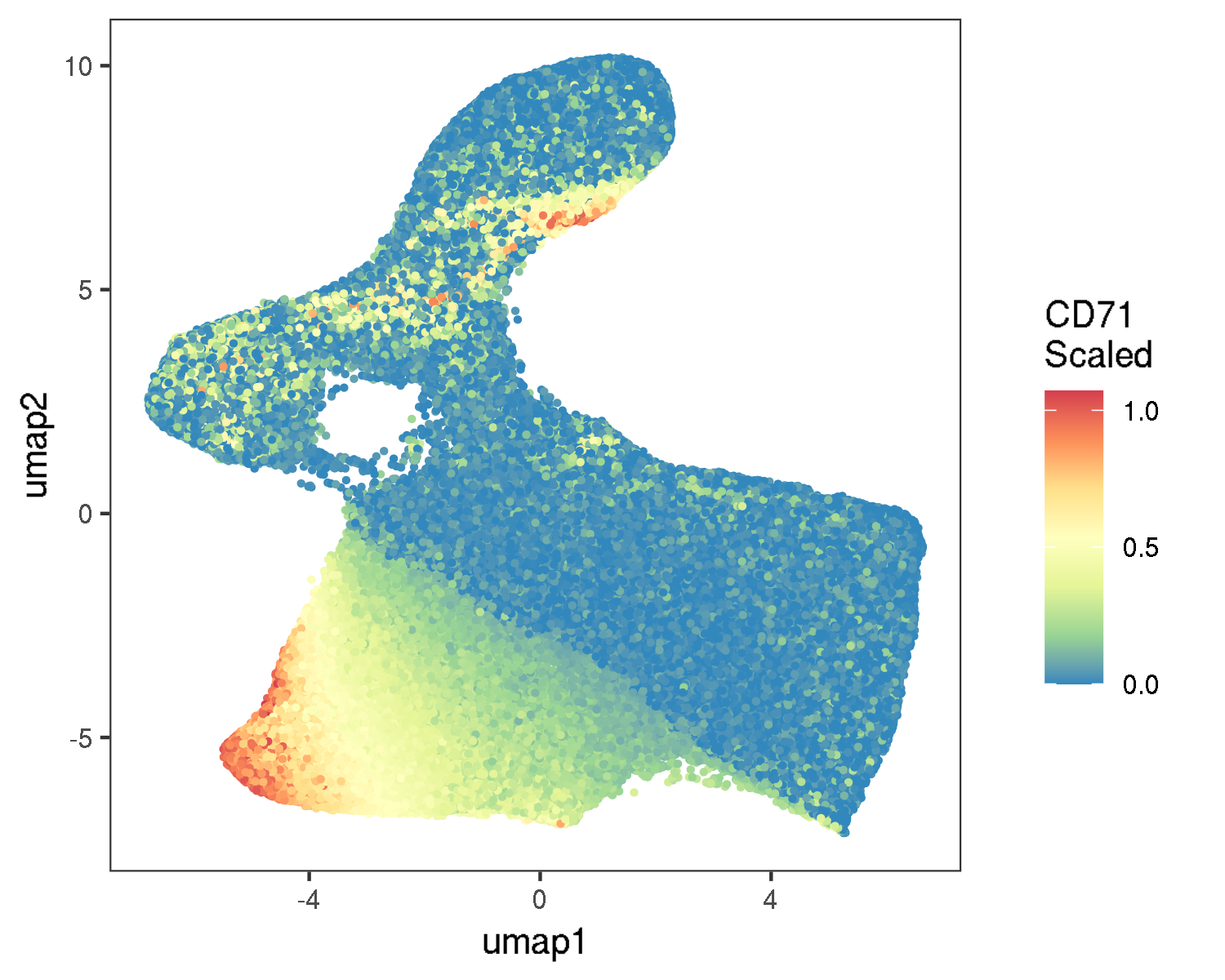

Supplement: Supplementary file 7 — Supplementary Data 4 [file 41467_2024_49883_MOESM7_ESM.zip › png/CD71.png]

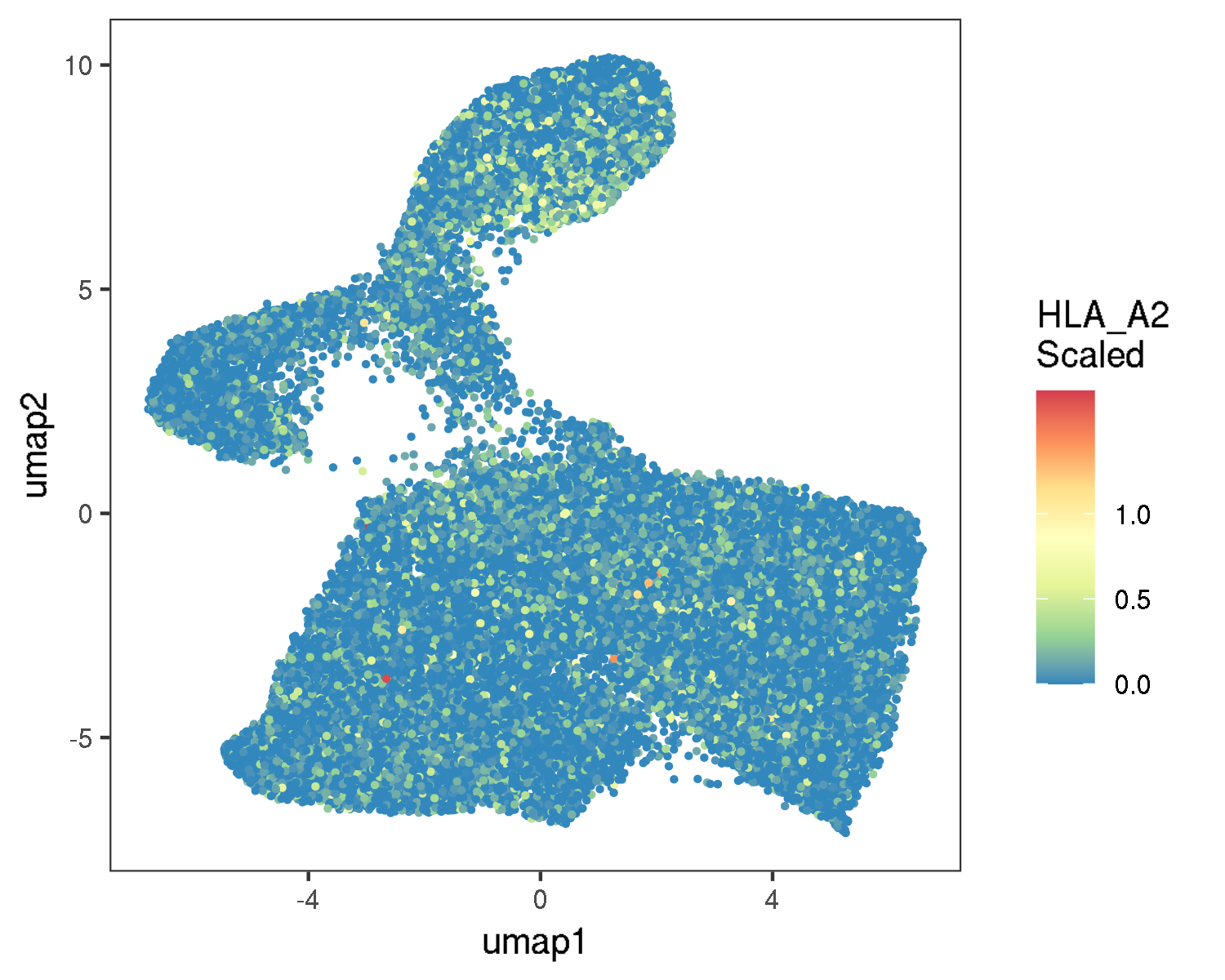

Supplement: Supplementary file 7 — Supplementary Data 4 [file 41467_2024_49883_MOESM7_ESM.zip › png/HLA_A2.png]

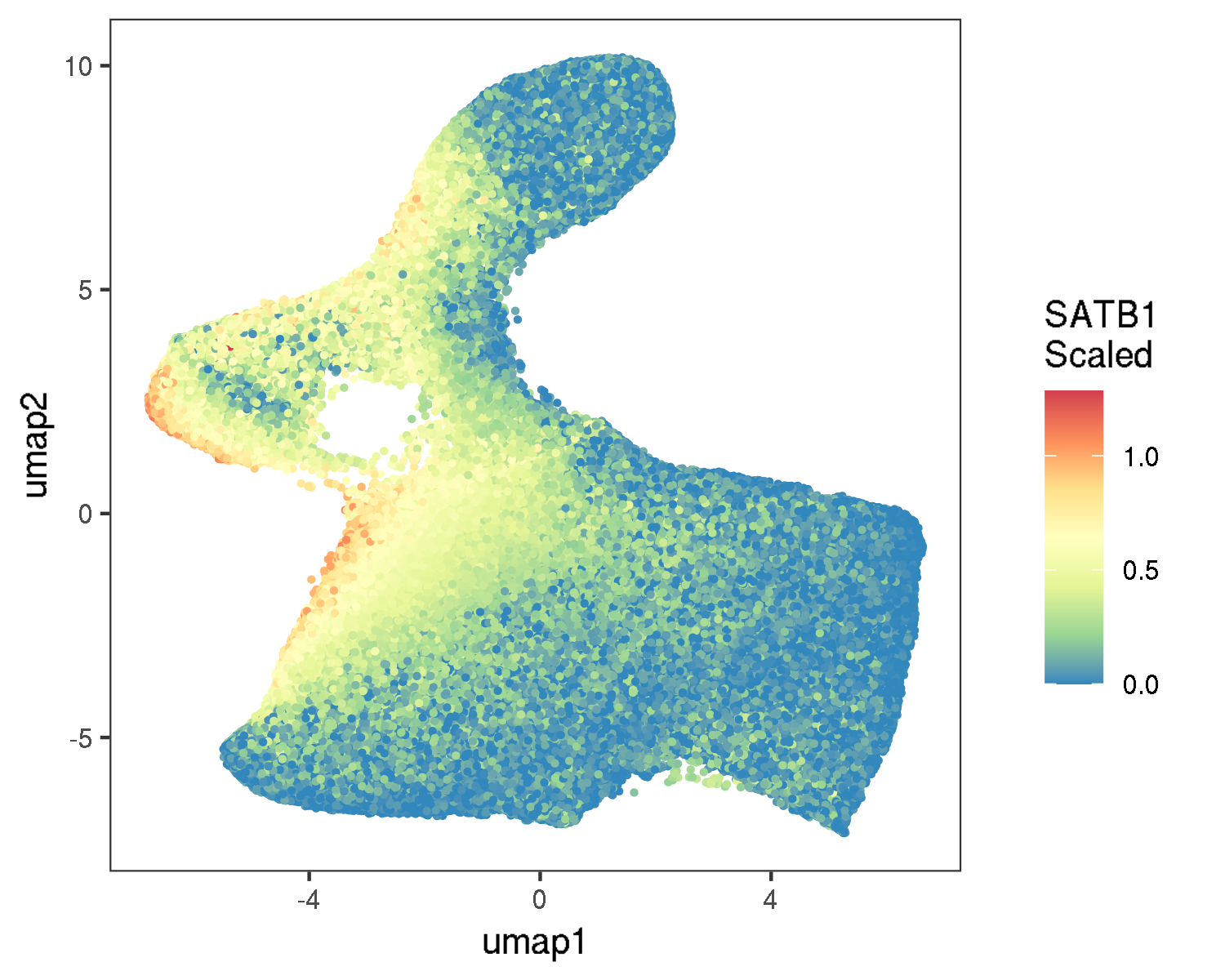

Supplement: Supplementary file 7 — Supplementary Data 4 [file 41467_2024_49883_MOESM7_ESM.zip › png/SATB1.png]

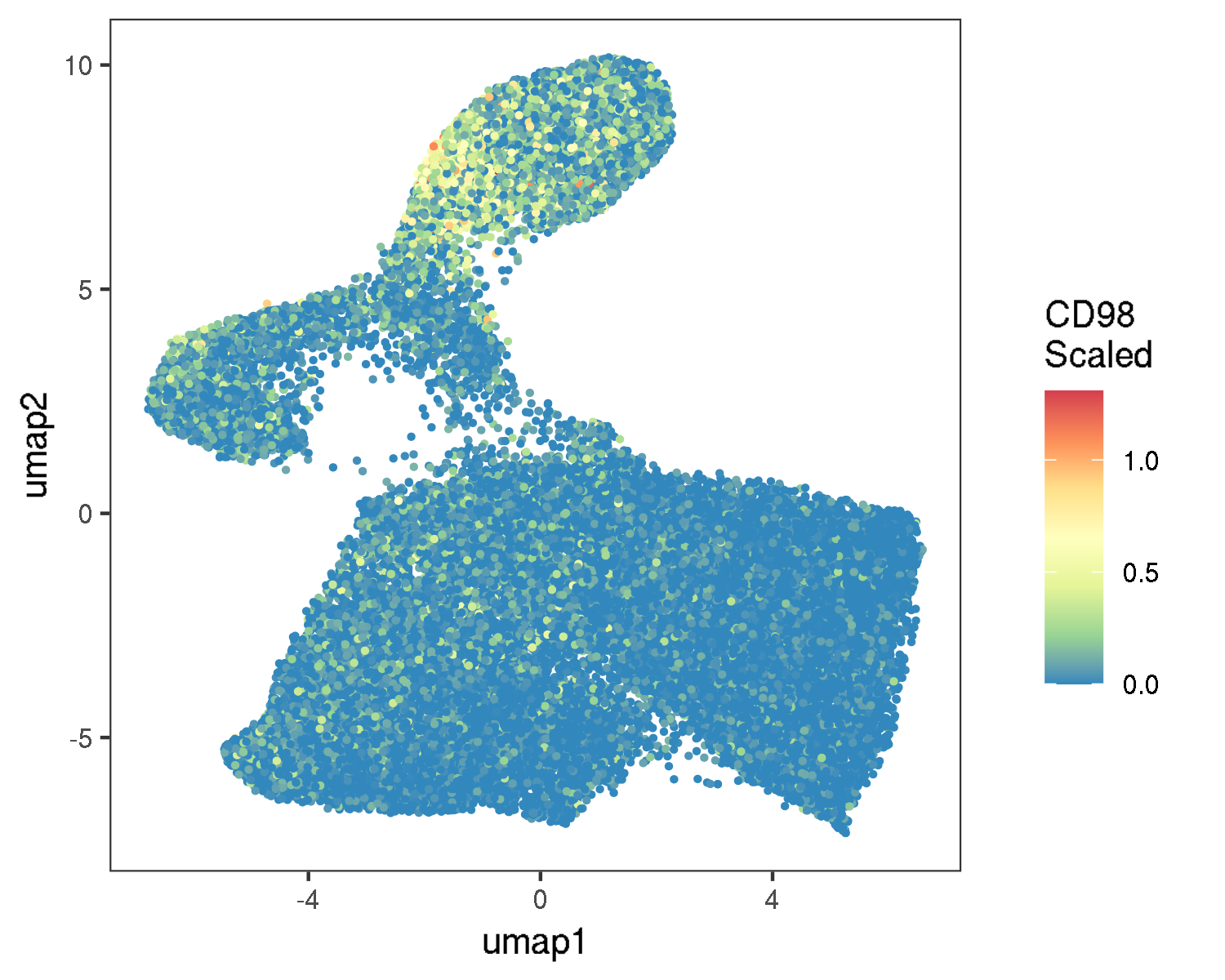

Supplement: Supplementary file 7 — Supplementary Data 4 [file 41467_2024_49883_MOESM7_ESM.zip › png/CD98.png]

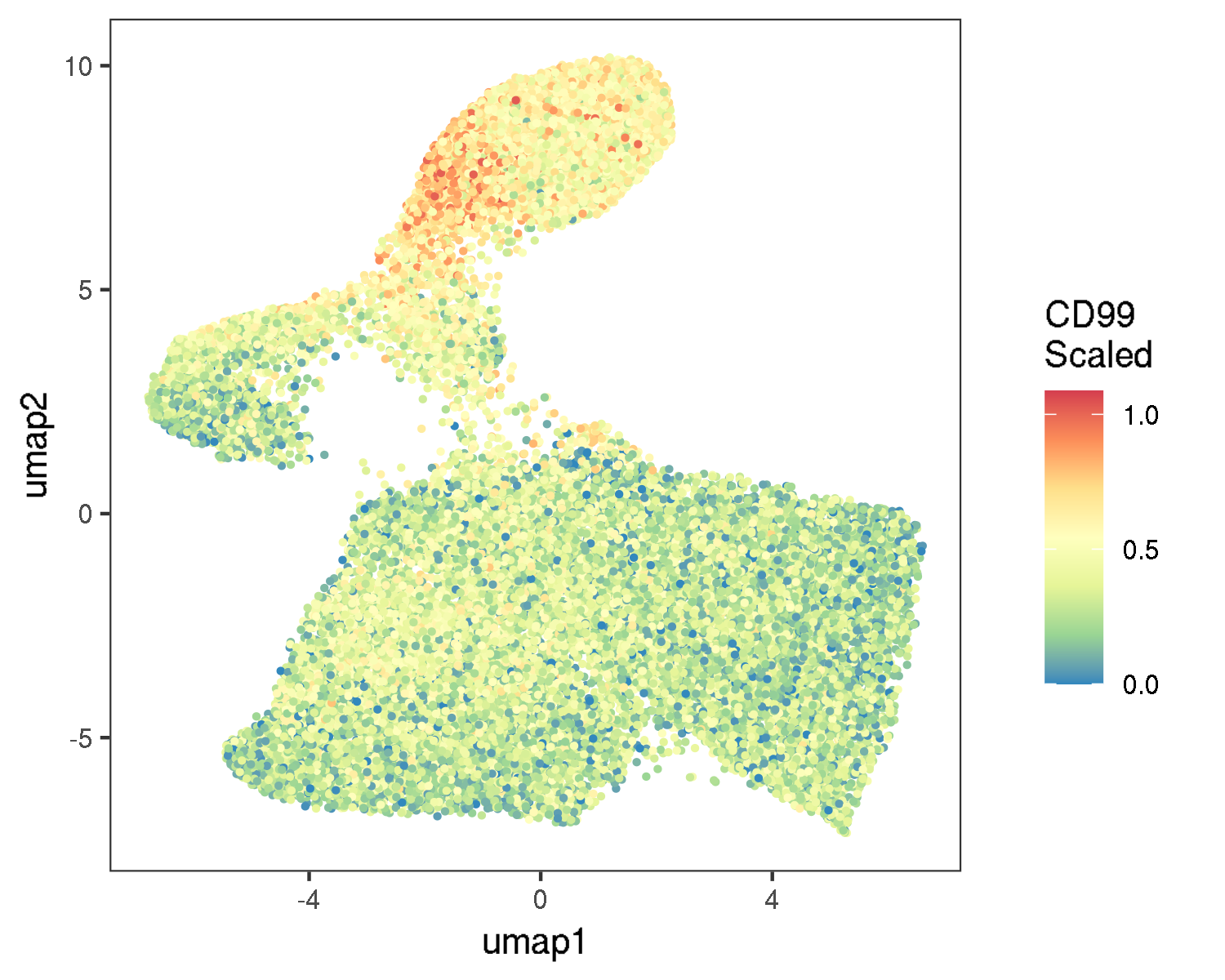

Supplement: Supplementary file 7 — Supplementary Data 4 [file 41467_2024_49883_MOESM7_ESM.zip › png/CD99.png]

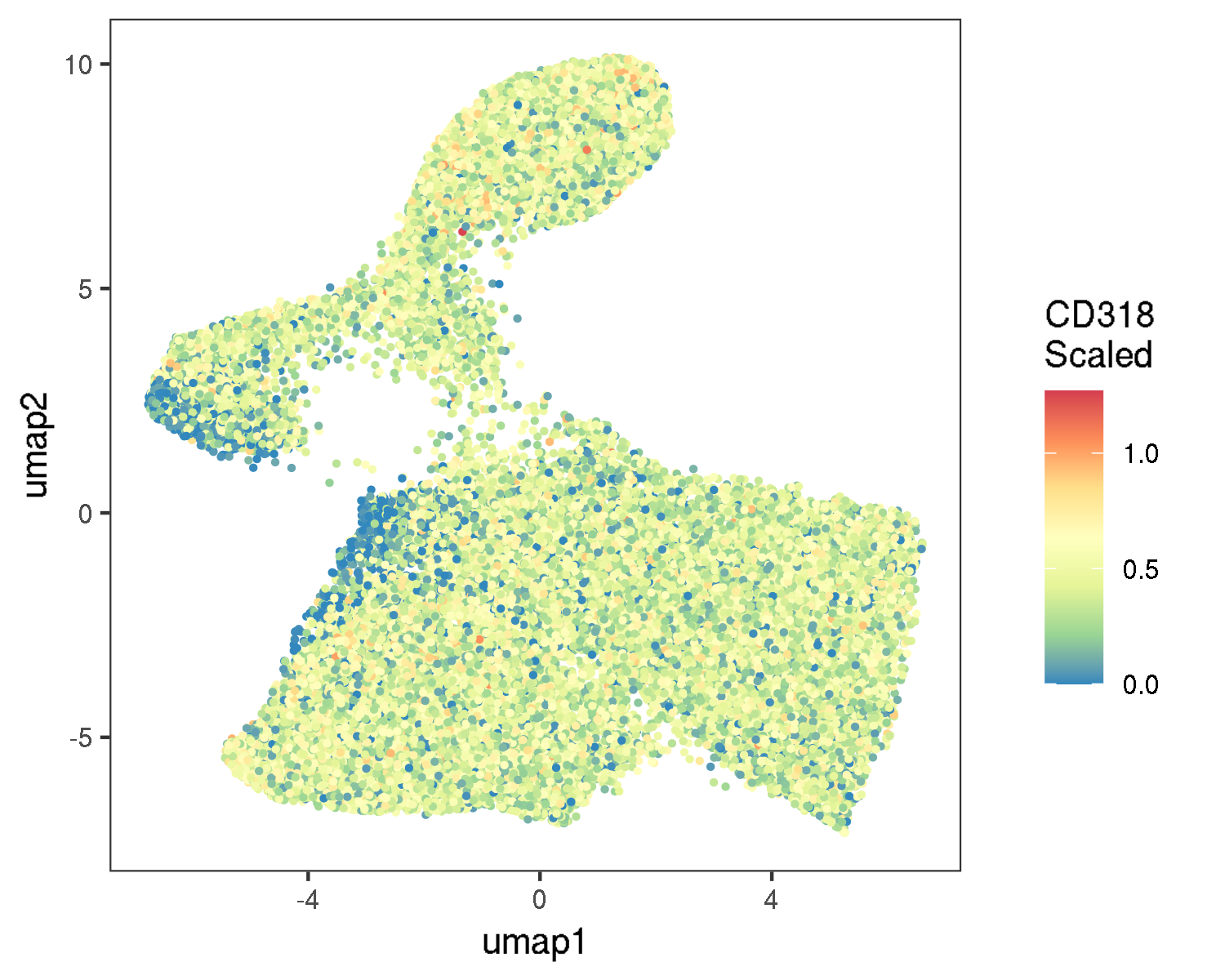

Supplement: Supplementary file 7 — Supplementary Data 4 [file 41467_2024_49883_MOESM7_ESM.zip › png/CD318.png]

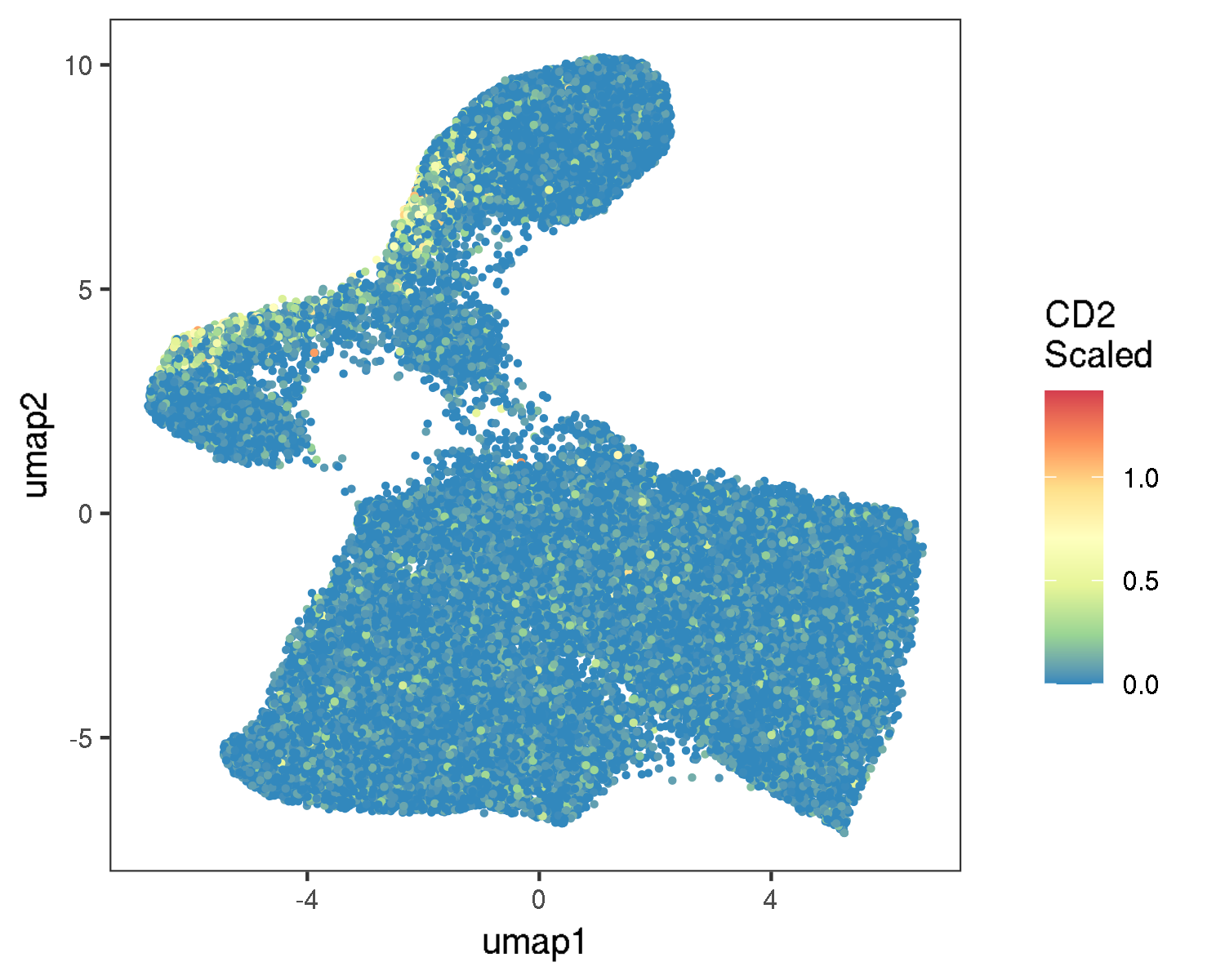

Supplement: Supplementary file 7 — Supplementary Data 4 [file 41467_2024_49883_MOESM7_ESM.zip › png/CD2.png]

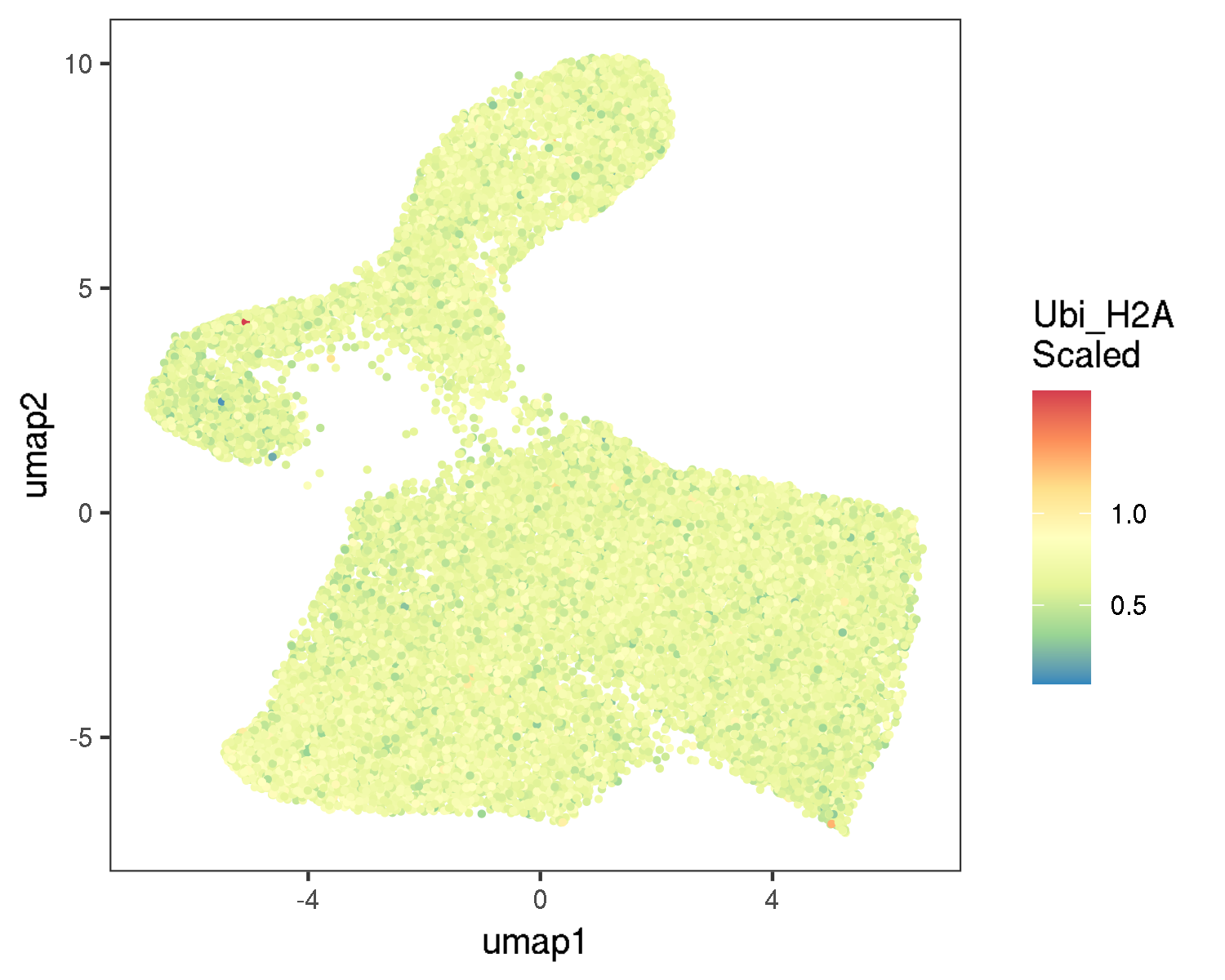

Supplement: Supplementary file 7 — Supplementary Data 4 [file 41467_2024_49883_MOESM7_ESM.zip › png/Ubi_H2A.png]

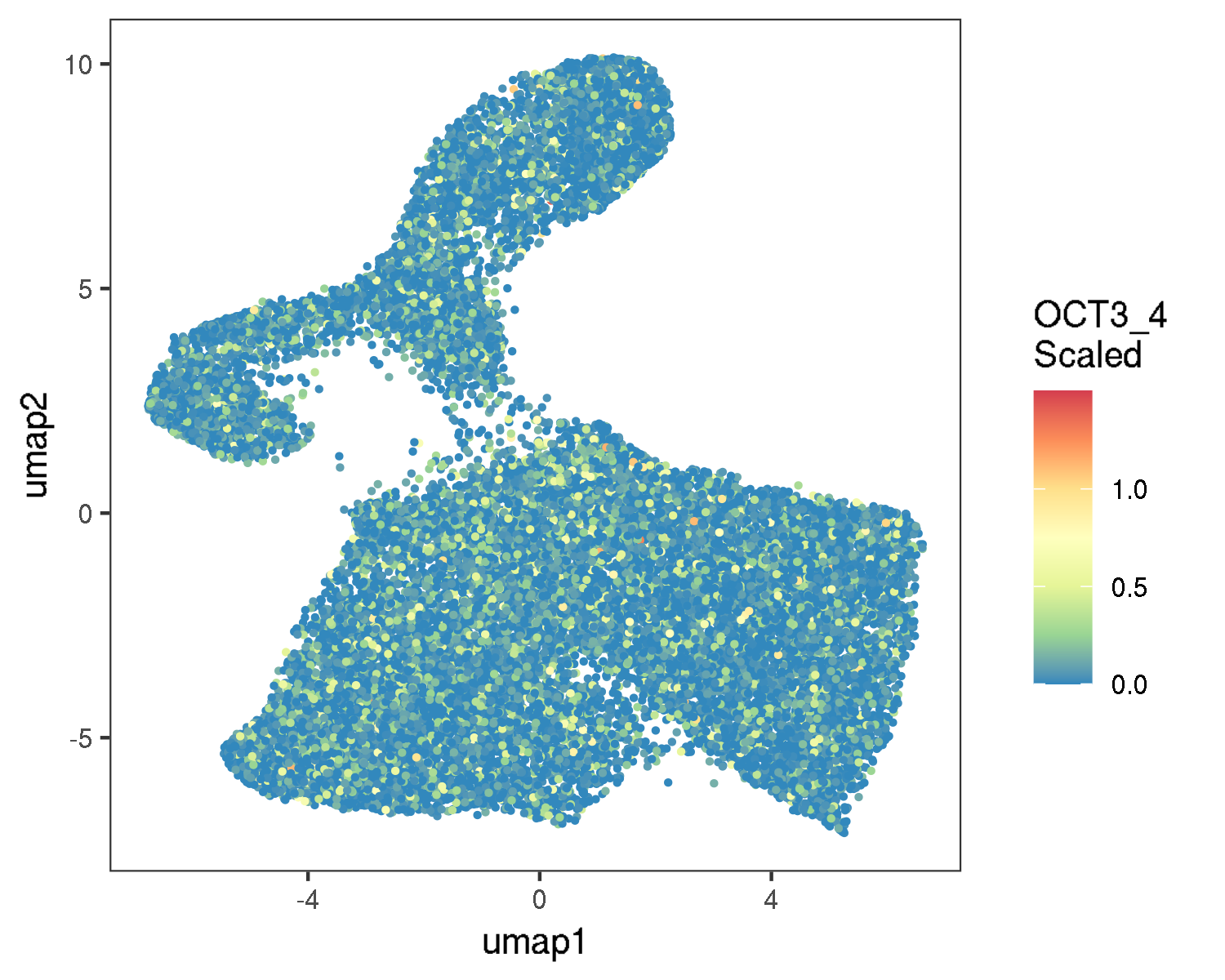

Supplement: Supplementary file 7 — Supplementary Data 4 [file 41467_2024_49883_MOESM7_ESM.zip › png/OCT3_4.png]

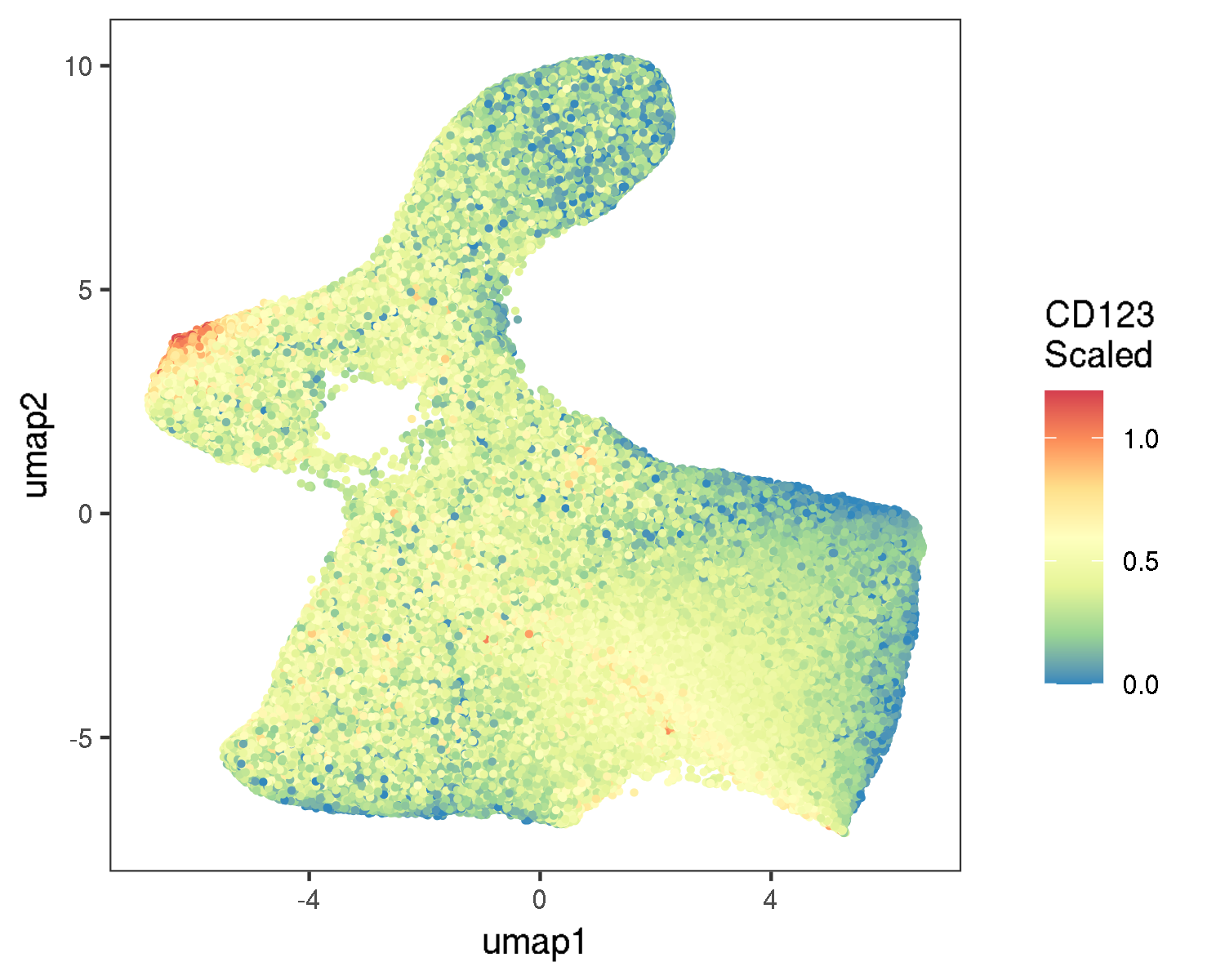

Supplement: Supplementary file 7 — Supplementary Data 4 [file 41467_2024_49883_MOESM7_ESM.zip › png/CD123.png]

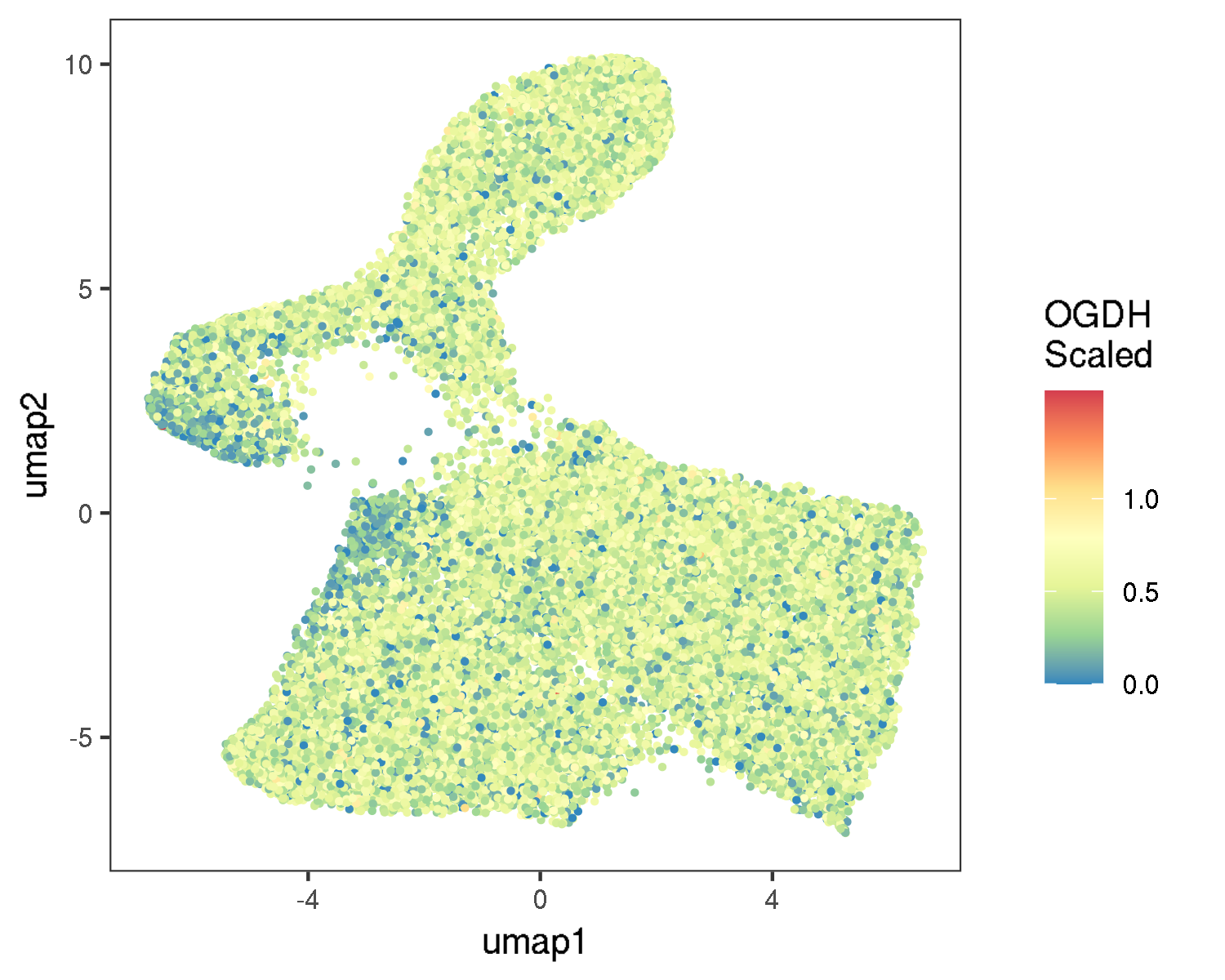

Supplement: Supplementary file 7 — Supplementary Data 4 [file 41467_2024_49883_MOESM7_ESM.zip › png/OGDH.png]

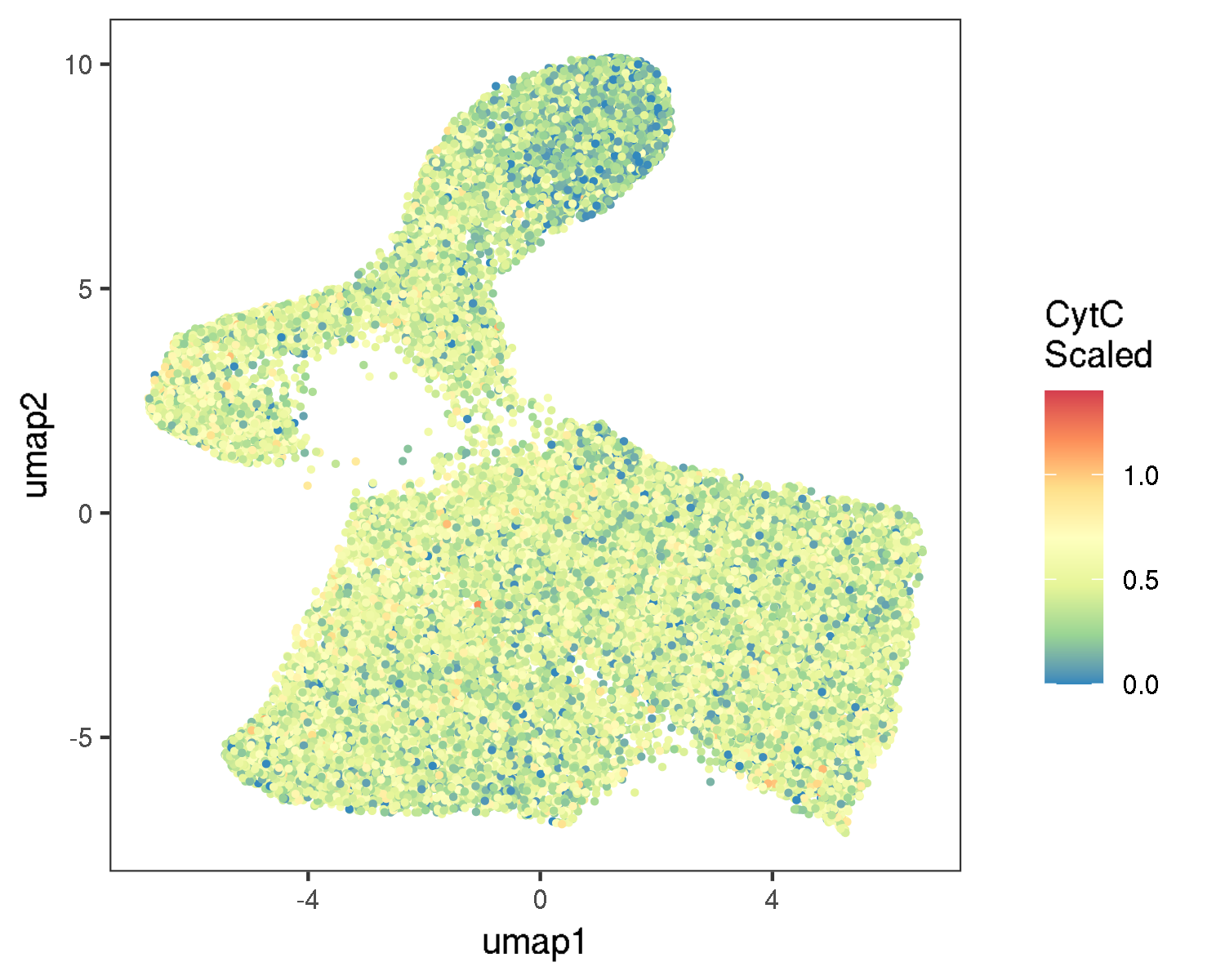

Supplement: Supplementary file 7 — Supplementary Data 4 [file 41467_2024_49883_MOESM7_ESM.zip › png/CytC.png]

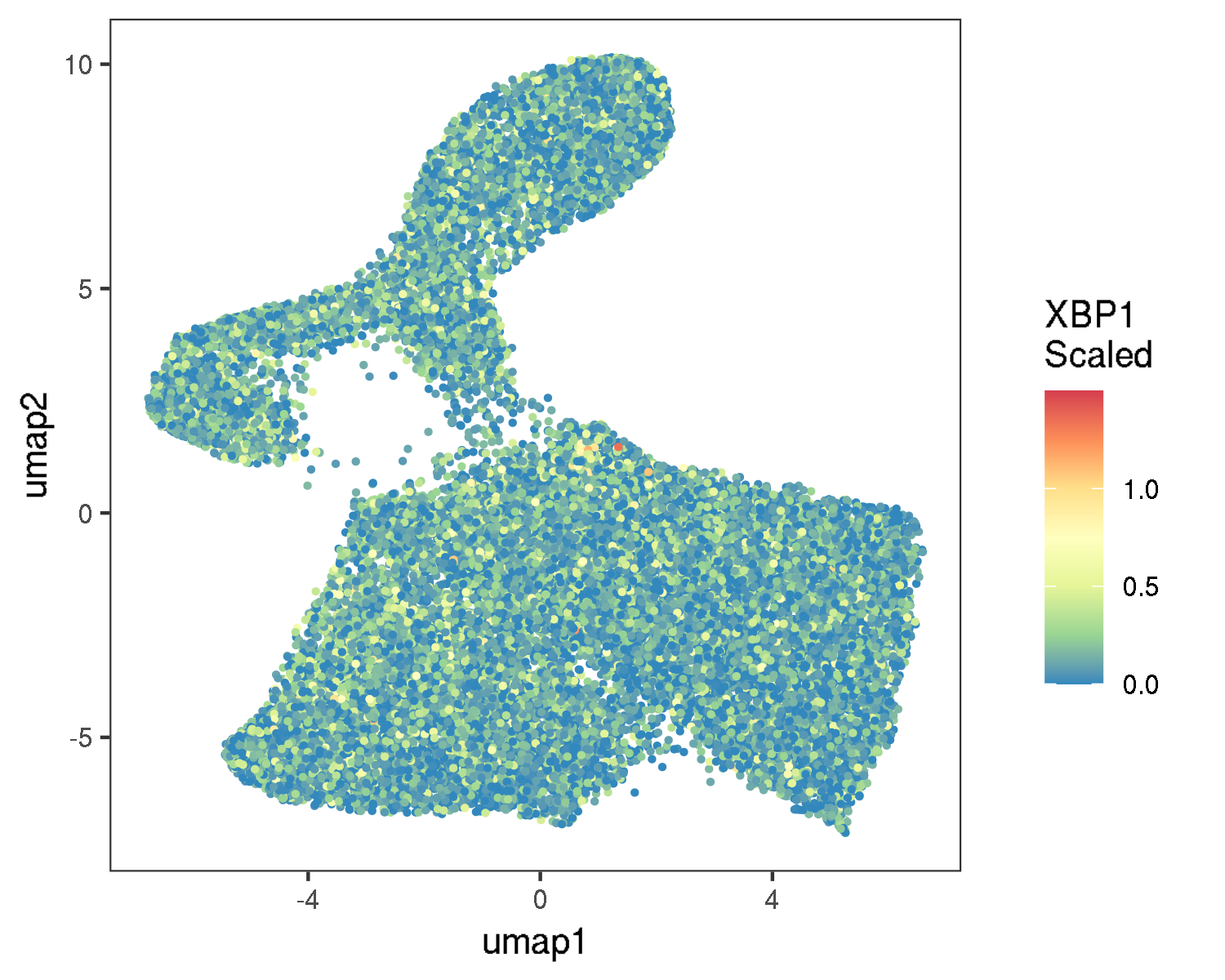

Supplement: Supplementary file 7 — Supplementary Data 4 [file 41467_2024_49883_MOESM7_ESM.zip › png/XBP1.png]

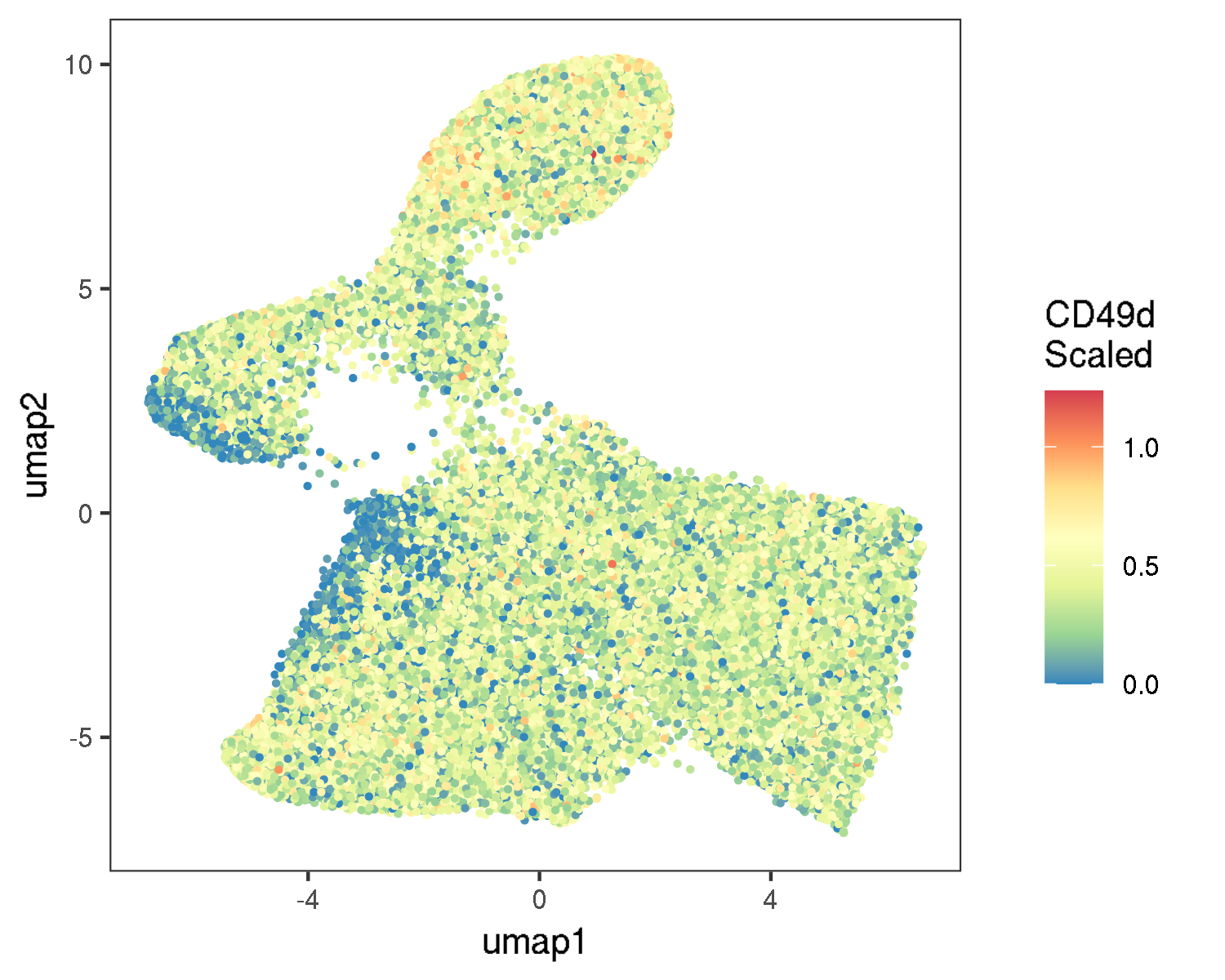

Supplement: Supplementary file 7 — Supplementary Data 4 [file 41467_2024_49883_MOESM7_ESM.zip › png/CD49d.png]

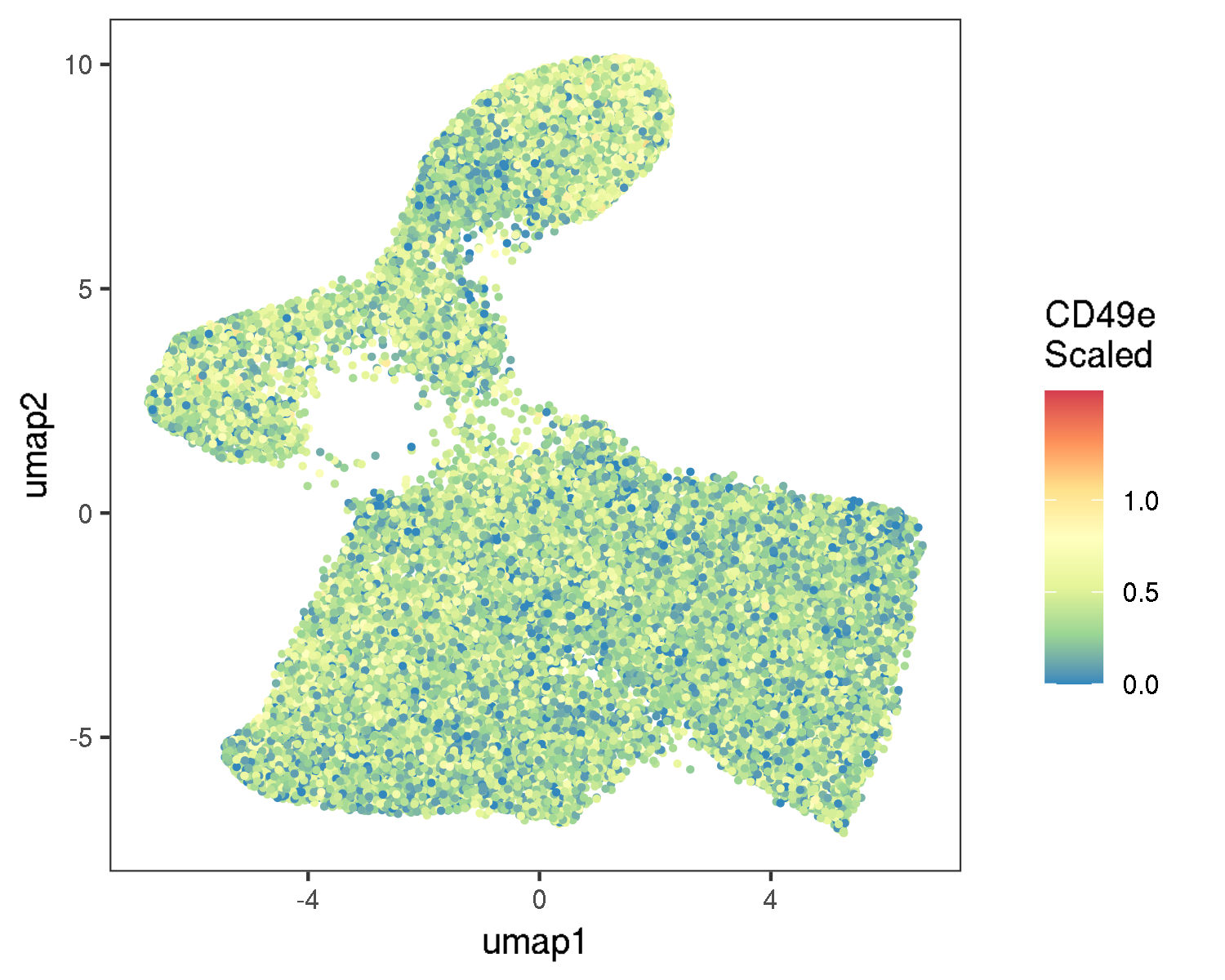

Supplement: Supplementary file 7 — Supplementary Data 4 [file 41467_2024_49883_MOESM7_ESM.zip › png/CD49e.png]

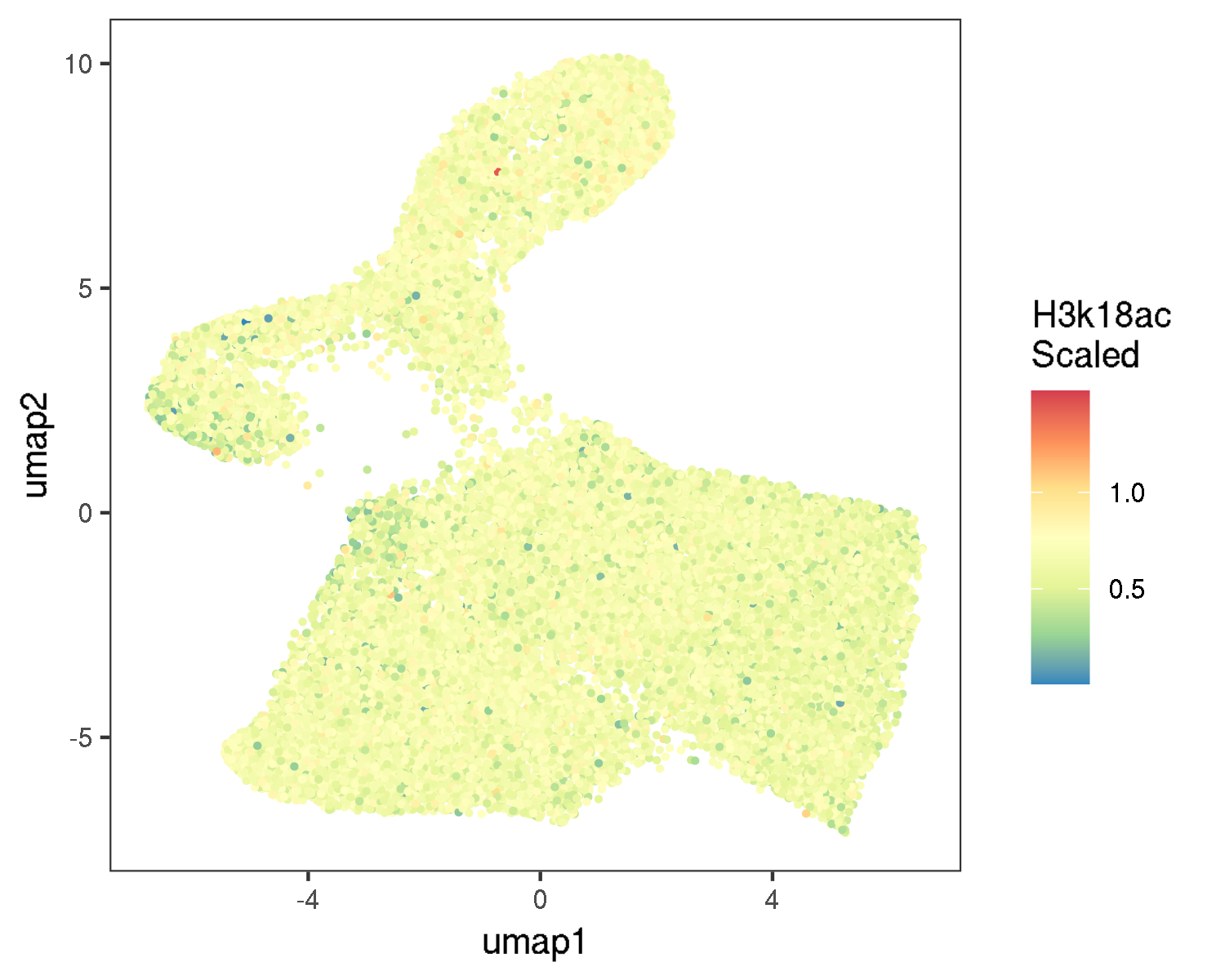

Supplement: Supplementary file 7 — Supplementary Data 4 [file 41467_2024_49883_MOESM7_ESM.zip › png/H3k18ac.png]

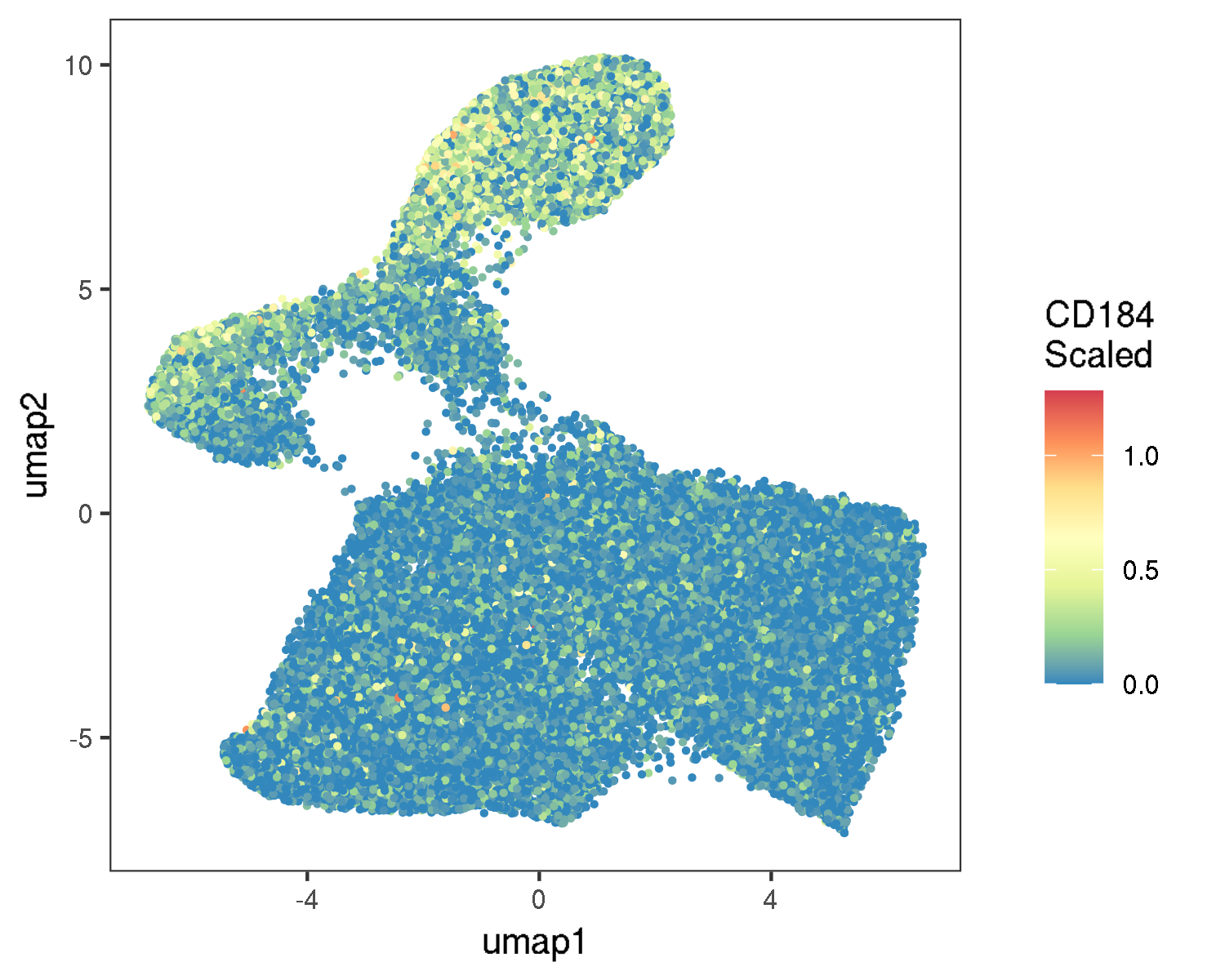

Supplement: Supplementary file 7 — Supplementary Data 4 [file 41467_2024_49883_MOESM7_ESM.zip › png/CD184.png]

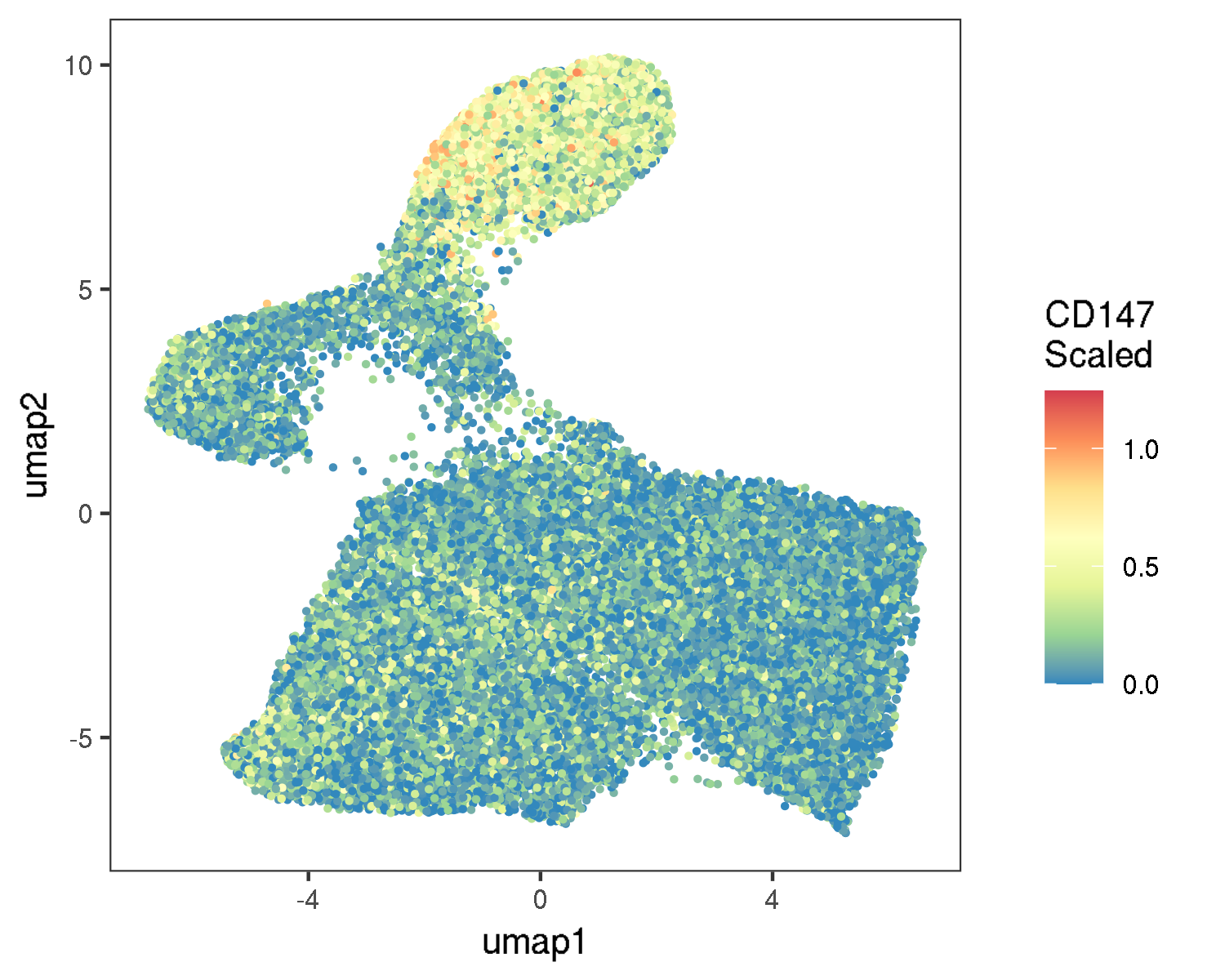

Supplement: Supplementary file 7 — Supplementary Data 4 [file 41467_2024_49883_MOESM7_ESM.zip › png/CD147.png]

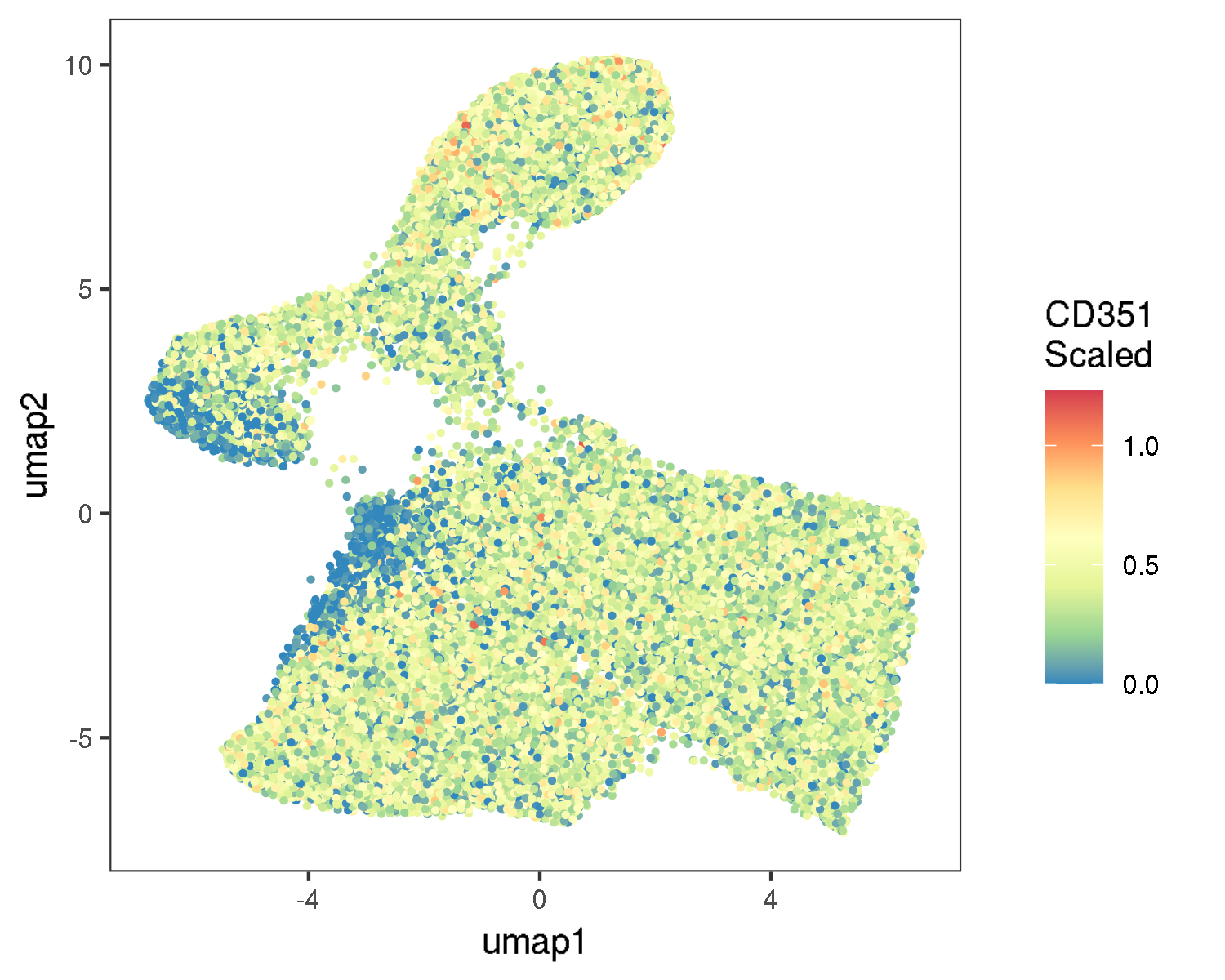

Supplement: Supplementary file 7 — Supplementary Data 4 [file 41467_2024_49883_MOESM7_ESM.zip › png/CD351.png]

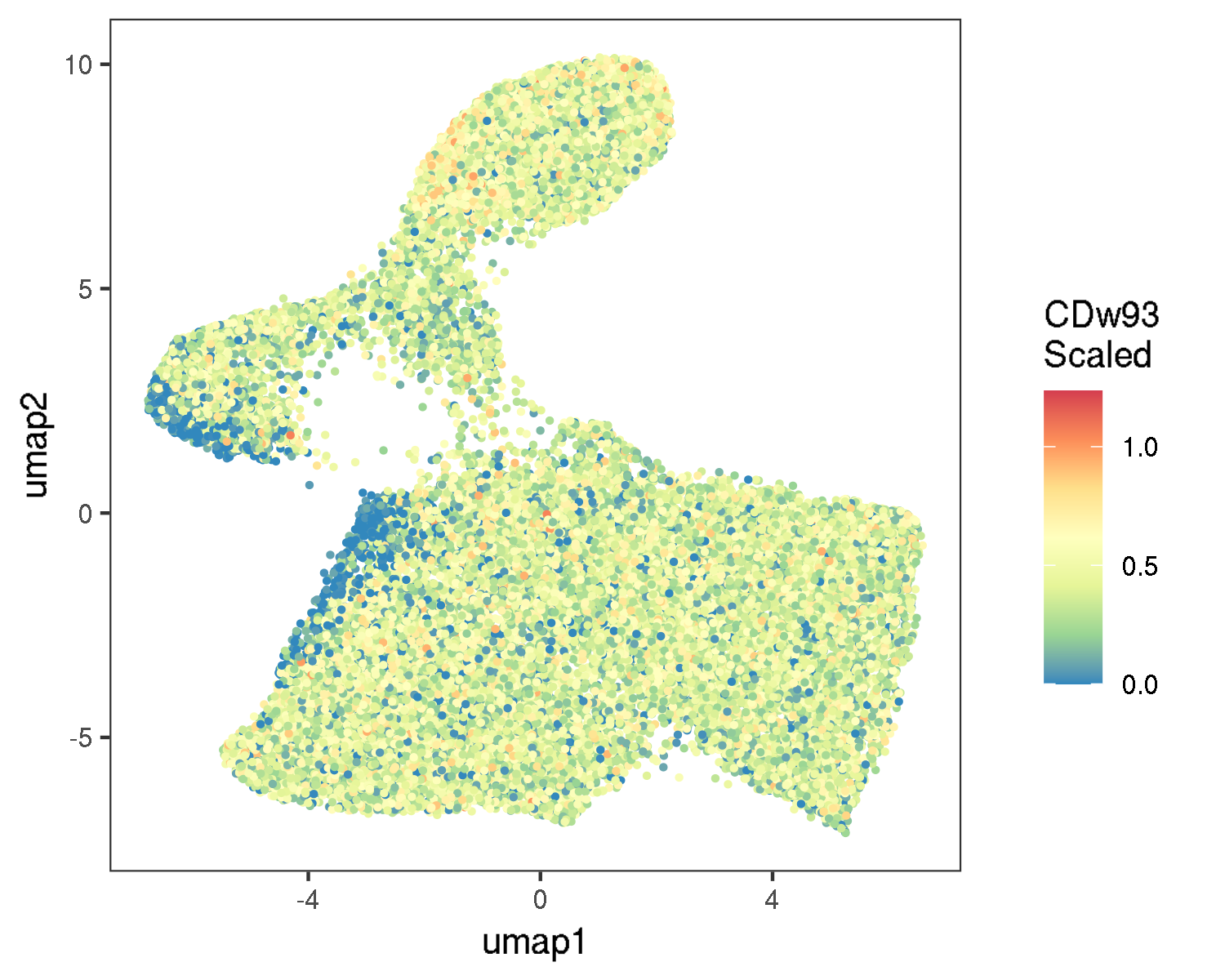

Supplement: Supplementary file 7 — Supplementary Data 4 [file 41467_2024_49883_MOESM7_ESM.zip › png/CDw93.png]

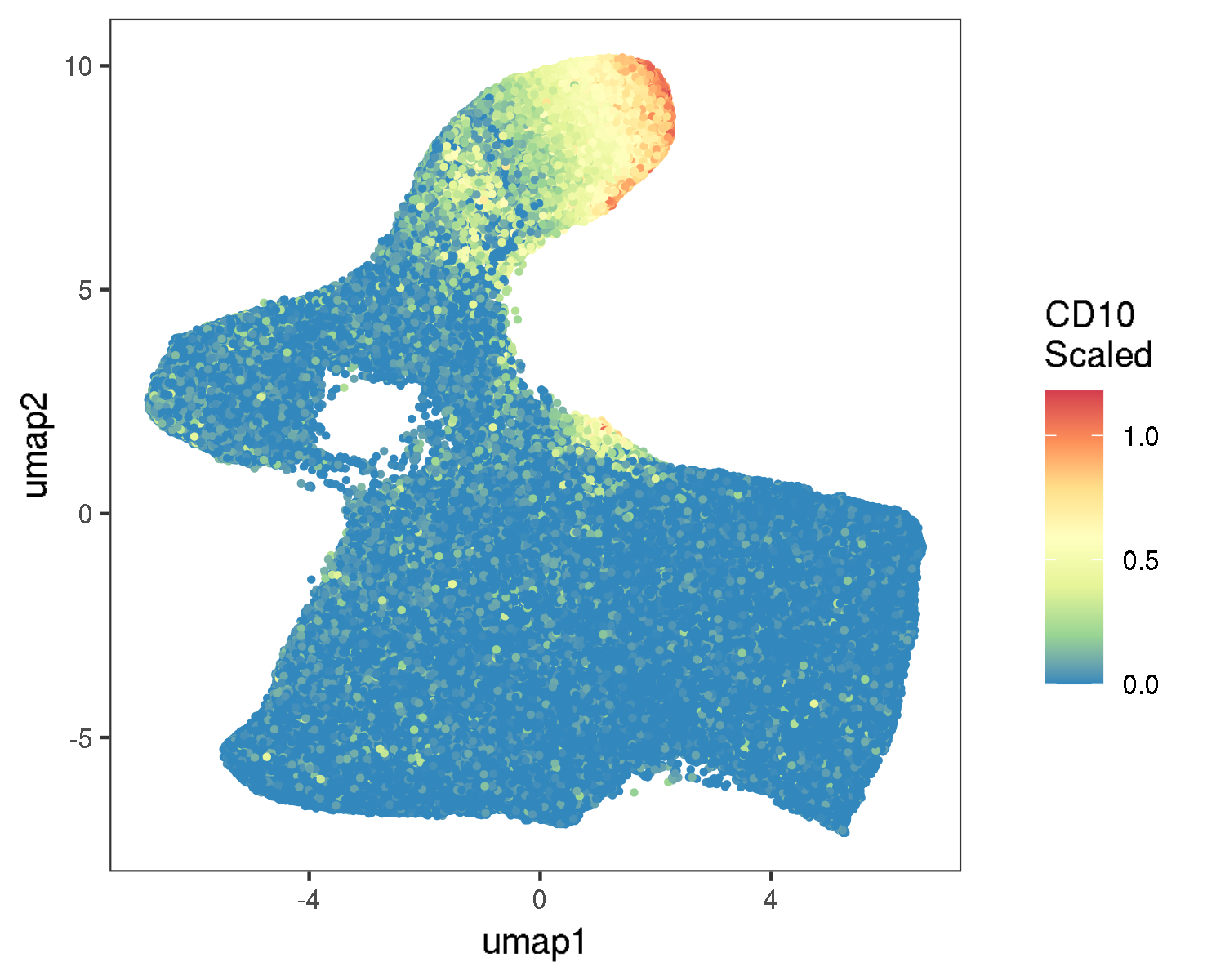

Supplement: Supplementary file 7 — Supplementary Data 4 [file 41467_2024_49883_MOESM7_ESM.zip › png/CD10.png]

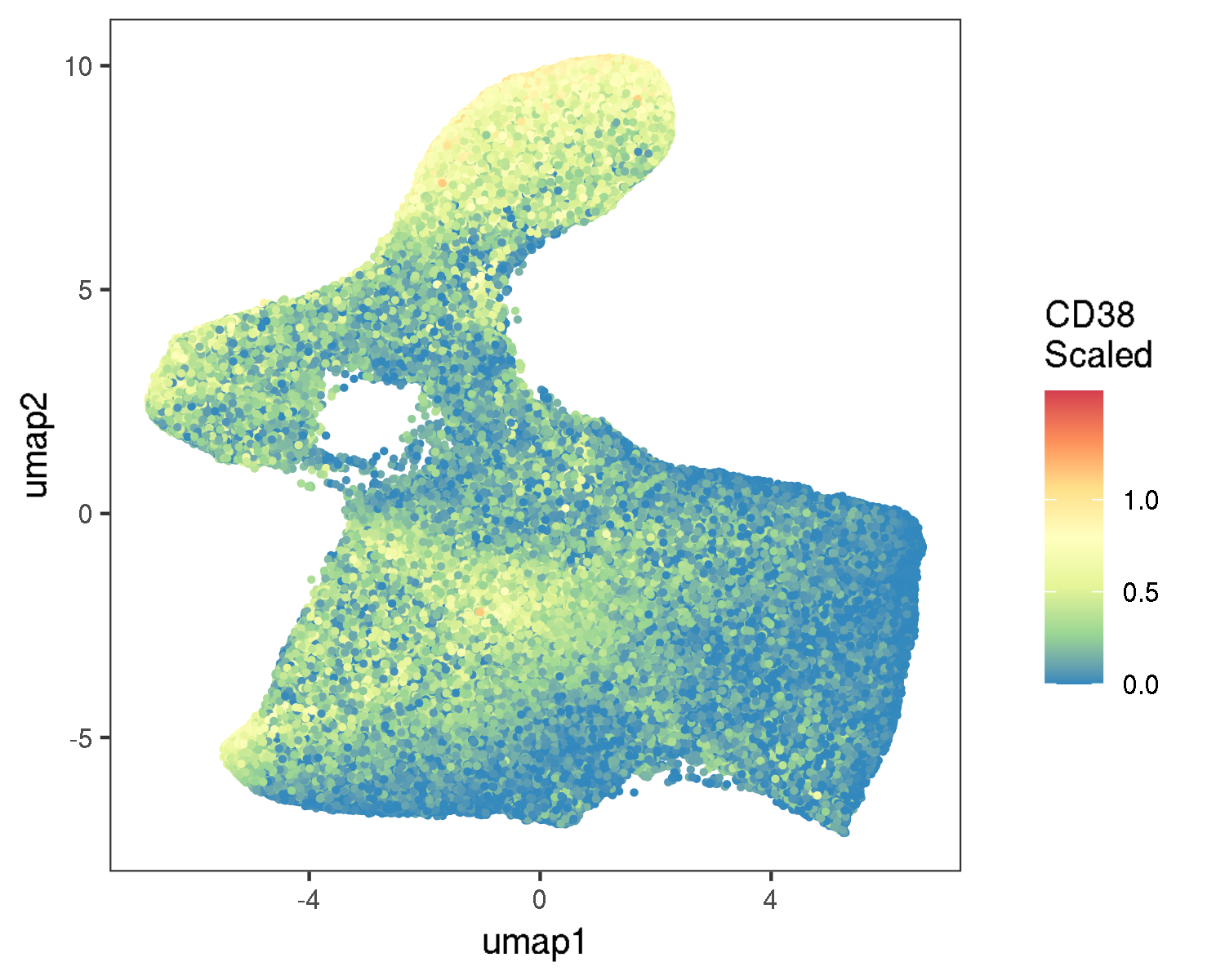

Supplement: Supplementary file 7 — Supplementary Data 4 [file 41467_2024_49883_MOESM7_ESM.zip › png/CD38.png]

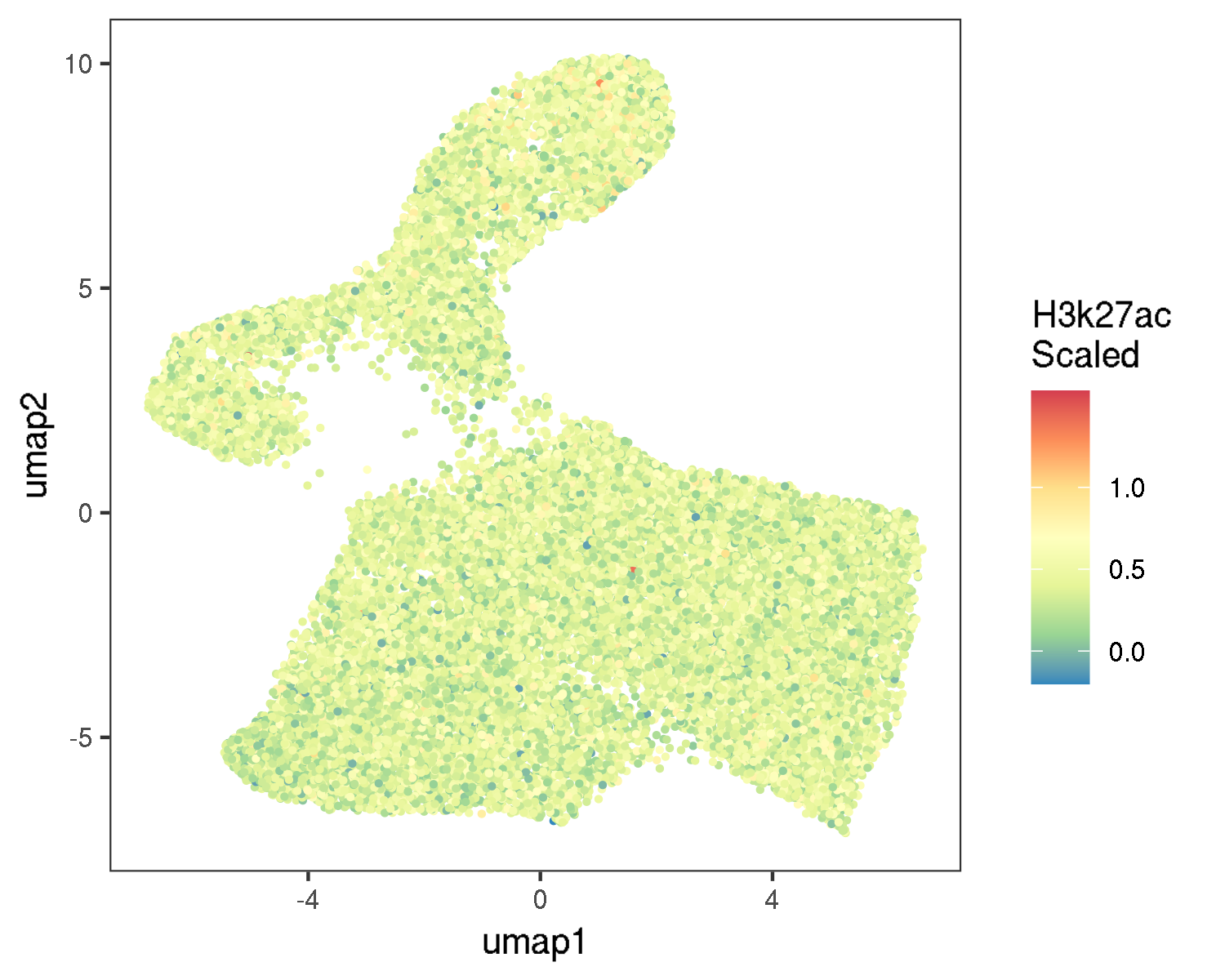

Supplement: Supplementary file 7 — Supplementary Data 4 [file 41467_2024_49883_MOESM7_ESM.zip › png/H3k27ac.png]

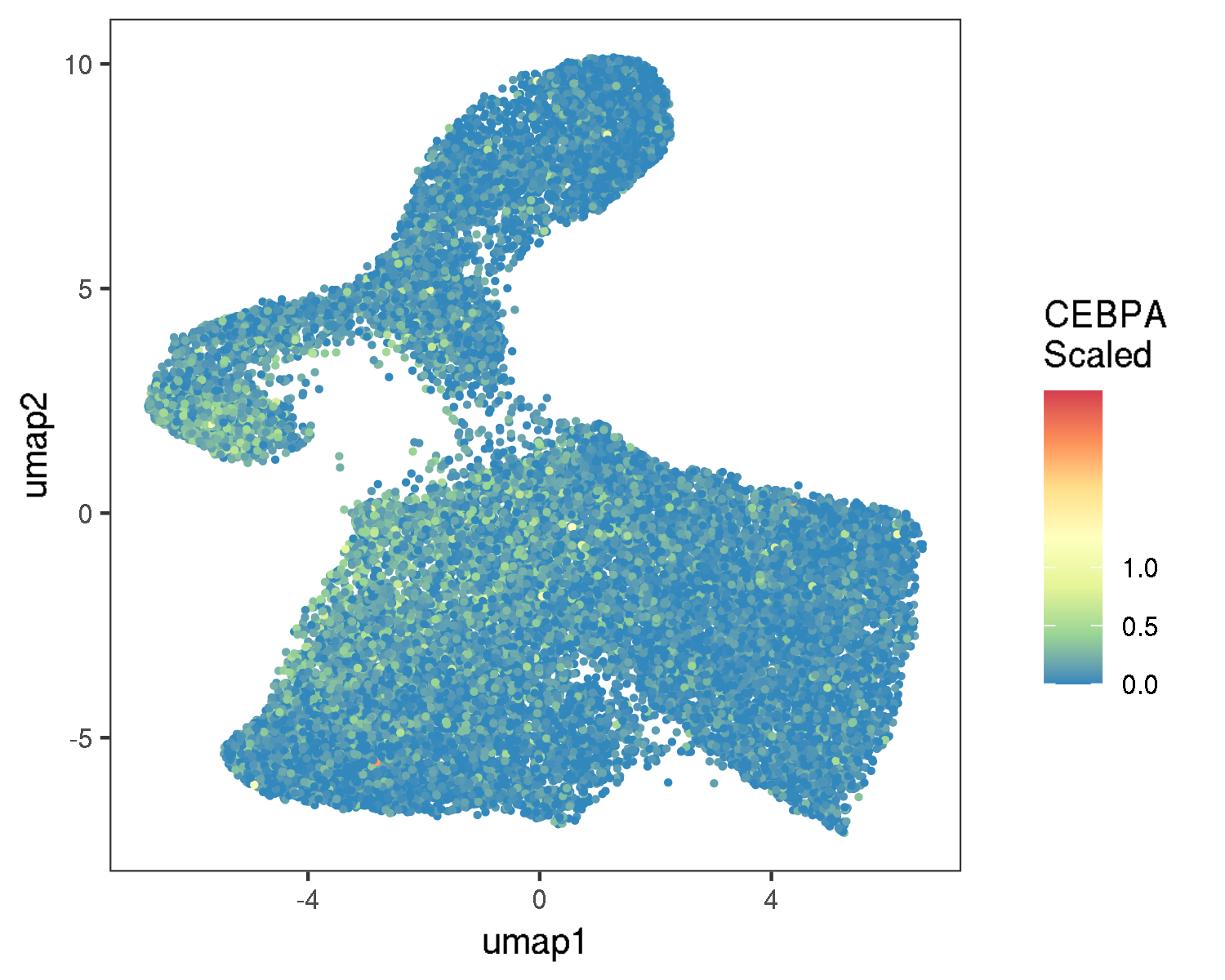

Supplement: Supplementary file 7 — Supplementary Data 4 [file 41467_2024_49883_MOESM7_ESM.zip › png/CEBPA.png]

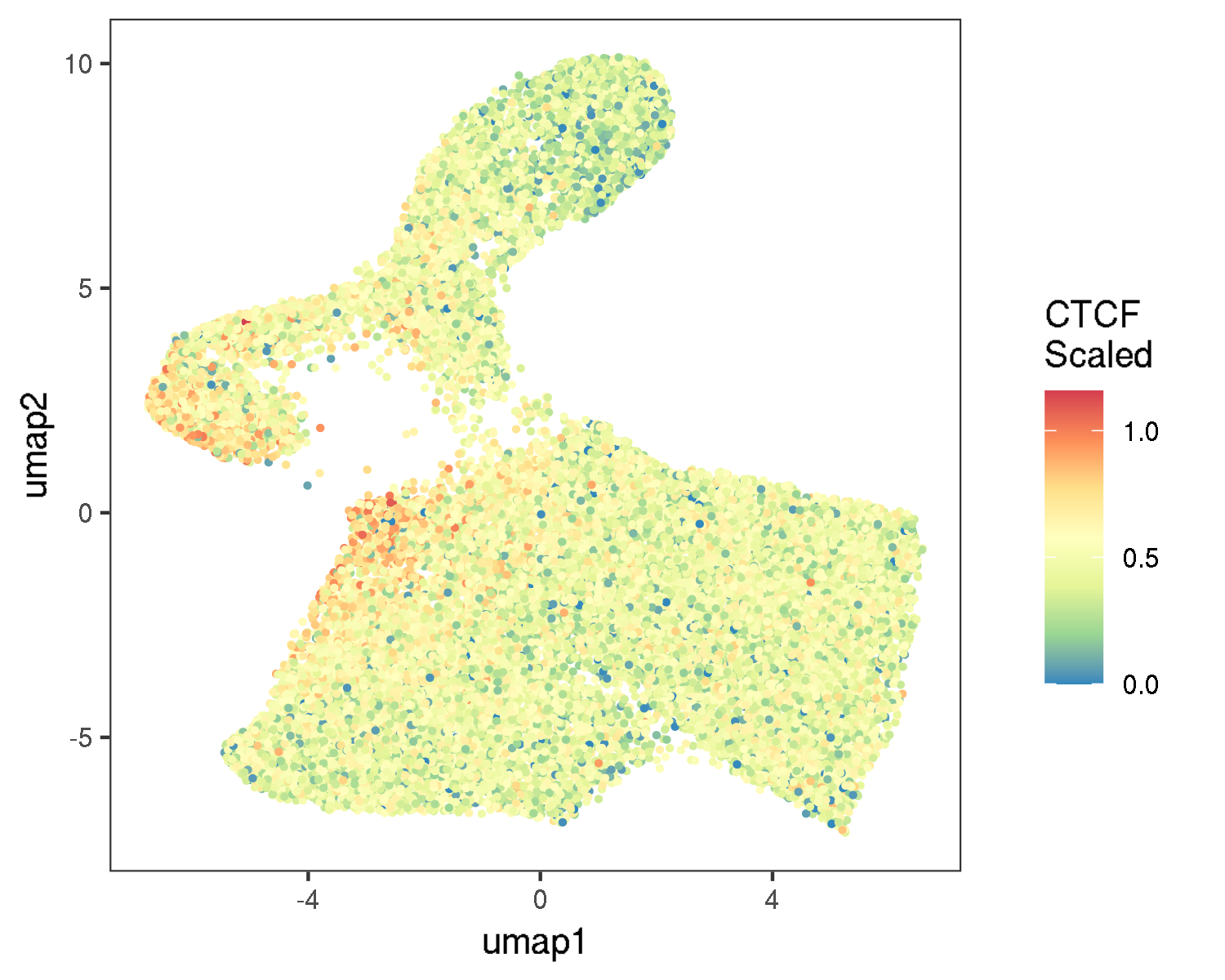

Supplement: Supplementary file 7 — Supplementary Data 4 [file 41467_2024_49883_MOESM7_ESM.zip › png/CTCF.png]

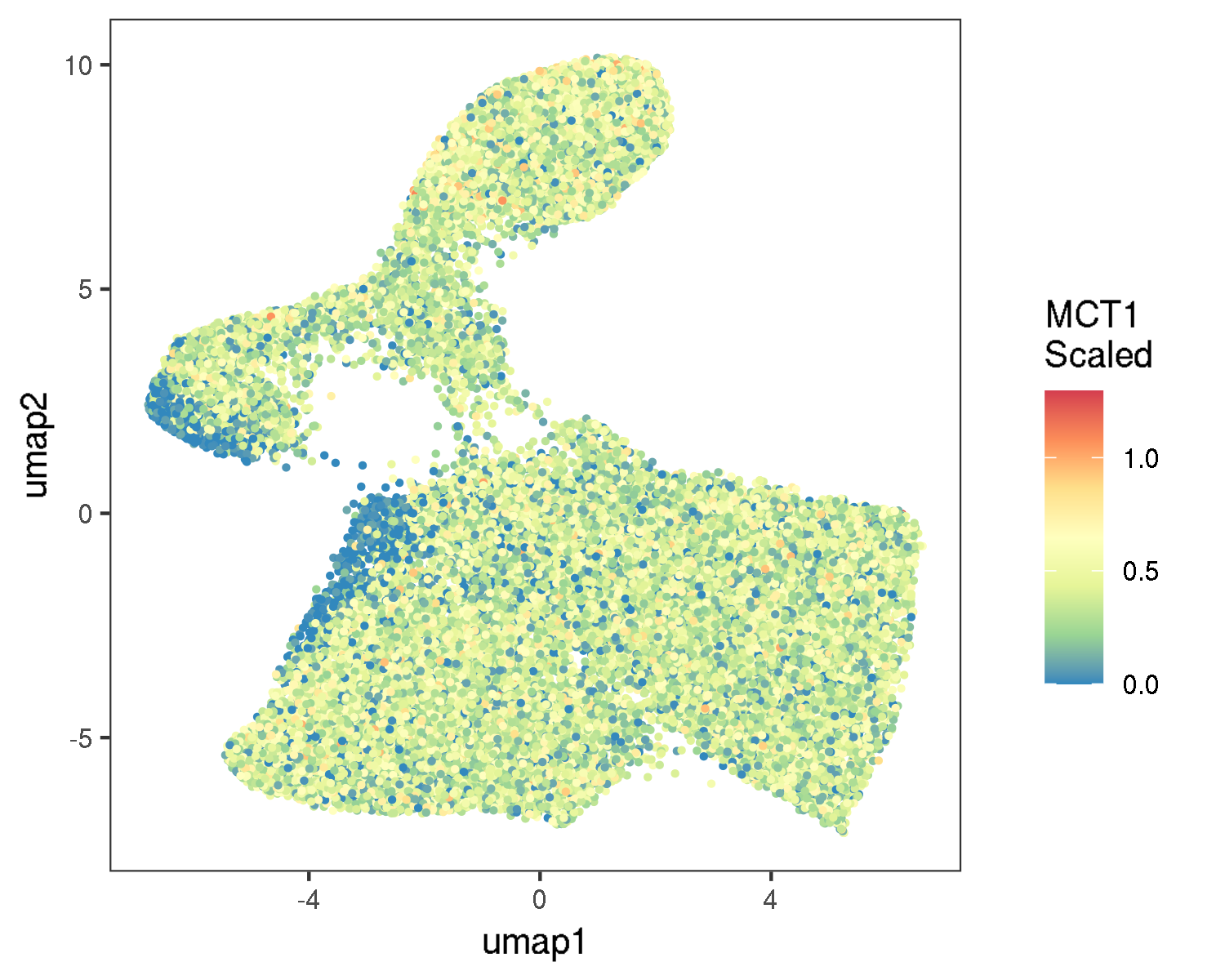

Supplement: Supplementary file 7 — Supplementary Data 4 [file 41467_2024_49883_MOESM7_ESM.zip › png/MCT1.png]

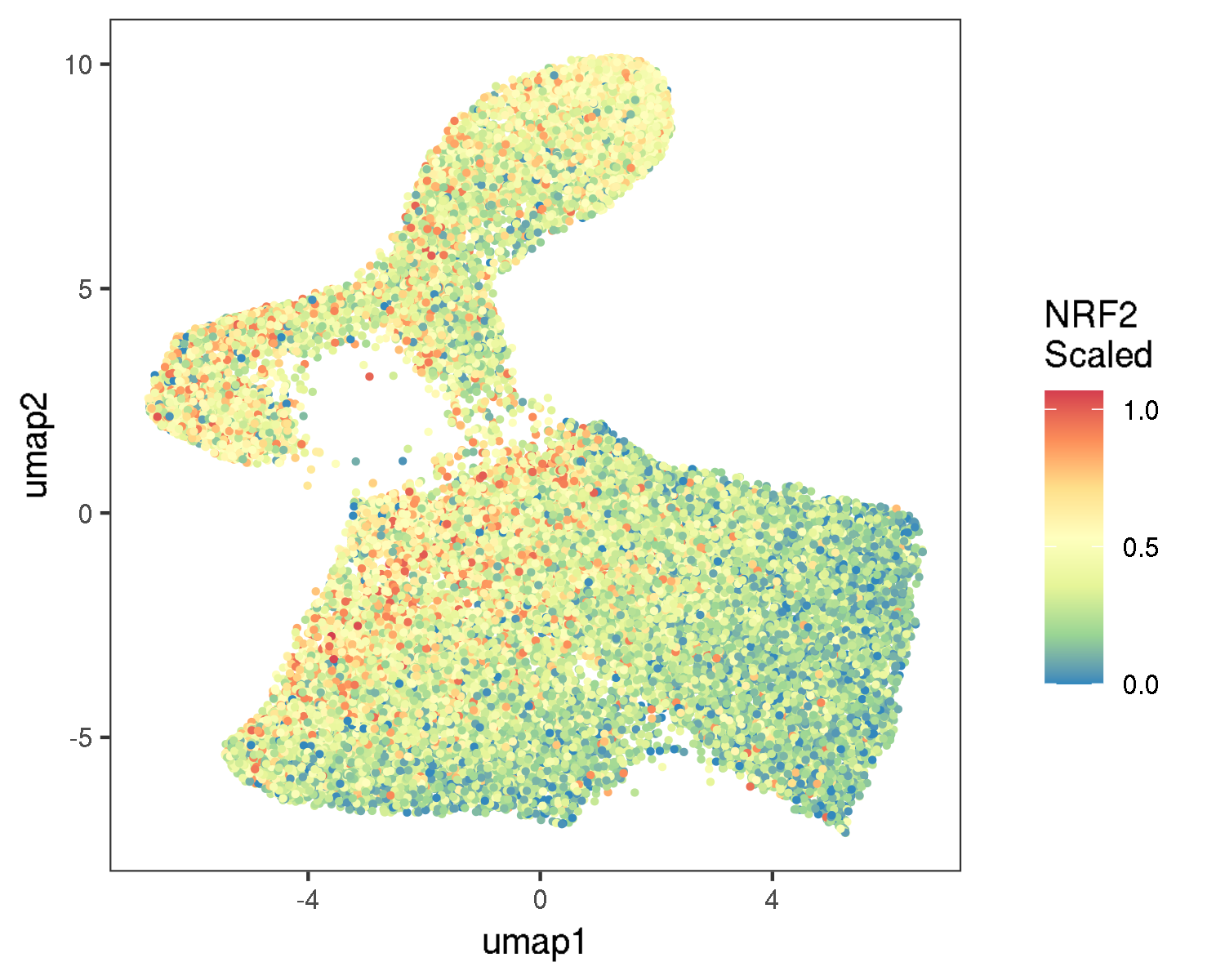

Supplement: Supplementary file 7 — Supplementary Data 4 [file 41467_2024_49883_MOESM7_ESM.zip › png/NRF2.png]

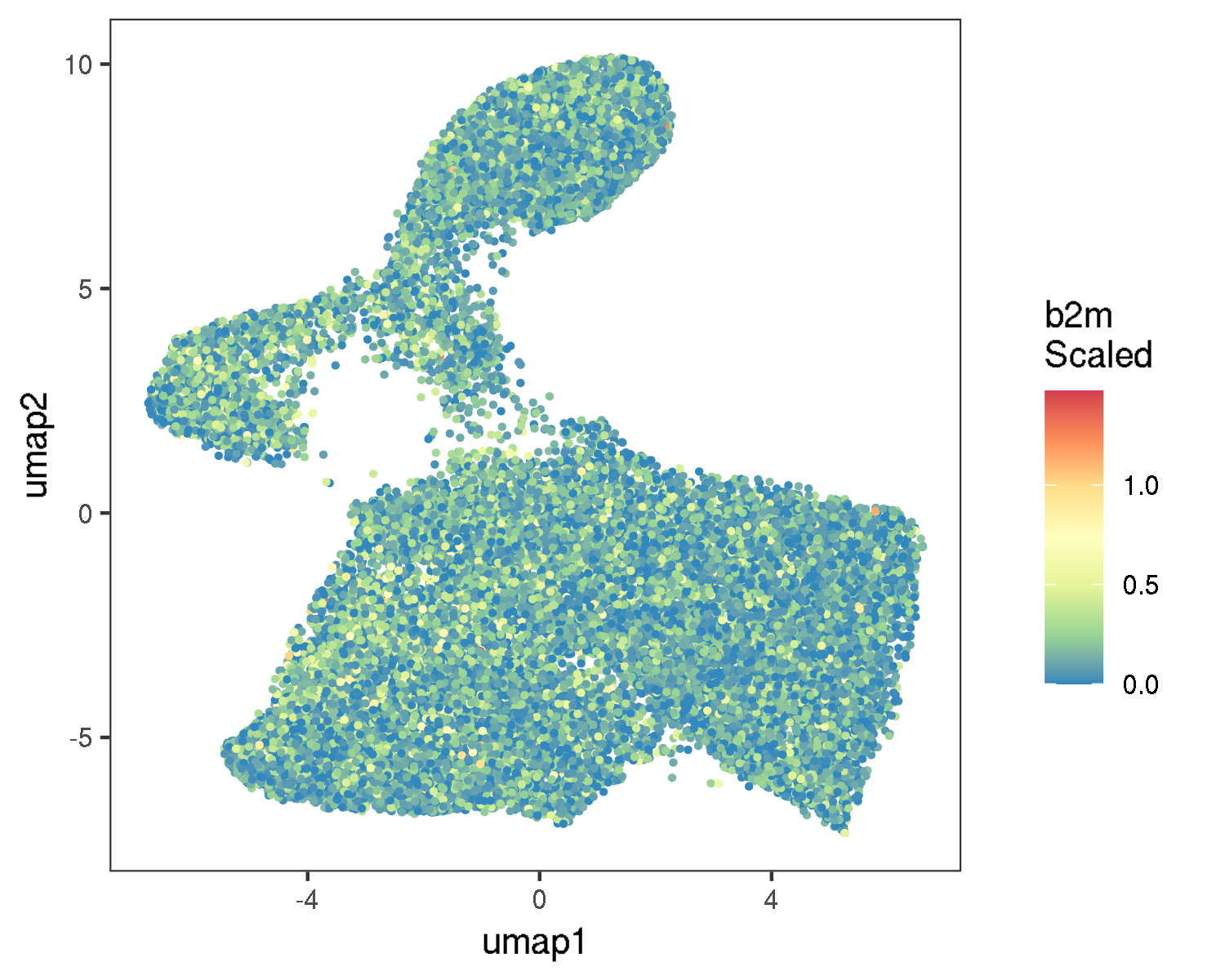

Supplement: Supplementary file 7 — Supplementary Data 4 [file 41467_2024_49883_MOESM7_ESM.zip › png/b2_microglobulin.png]

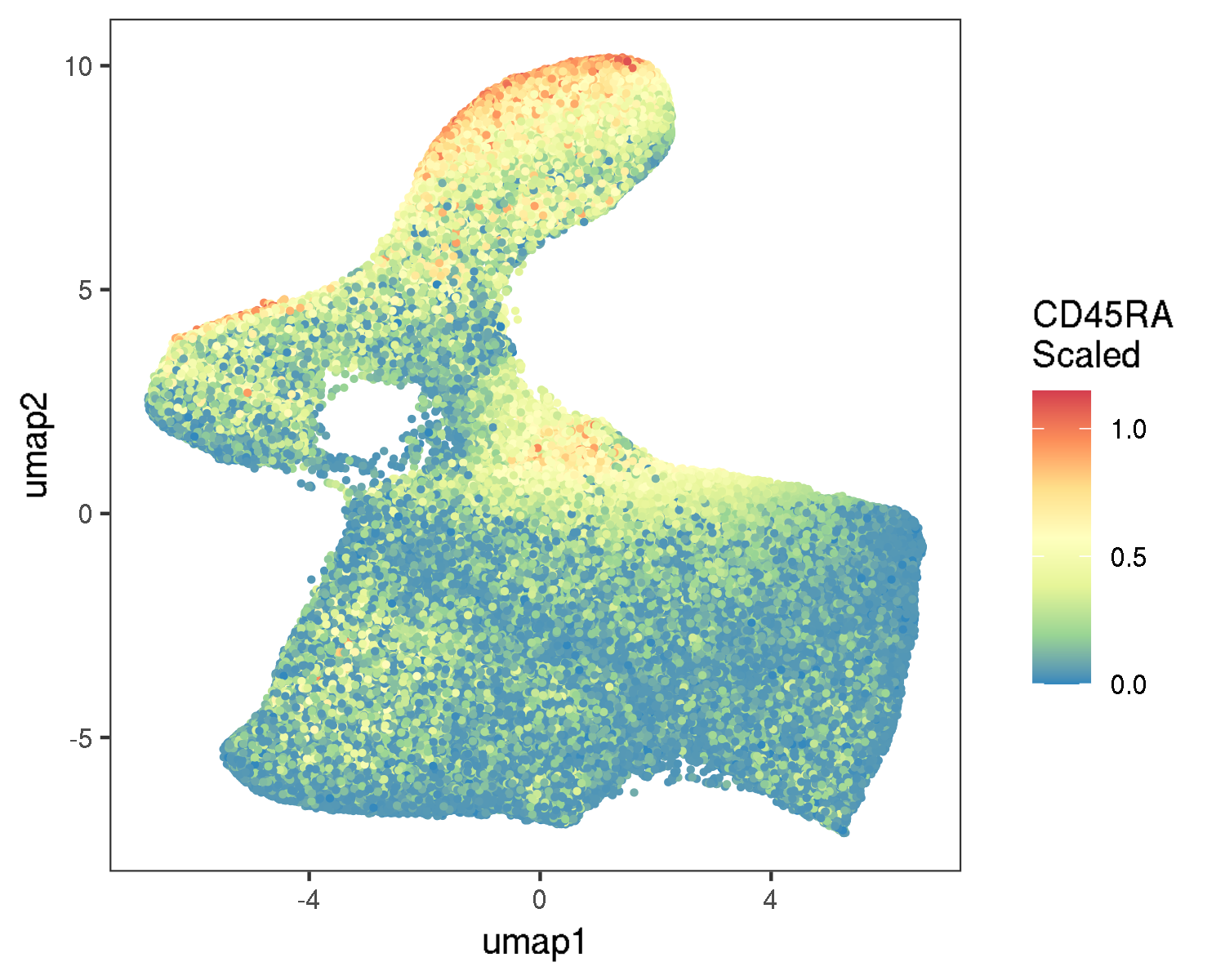

Supplement: Supplementary file 7 — Supplementary Data 4 [file 41467_2024_49883_MOESM7_ESM.zip › png/CD45RA.png]

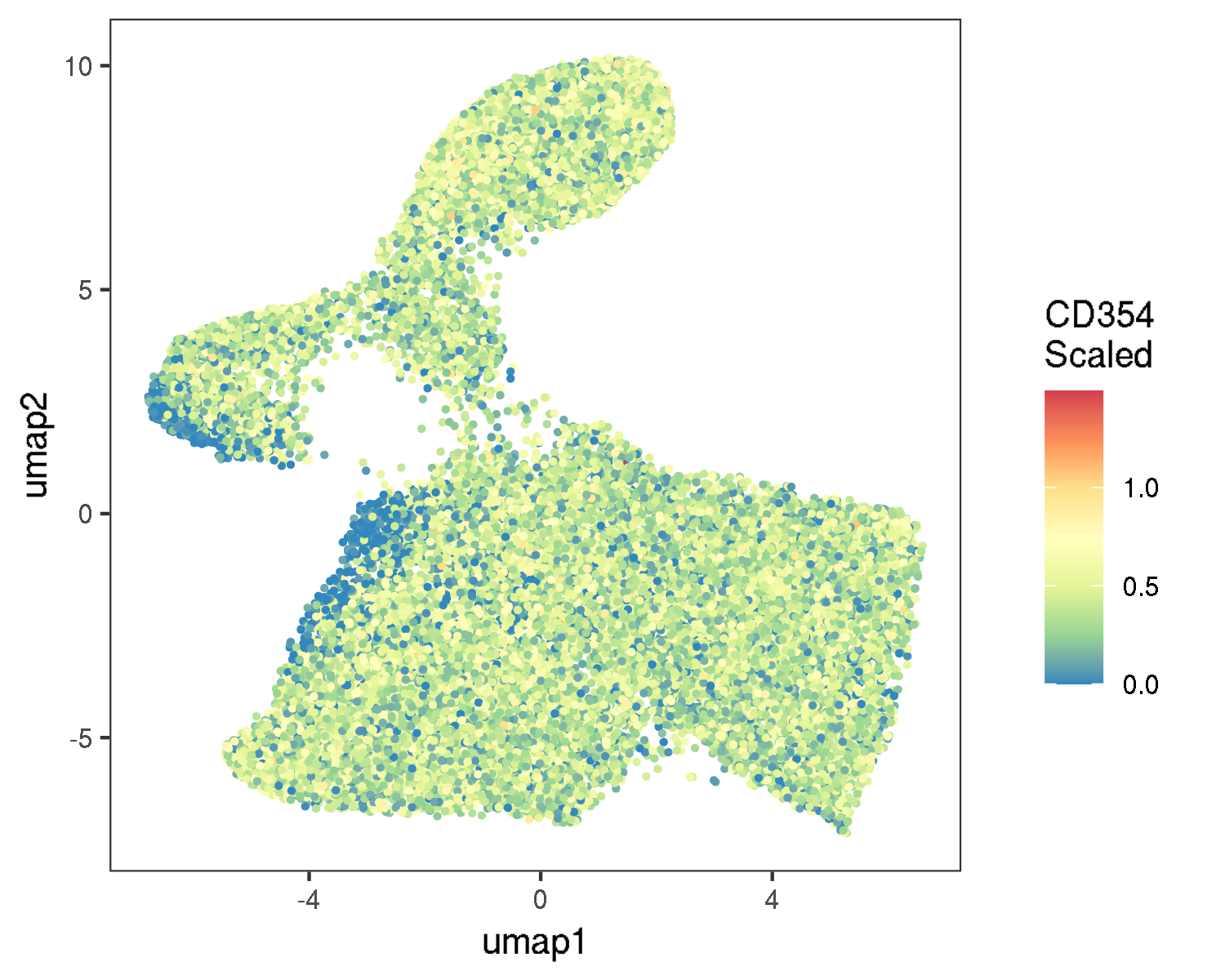

Supplement: Supplementary file 7 — Supplementary Data 4 [file 41467_2024_49883_MOESM7_ESM.zip › png/CD354.png]

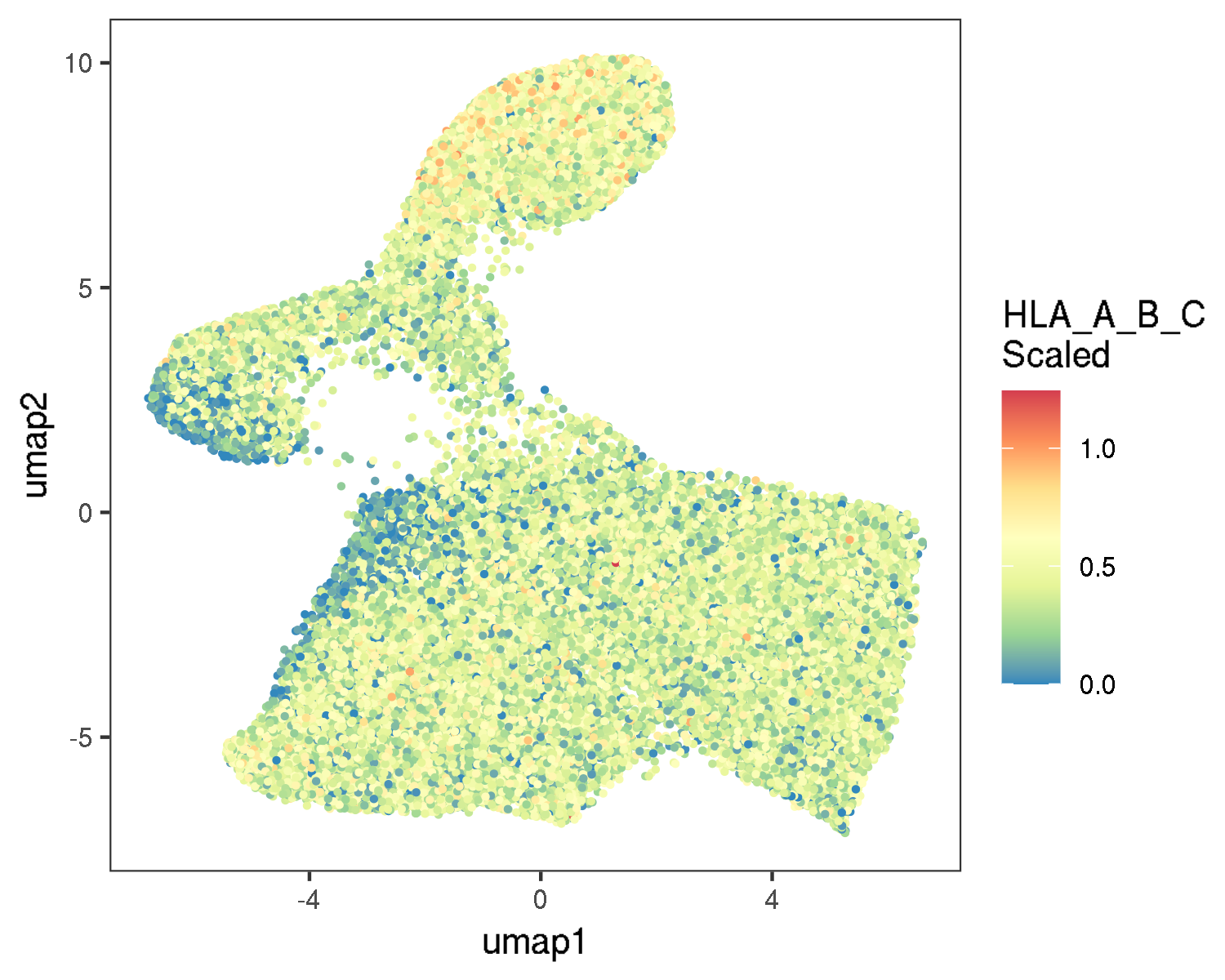

Supplement: Supplementary file 7 — Supplementary Data 4 [file 41467_2024_49883_MOESM7_ESM.zip › png/HLA_A_B_C.png]

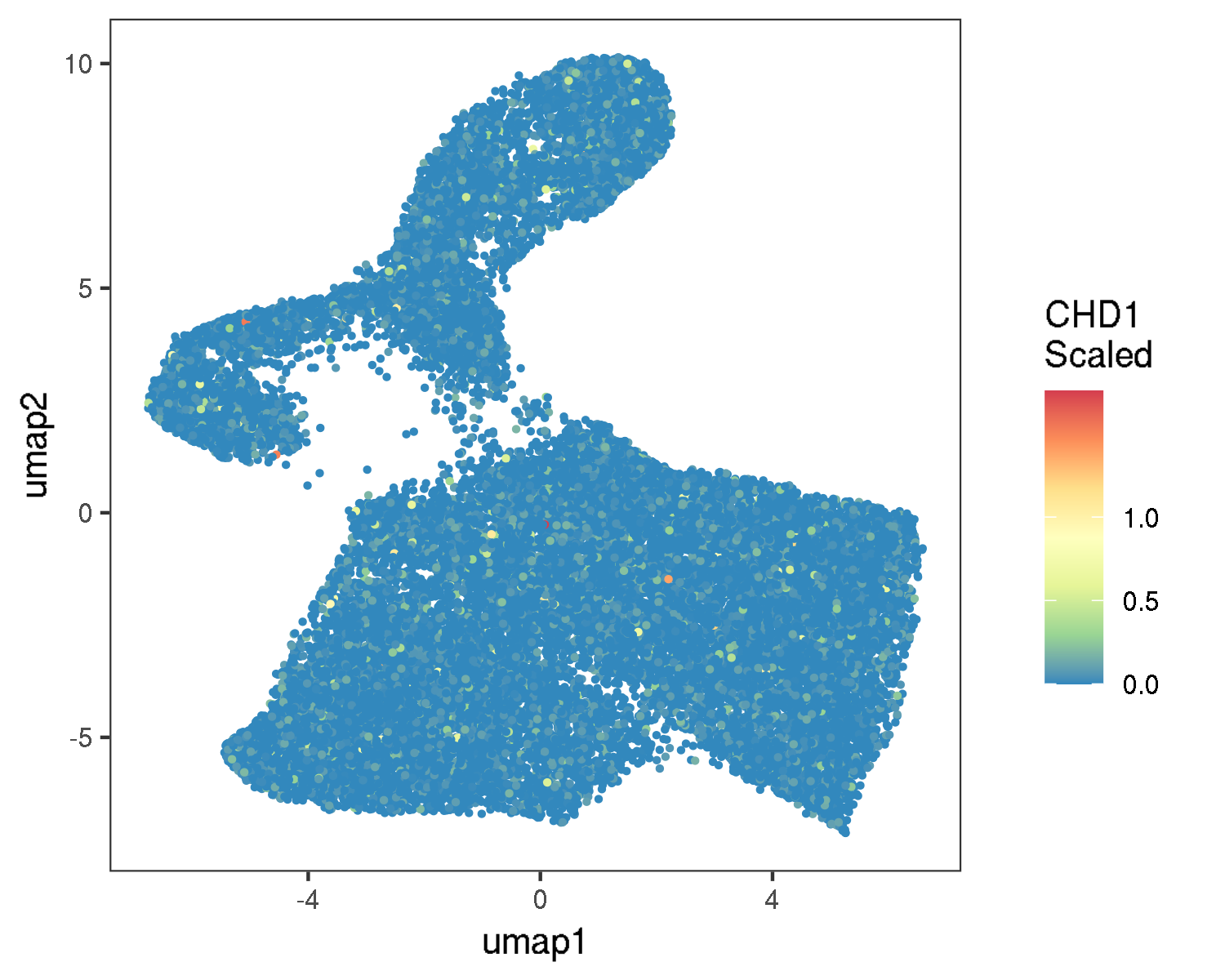

Supplement: Supplementary file 7 — Supplementary Data 4 [file 41467_2024_49883_MOESM7_ESM.zip › png/CHD1.png]

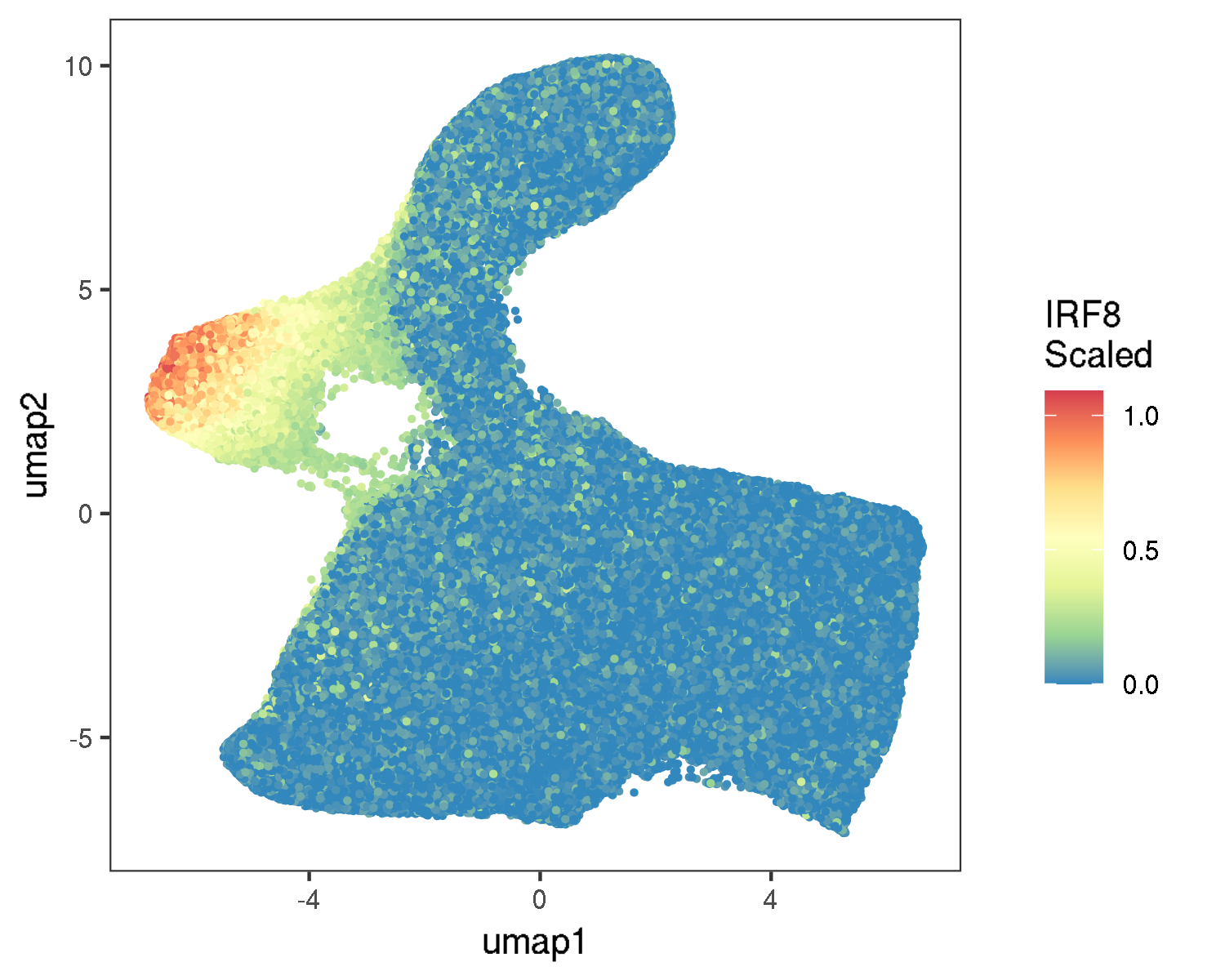

Supplement: Supplementary file 7 — Supplementary Data 4 [file 41467_2024_49883_MOESM7_ESM.zip › png/IRF8.png]

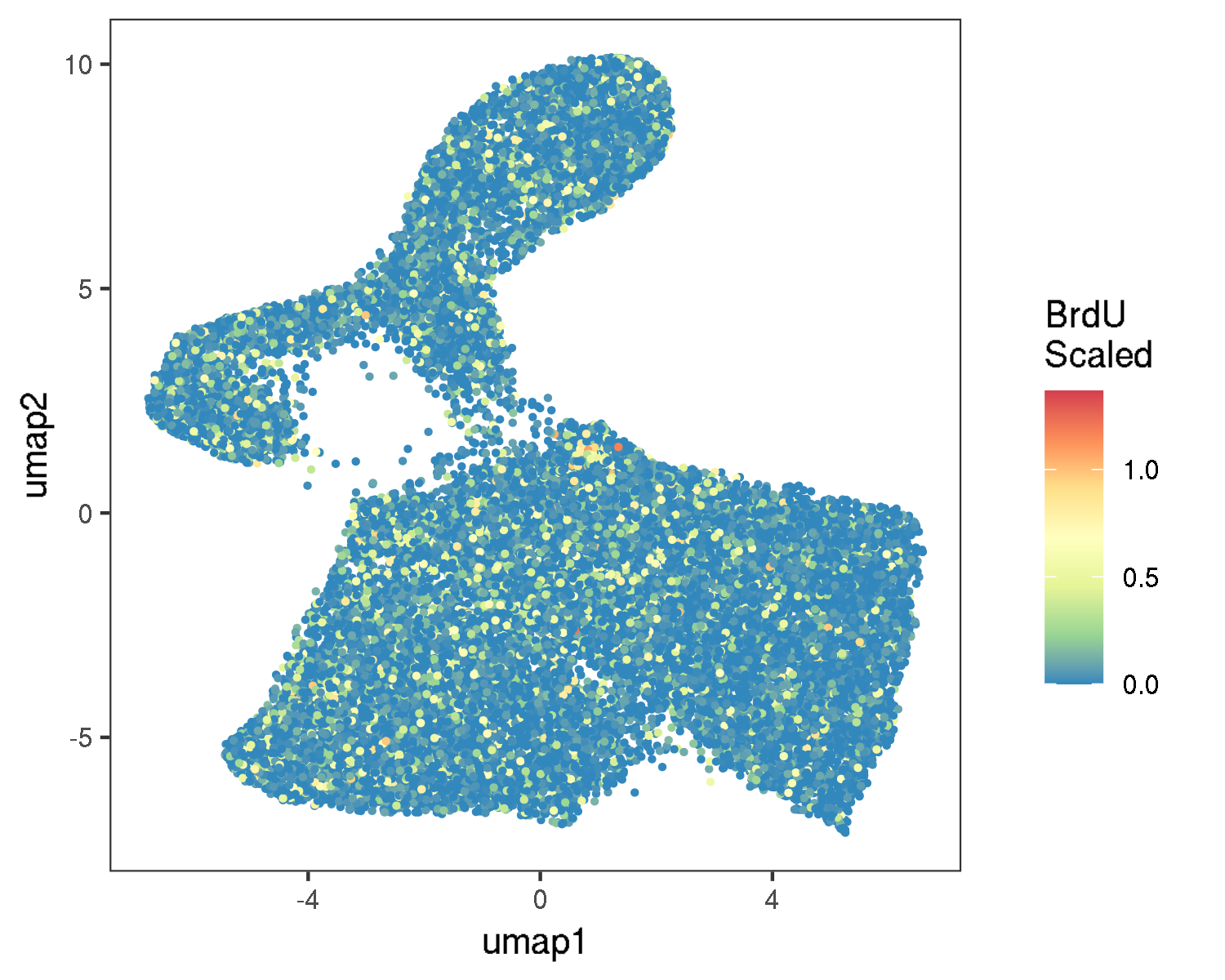

Supplement: Supplementary file 7 — Supplementary Data 4 [file 41467_2024_49883_MOESM7_ESM.zip › png/BrdU.png]

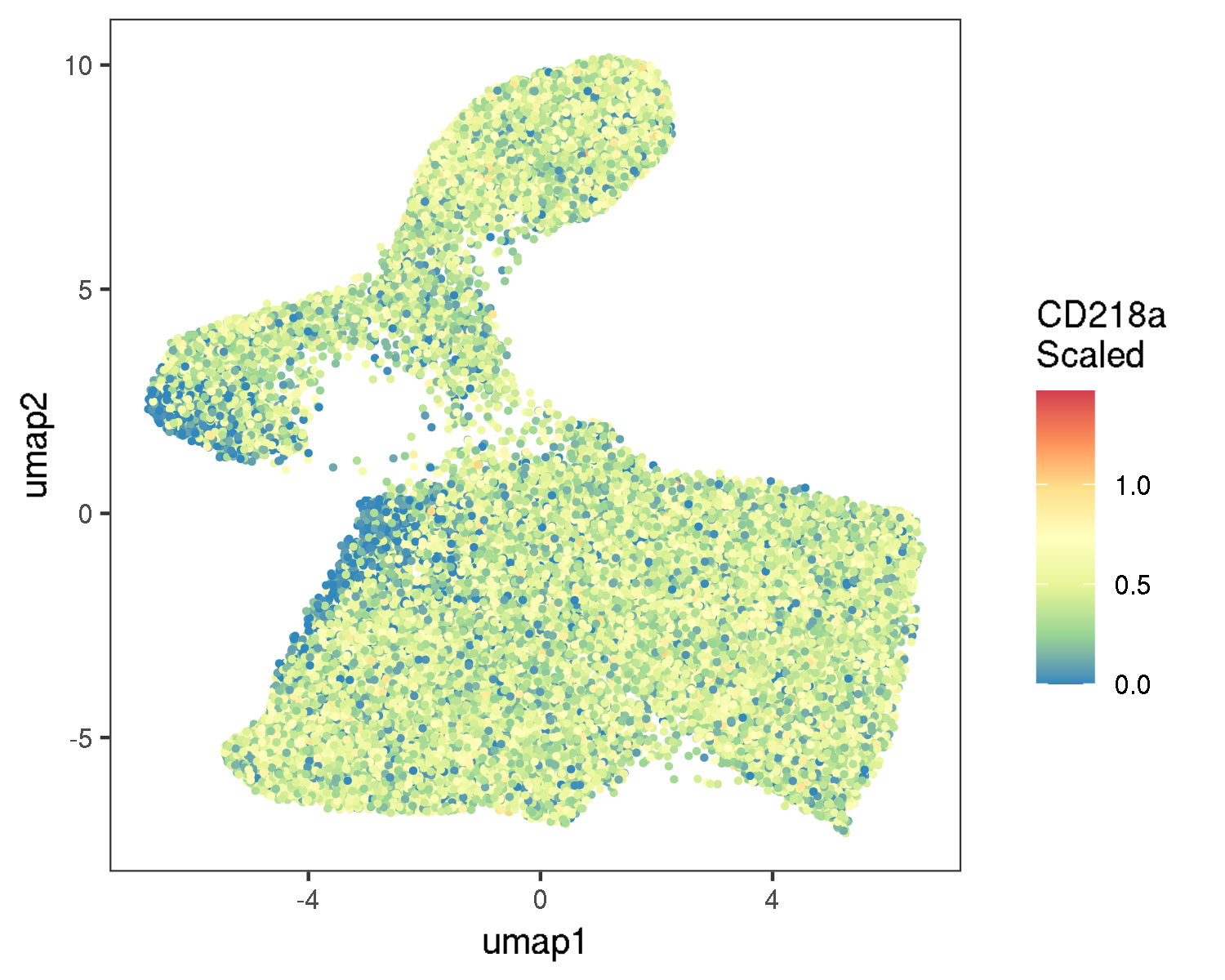

Supplement: Supplementary file 7 — Supplementary Data 4 [file 41467_2024_49883_MOESM7_ESM.zip › png/CD218a.png]

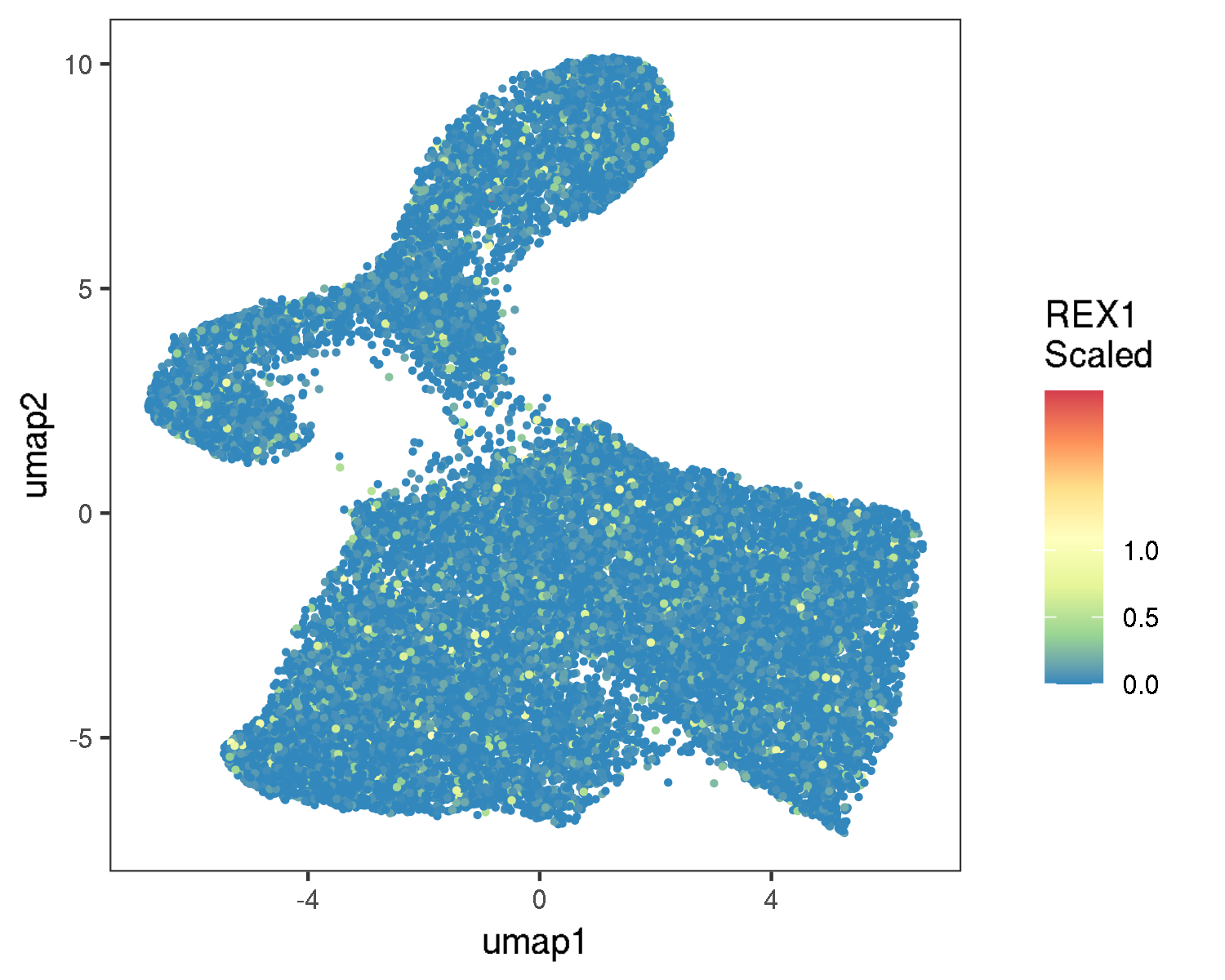

Supplement: Supplementary file 7 — Supplementary Data 4 [file 41467_2024_49883_MOESM7_ESM.zip › png/REX1.png]

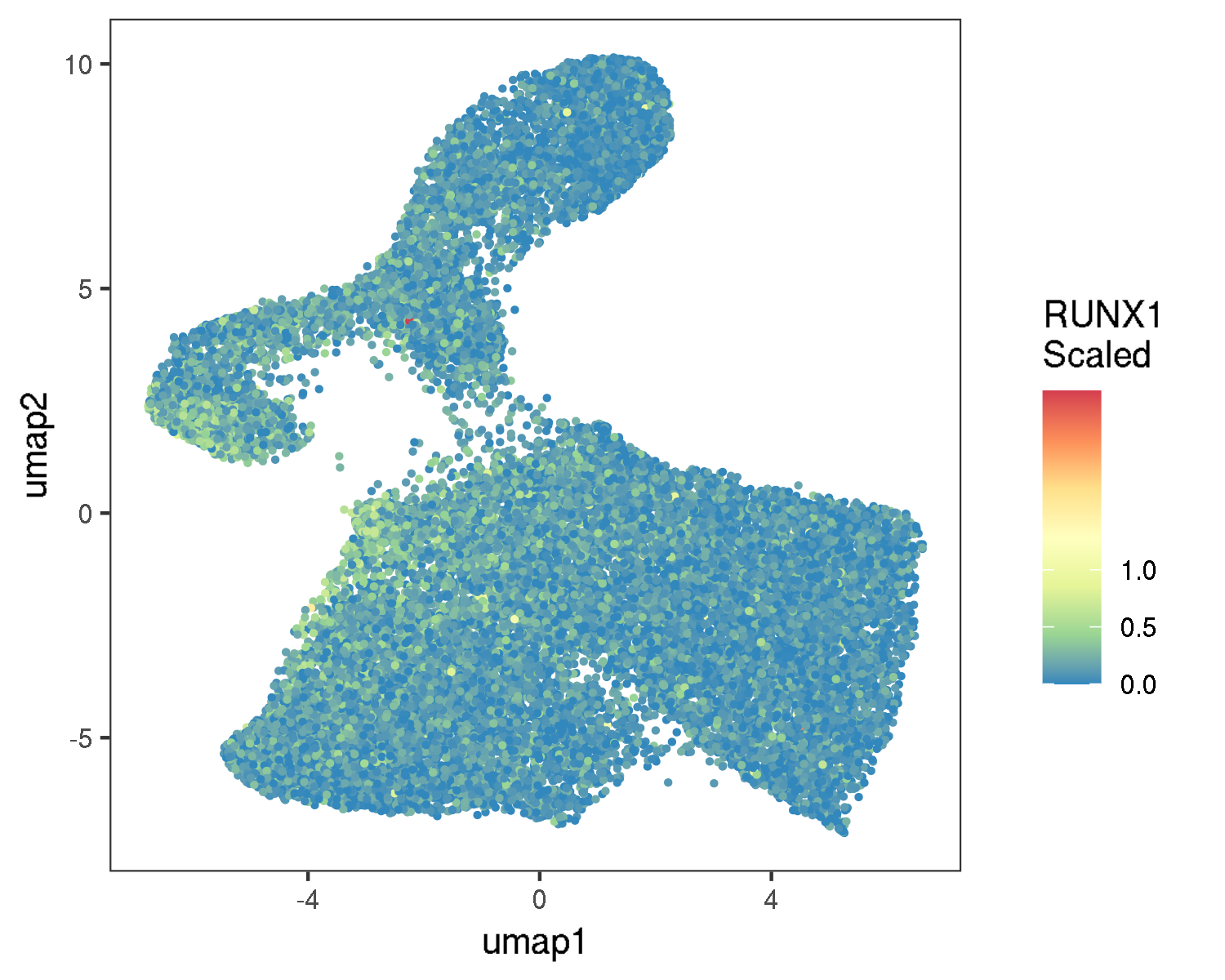

Supplement: Supplementary file 7 — Supplementary Data 4 [file 41467_2024_49883_MOESM7_ESM.zip › png/RUNX1.png]

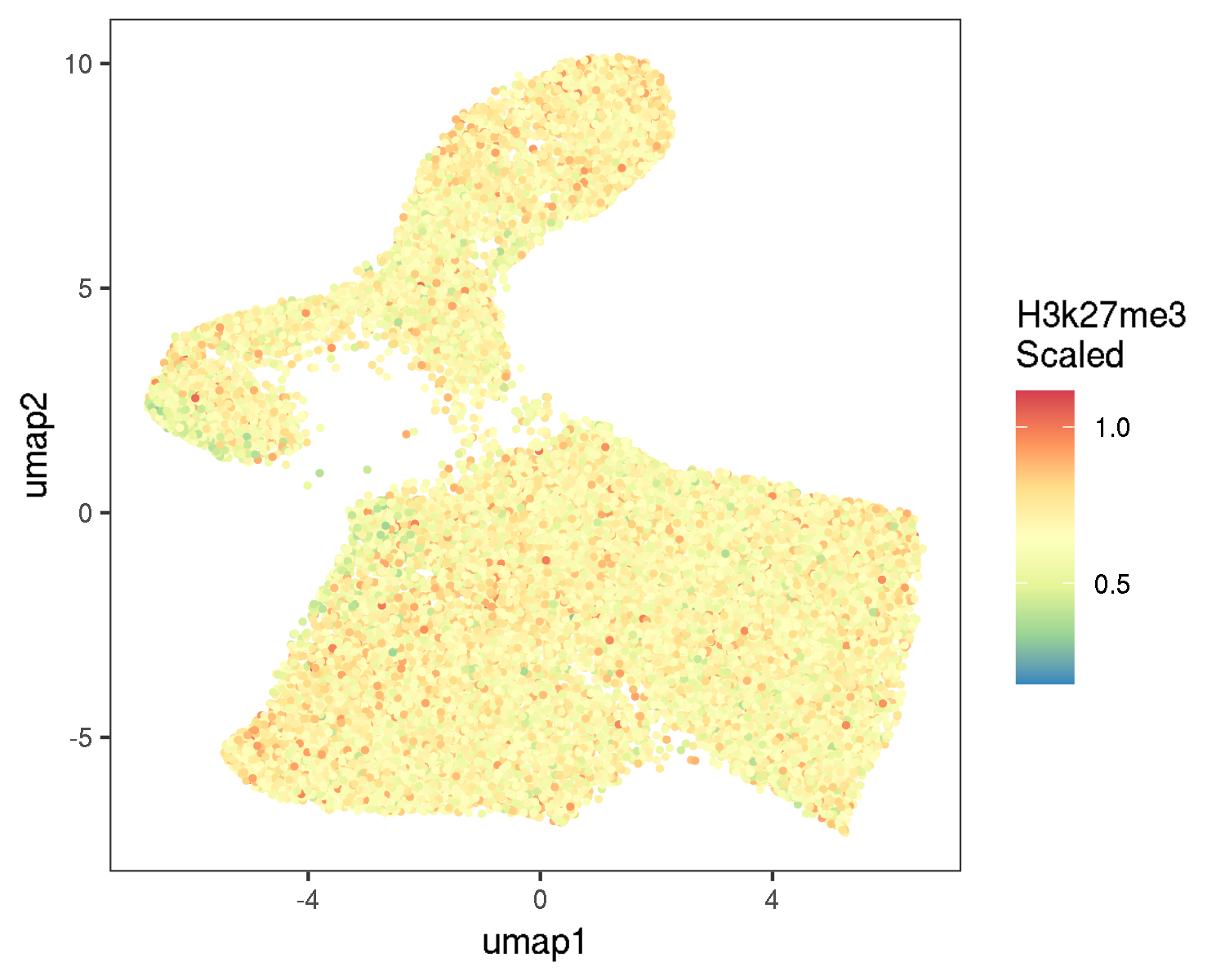

Supplement: Supplementary file 7 — Supplementary Data 4 [file 41467_2024_49883_MOESM7_ESM.zip › png/H3k27me3.png]

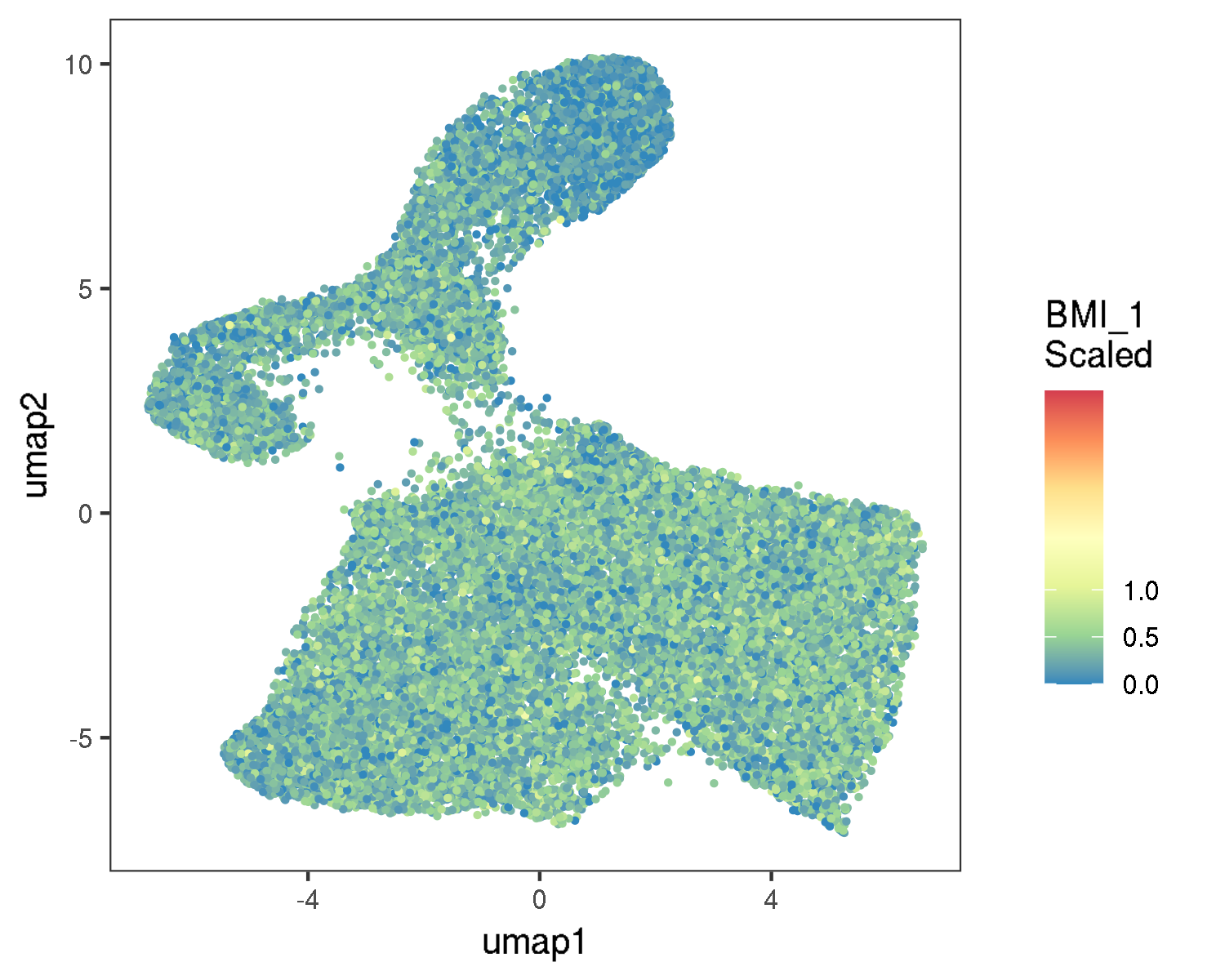

Supplement: Supplementary file 7 — Supplementary Data 4 [file 41467_2024_49883_MOESM7_ESM.zip › png/BMI_1.png]

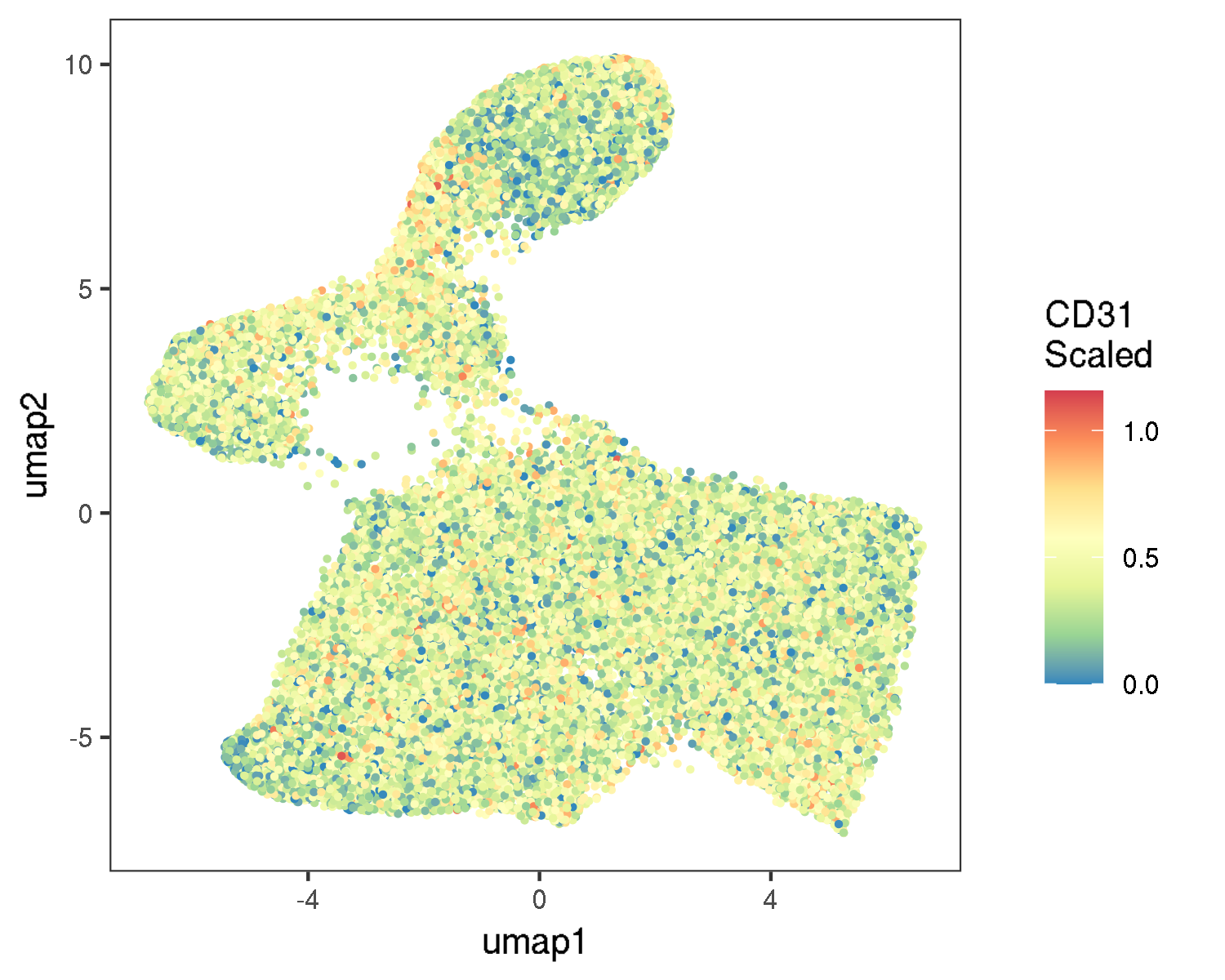

Supplement: Supplementary file 7 — Supplementary Data 4 [file 41467_2024_49883_MOESM7_ESM.zip › png/CD31.png]

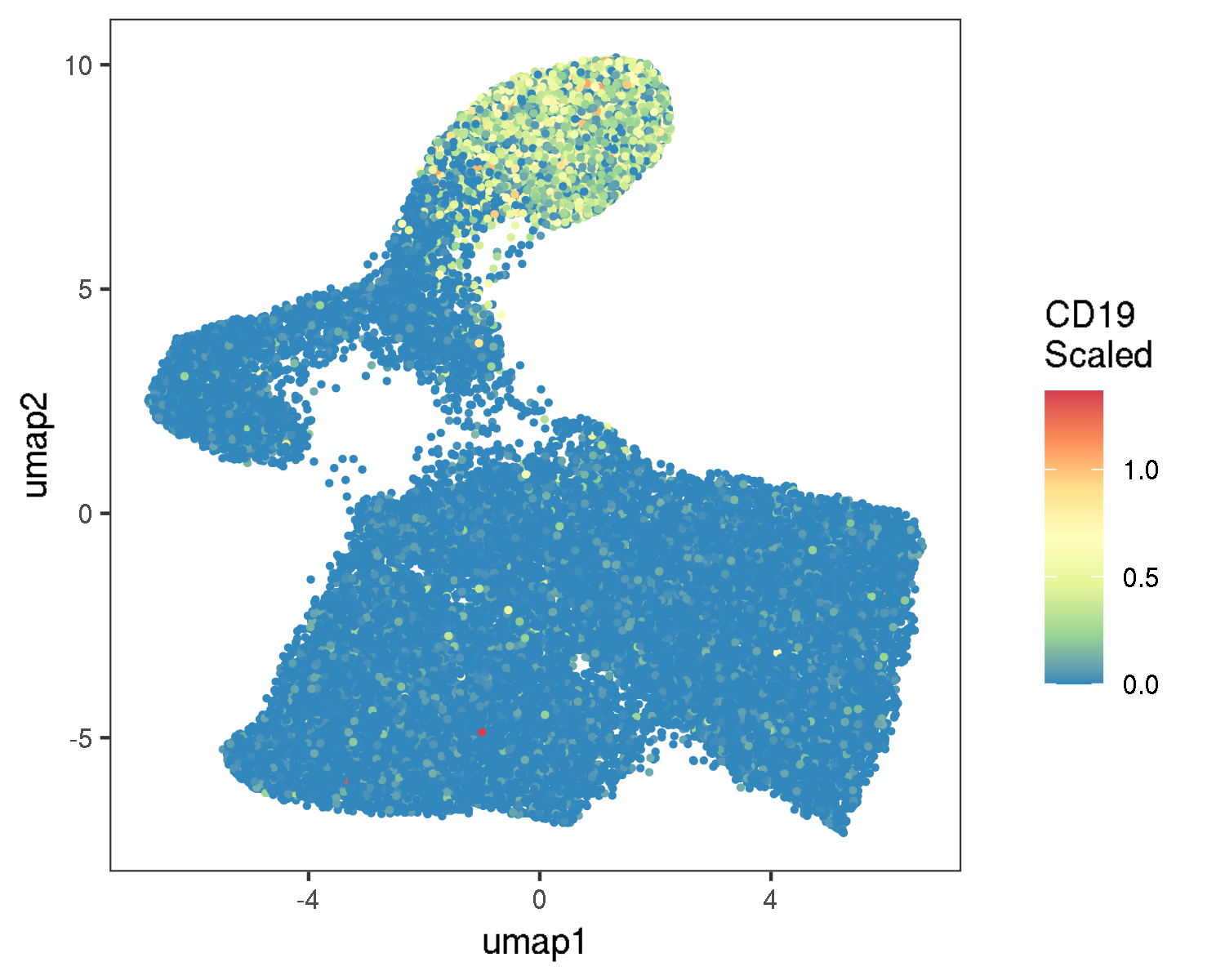

Supplement: Supplementary file 7 — Supplementary Data 4 [file 41467_2024_49883_MOESM7_ESM.zip › png/CD19.png]

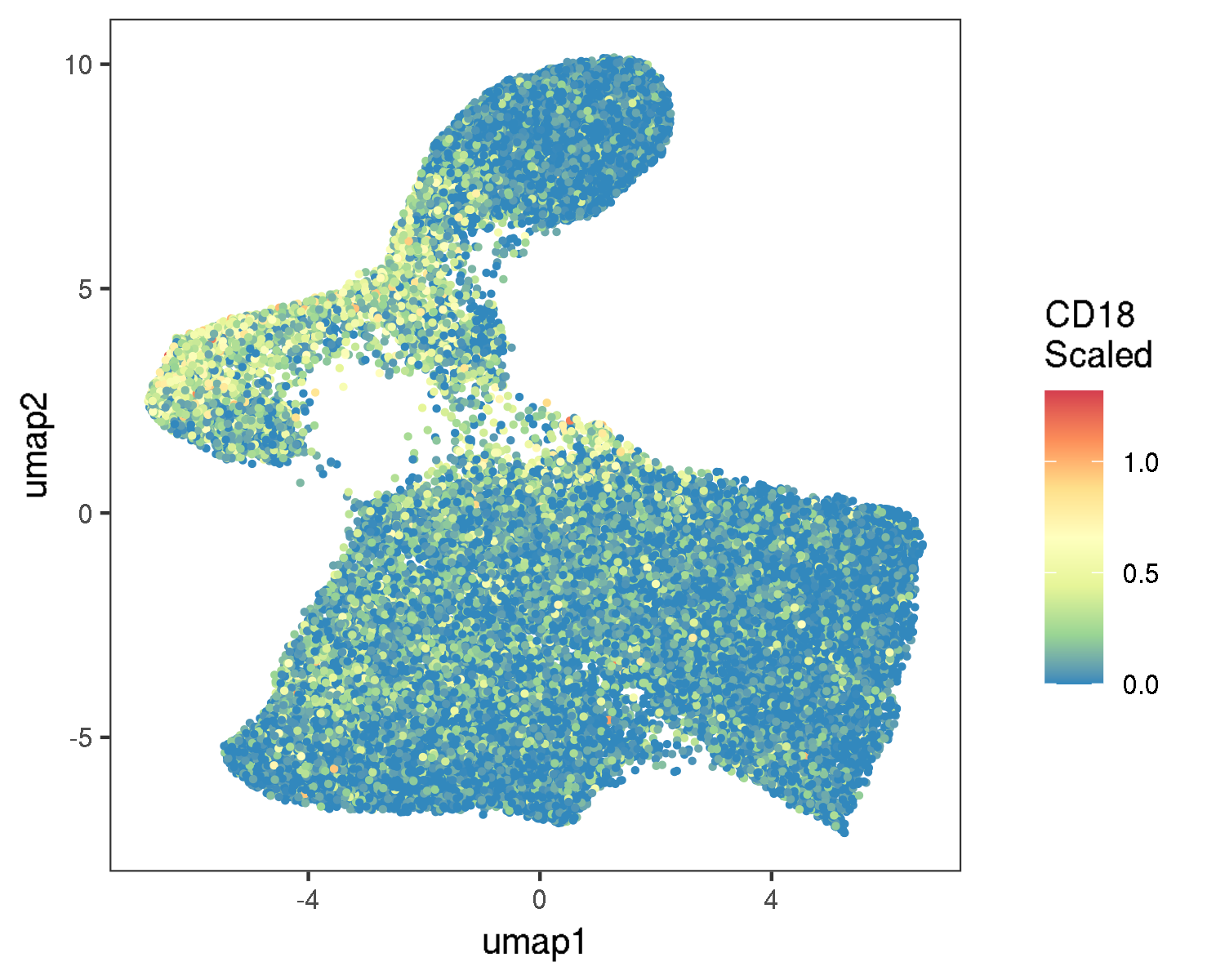

Supplement: Supplementary file 7 — Supplementary Data 4 [file 41467_2024_49883_MOESM7_ESM.zip › png/CD18.png]

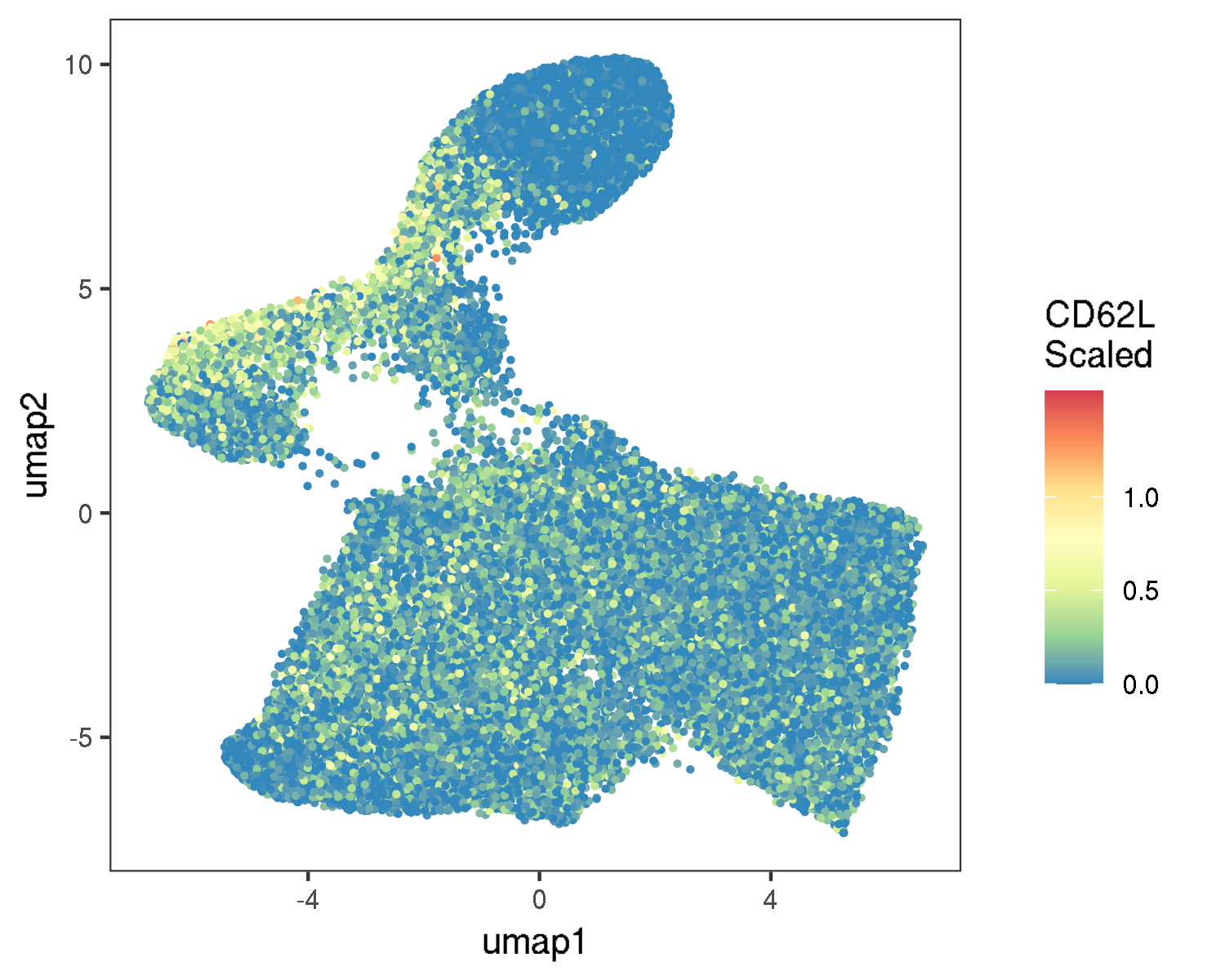

Supplement: Supplementary file 7 — Supplementary Data 4 [file 41467_2024_49883_MOESM7_ESM.zip › png/CD62L.png]

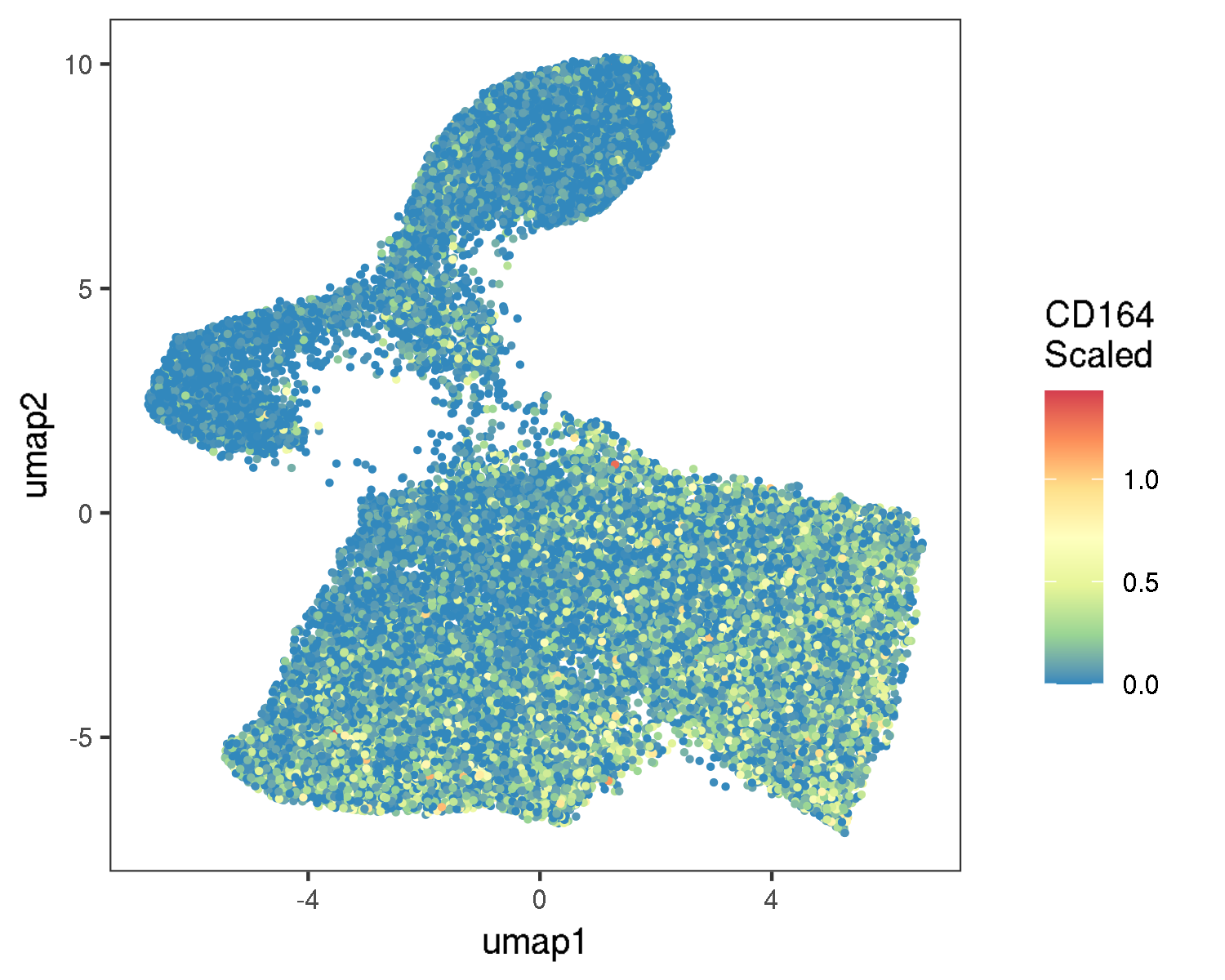

Supplement: Supplementary file 7 — Supplementary Data 4 [file 41467_2024_49883_MOESM7_ESM.zip › png/CD164.png]

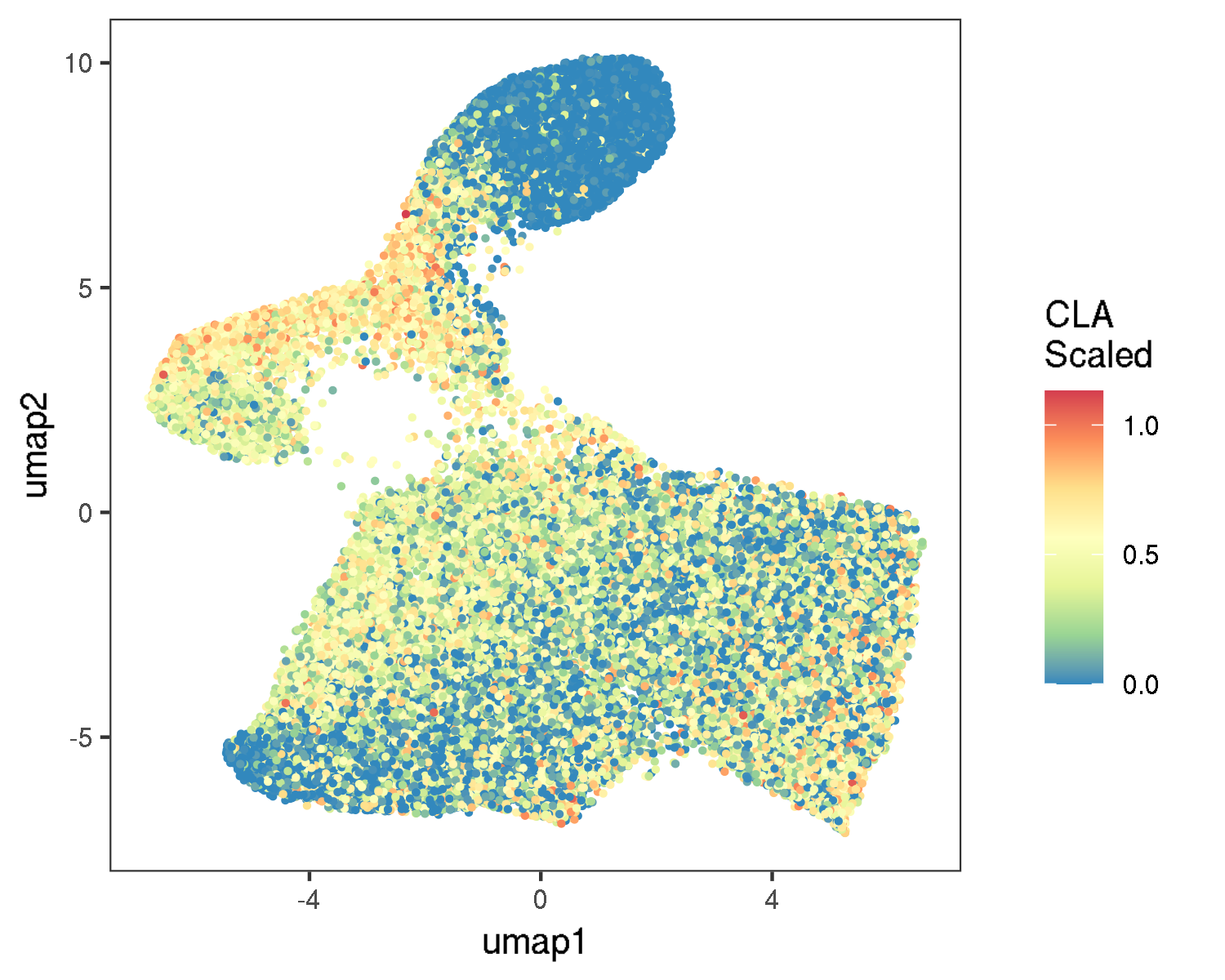

Supplement: Supplementary file 7 — Supplementary Data 4 [file 41467_2024_49883_MOESM7_ESM.zip › png/CLA.png]

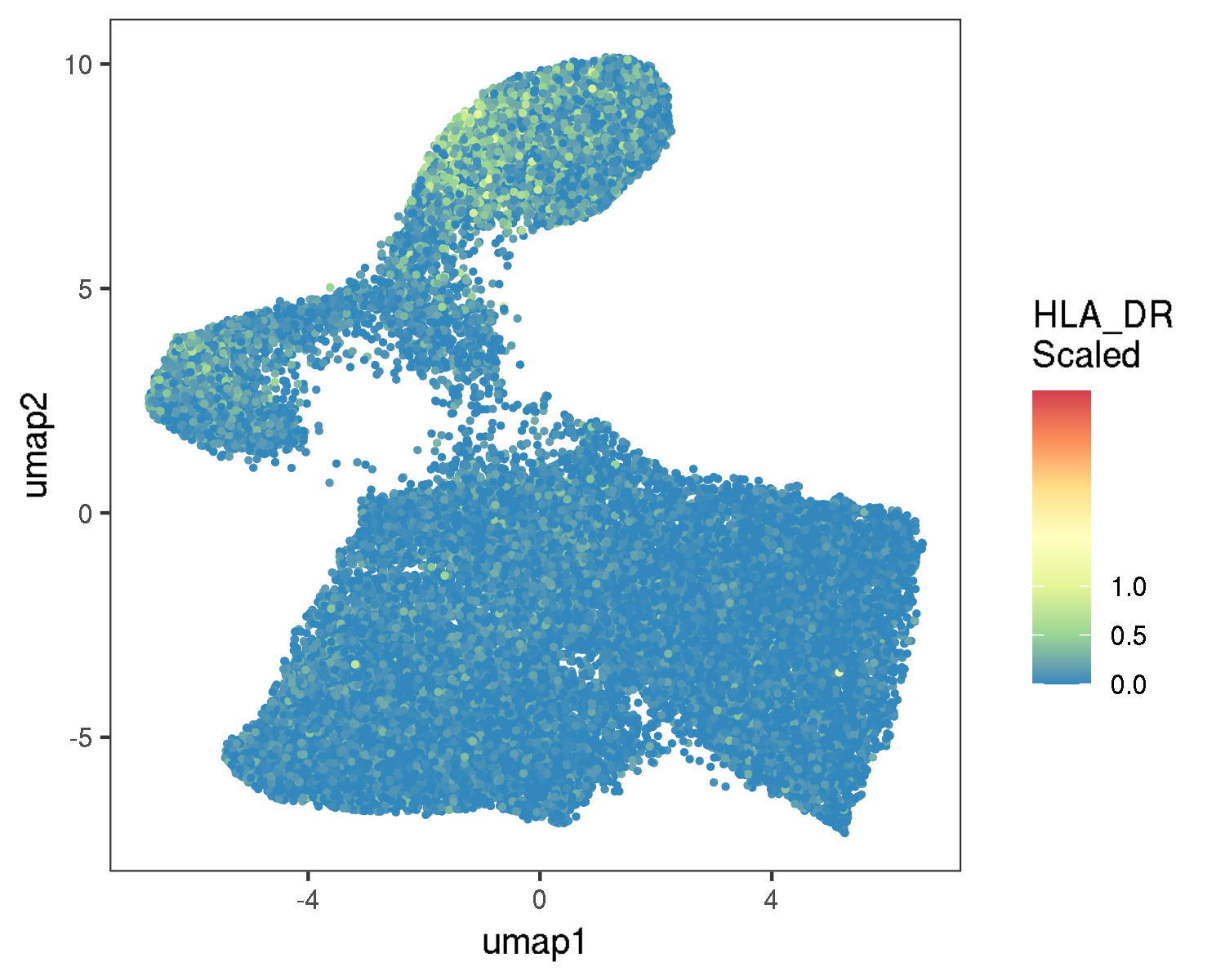

Supplement: Supplementary file 7 — Supplementary Data 4 [file 41467_2024_49883_MOESM7_ESM.zip › png/HLA_DR.png]

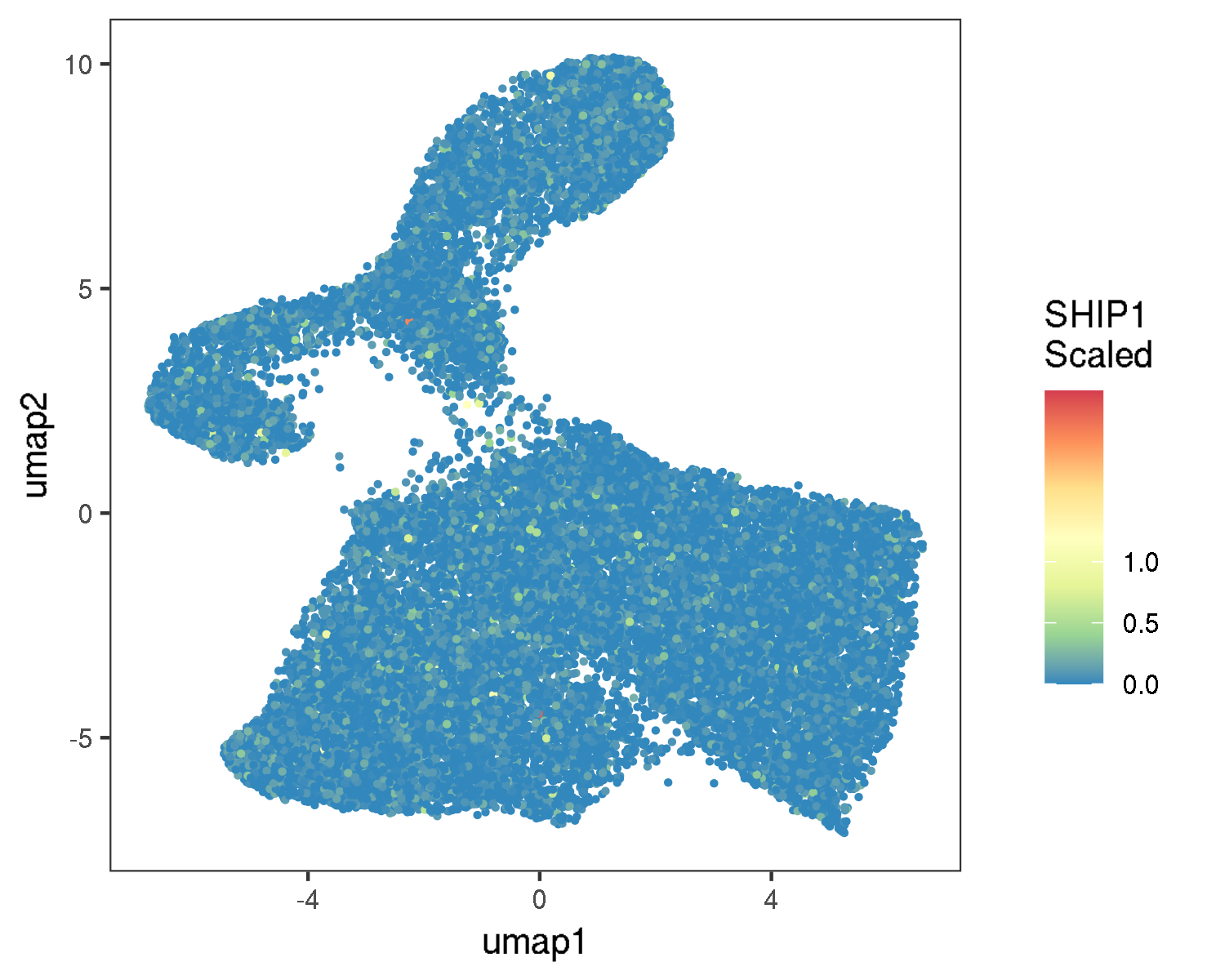

Supplement: Supplementary file 7 — Supplementary Data 4 [file 41467_2024_49883_MOESM7_ESM.zip › png/SHIP1.png]

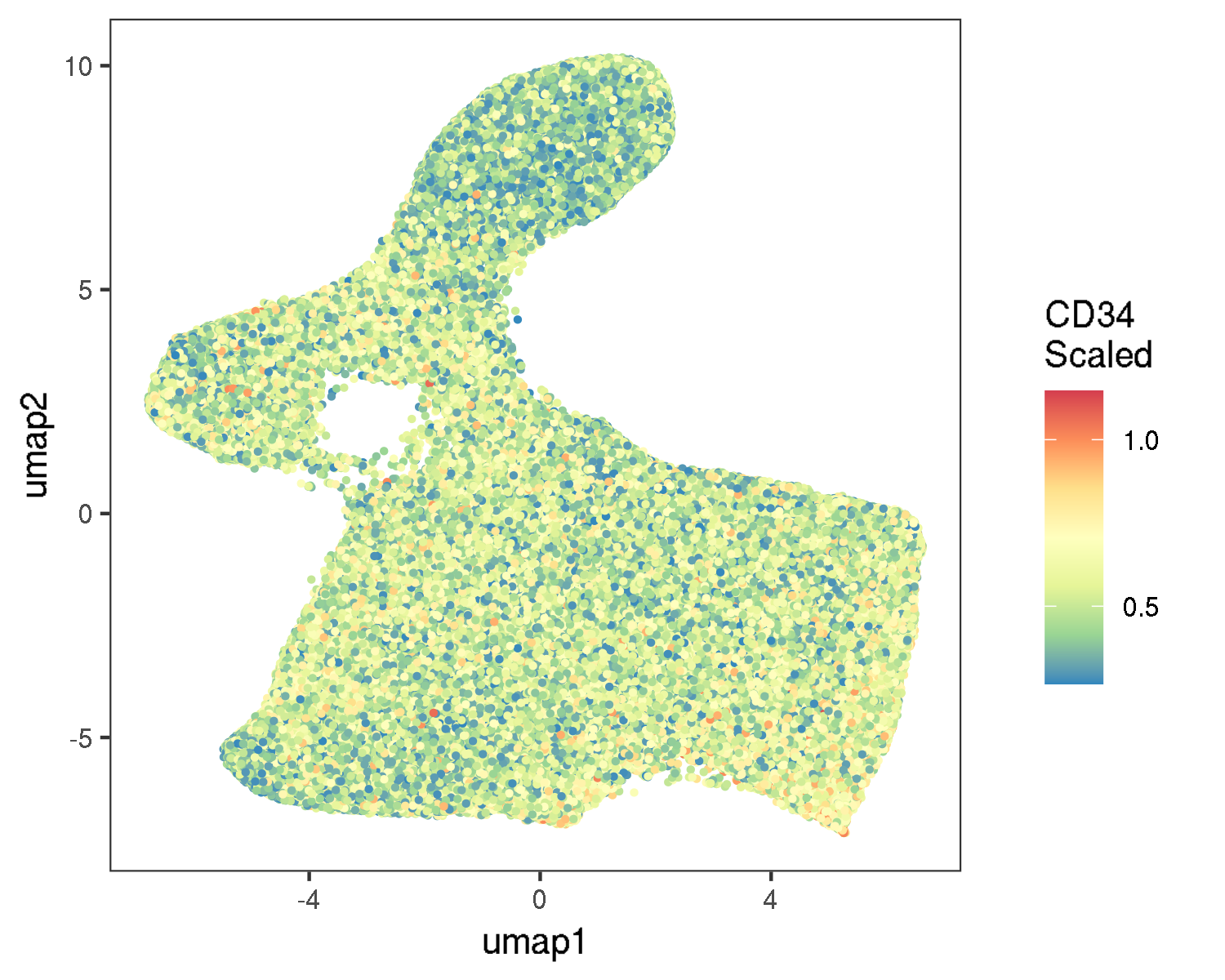

Supplement: Supplementary file 7 — Supplementary Data 4 [file 41467_2024_49883_MOESM7_ESM.zip › png/CD34.png]

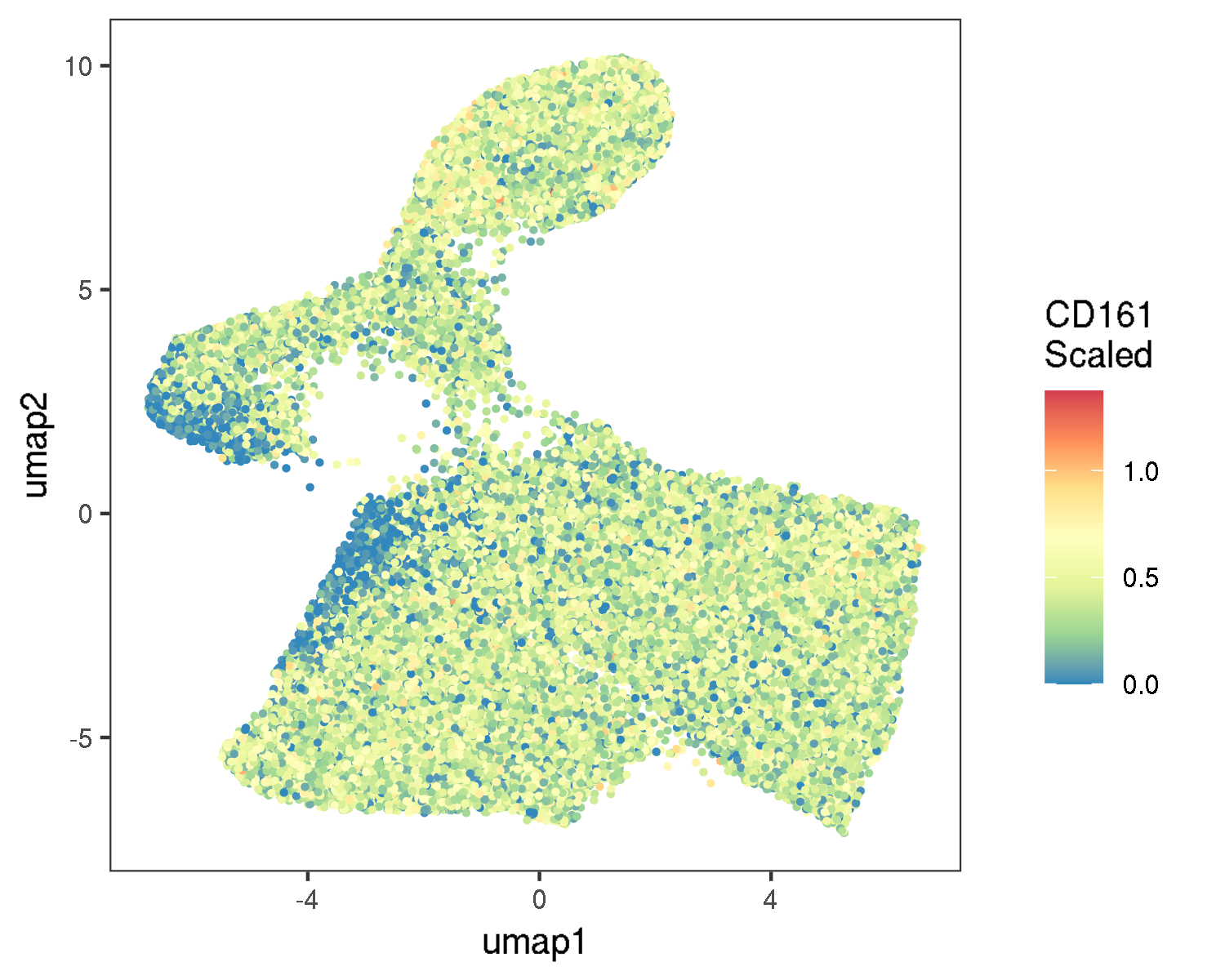

Supplement: Supplementary file 7 — Supplementary Data 4 [file 41467_2024_49883_MOESM7_ESM.zip › png/CD161.png]

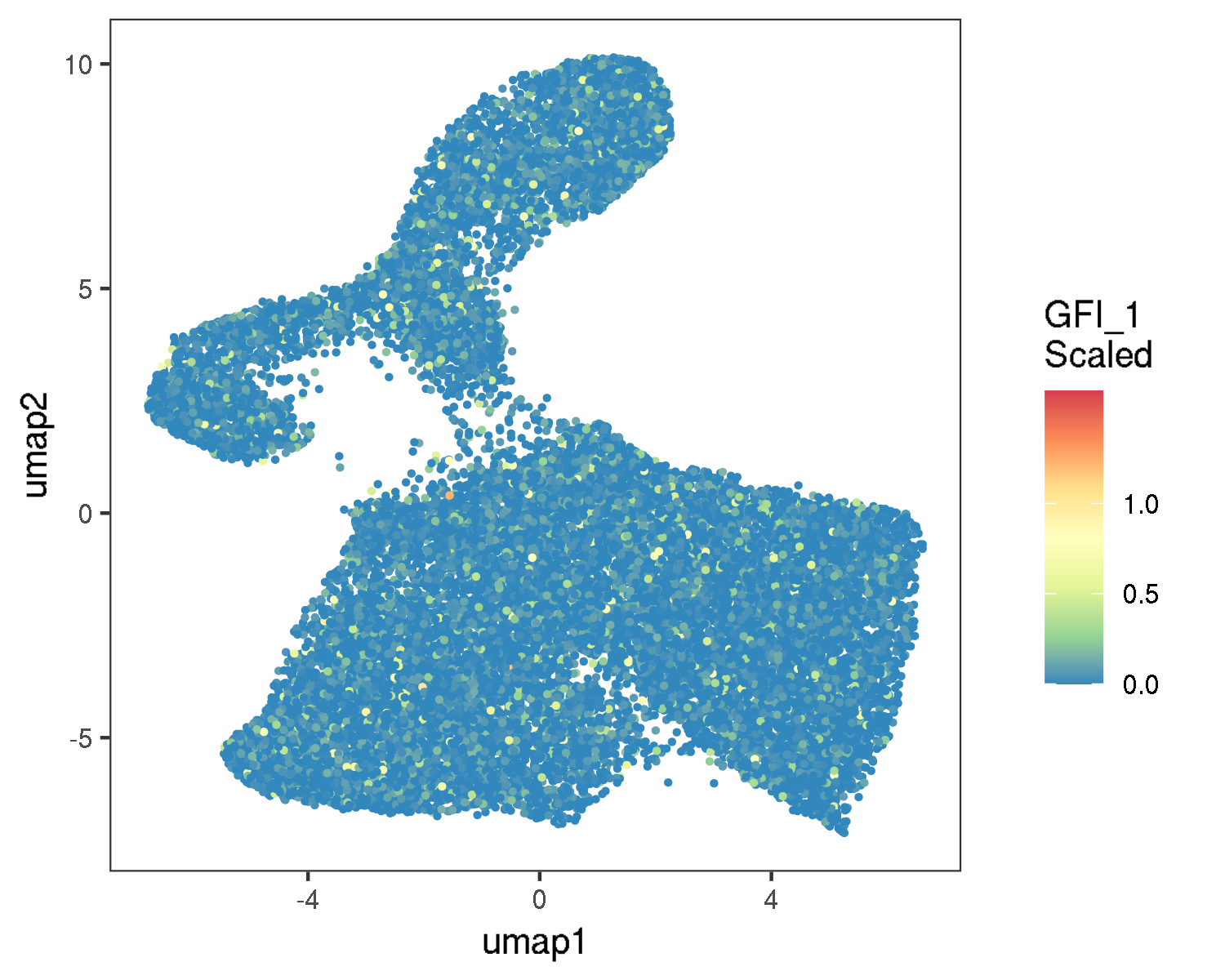

Supplement: Supplementary file 7 — Supplementary Data 4 [file 41467_2024_49883_MOESM7_ESM.zip › png/GFI_1.png]

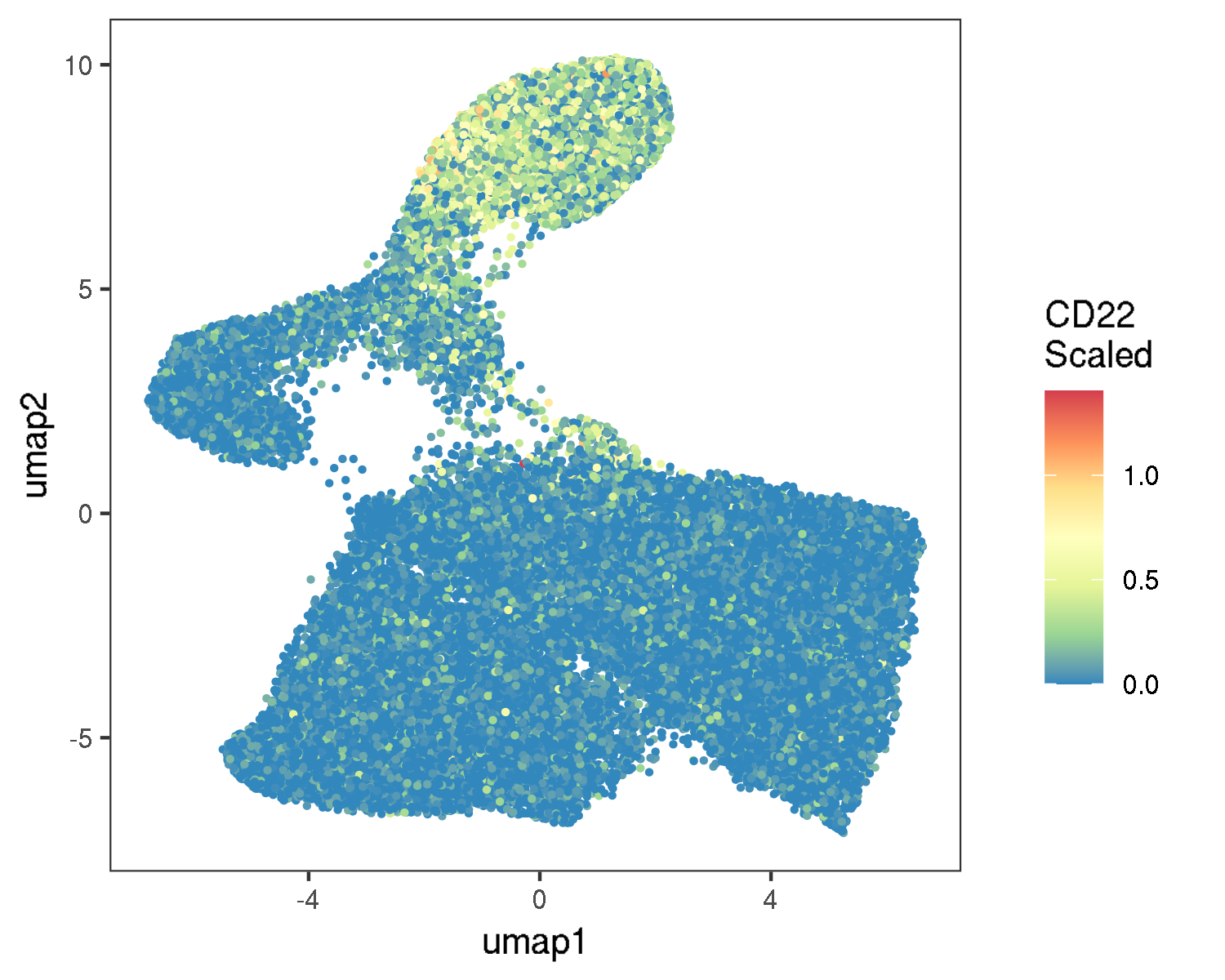

Supplement: Supplementary file 7 — Supplementary Data 4 [file 41467_2024_49883_MOESM7_ESM.zip › png/CD22.png]

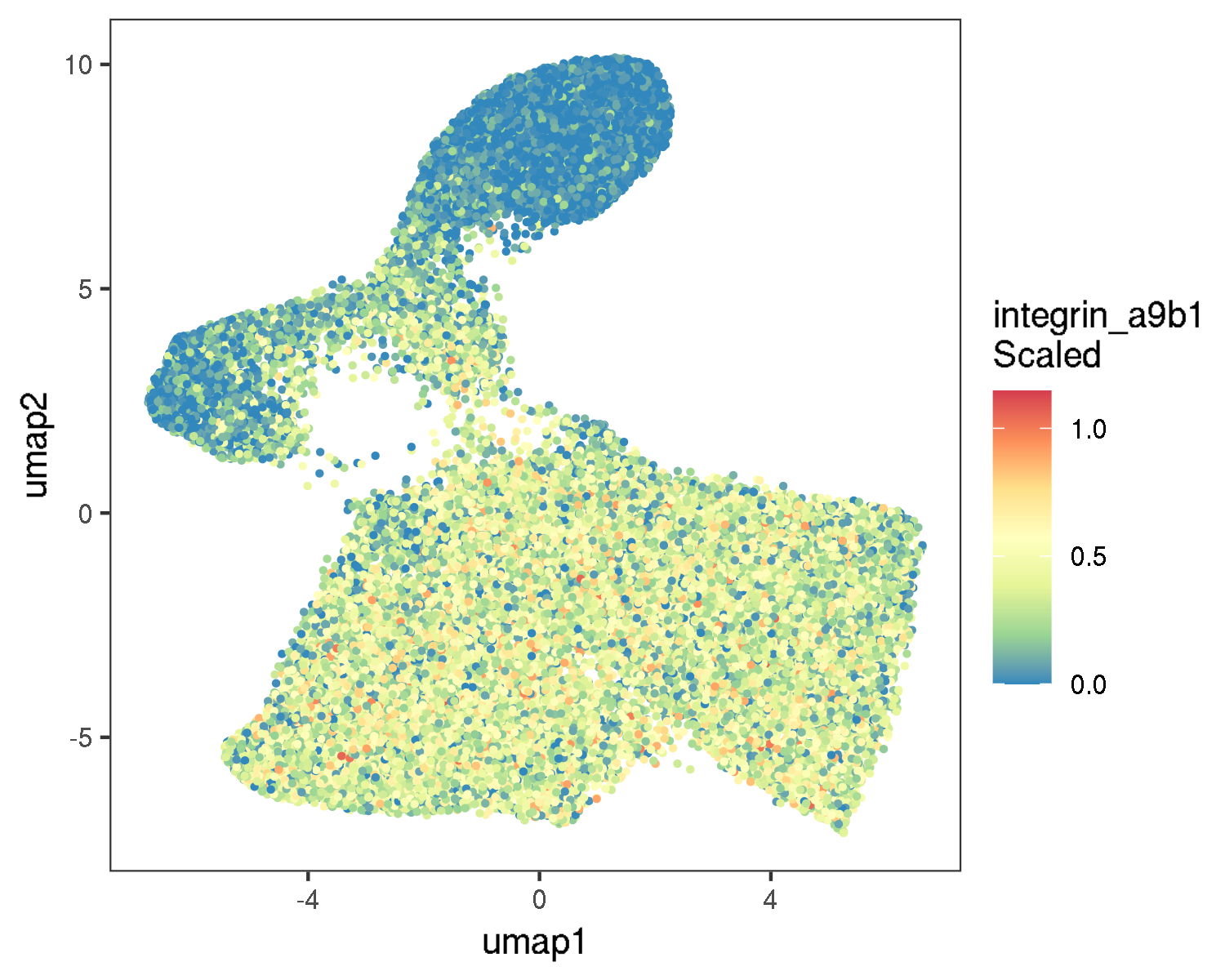

Supplement: Supplementary file 7 — Supplementary Data 4 [file 41467_2024_49883_MOESM7_ESM.zip › png/integrin_a9b1.png]

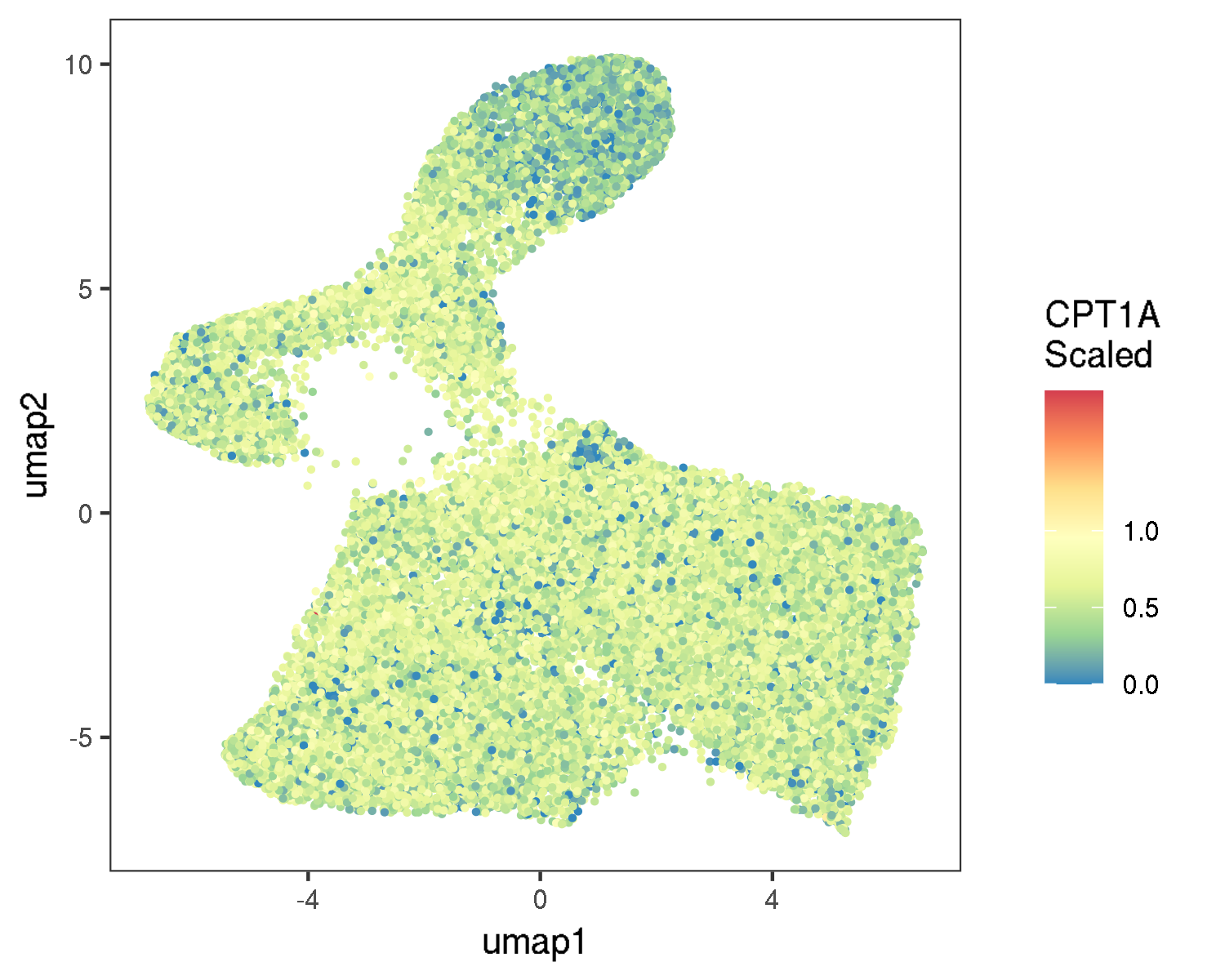

Supplement: Supplementary file 7 — Supplementary Data 4 [file 41467_2024_49883_MOESM7_ESM.zip › png/CPT1A.png]

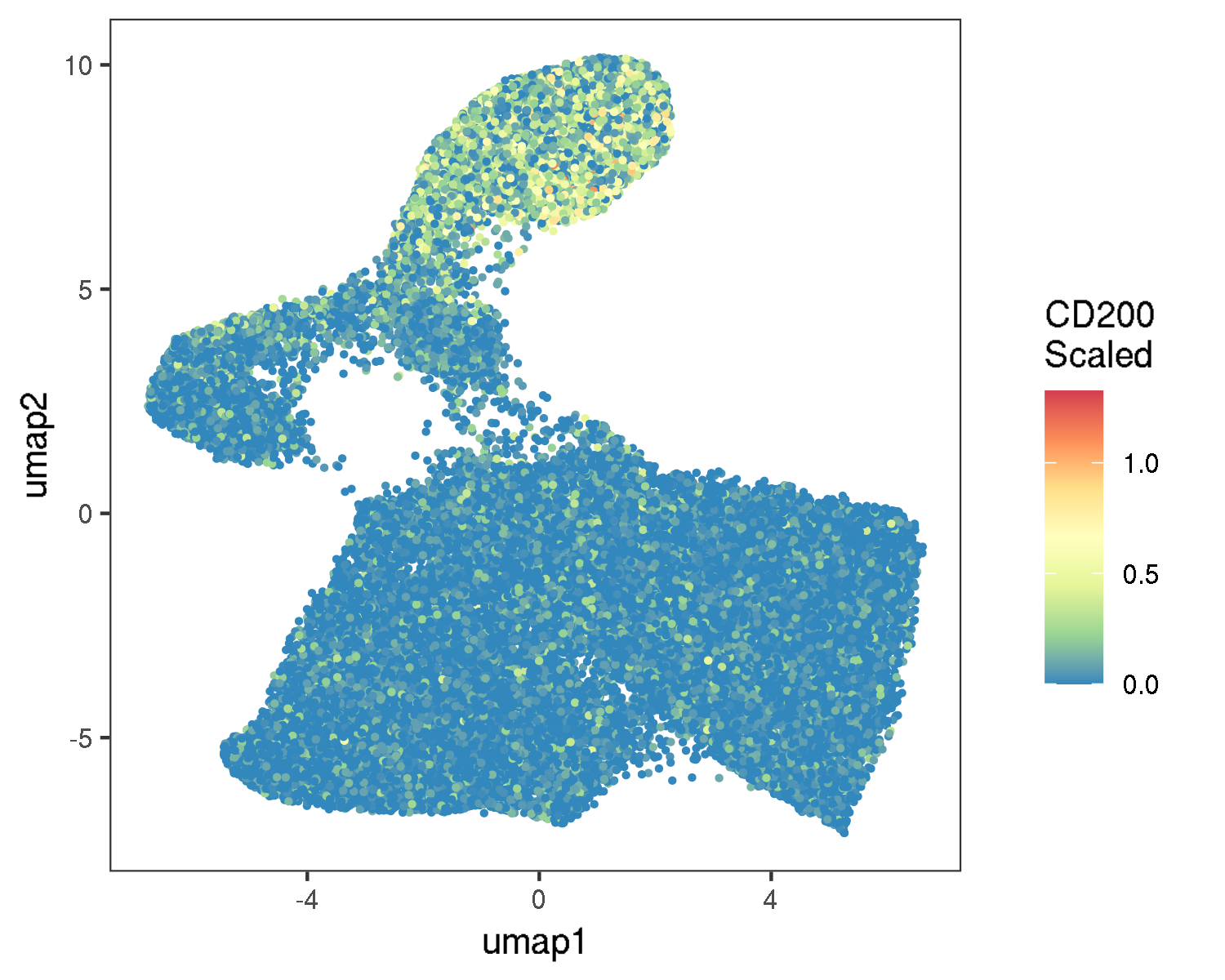

Supplement: Supplementary file 7 — Supplementary Data 4 [file 41467_2024_49883_MOESM7_ESM.zip › png/CD200.png]

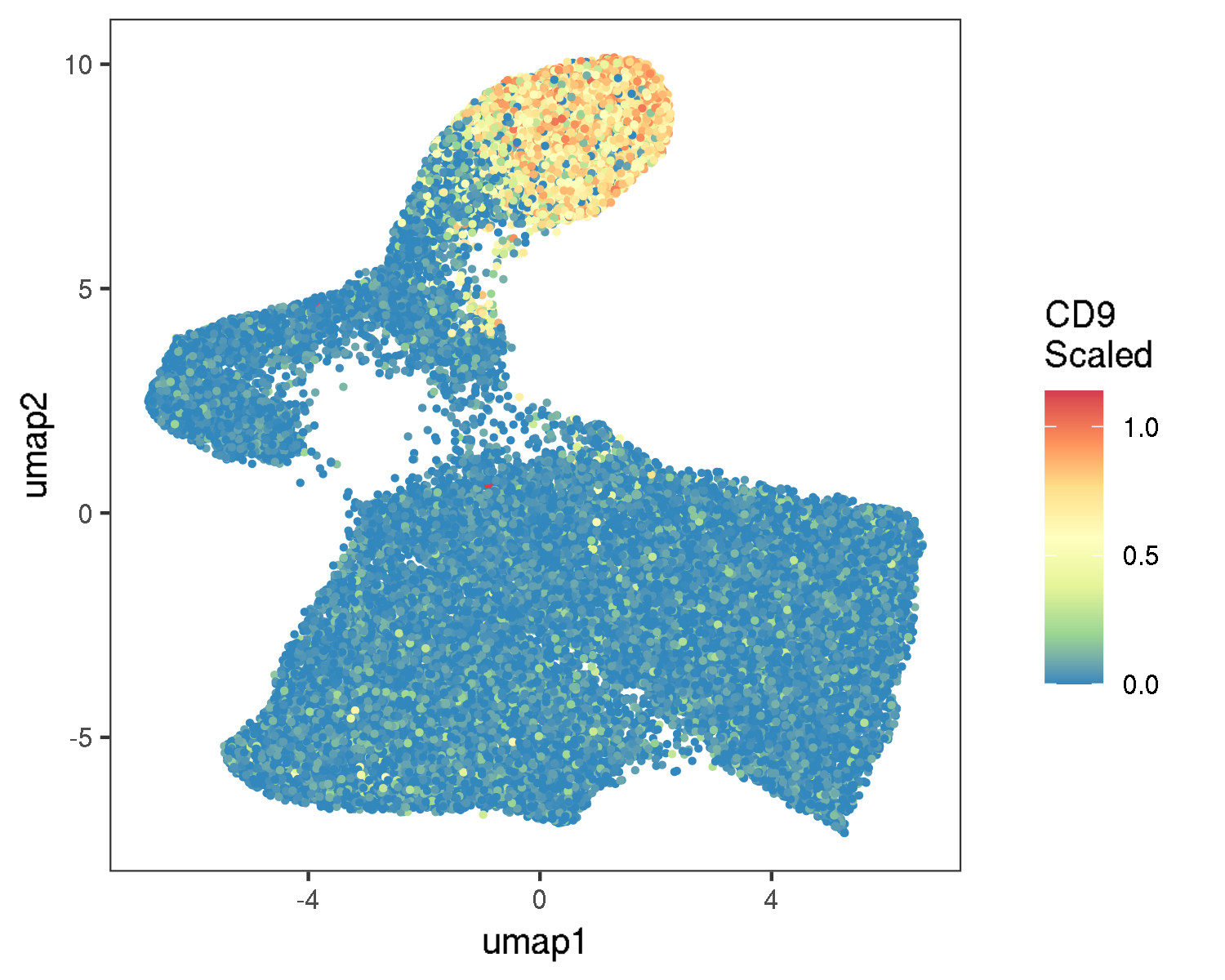

Supplement: Supplementary file 7 — Supplementary Data 4 [file 41467_2024_49883_MOESM7_ESM.zip › png/CD9.png]

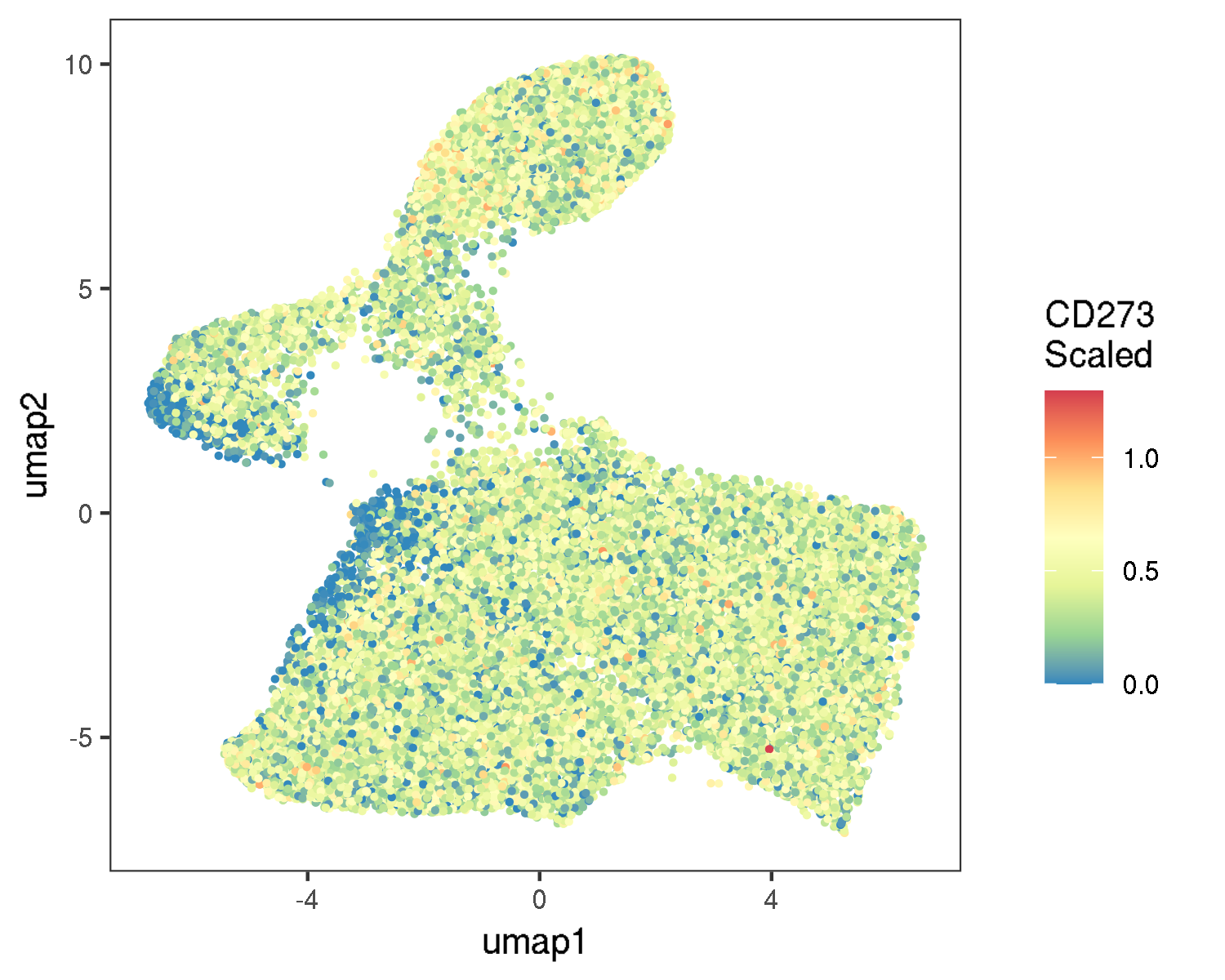

Supplement: Supplementary file 7 — Supplementary Data 4 [file 41467_2024_49883_MOESM7_ESM.zip › png/CD273.png]

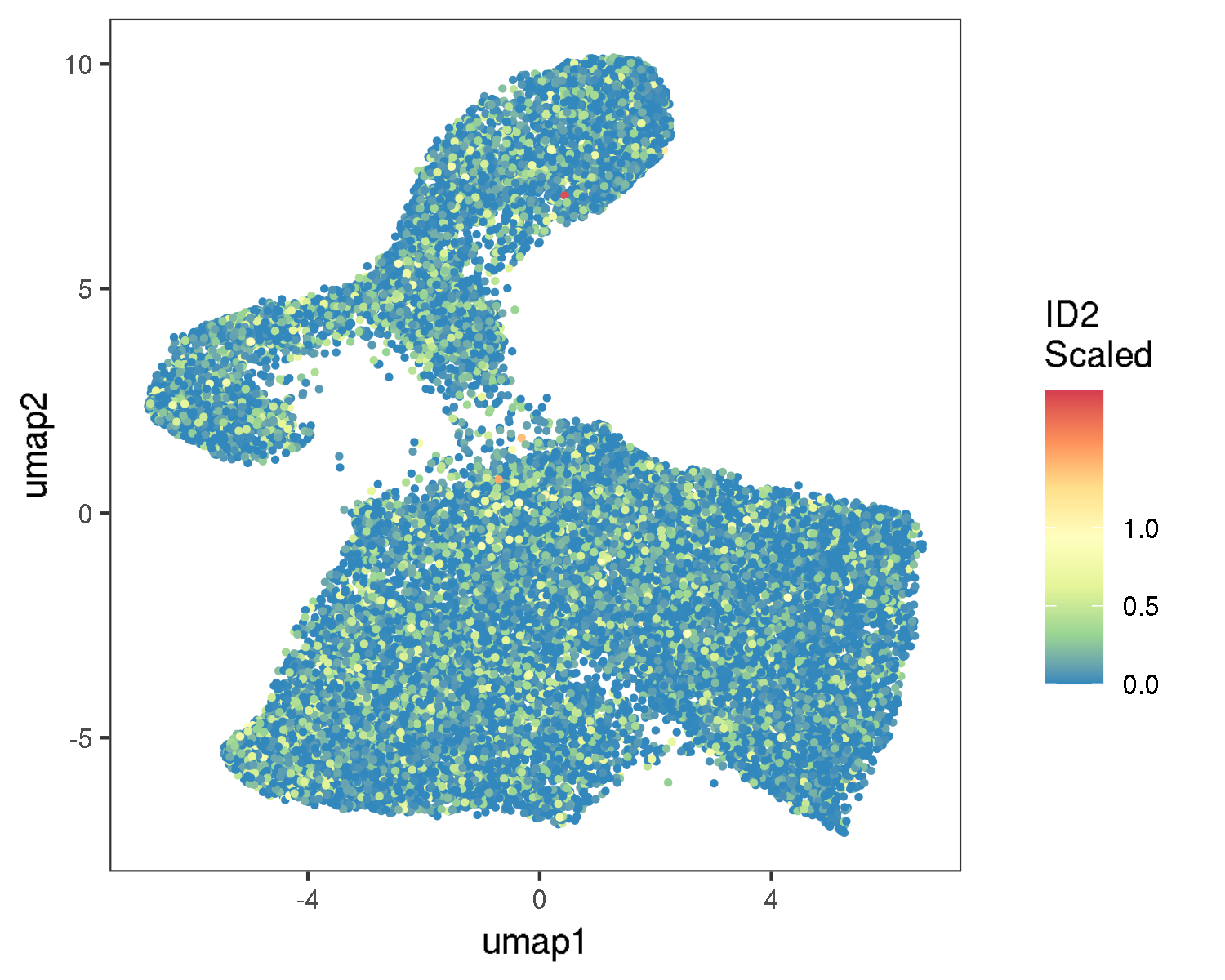

Supplement: Supplementary file 7 — Supplementary Data 4 [file 41467_2024_49883_MOESM7_ESM.zip › png/ID2.png]

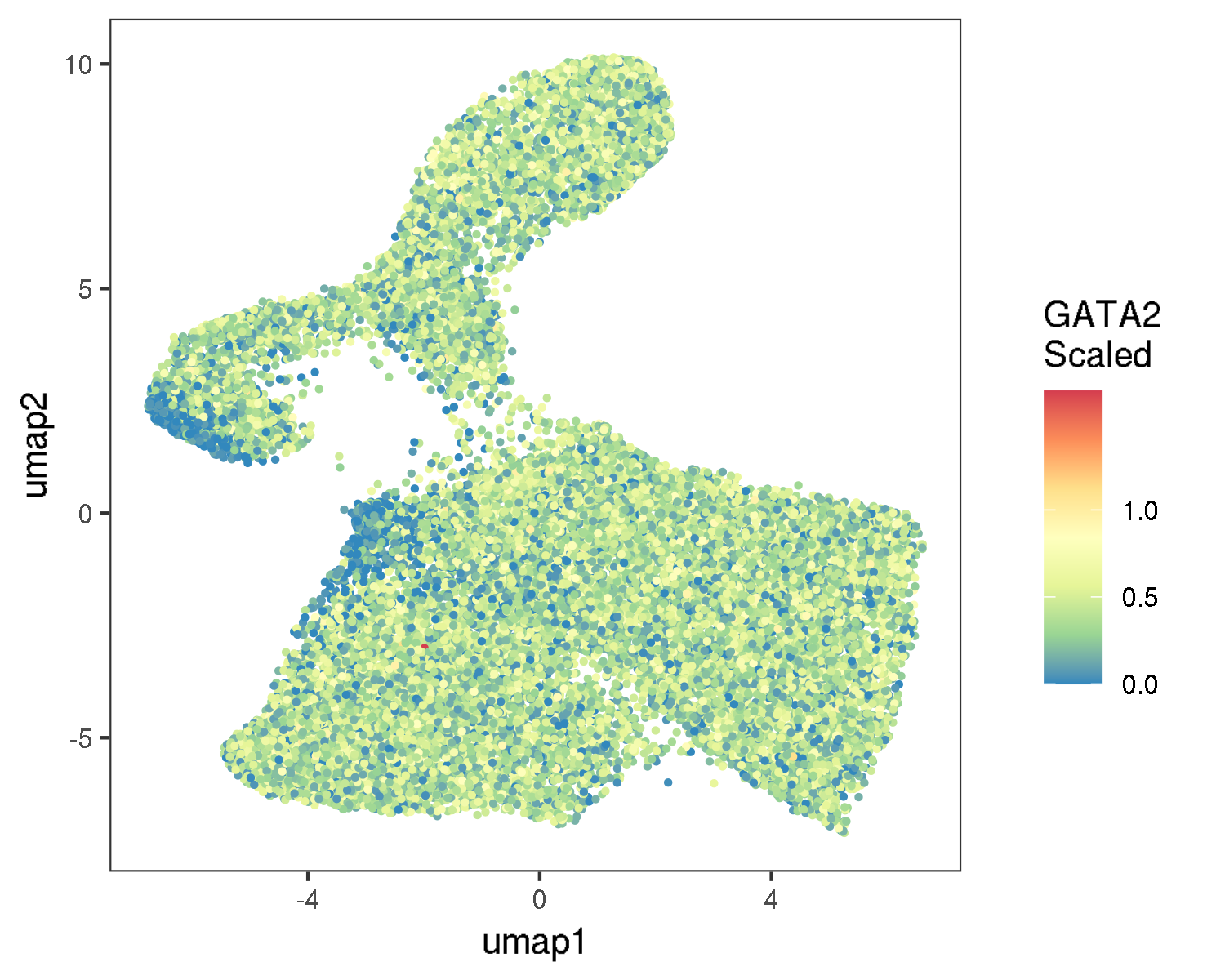

Supplement: Supplementary file 7 — Supplementary Data 4 [file 41467_2024_49883_MOESM7_ESM.zip › png/GATA2.png]

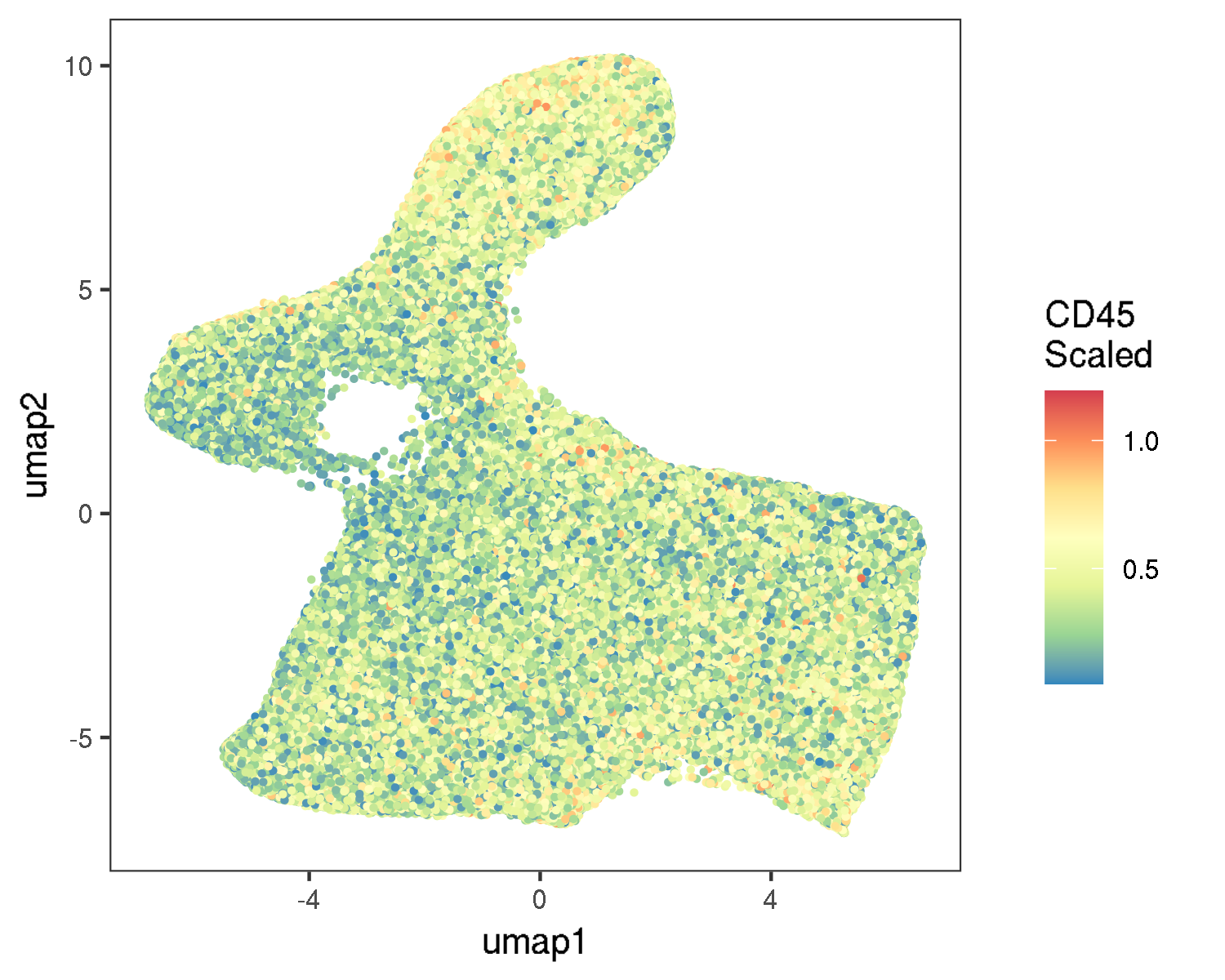

Supplement: Supplementary file 7 — Supplementary Data 4 [file 41467_2024_49883_MOESM7_ESM.zip › png/CD45.png]

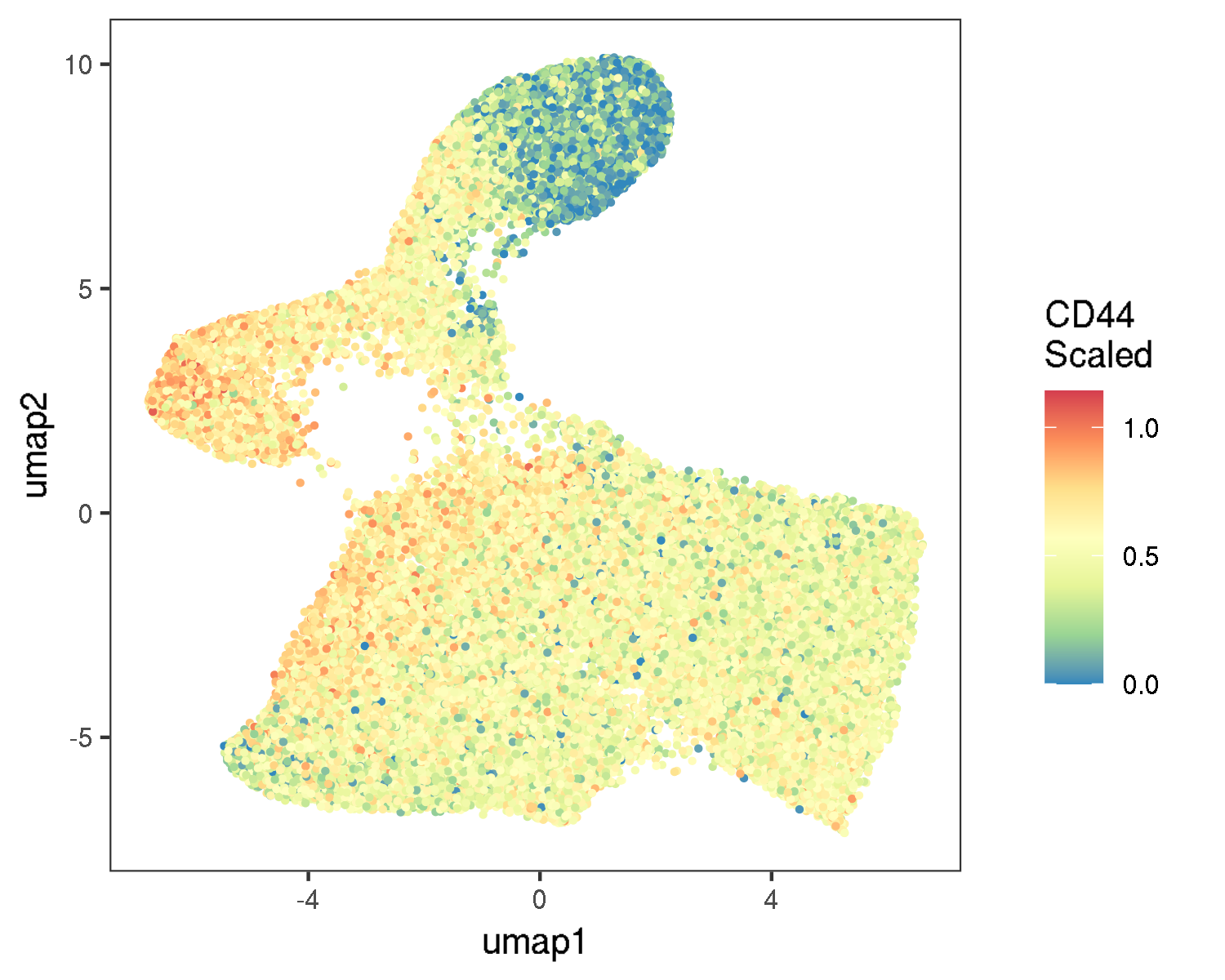

Supplement: Supplementary file 7 — Supplementary Data 4 [file 41467_2024_49883_MOESM7_ESM.zip › png/CD44.png]

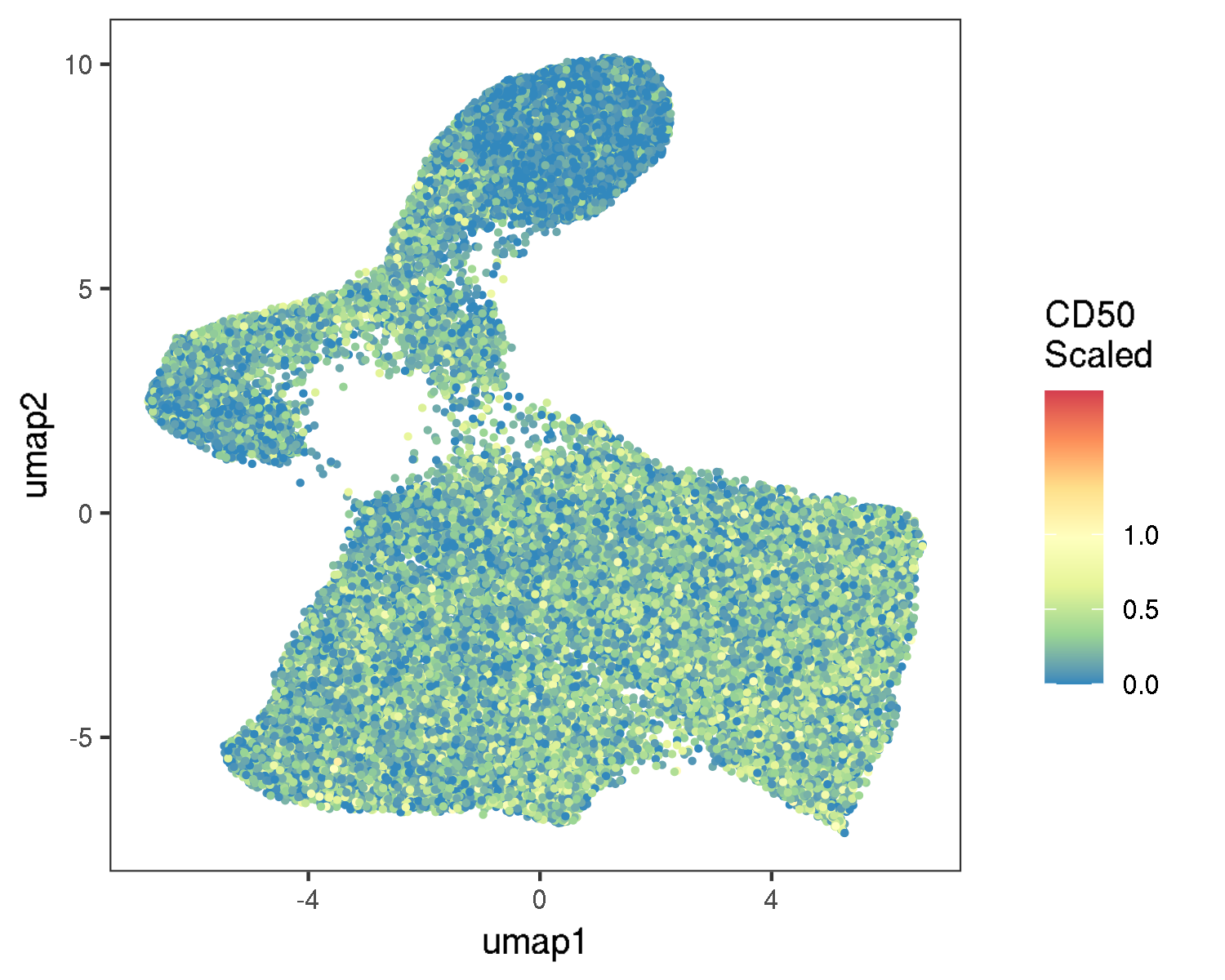

Supplement: Supplementary file 7 — Supplementary Data 4 [file 41467_2024_49883_MOESM7_ESM.zip › png/CD50.png]

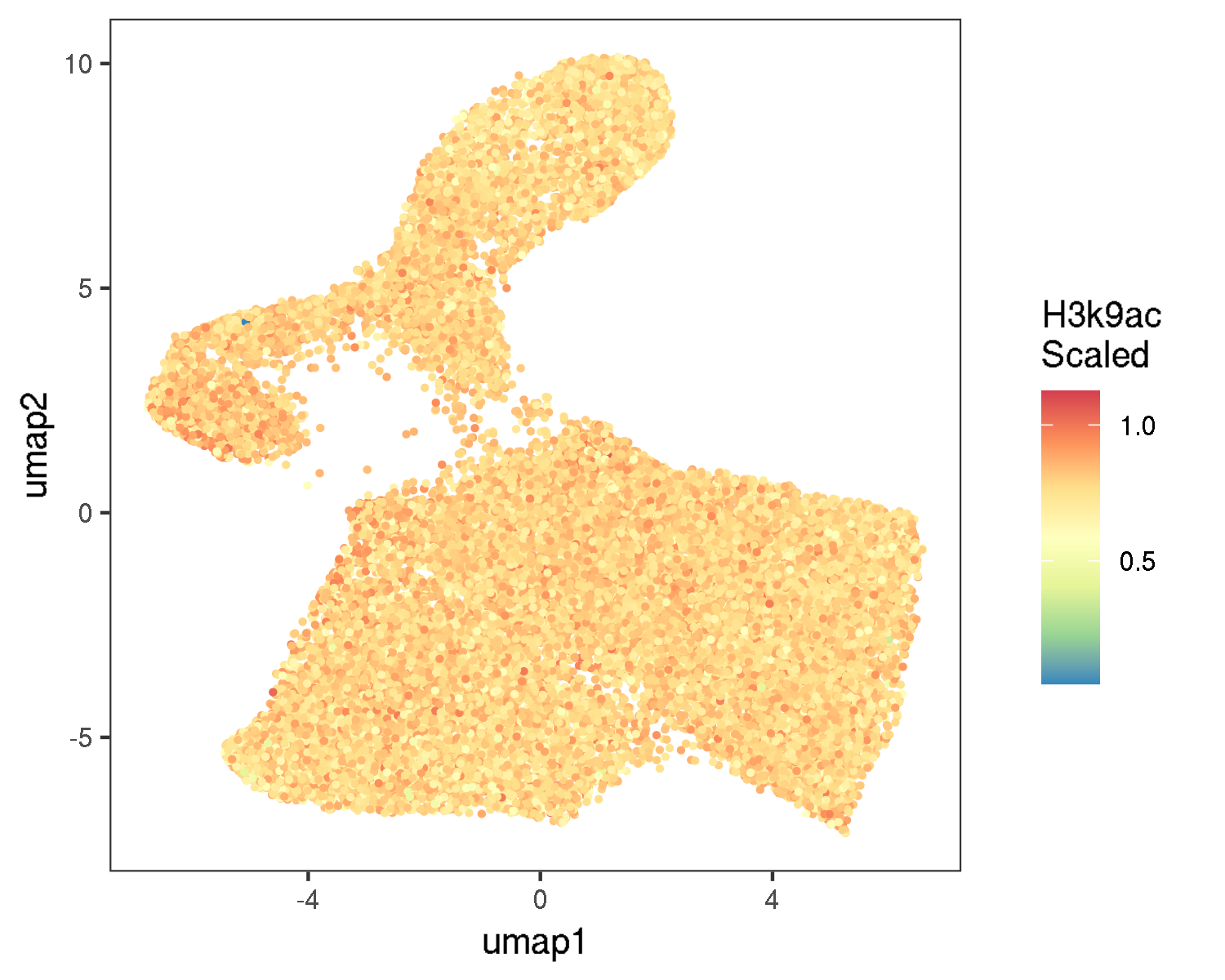

Supplement: Supplementary file 7 — Supplementary Data 4 [file 41467_2024_49883_MOESM7_ESM.zip › png/H3k9ac.png]

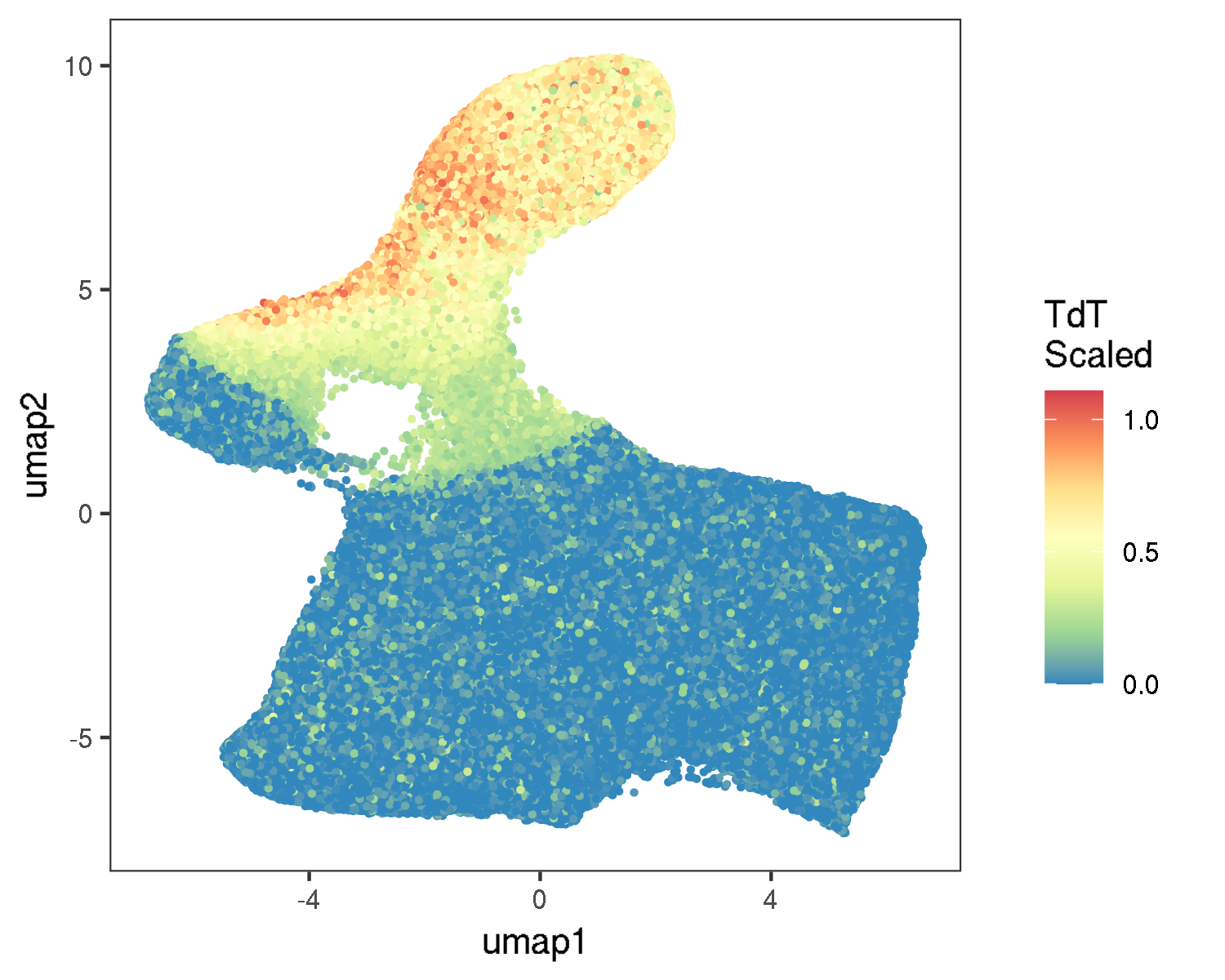

Supplement: Supplementary file 7 — Supplementary Data 4 [file 41467_2024_49883_MOESM7_ESM.zip › png/TdT.png]

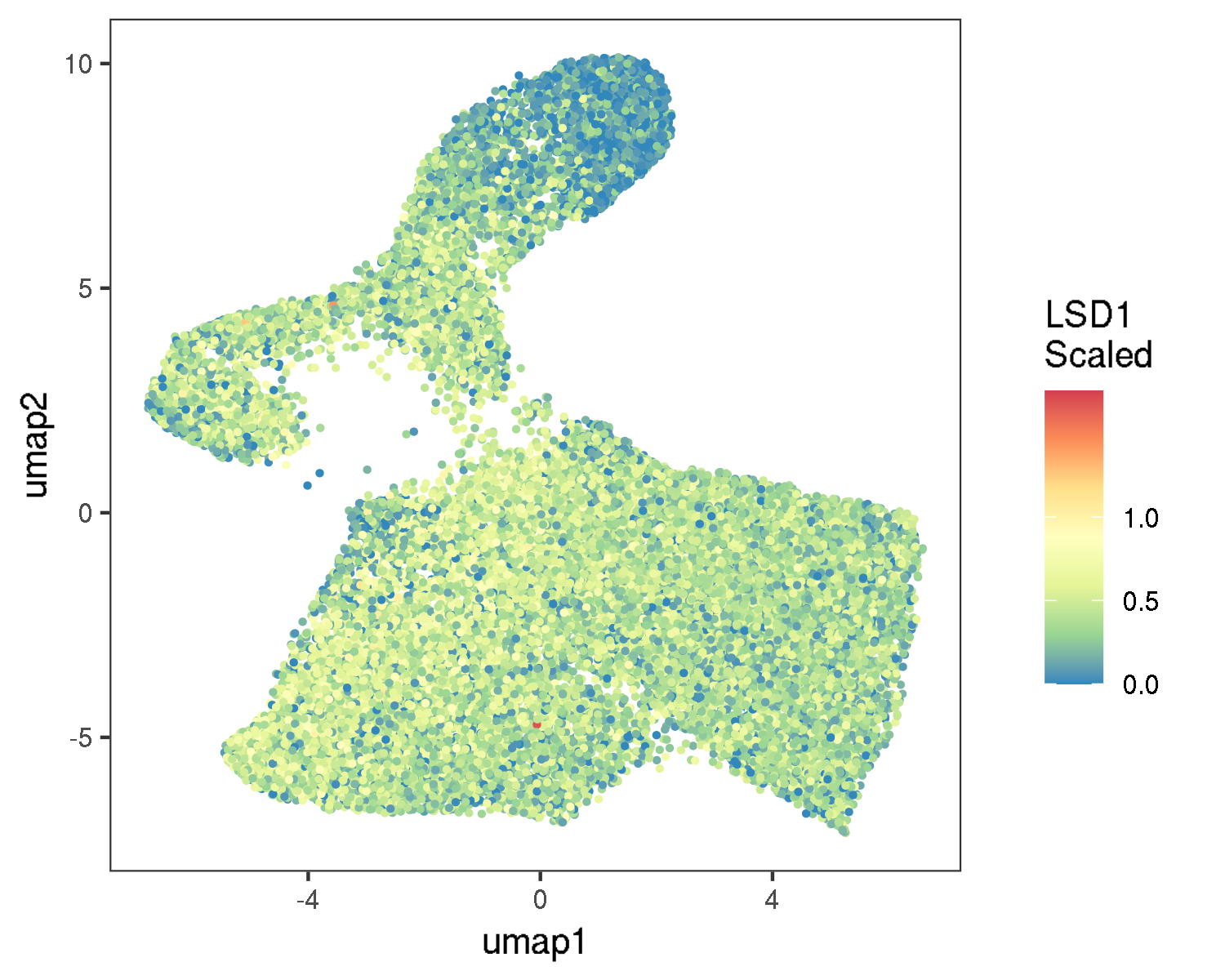

Supplement: Supplementary file 7 — Supplementary Data 4 [file 41467_2024_49883_MOESM7_ESM.zip › png/LSD1.png]

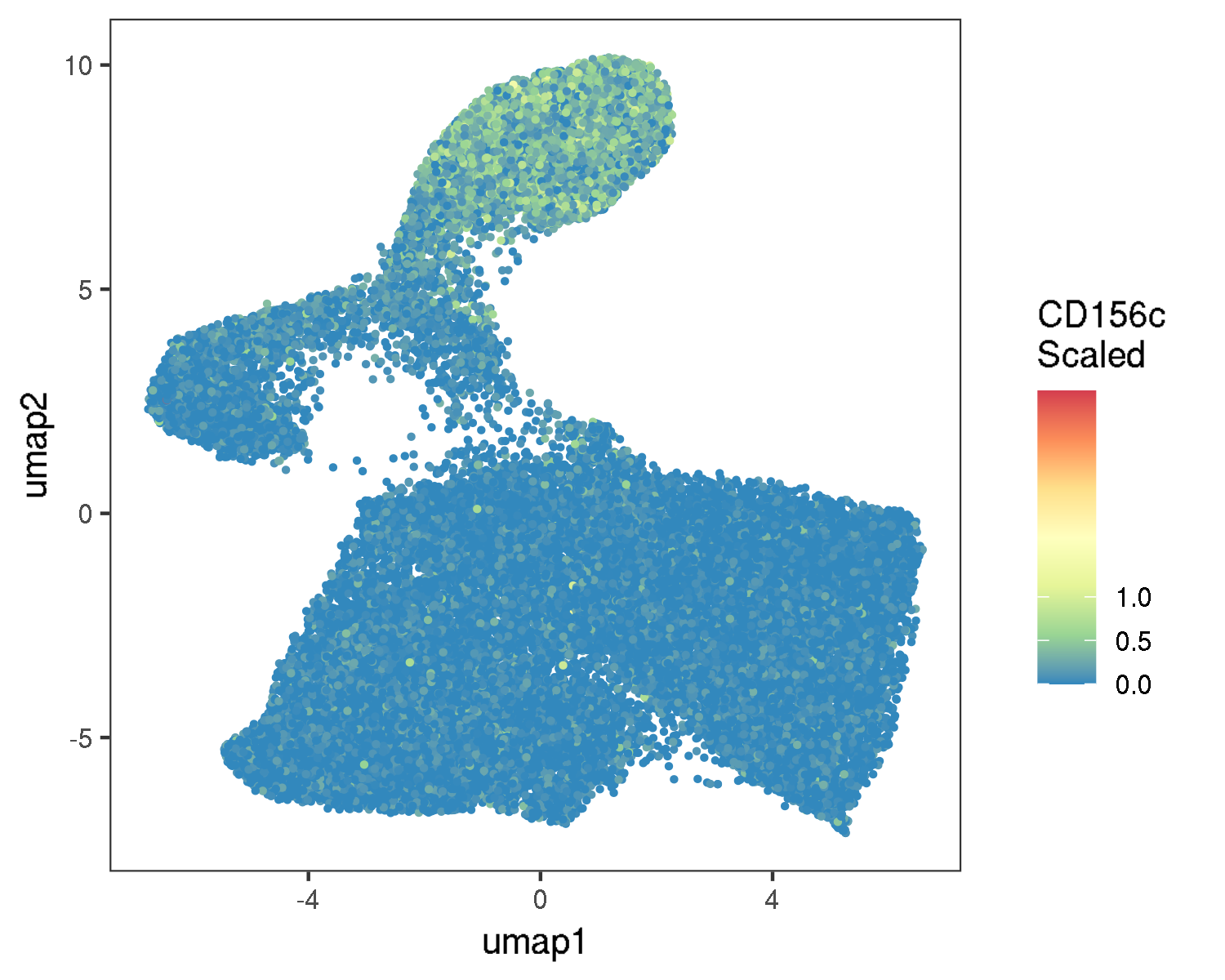

Supplement: Supplementary file 7 — Supplementary Data 4 [file 41467_2024_49883_MOESM7_ESM.zip › png/CD156c.png]

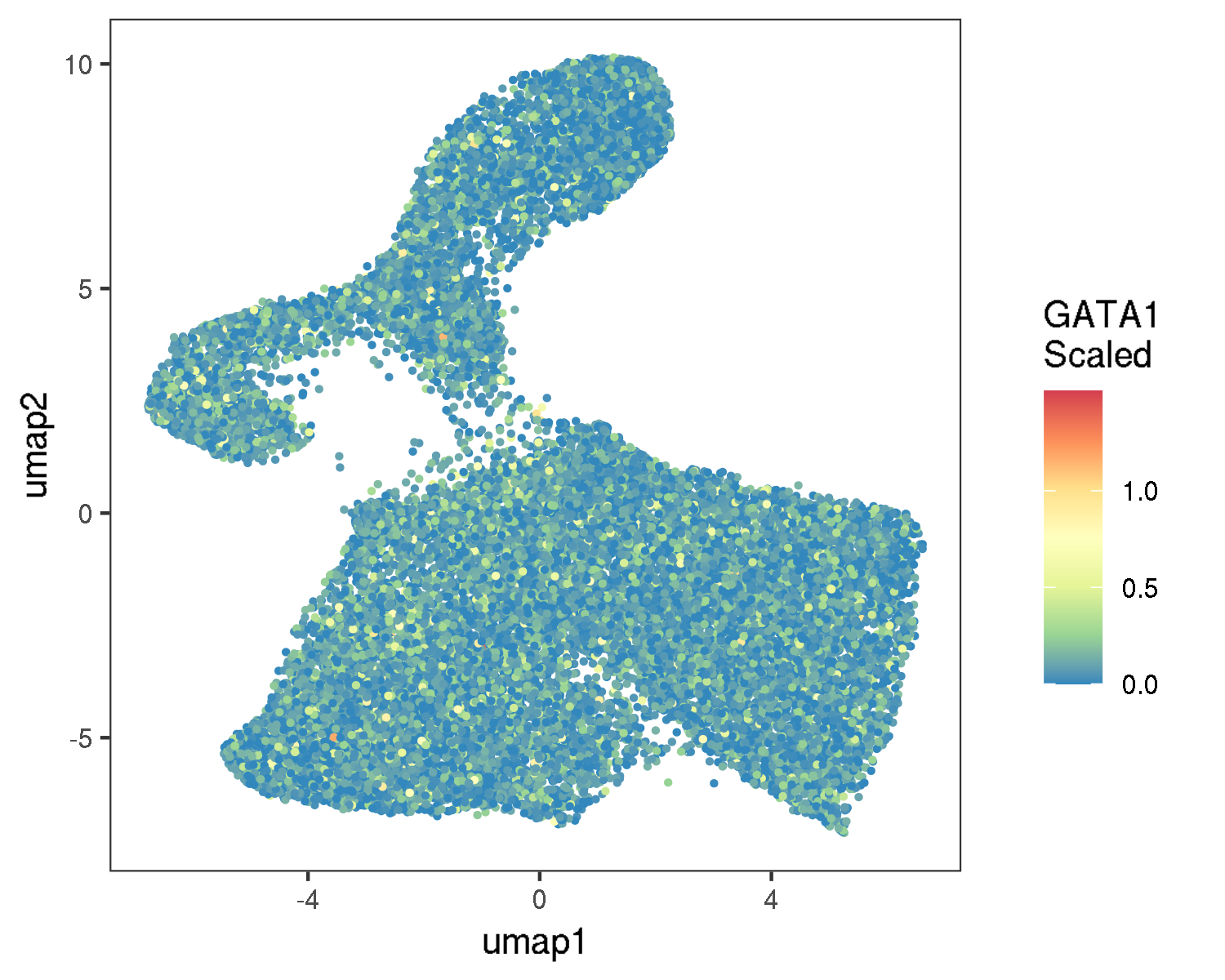

Supplement: Supplementary file 7 — Supplementary Data 4 [file 41467_2024_49883_MOESM7_ESM.zip › png/GATA1.png]

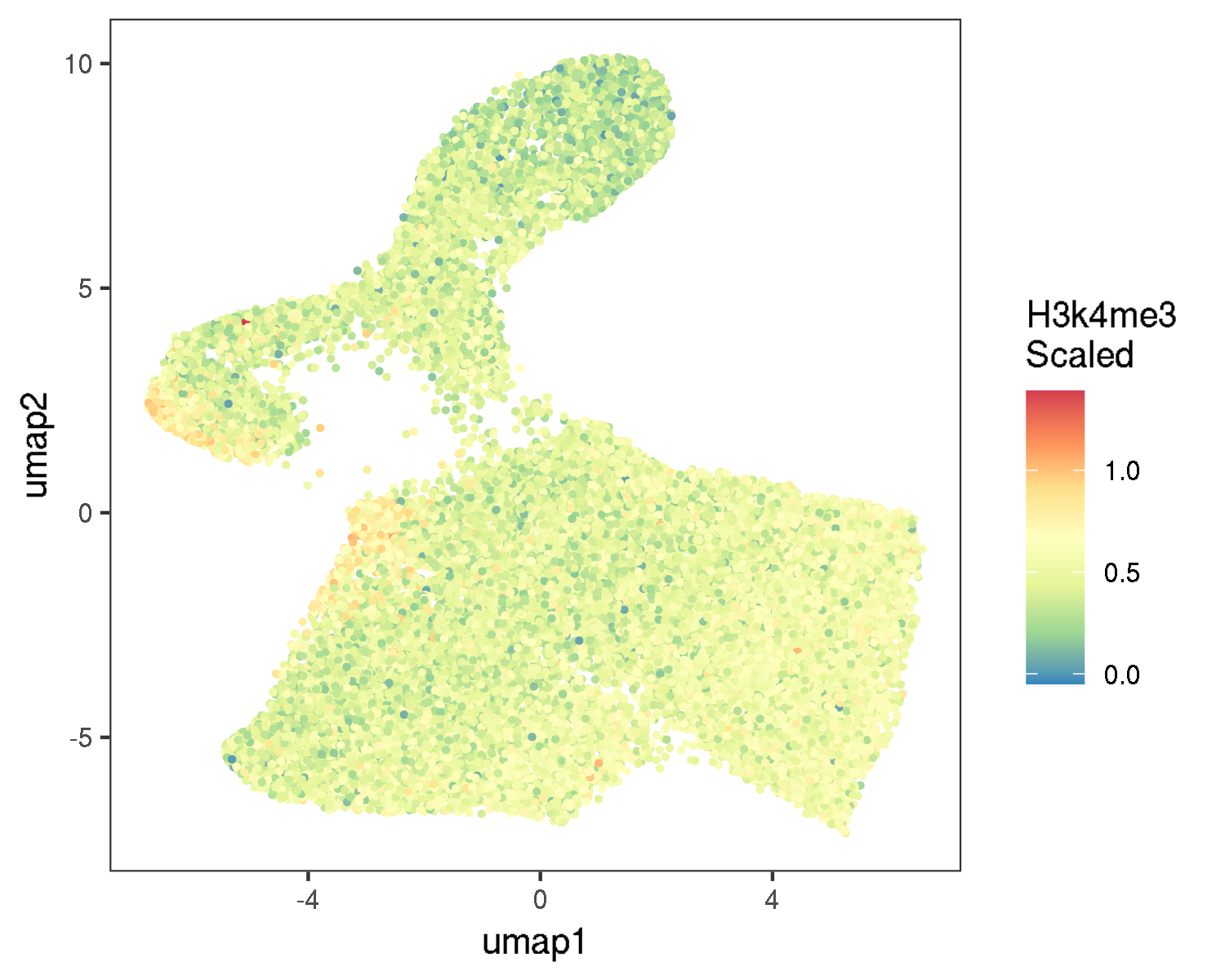

Supplement: Supplementary file 7 — Supplementary Data 4 [file 41467_2024_49883_MOESM7_ESM.zip › png/H3k4me3.png]

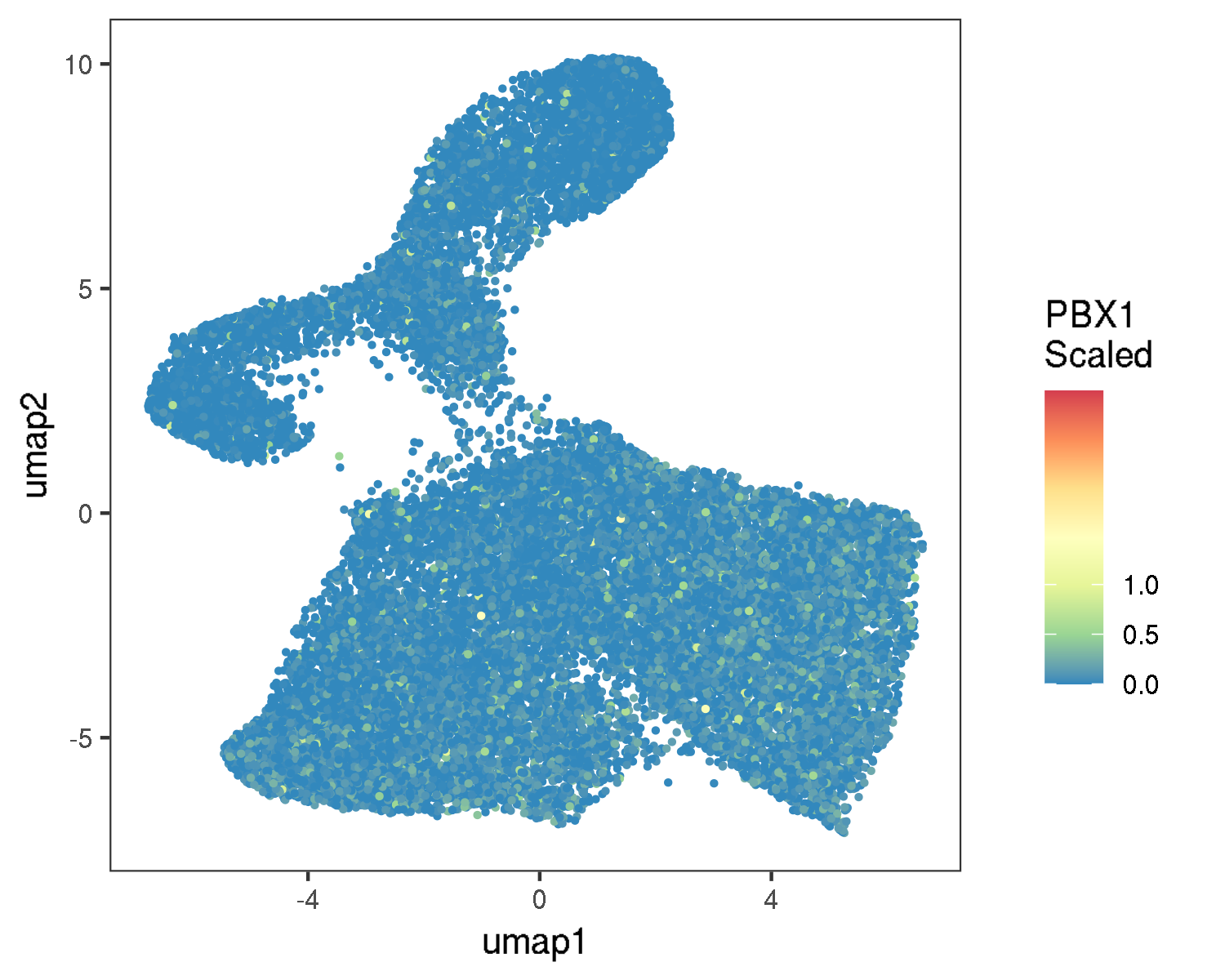

Supplement: Supplementary file 7 — Supplementary Data 4 [file 41467_2024_49883_MOESM7_ESM.zip › png/PBX1.png]

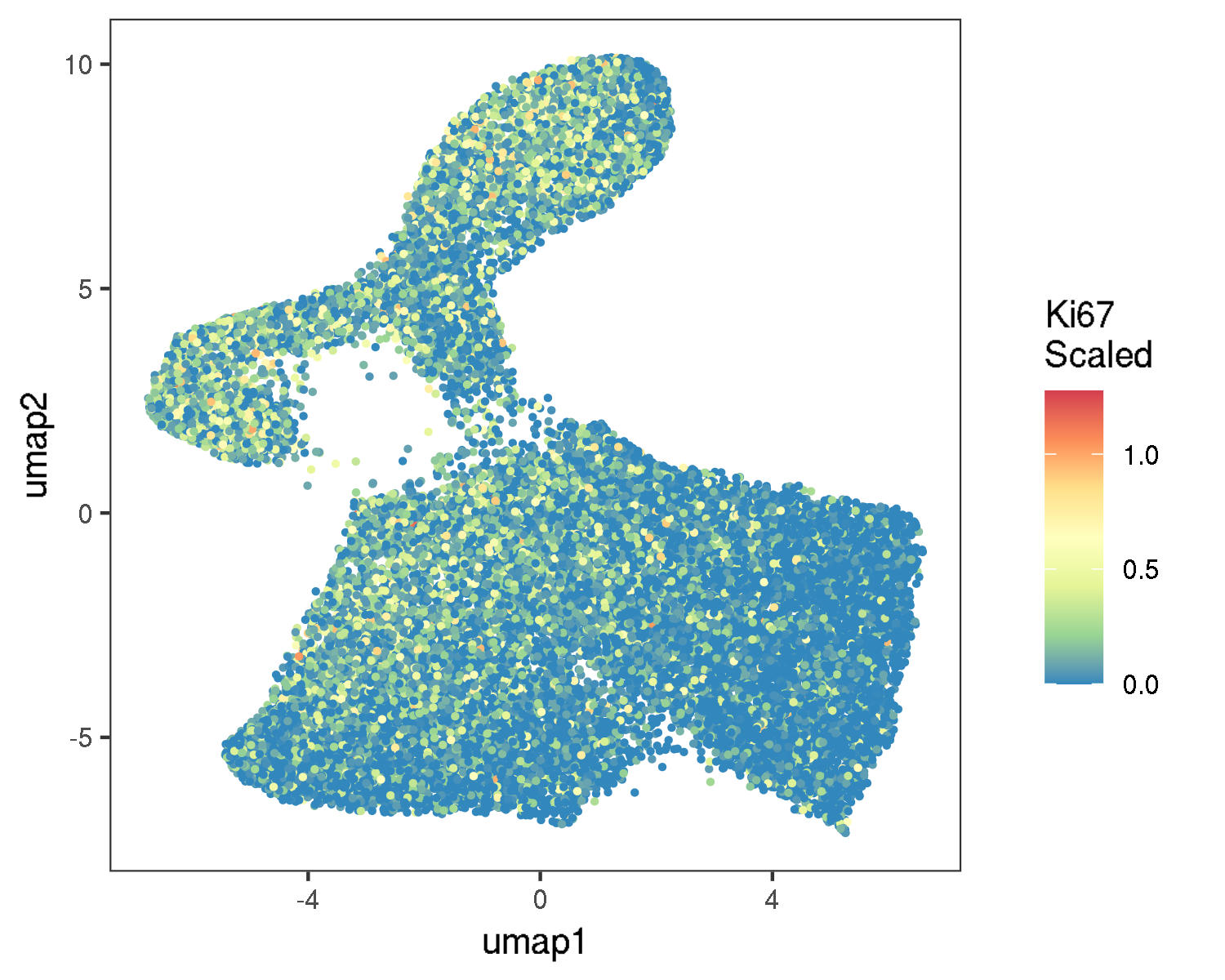

Supplement: Supplementary file 7 — Supplementary Data 4 [file 41467_2024_49883_MOESM7_ESM.zip › png/Ki67.png]

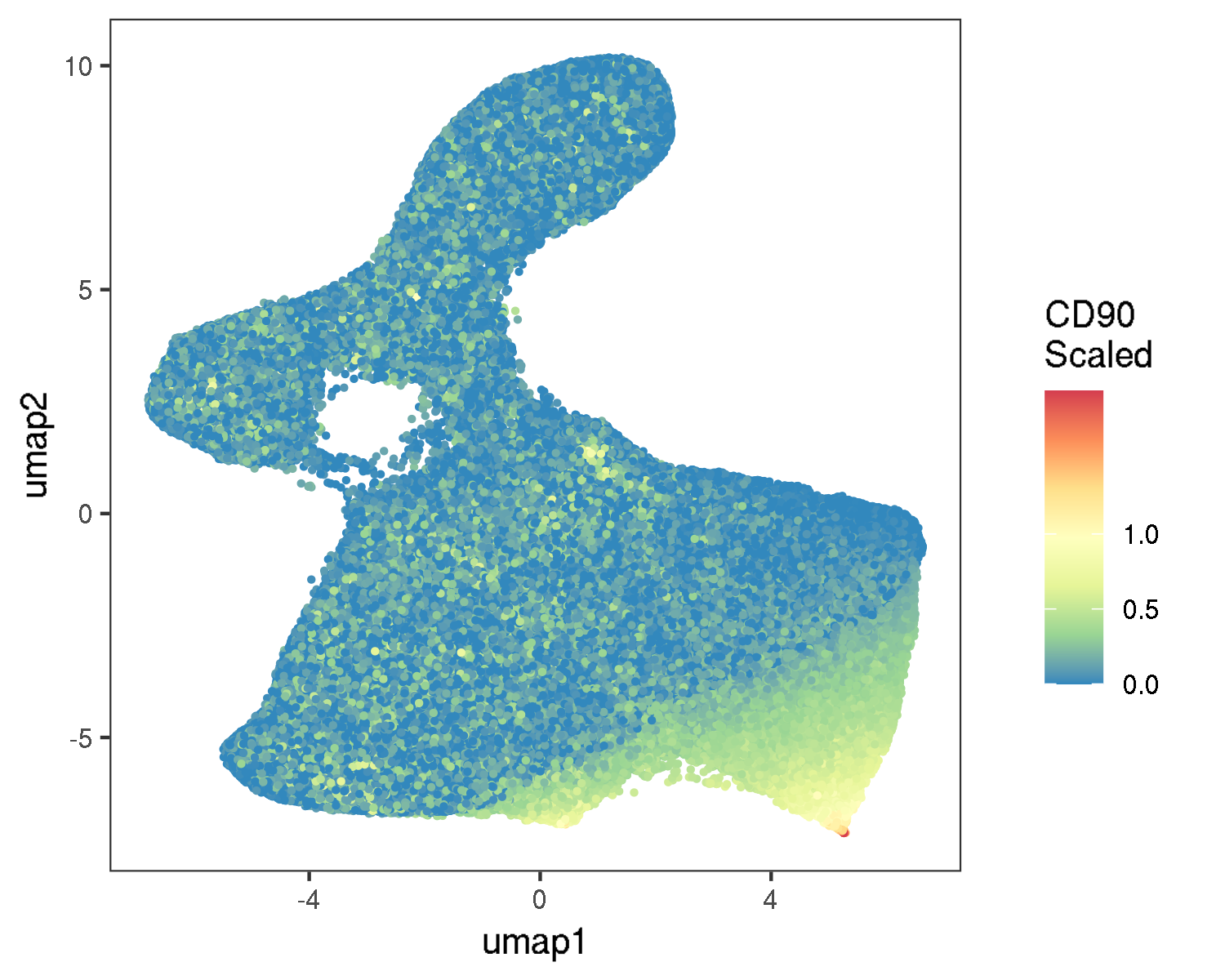

Supplement: Supplementary file 7 — Supplementary Data 4 [file 41467_2024_49883_MOESM7_ESM.zip › png/CD90.png]

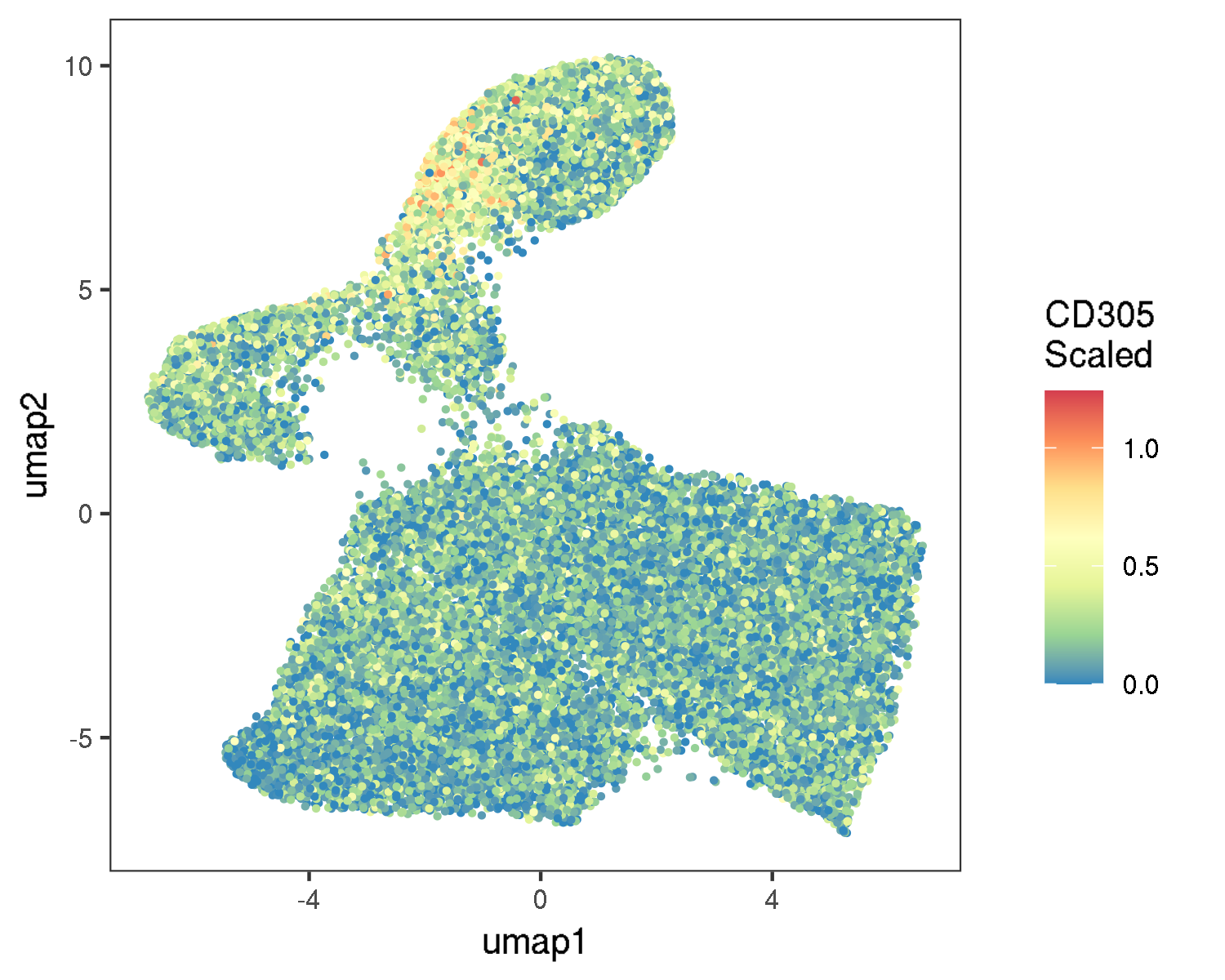

Supplement: Supplementary file 7 — Supplementary Data 4 [file 41467_2024_49883_MOESM7_ESM.zip › png/CD305.png]

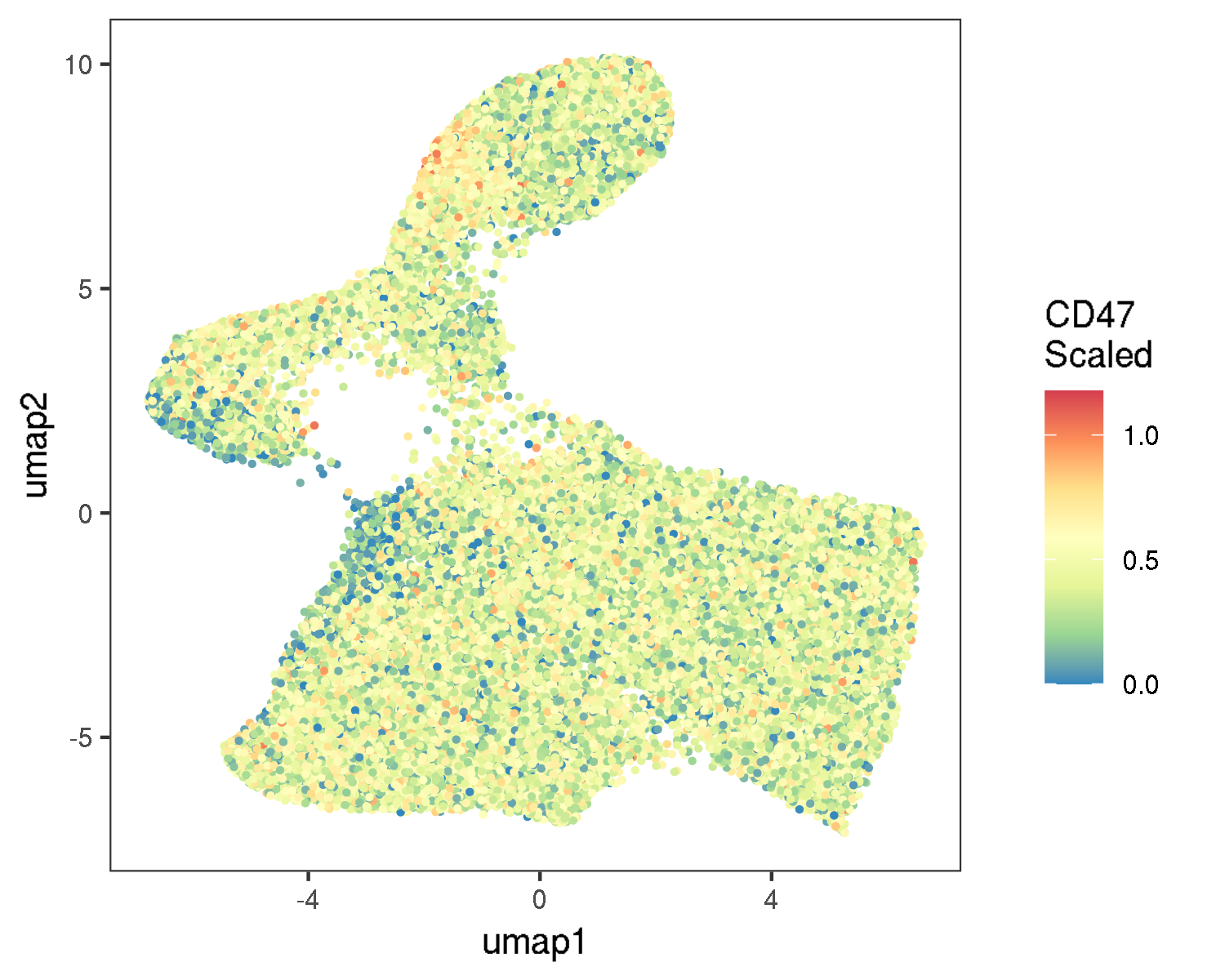

Supplement: Supplementary file 7 — Supplementary Data 4 [file 41467_2024_49883_MOESM7_ESM.zip › png/CD47.png]

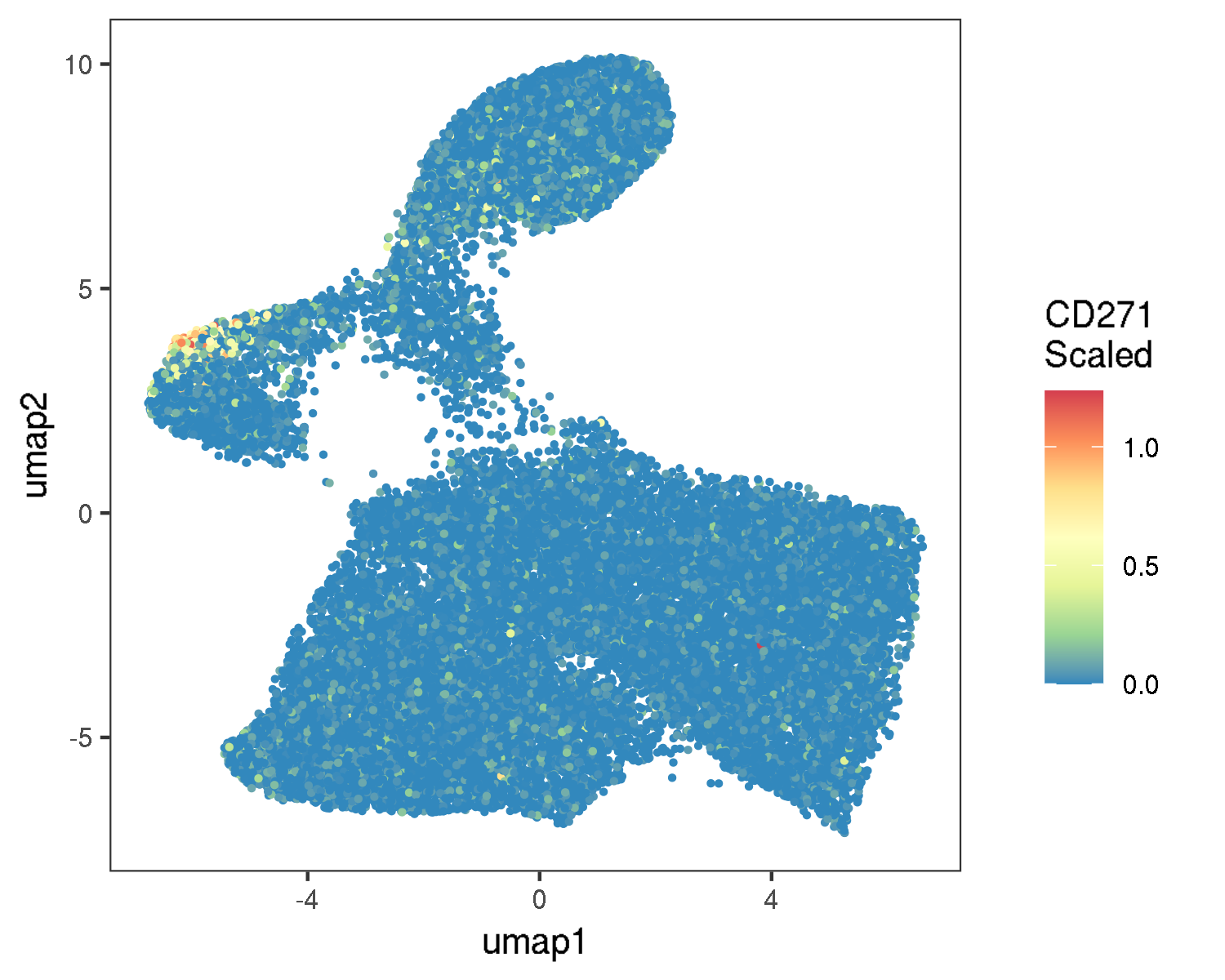

Supplement: Supplementary file 7 — Supplementary Data 4 [file 41467_2024_49883_MOESM7_ESM.zip › png/CD271.png]

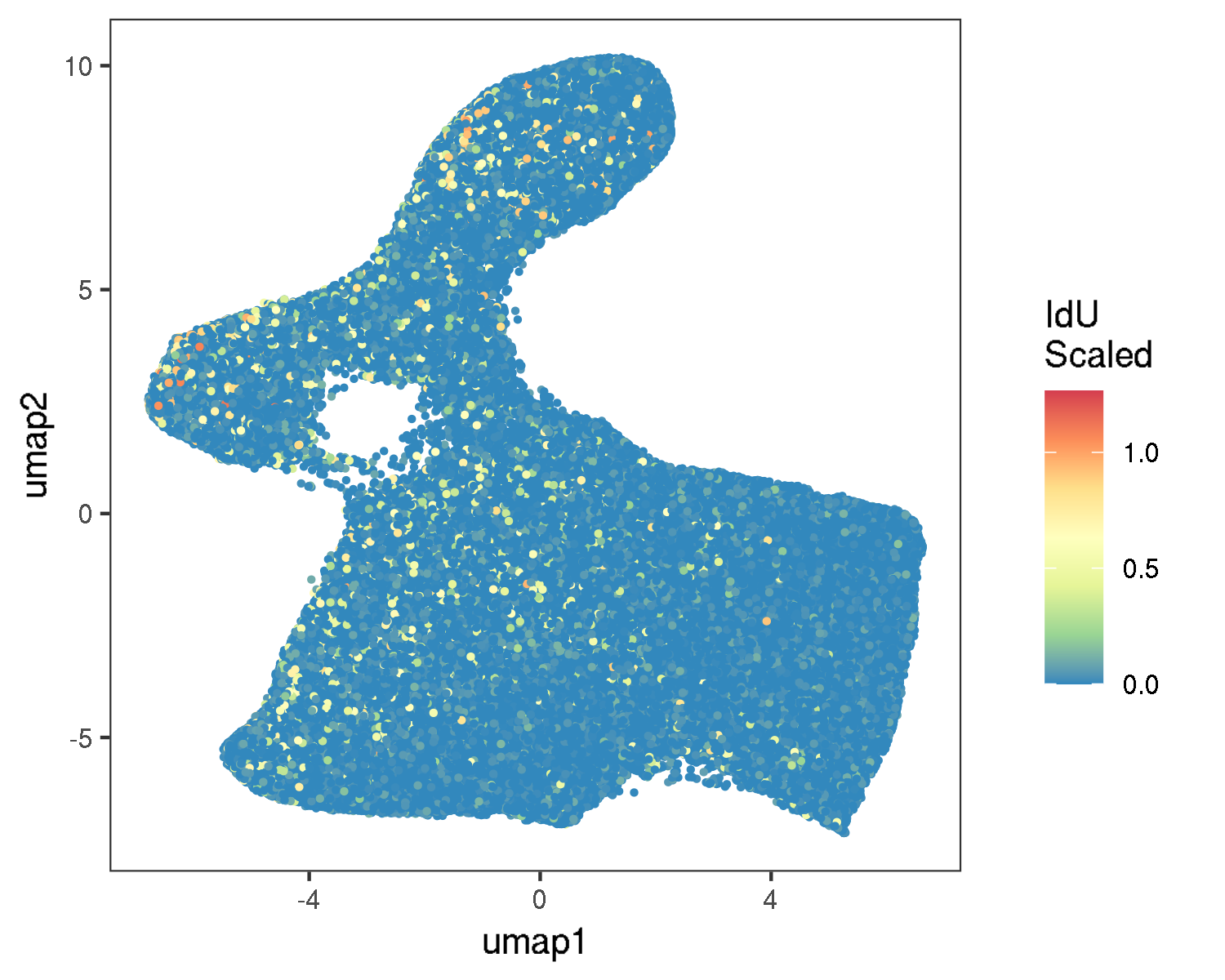

Supplement: Supplementary file 7 — Supplementary Data 4 [file 41467_2024_49883_MOESM7_ESM.zip › png/IdU.png]

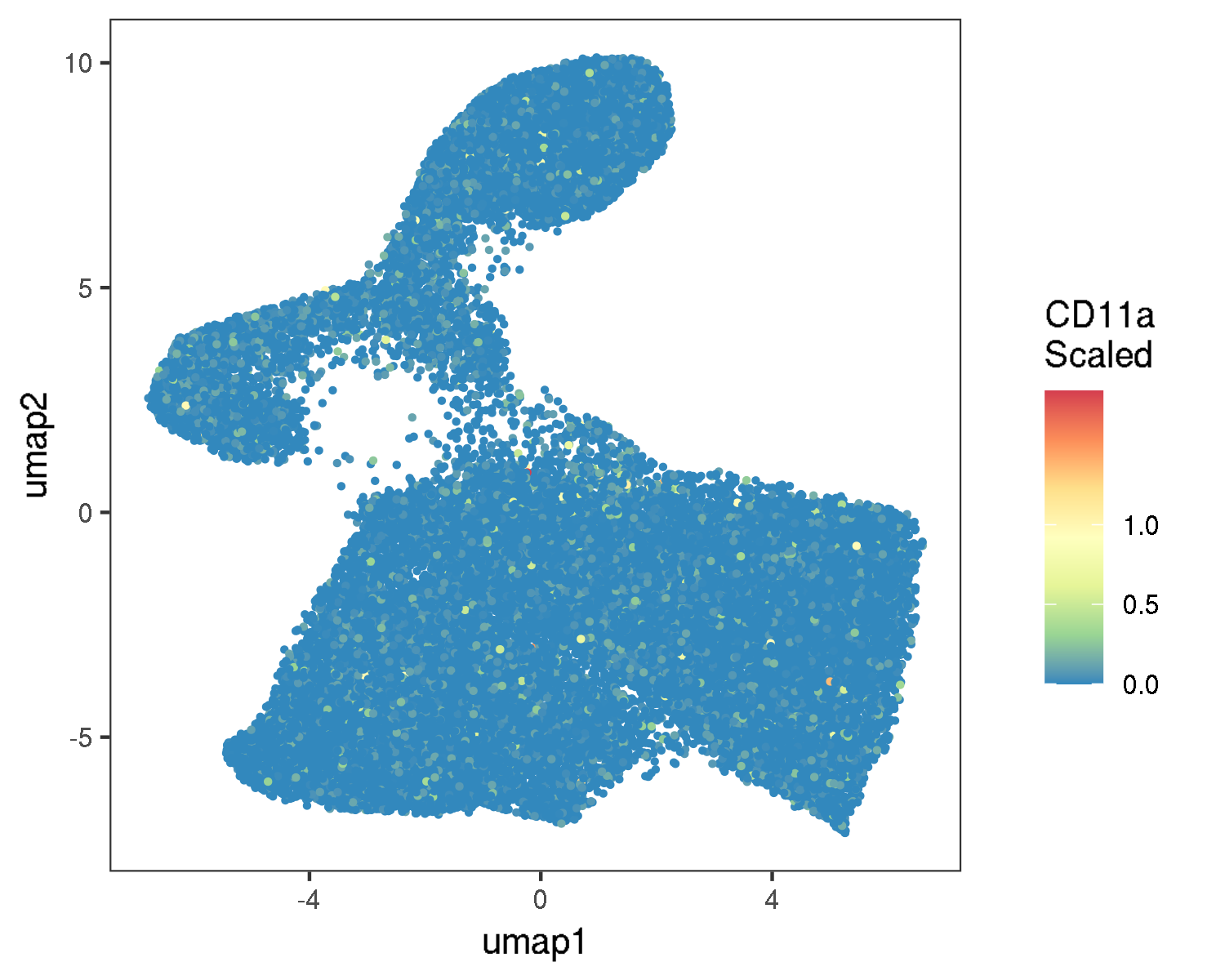

Supplement: Supplementary file 7 — Supplementary Data 4 [file 41467_2024_49883_MOESM7_ESM.zip › png/CD11a.png]

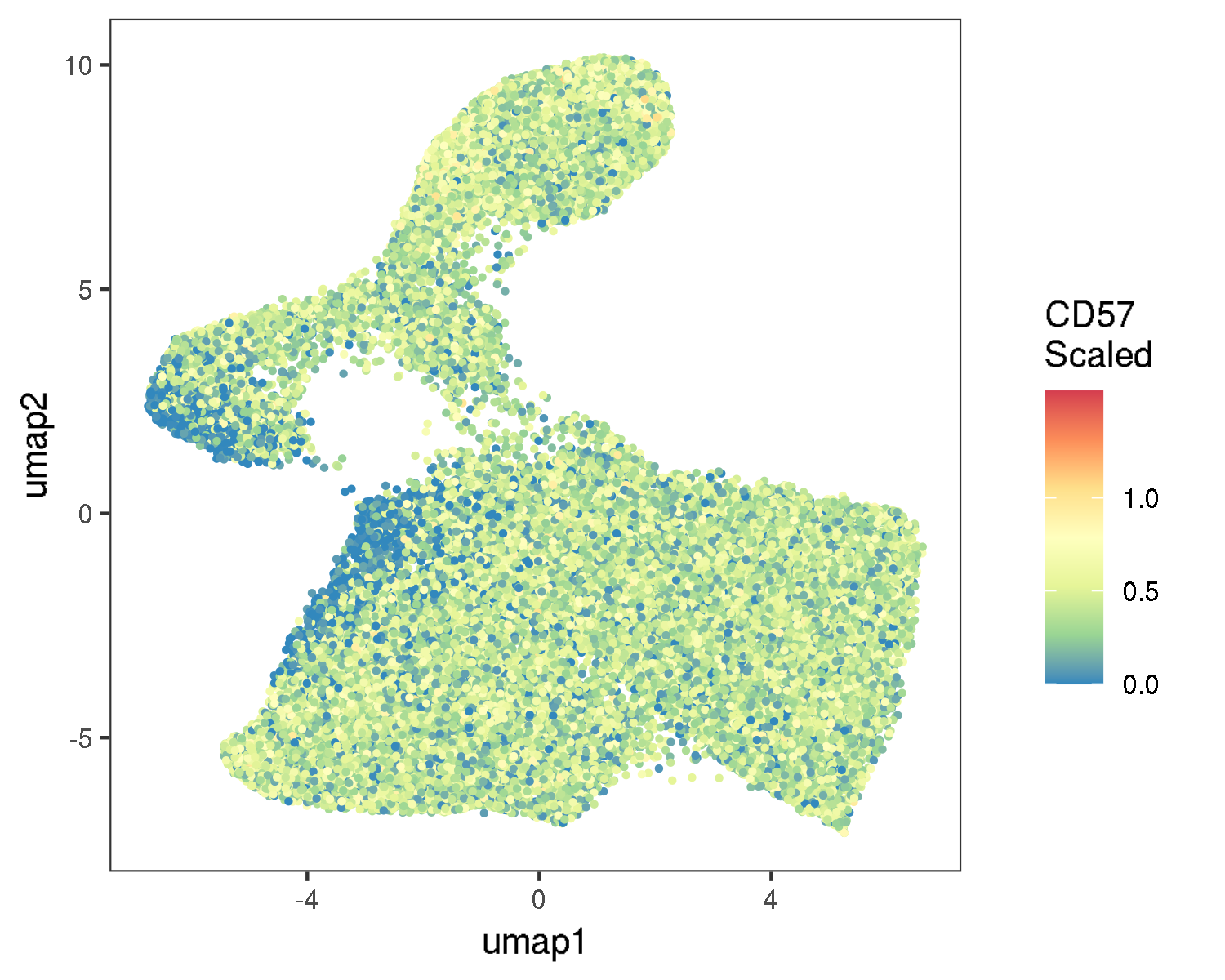

Supplement: Supplementary file 7 — Supplementary Data 4 [file 41467_2024_49883_MOESM7_ESM.zip › png/CD57.png]

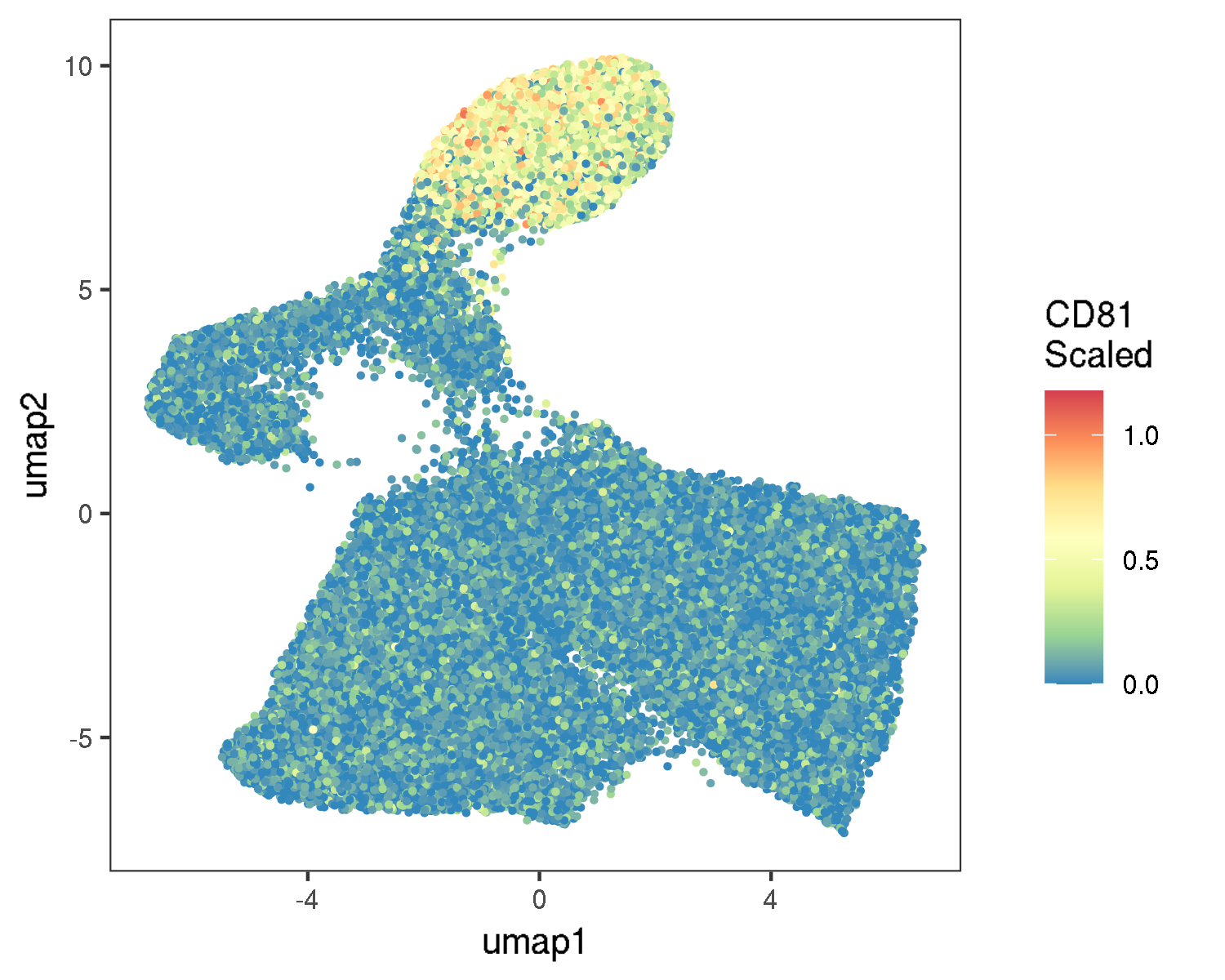

Supplement: Supplementary file 7 — Supplementary Data 4 [file 41467_2024_49883_MOESM7_ESM.zip › png/CD81.png]

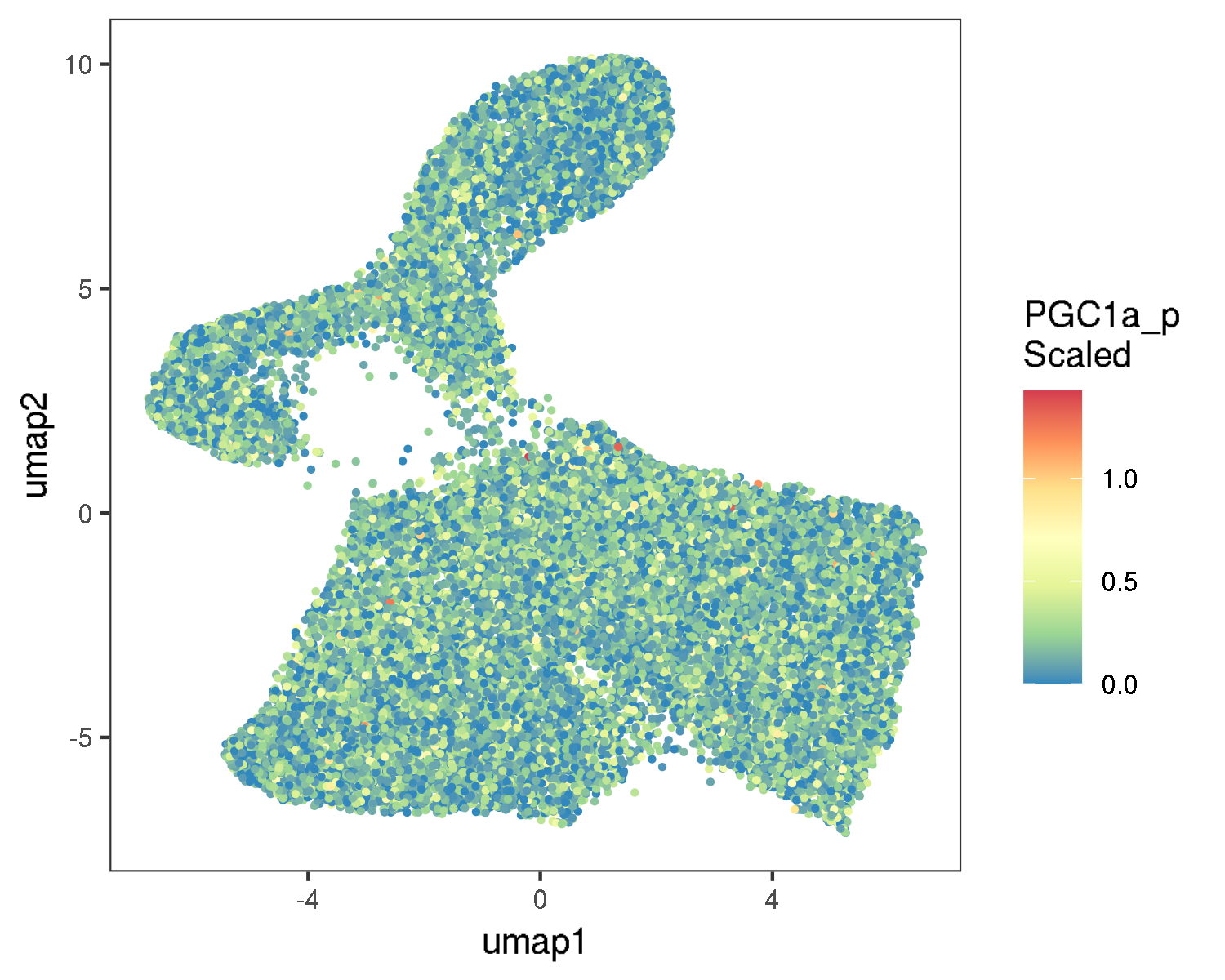

Supplement: Supplementary file 7 — Supplementary Data 4 [file 41467_2024_49883_MOESM7_ESM.zip › png/PGC1a_p.png]

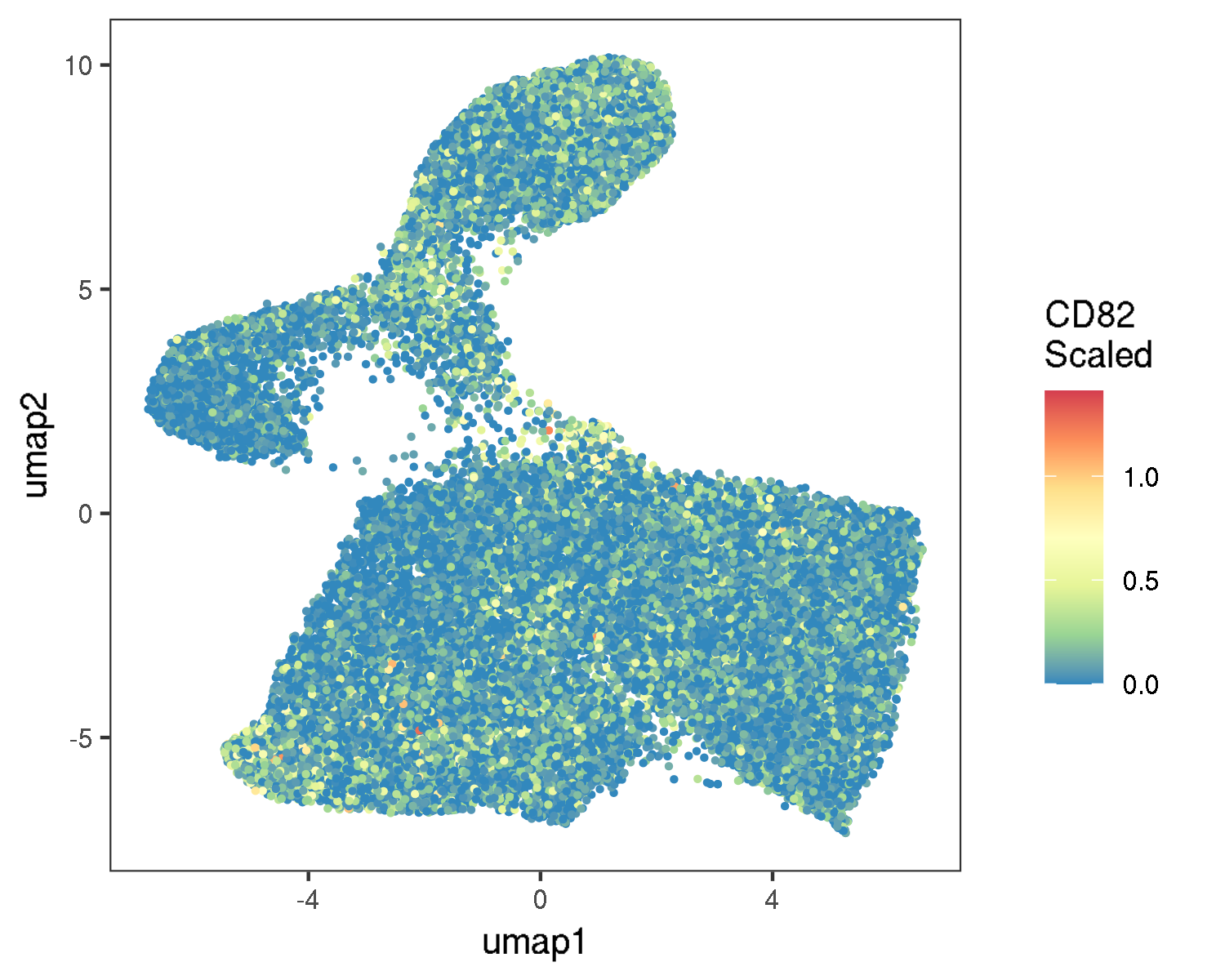

Supplement: Supplementary file 7 — Supplementary Data 4 [file 41467_2024_49883_MOESM7_ESM.zip › png/CD82.png]

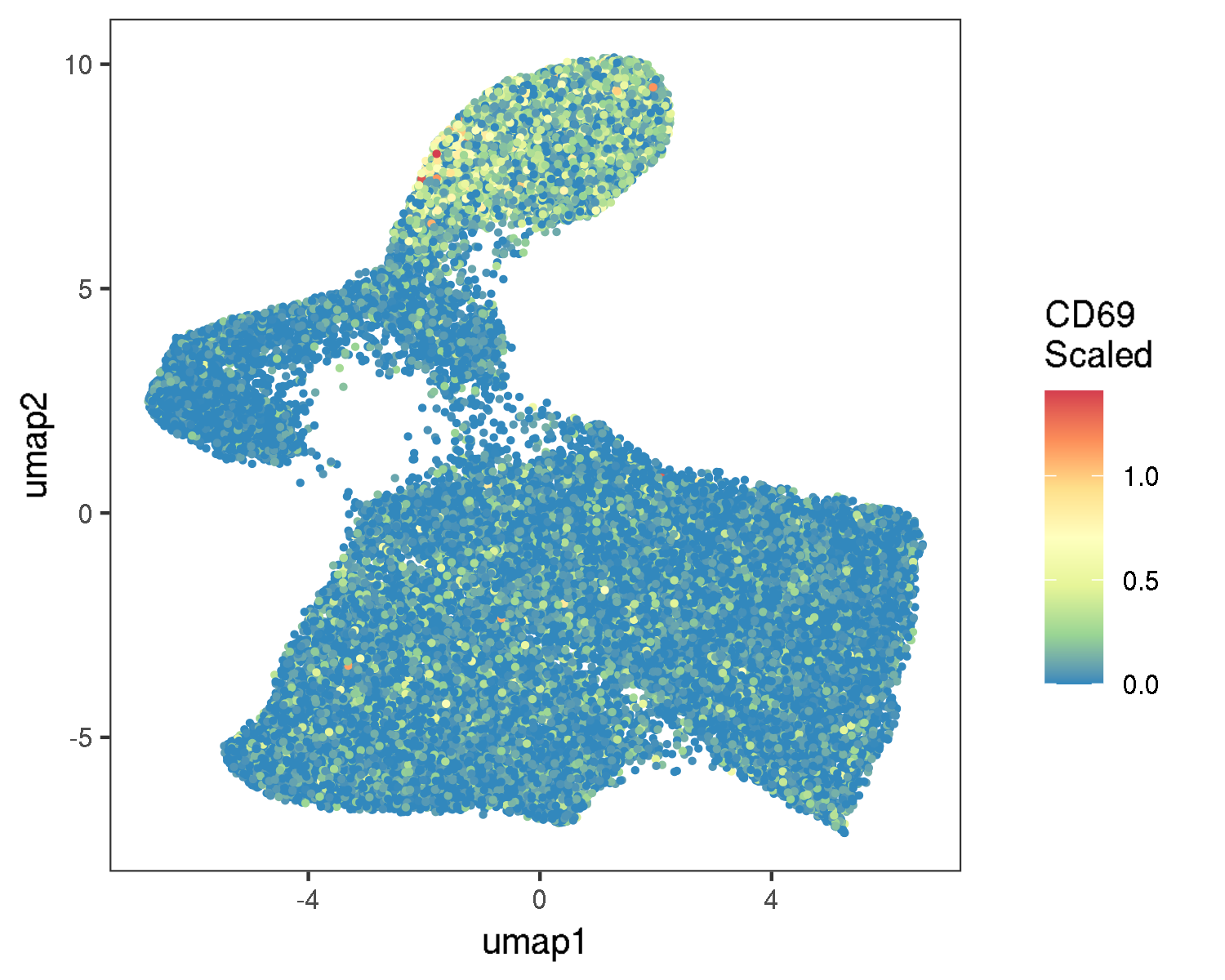

Supplement: Supplementary file 7 — Supplementary Data 4 [file 41467_2024_49883_MOESM7_ESM.zip › png/CD69.png]

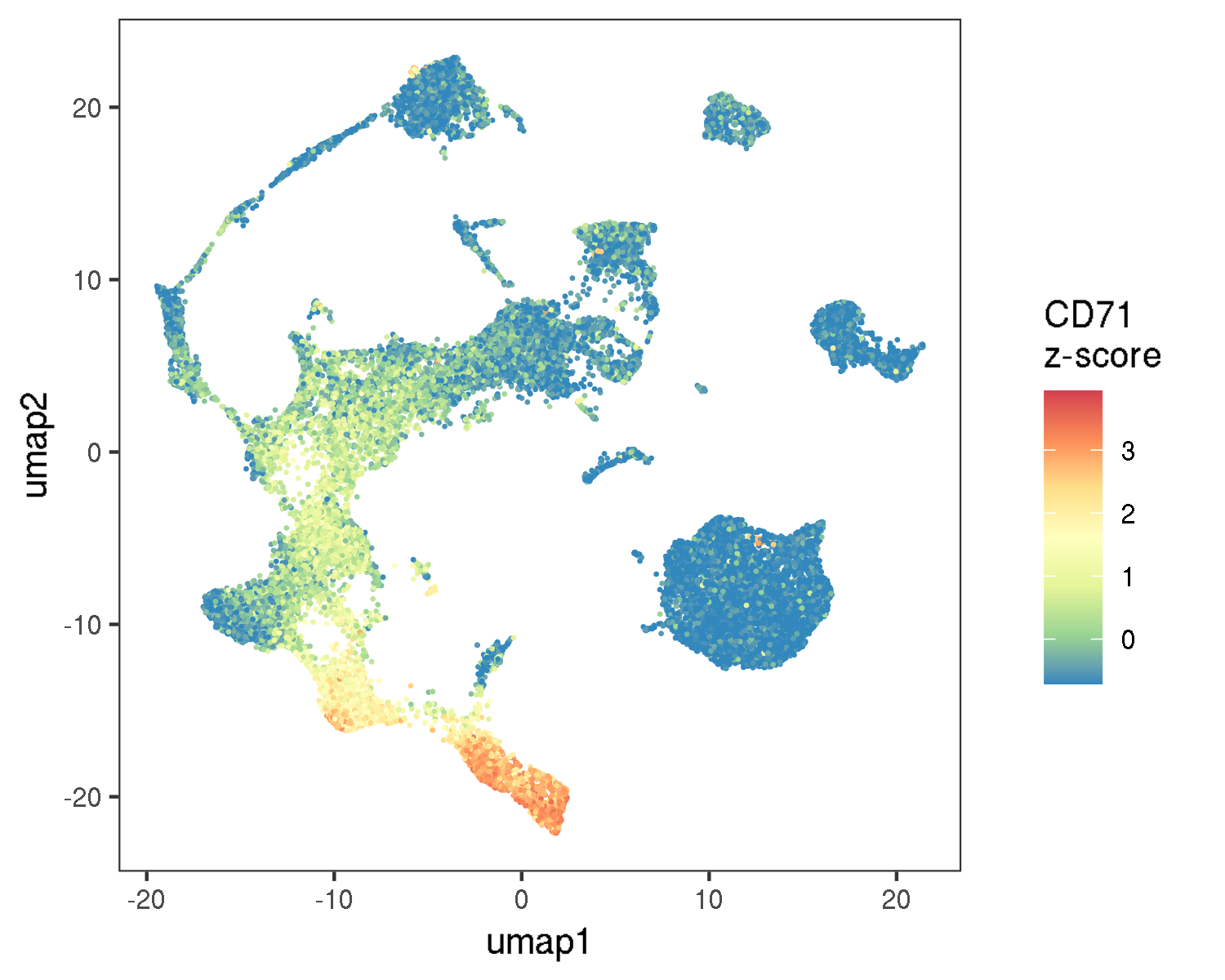

Supplement: Supplementary file 8 — Supplementary Data 5 [file 41467_2024_49883_MOESM8_ESM.zip › BMMC_final_panel_all_markers/CD71.png]

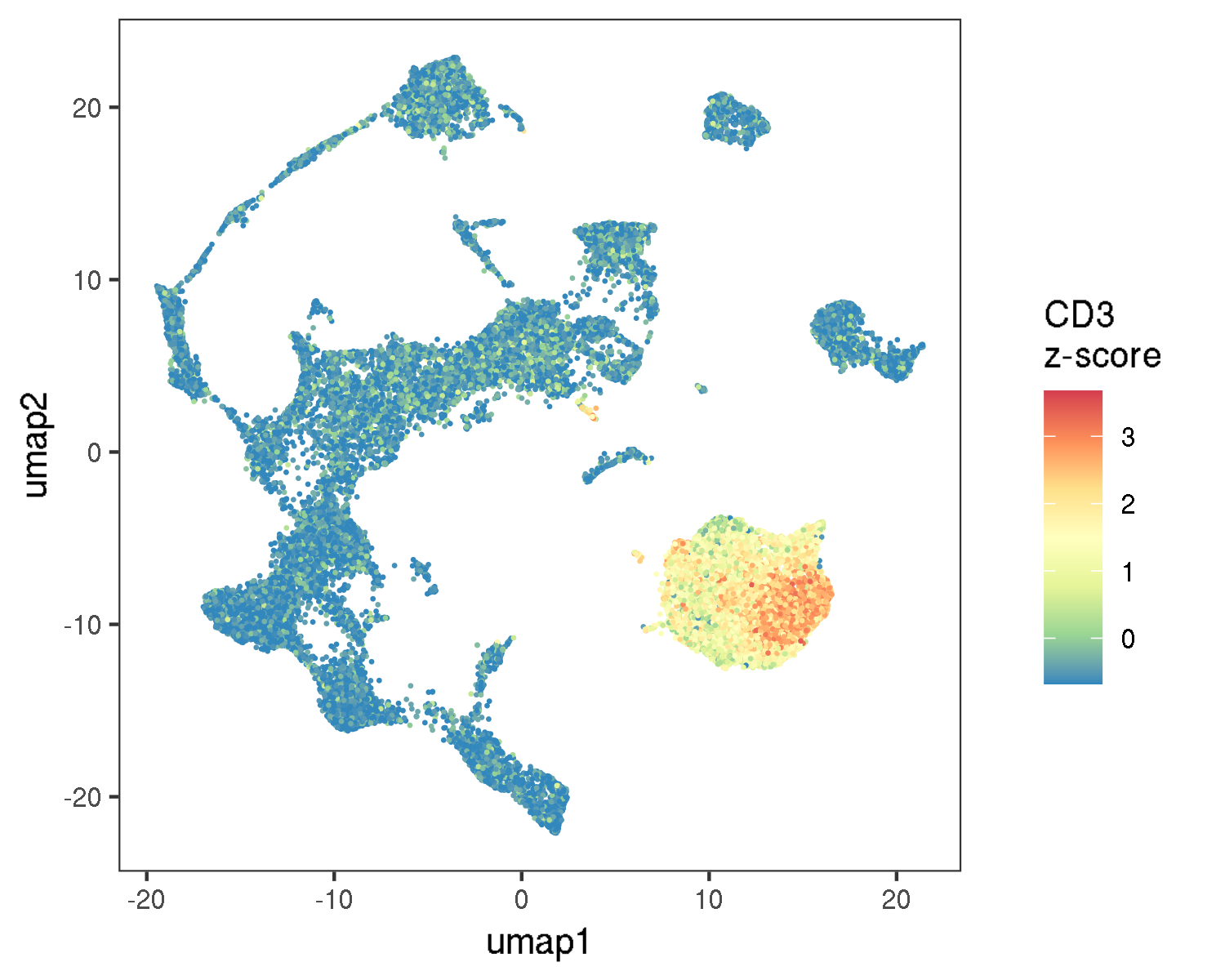

Supplement: Supplementary file 8 — Supplementary Data 5 [file 41467_2024_49883_MOESM8_ESM.zip › BMMC_final_panel_all_markers/CD3.png]

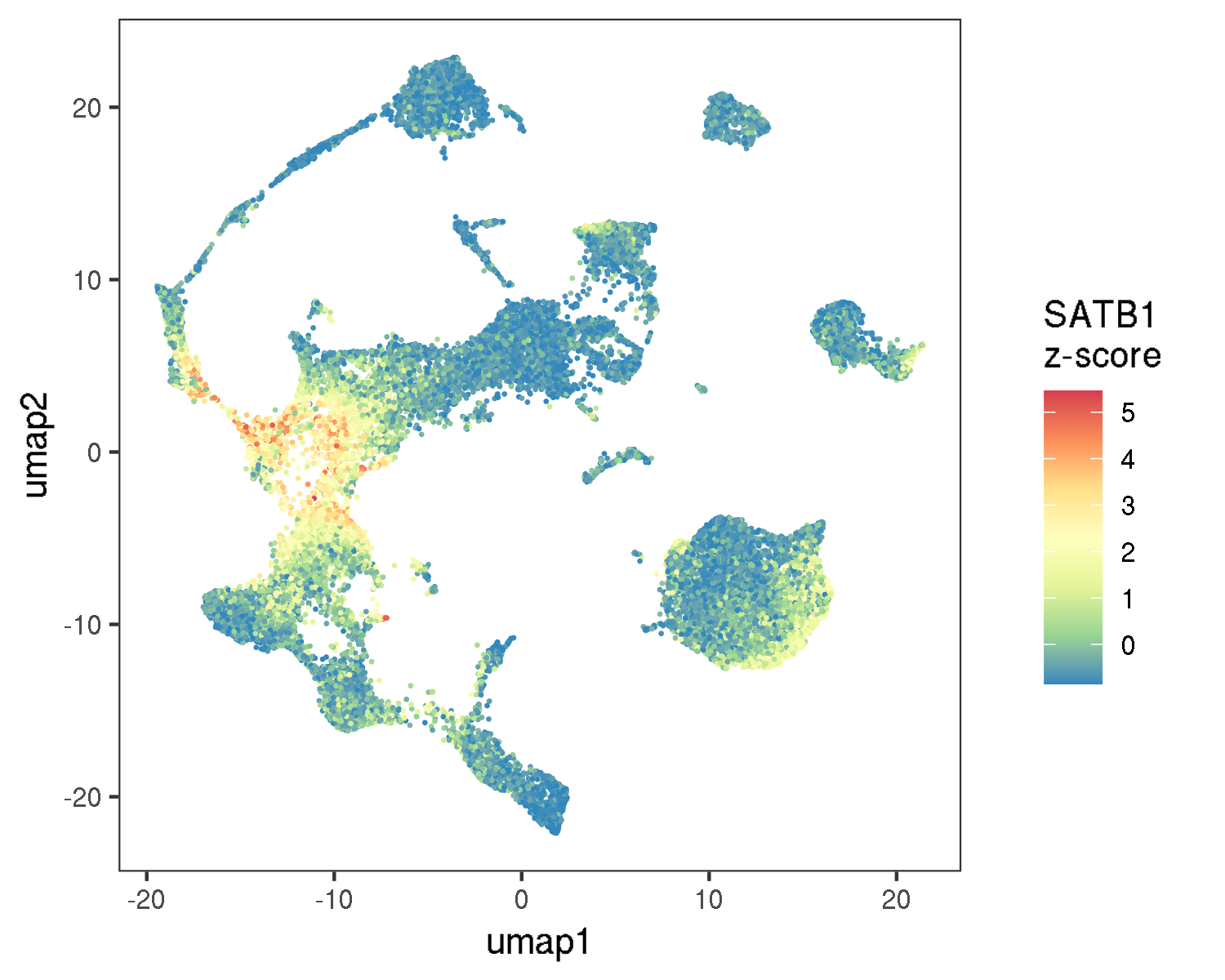

Supplement: Supplementary file 8 — Supplementary Data 5 [file 41467_2024_49883_MOESM8_ESM.zip › BMMC_final_panel_all_markers/SATB1.png]

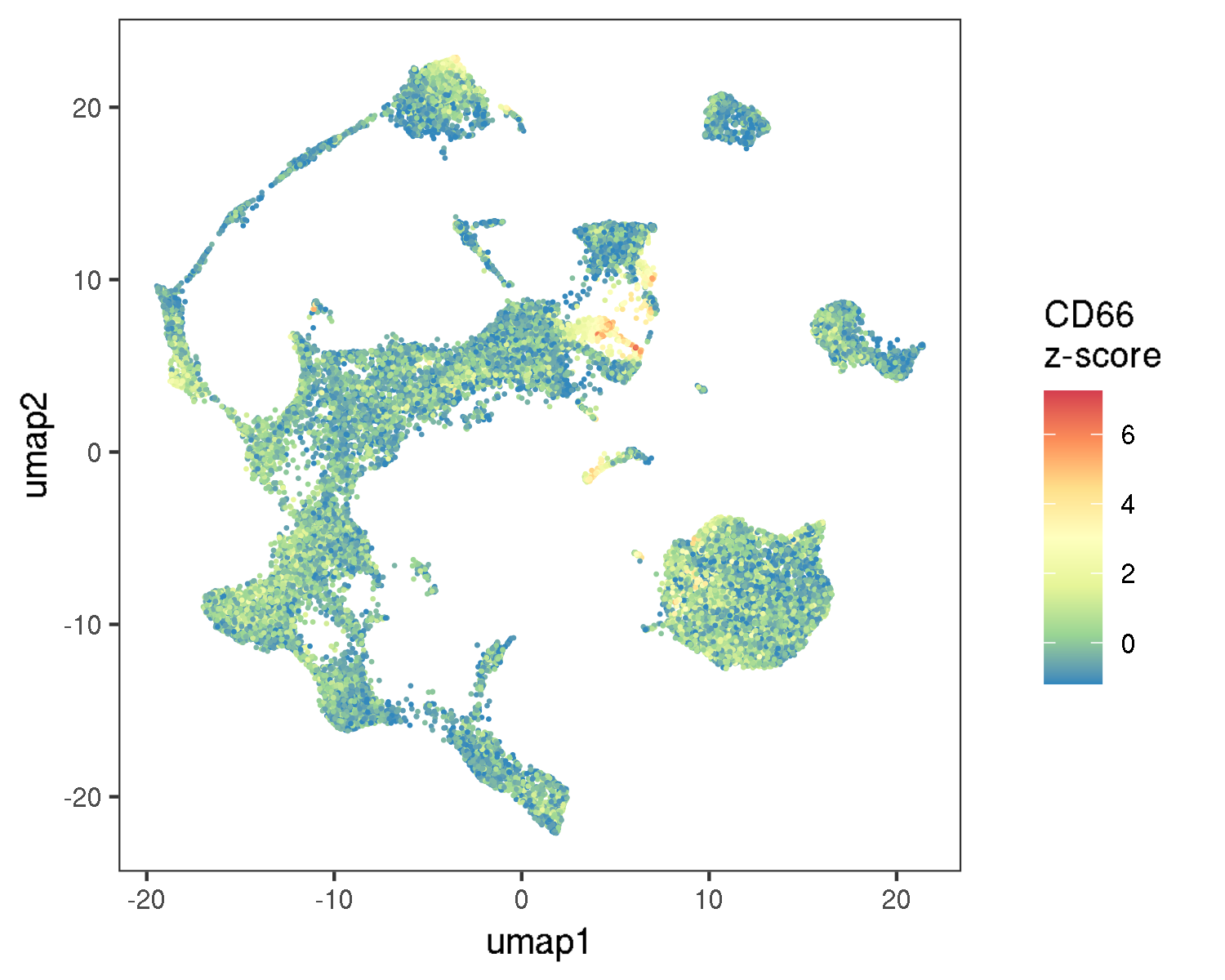

Supplement: Supplementary file 8 — Supplementary Data 5 [file 41467_2024_49883_MOESM8_ESM.zip › BMMC_final_panel_all_markers/CD66.png]

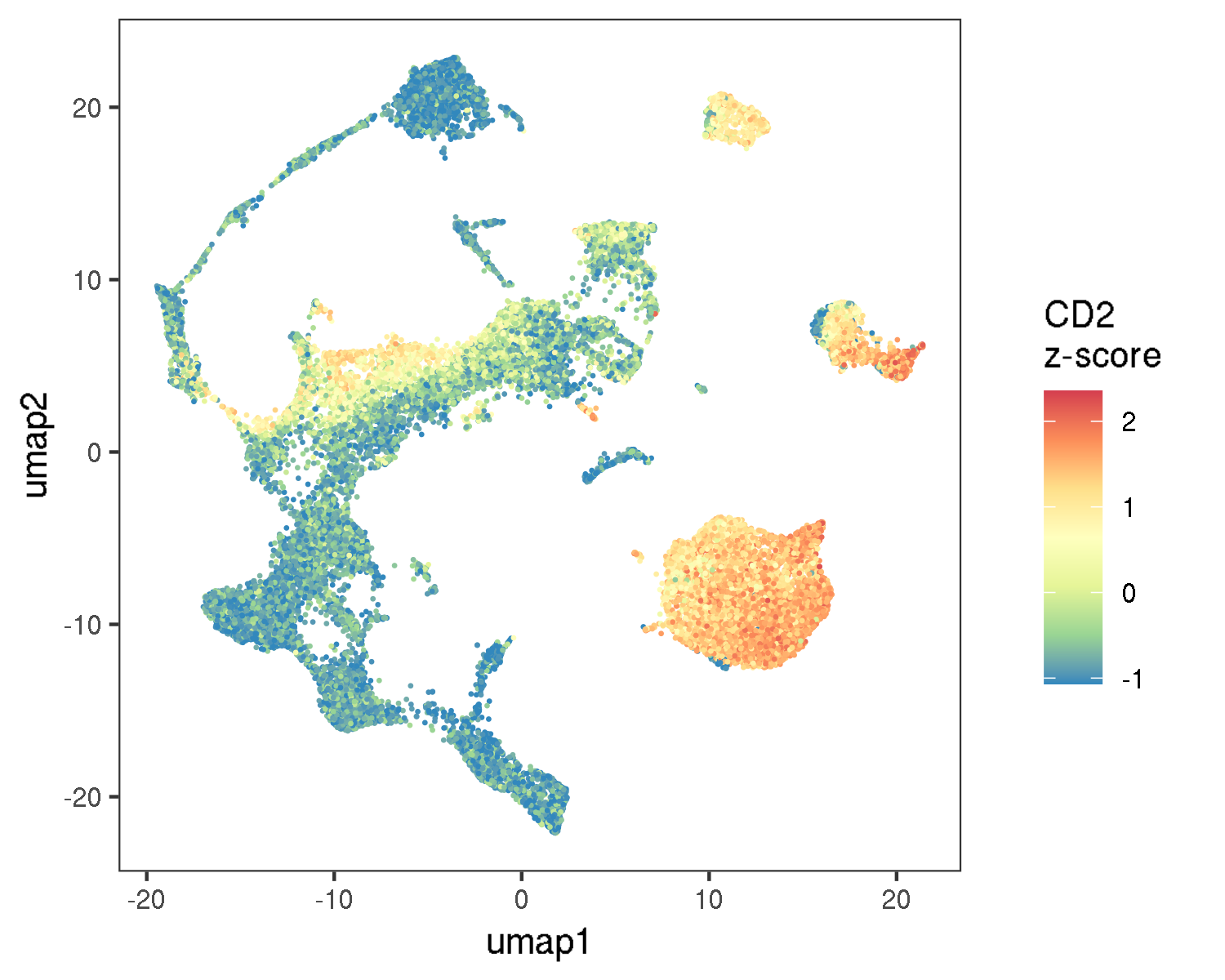

Supplement: Supplementary file 8 — Supplementary Data 5 [file 41467_2024_49883_MOESM8_ESM.zip › BMMC_final_panel_all_markers/CD2.png]

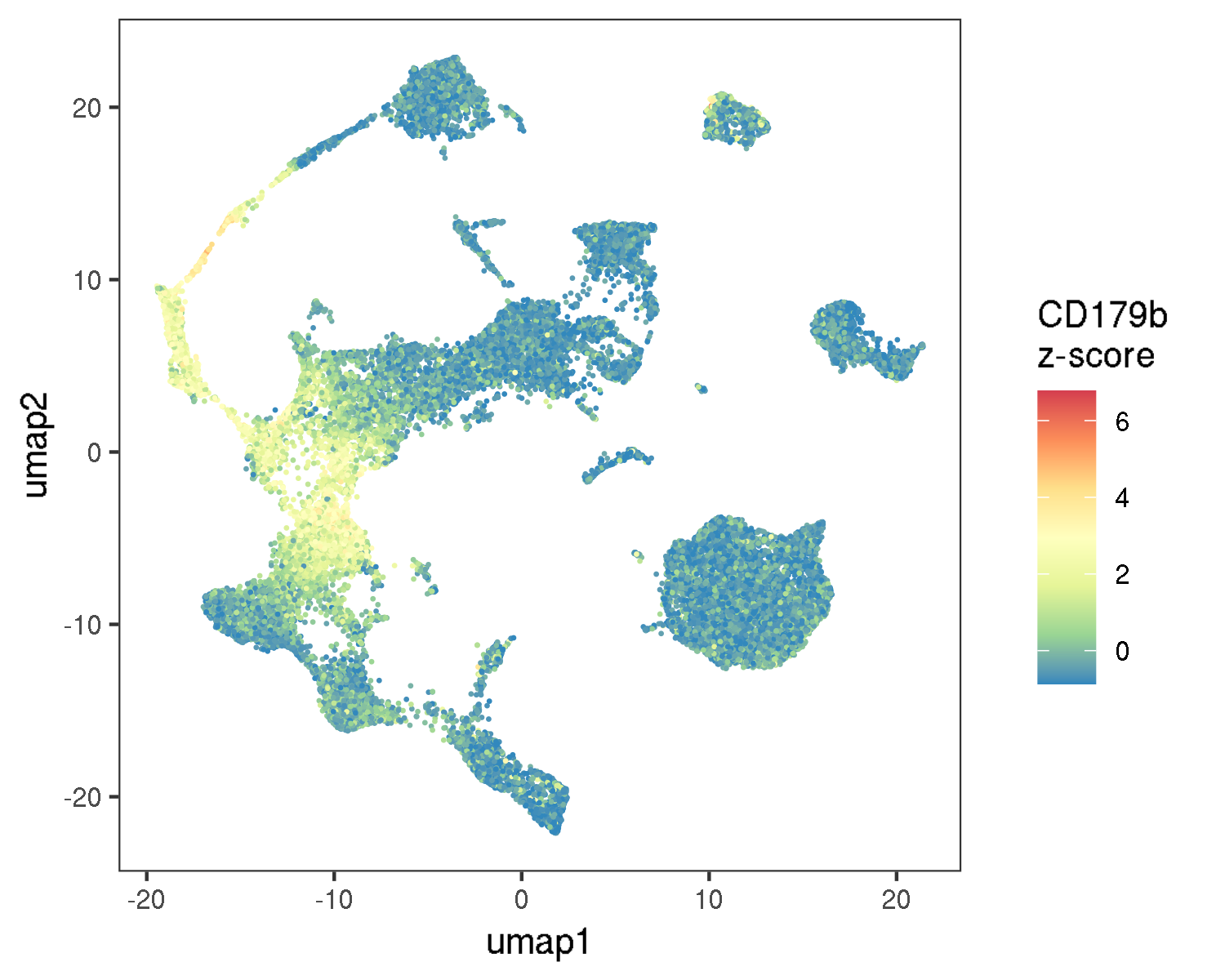

Supplement: Supplementary file 8 — Supplementary Data 5 [file 41467_2024_49883_MOESM8_ESM.zip › BMMC_final_panel_all_markers/CD179b.png]

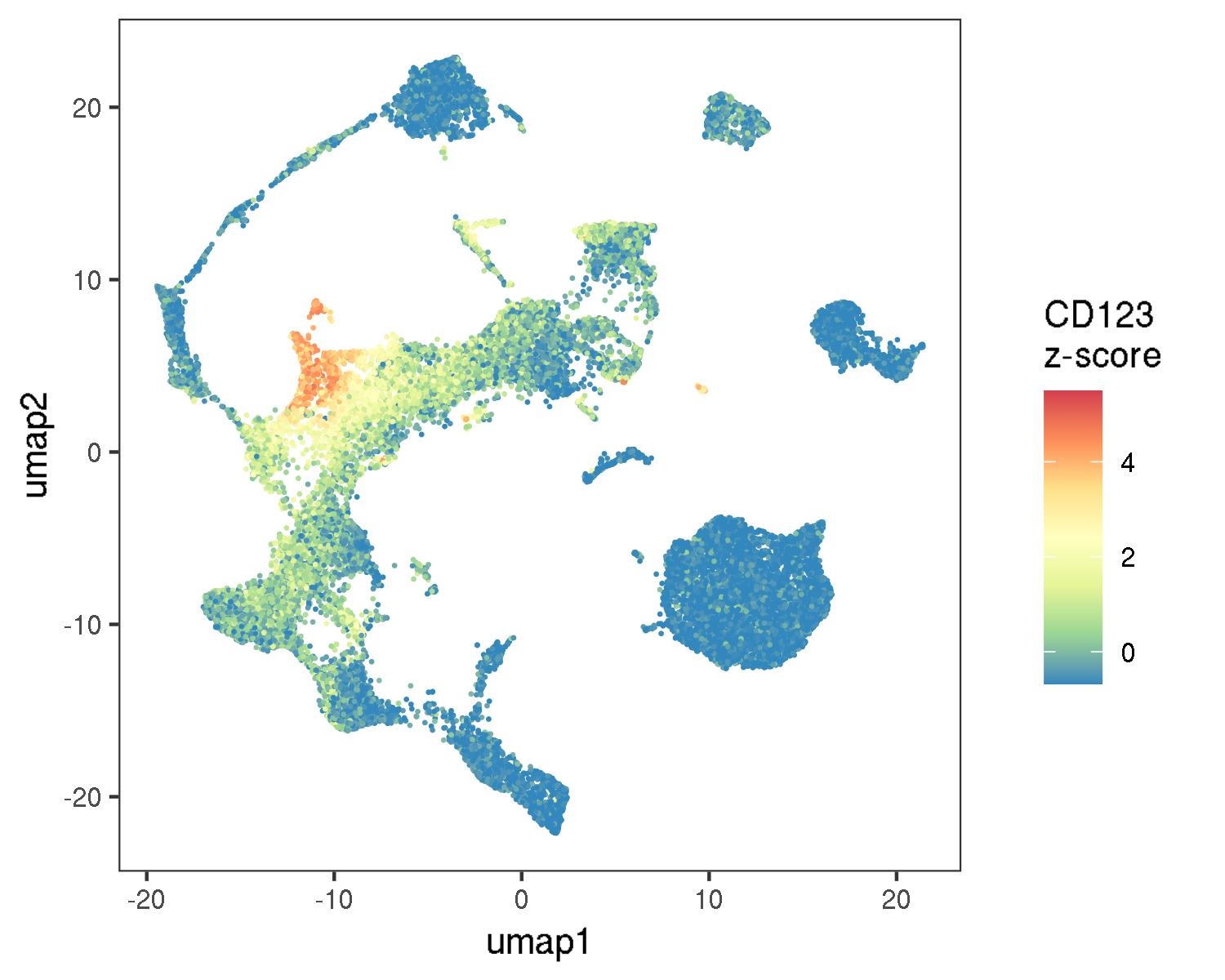

Supplement: Supplementary file 8 — Supplementary Data 5 [file 41467_2024_49883_MOESM8_ESM.zip › BMMC_final_panel_all_markers/CD123.png]

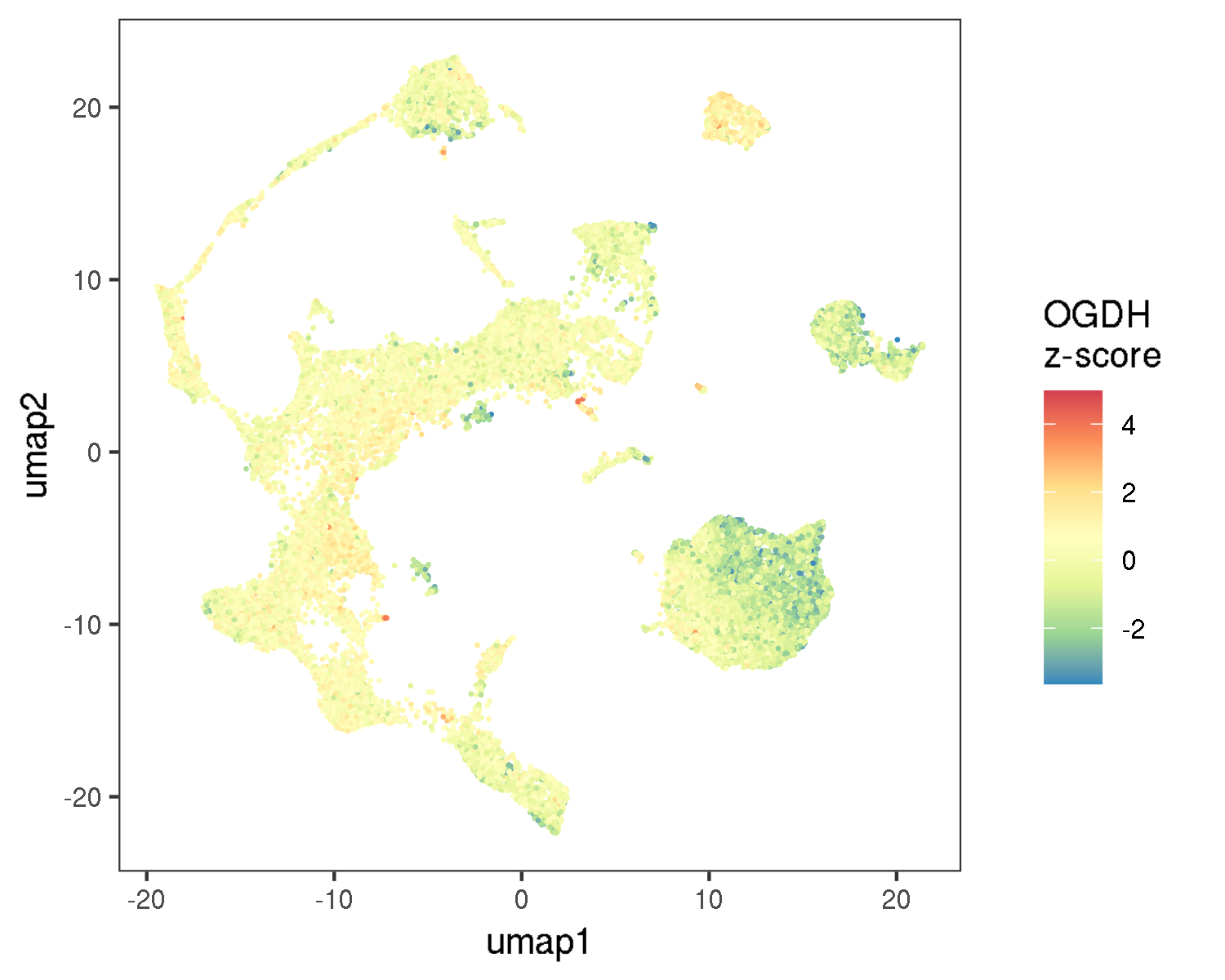

Supplement: Supplementary file 8 — Supplementary Data 5 [file 41467_2024_49883_MOESM8_ESM.zip › BMMC_final_panel_all_markers/OGDH.png]

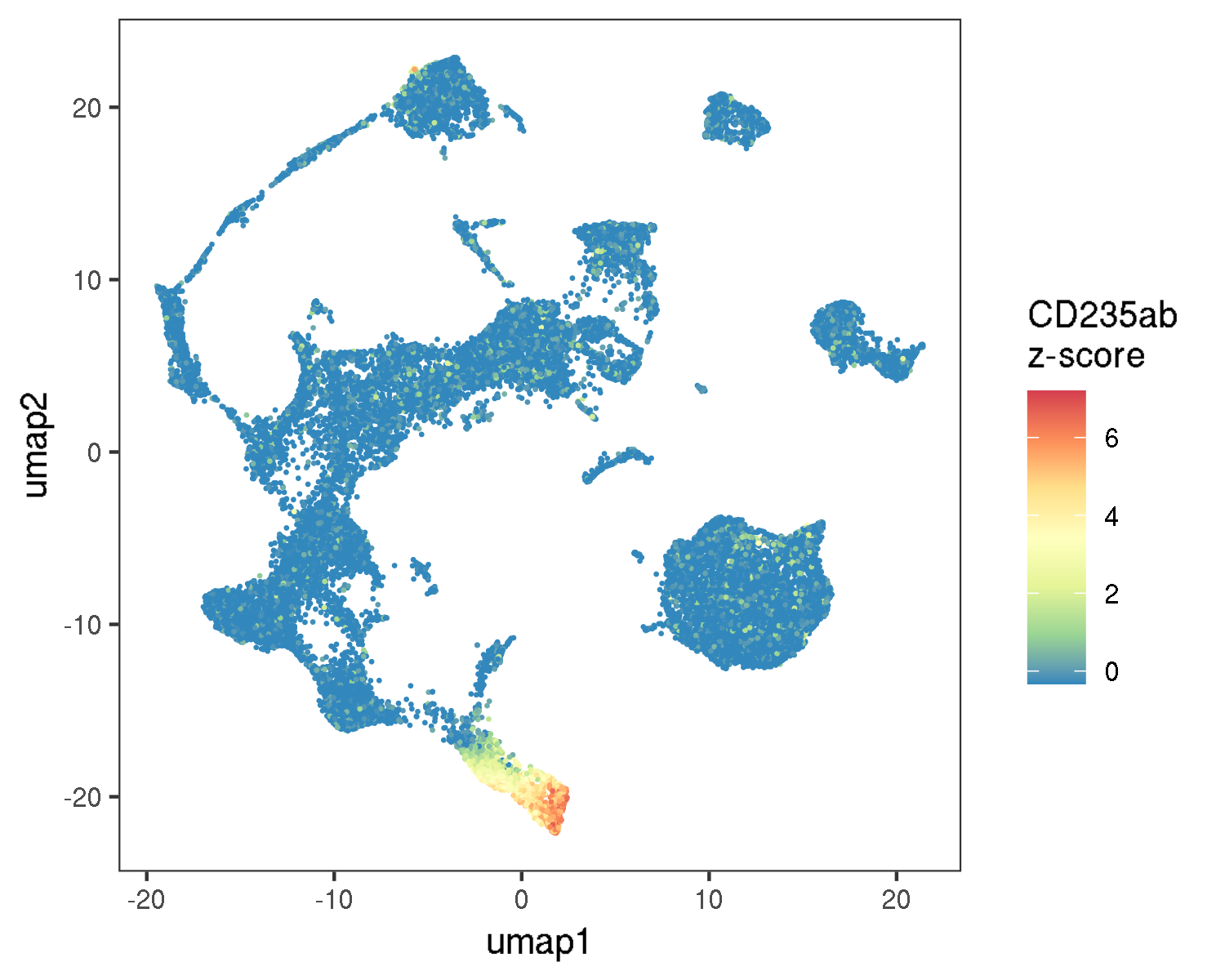

Supplement: Supplementary file 8 — Supplementary Data 5 [file 41467_2024_49883_MOESM8_ESM.zip › BMMC_final_panel_all_markers/CD235ab.png]

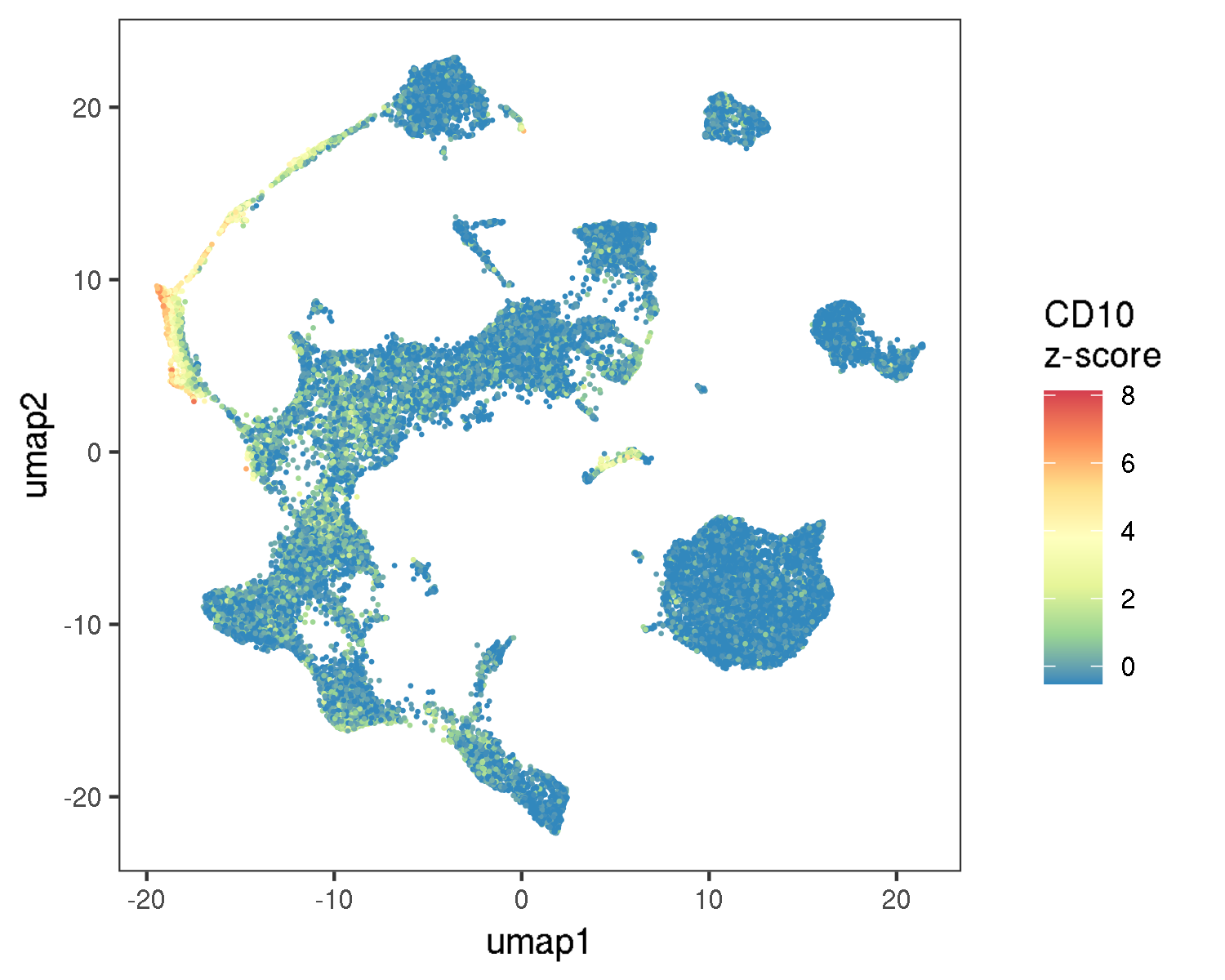

Supplement: Supplementary file 8 — Supplementary Data 5 [file 41467_2024_49883_MOESM8_ESM.zip › BMMC_final_panel_all_markers/CD10.png]

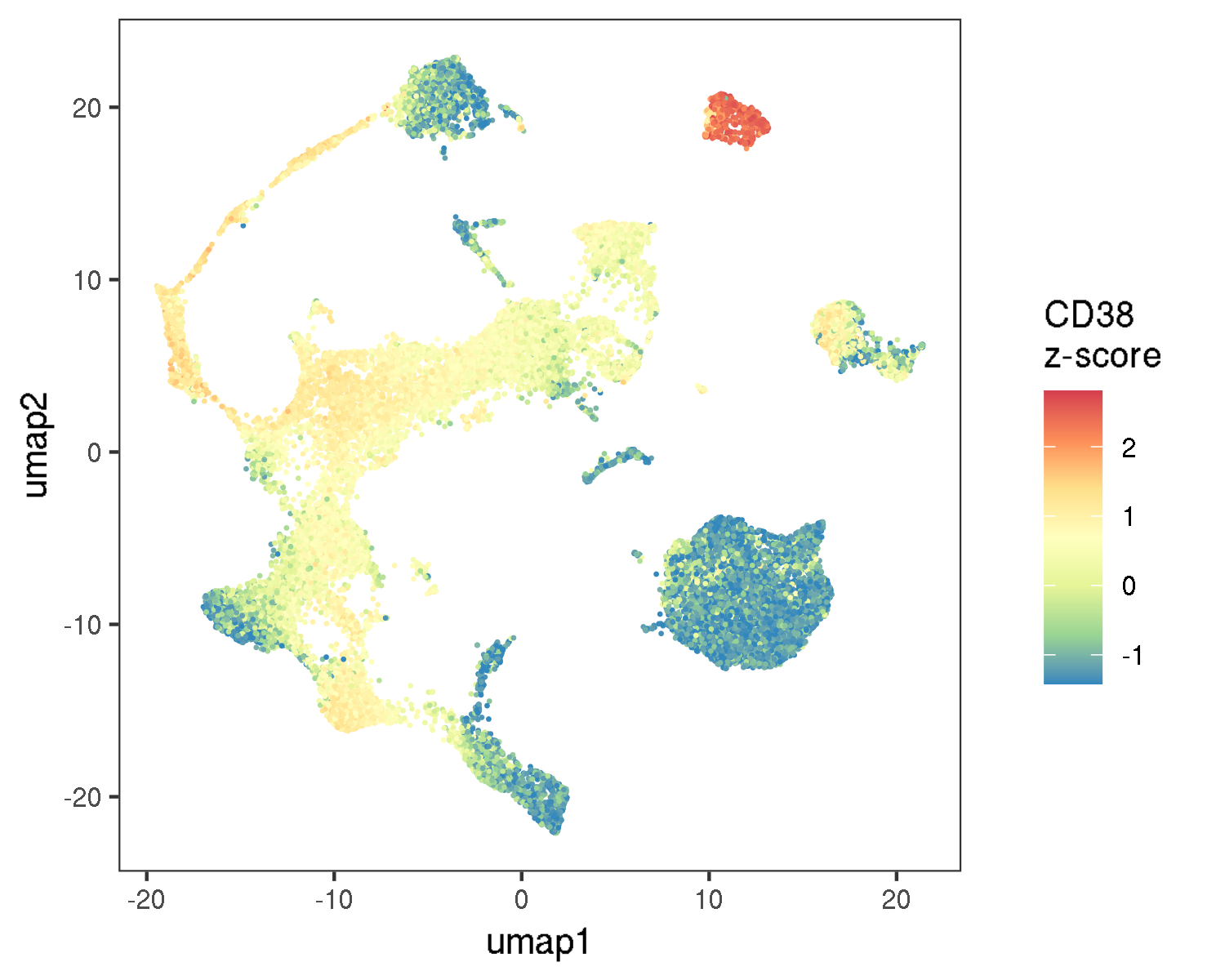

Supplement: Supplementary file 8 — Supplementary Data 5 [file 41467_2024_49883_MOESM8_ESM.zip › BMMC_final_panel_all_markers/CD38.png]

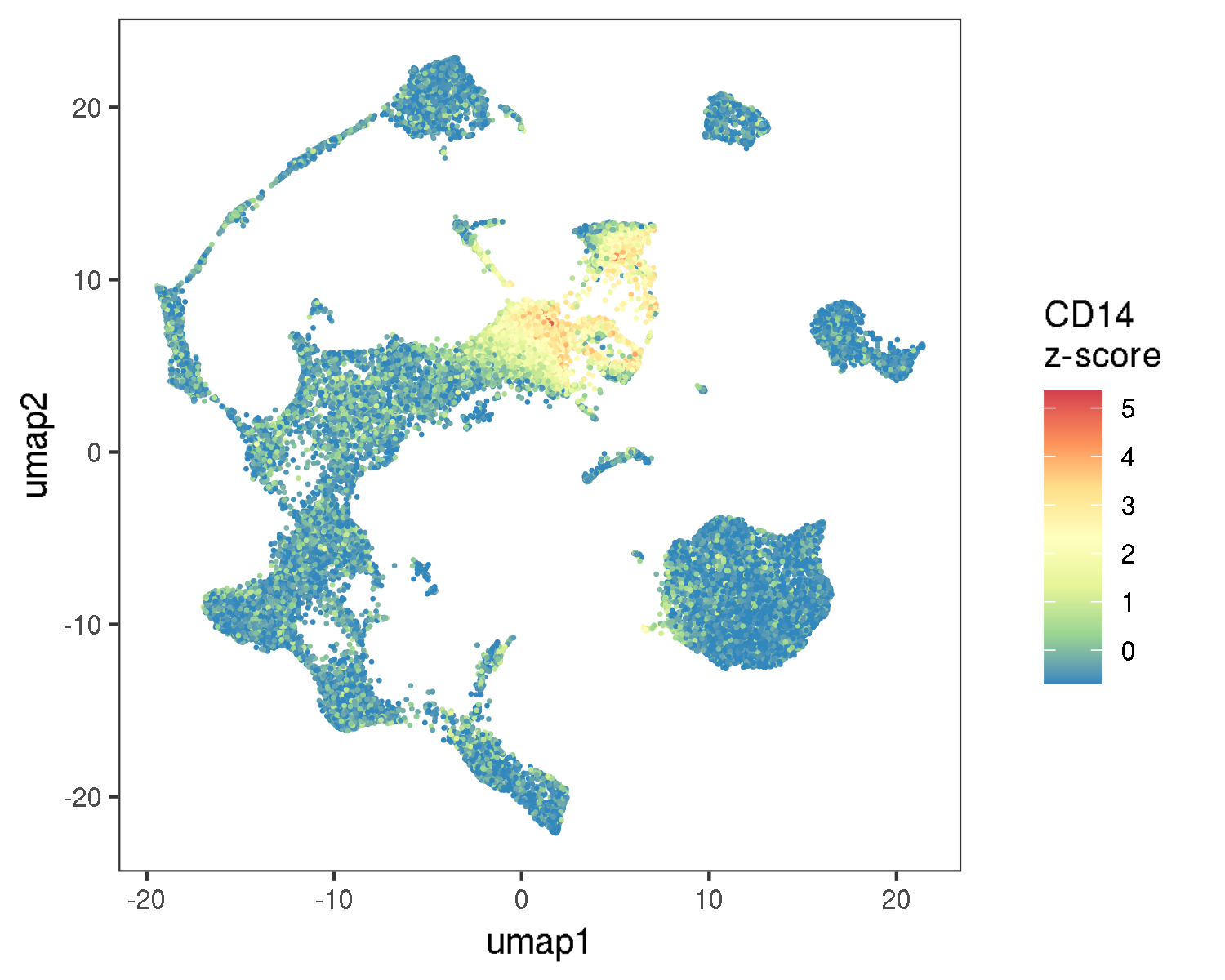

Supplement: Supplementary file 8 — Supplementary Data 5 [file 41467_2024_49883_MOESM8_ESM.zip › BMMC_final_panel_all_markers/CD14.png]

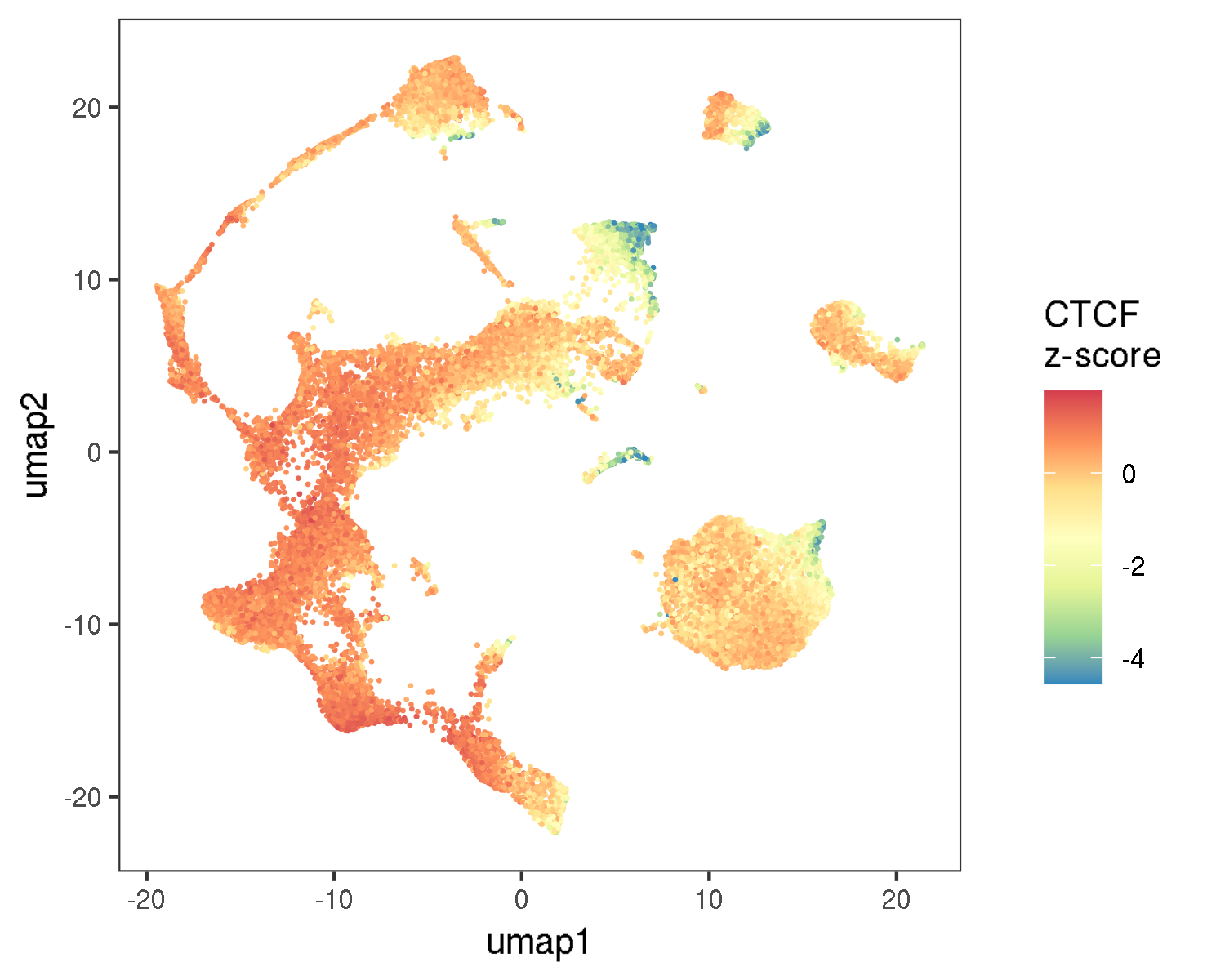

Supplement: Supplementary file 8 — Supplementary Data 5 [file 41467_2024_49883_MOESM8_ESM.zip › BMMC_final_panel_all_markers/CTCF.png]

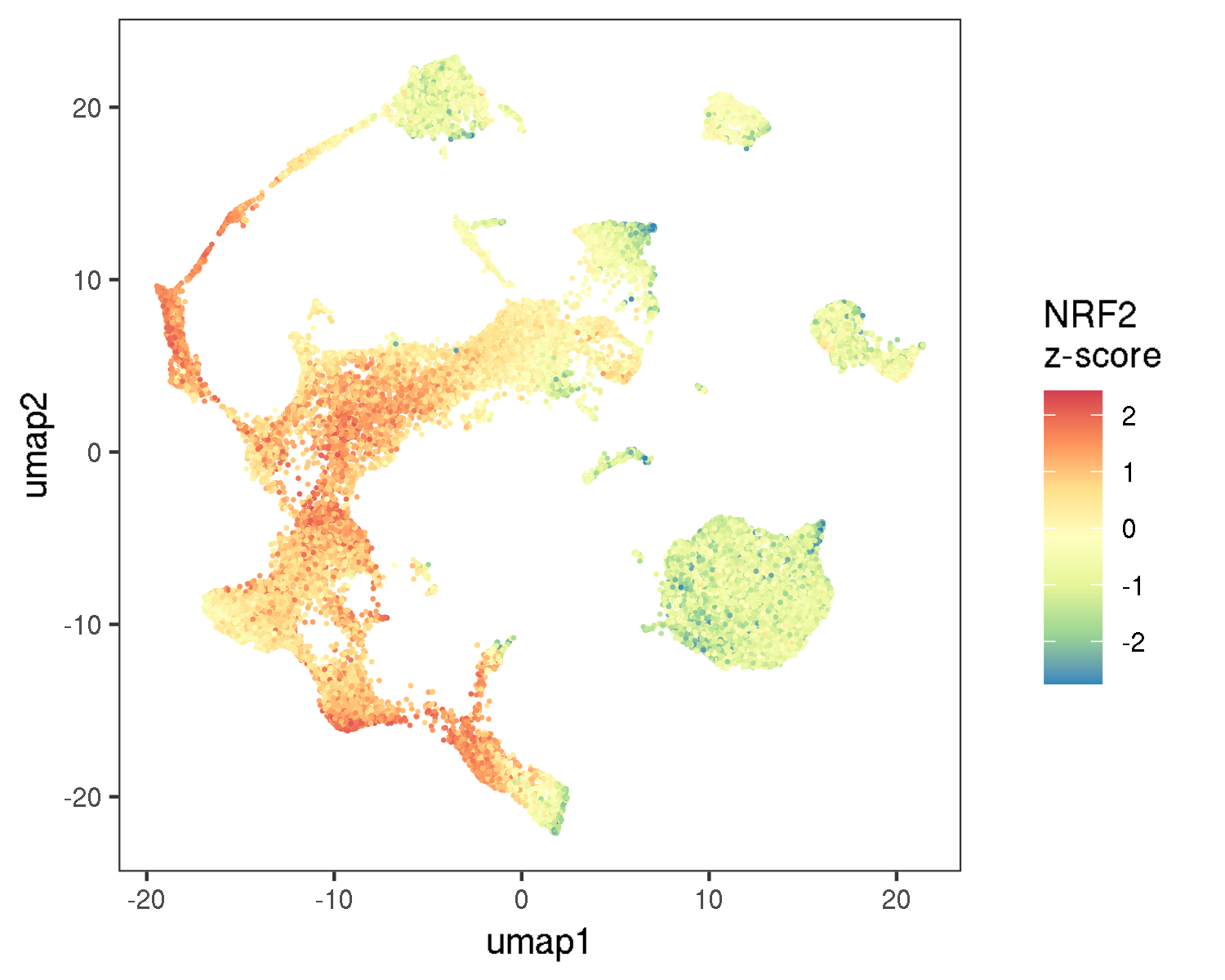

Supplement: Supplementary file 8 — Supplementary Data 5 [file 41467_2024_49883_MOESM8_ESM.zip › BMMC_final_panel_all_markers/NRF2.png]

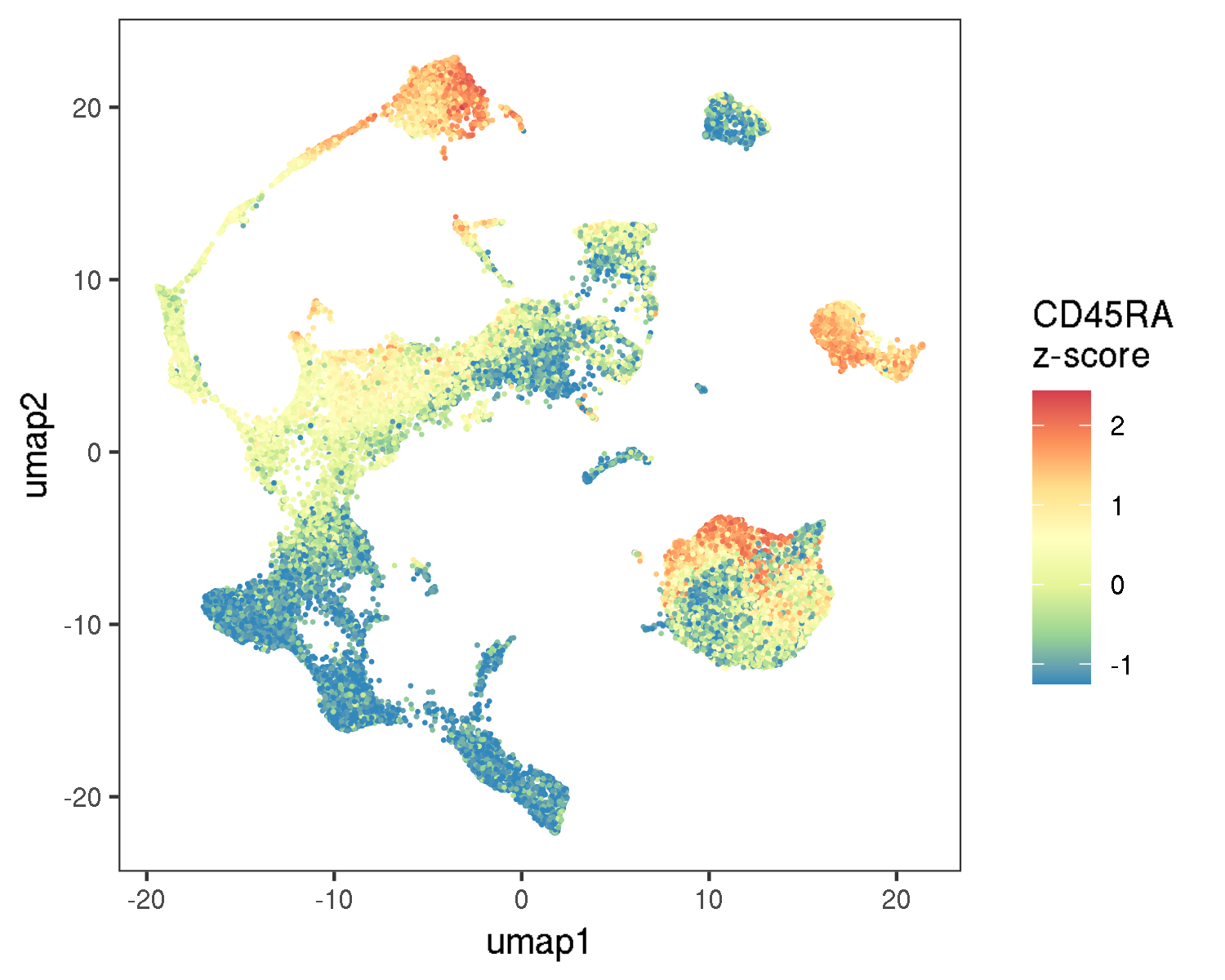

Supplement: Supplementary file 8 — Supplementary Data 5 [file 41467_2024_49883_MOESM8_ESM.zip › BMMC_final_panel_all_markers/CD45RA.png]

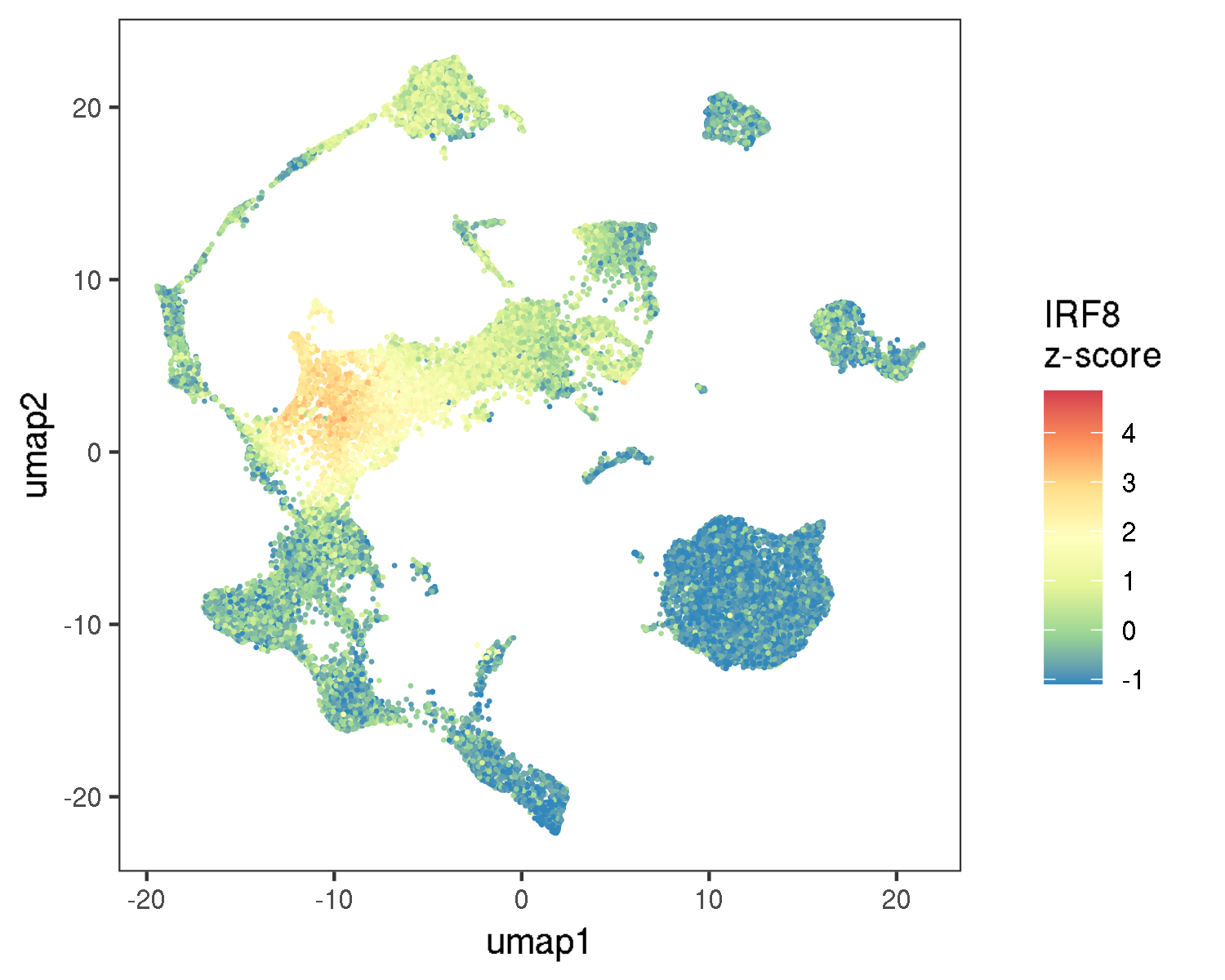

Supplement: Supplementary file 8 — Supplementary Data 5 [file 41467_2024_49883_MOESM8_ESM.zip › BMMC_final_panel_all_markers/IRF8.png]

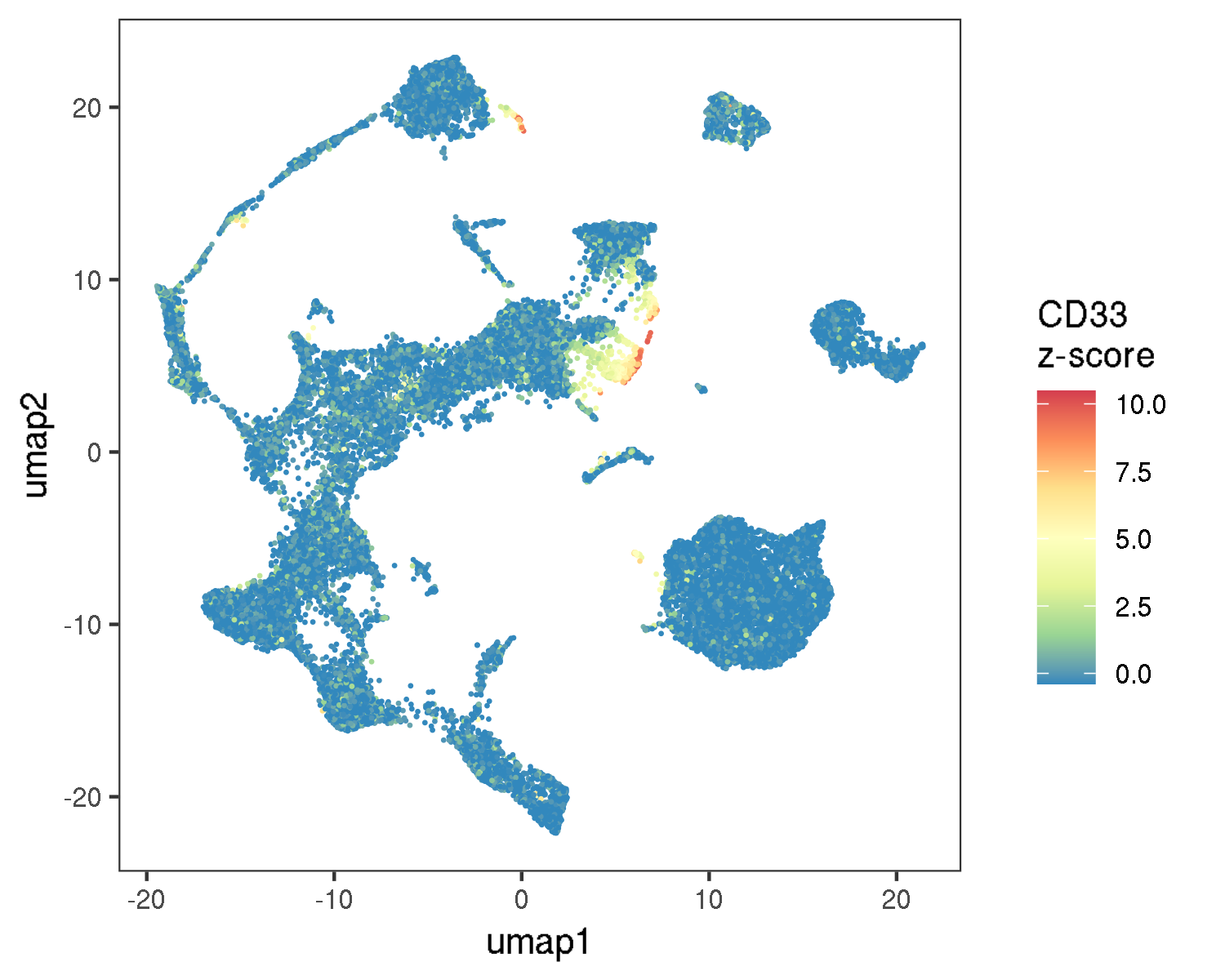

Supplement: Supplementary file 8 — Supplementary Data 5 [file 41467_2024_49883_MOESM8_ESM.zip › BMMC_final_panel_all_markers/CD33.png]

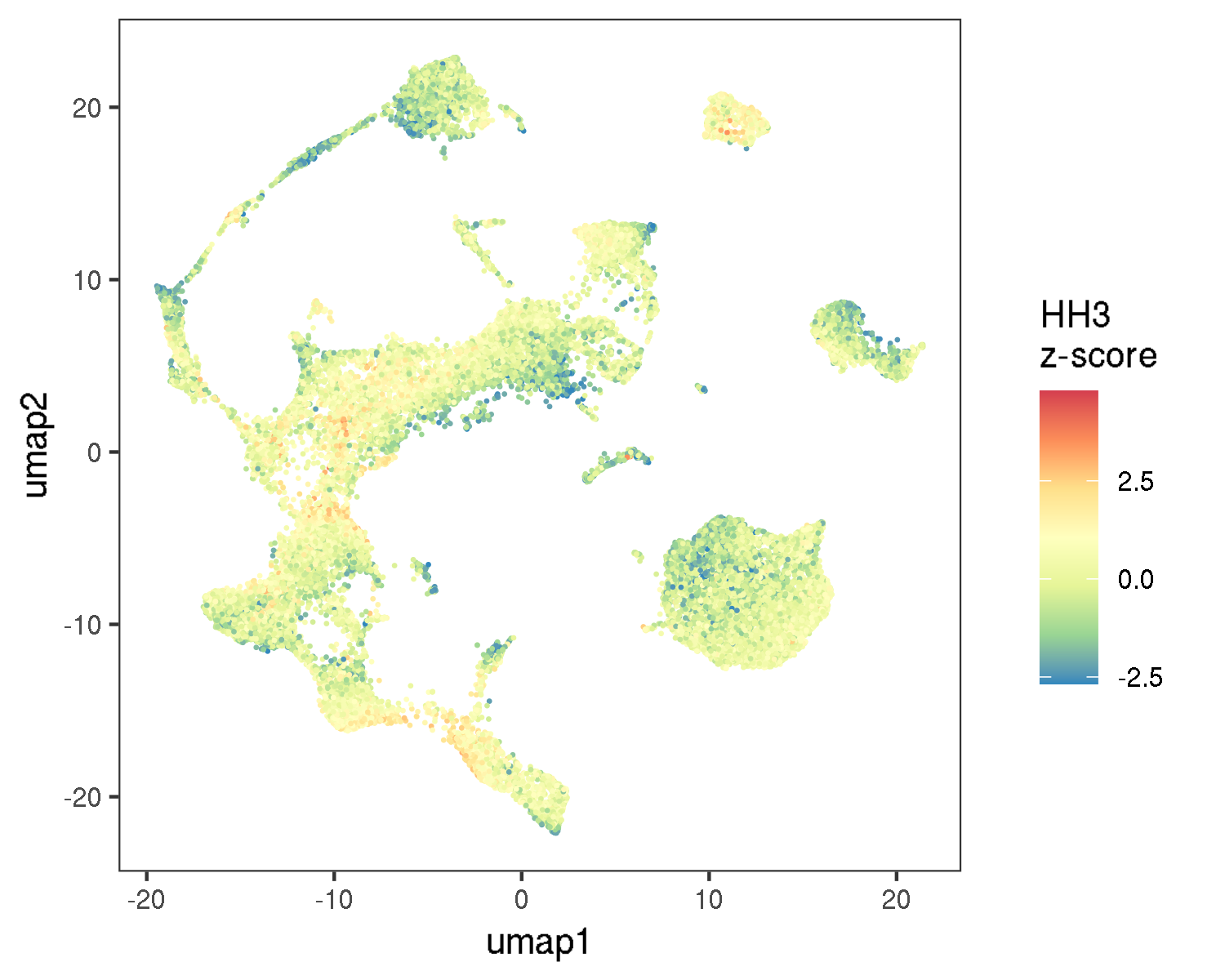

Supplement: Supplementary file 8 — Supplementary Data 5 [file 41467_2024_49883_MOESM8_ESM.zip › BMMC_final_panel_all_markers/HH3.png]

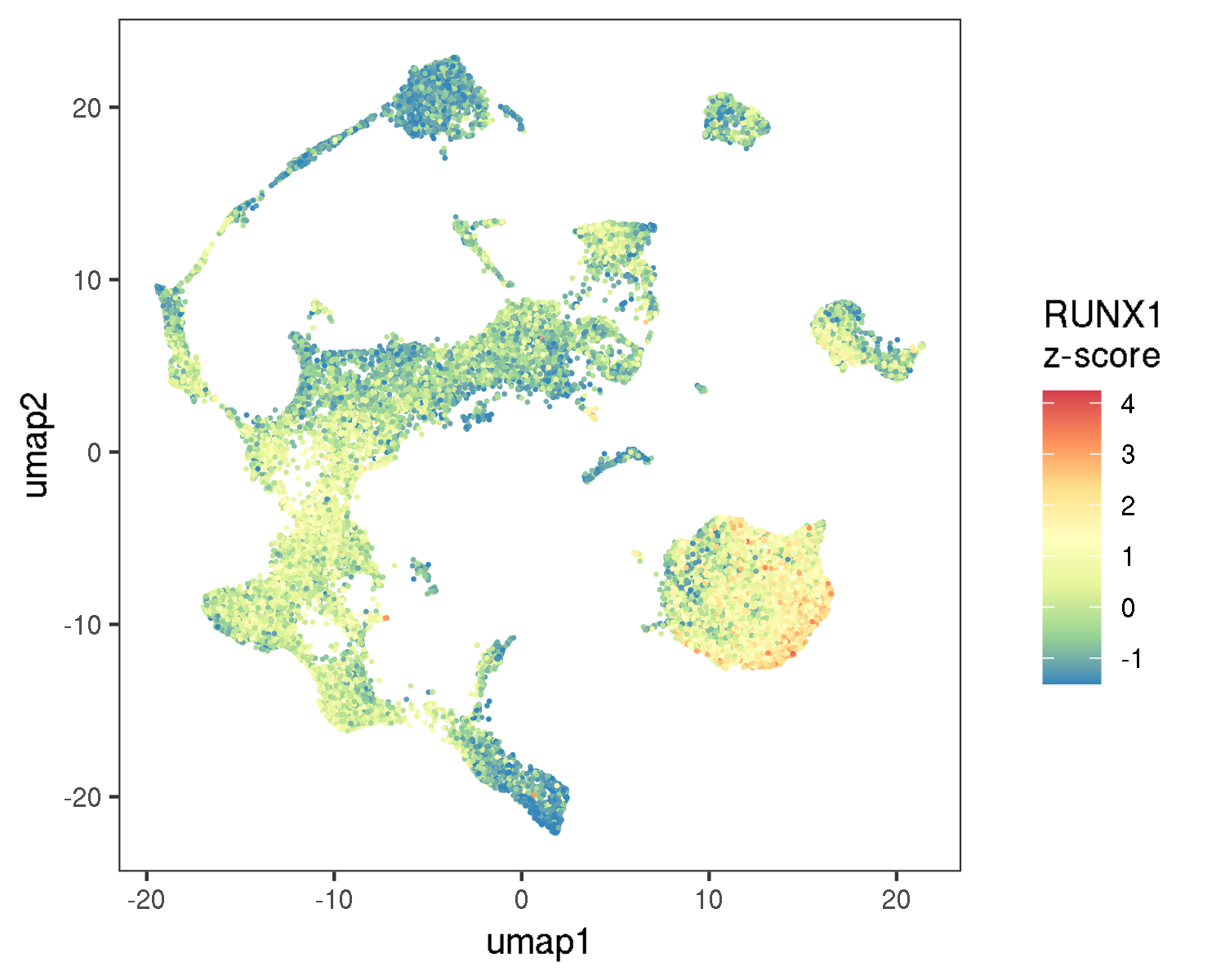

Supplement: Supplementary file 8 — Supplementary Data 5 [file 41467_2024_49883_MOESM8_ESM.zip › BMMC_final_panel_all_markers/RUNX1.png]
