# Supplementary figures and images for: Terminal deoxynucleotidyl transferase and CD84 identify human multi-potent lymphoid progenitors (part 2 of 2)
Source: Nat Commun. 2024 Jul 13;15:5910. doi: 10.1038/s41467-024-49883-w (PMC11246490; doi:10.1038/s41467-024-49883-w)

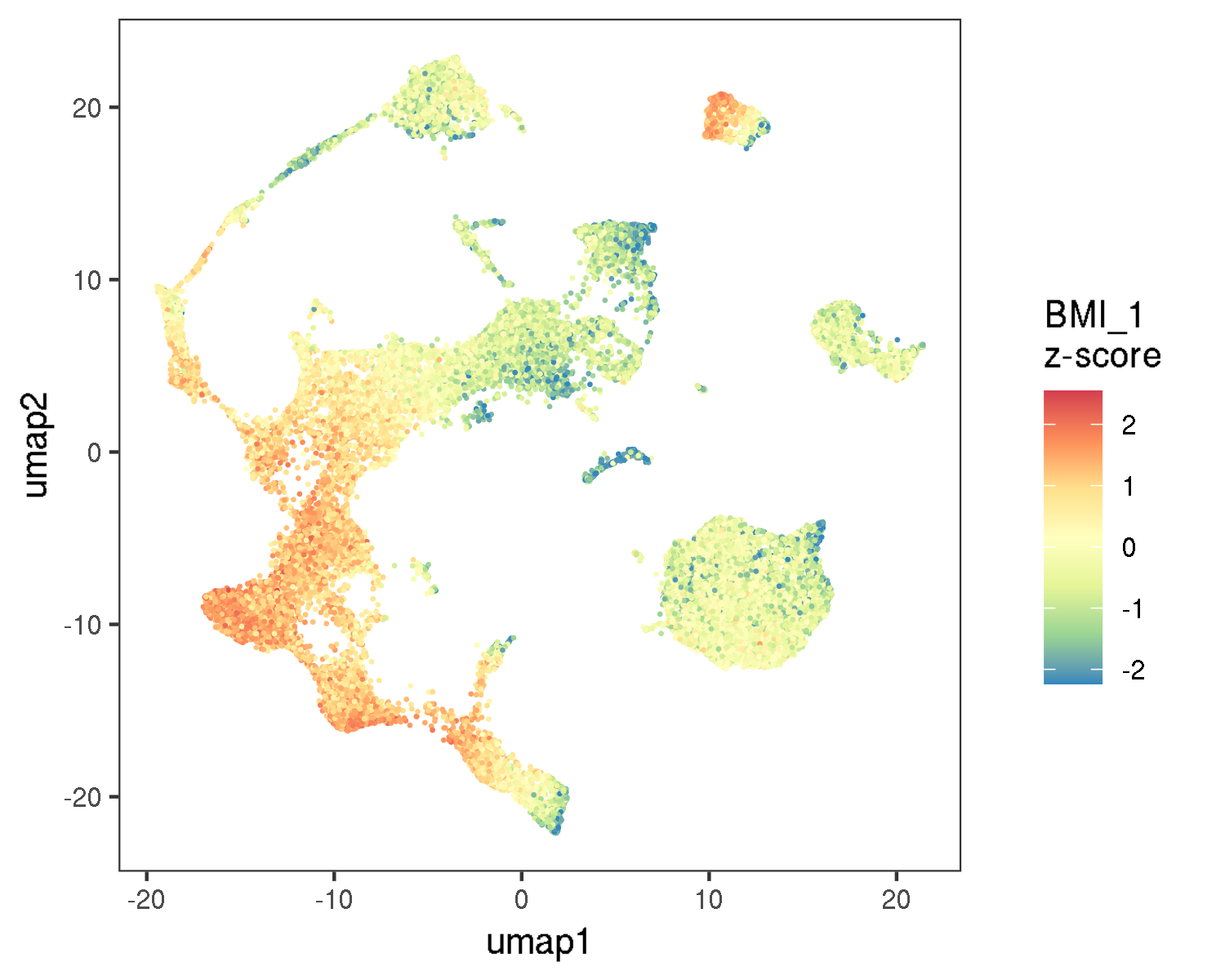

Supplement: Supplementary file 8 — Supplementary Data 5 [file 41467_2024_49883_MOESM8_ESM.zip › BMMC_final_panel_all_markers/BMI_1.png]

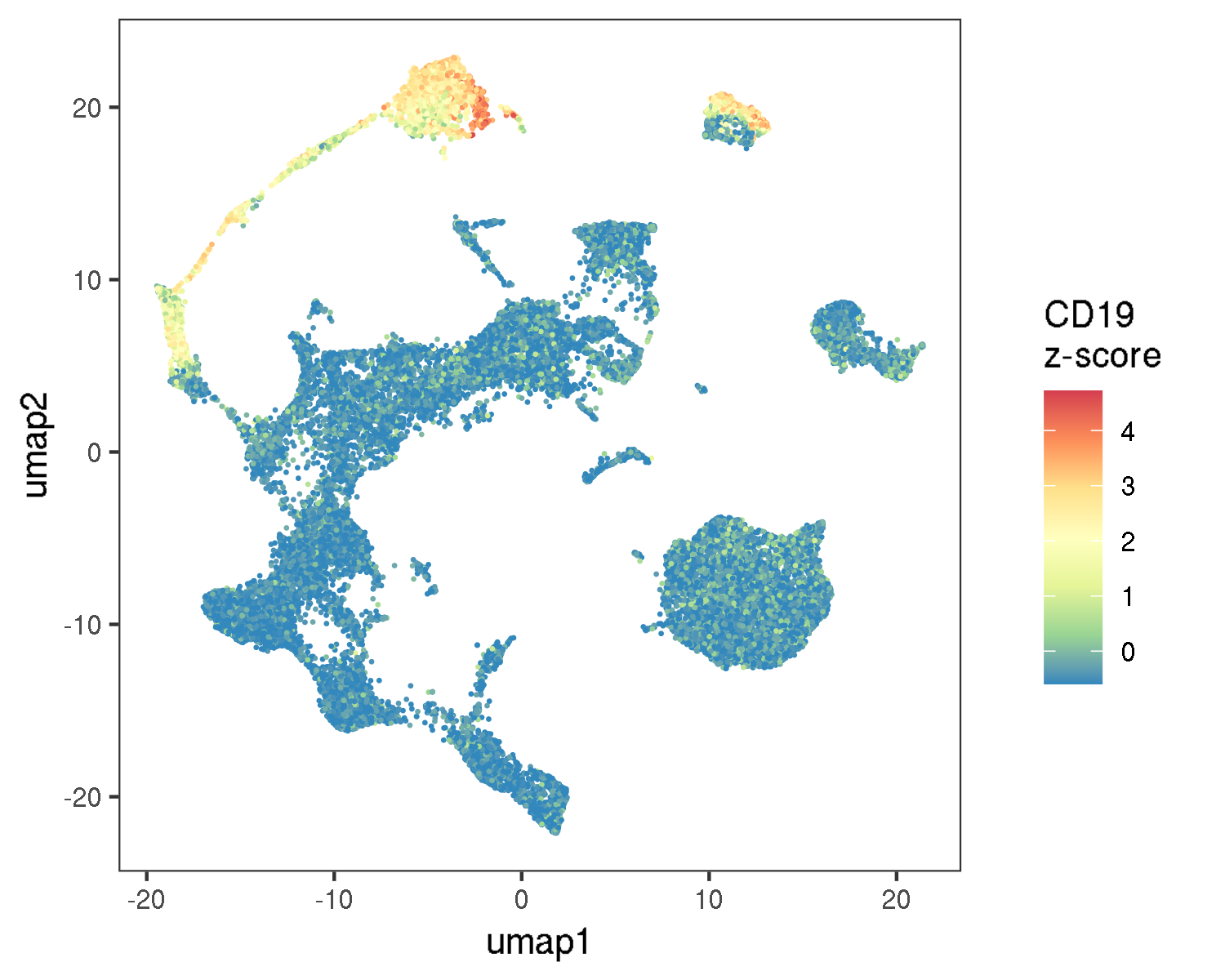

Supplement: Supplementary file 8 — Supplementary Data 5 [file 41467_2024_49883_MOESM8_ESM.zip › BMMC_final_panel_all_markers/CD19.png]

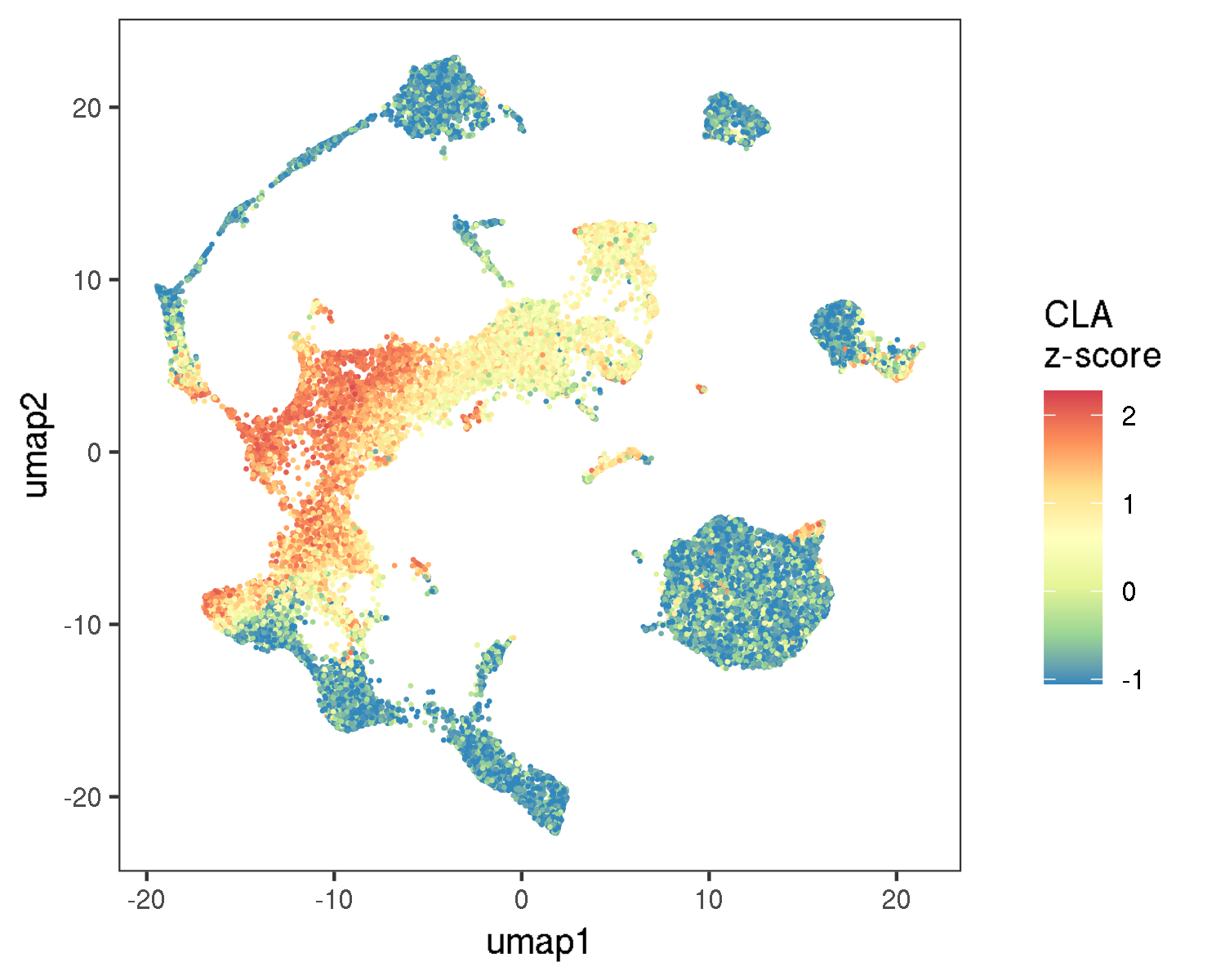

Supplement: Supplementary file 8 — Supplementary Data 5 [file 41467_2024_49883_MOESM8_ESM.zip › BMMC_final_panel_all_markers/CLA.png]

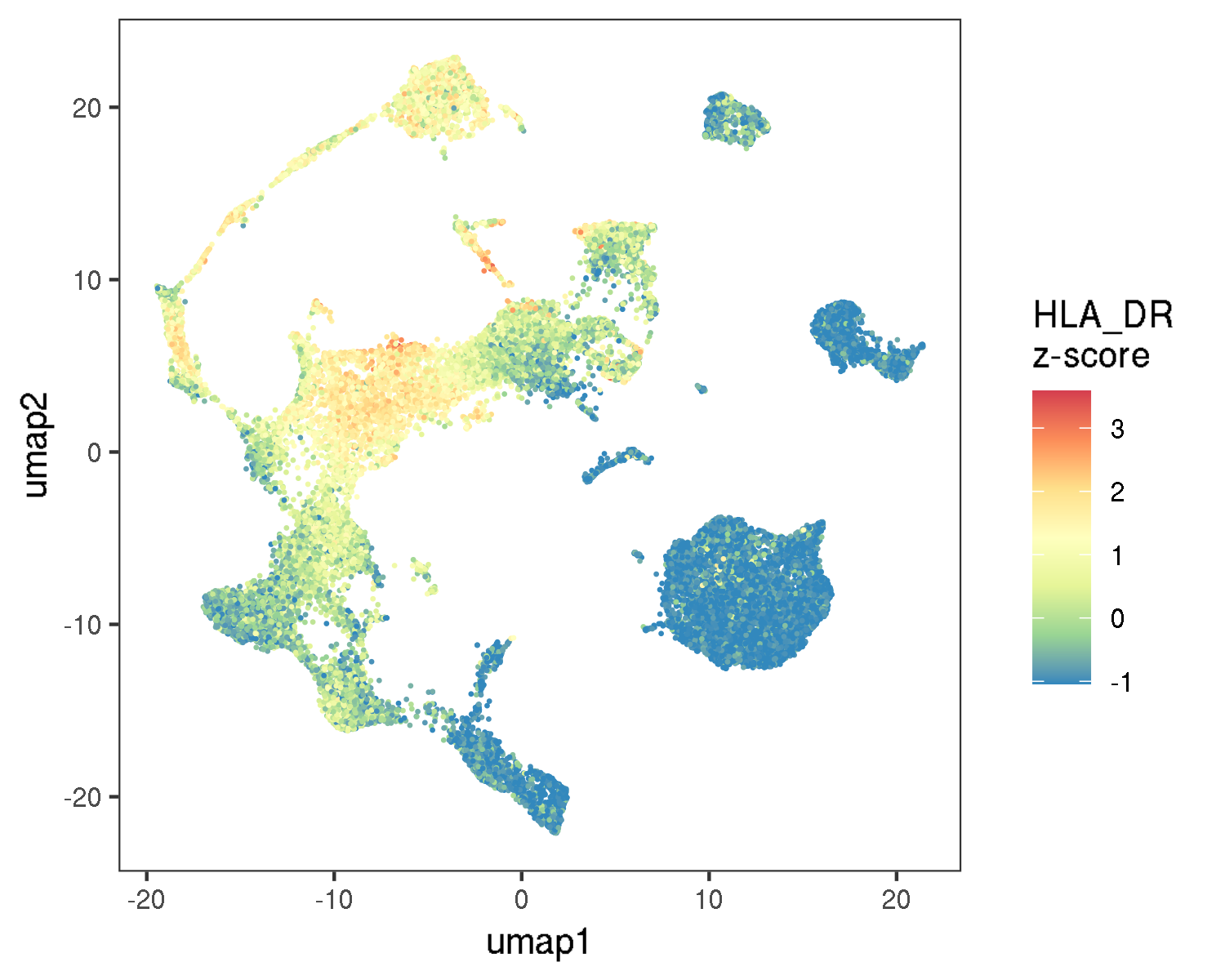

Supplement: Supplementary file 8 — Supplementary Data 5 [file 41467_2024_49883_MOESM8_ESM.zip › BMMC_final_panel_all_markers/HLA_DR.png]

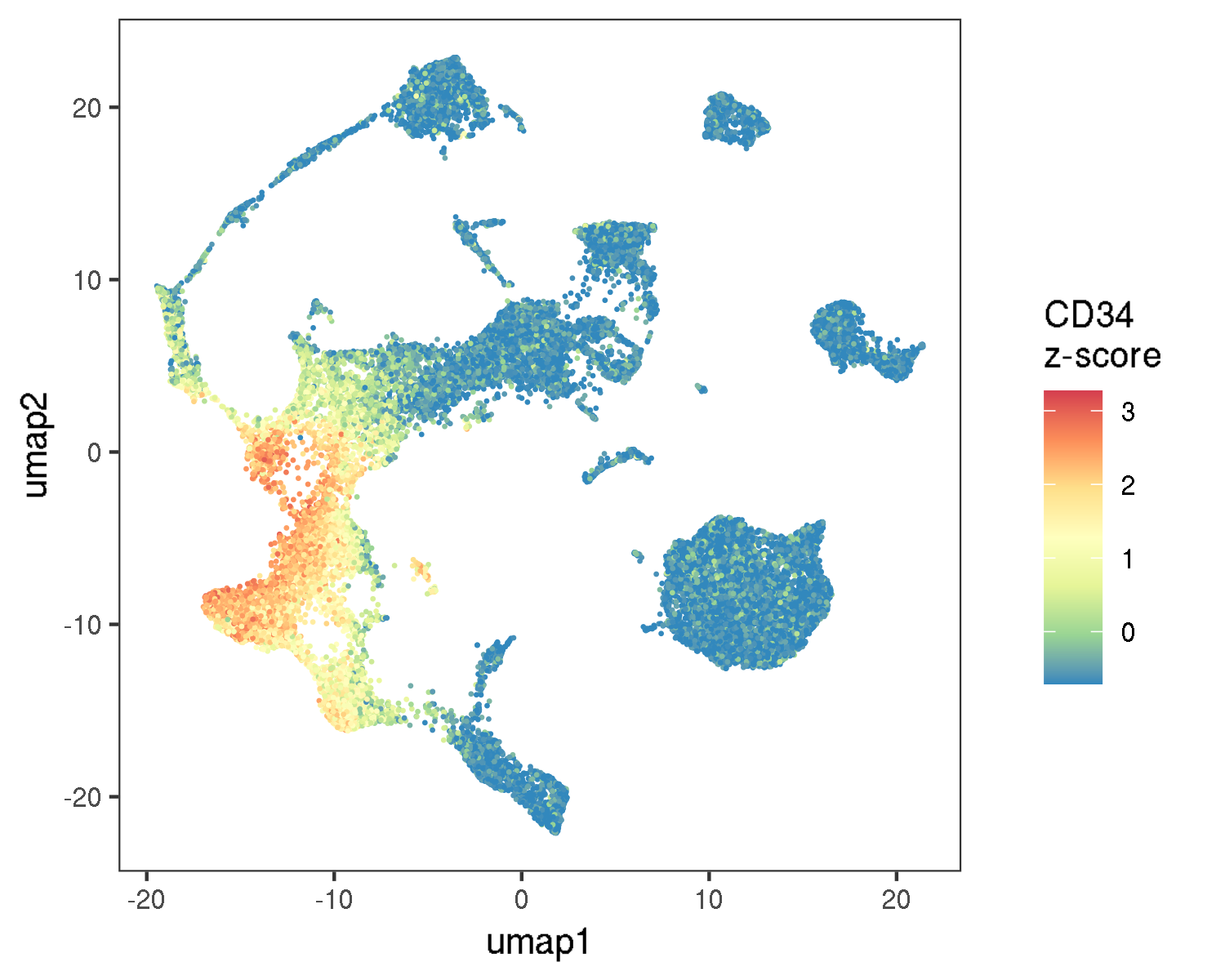

Supplement: Supplementary file 8 — Supplementary Data 5 [file 41467_2024_49883_MOESM8_ESM.zip › BMMC_final_panel_all_markers/CD34.png]

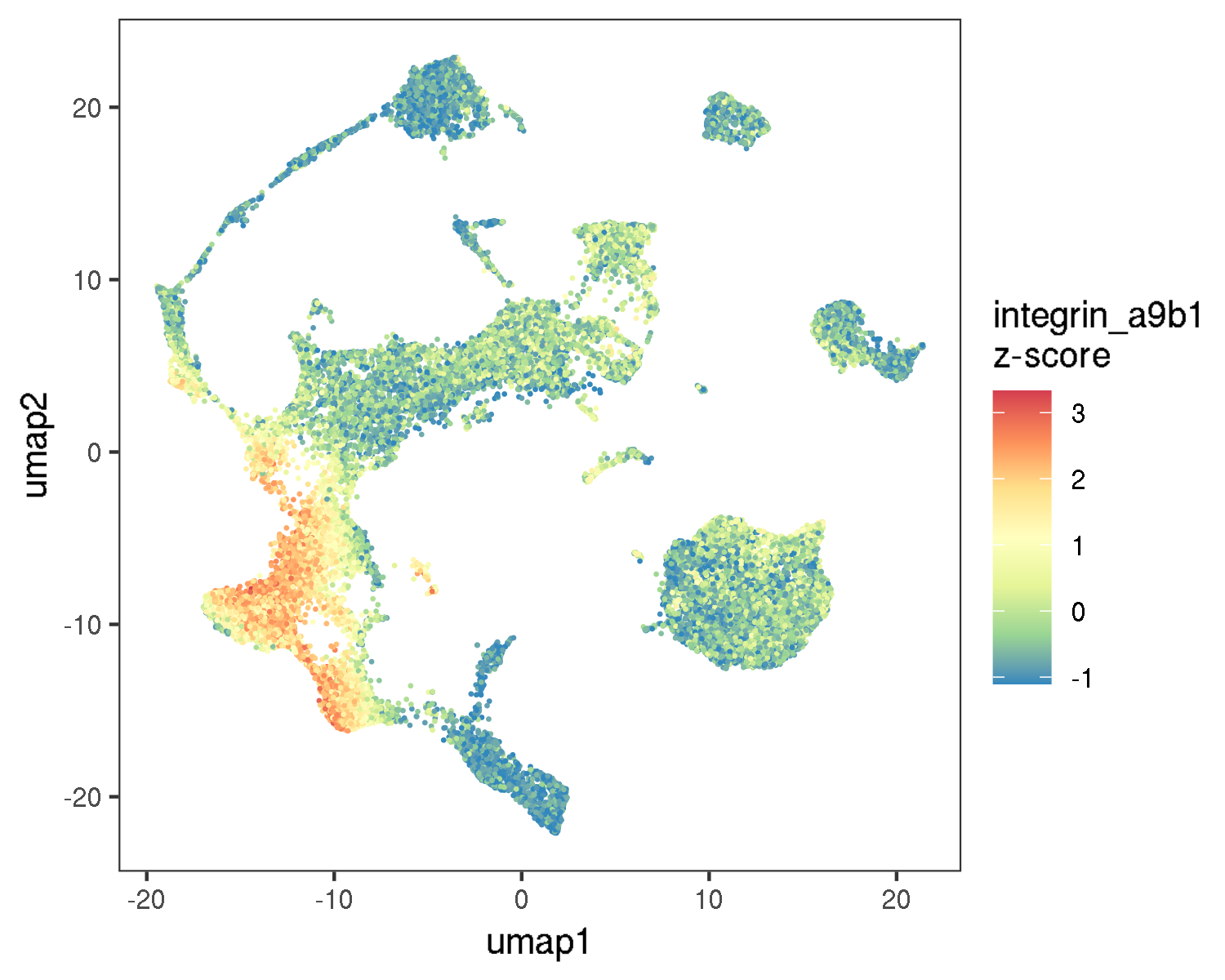

Supplement: Supplementary file 8 — Supplementary Data 5 [file 41467_2024_49883_MOESM8_ESM.zip › BMMC_final_panel_all_markers/integrin_a9b1.png]

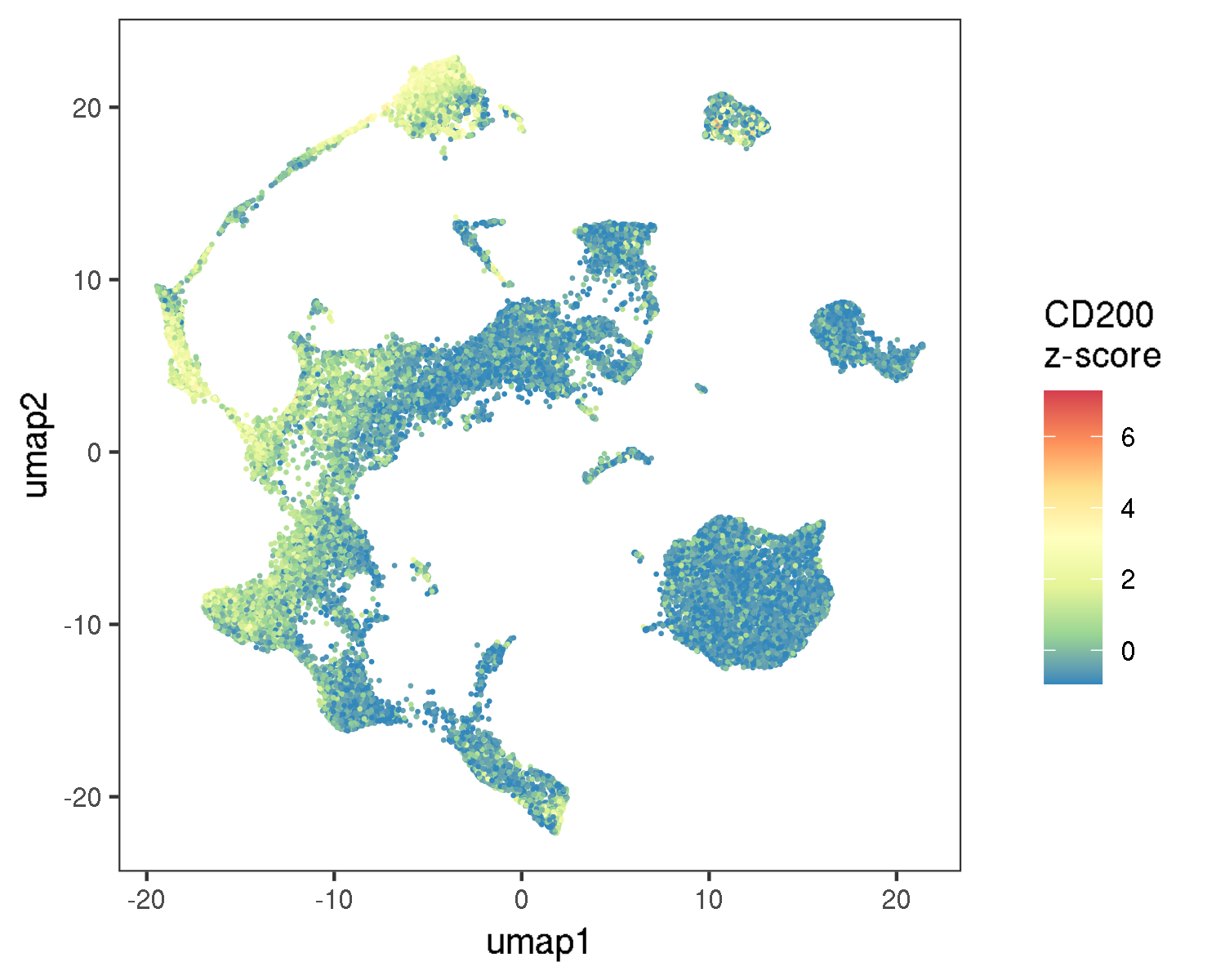

Supplement: Supplementary file 8 — Supplementary Data 5 [file 41467_2024_49883_MOESM8_ESM.zip › BMMC_final_panel_all_markers/CD200.png]

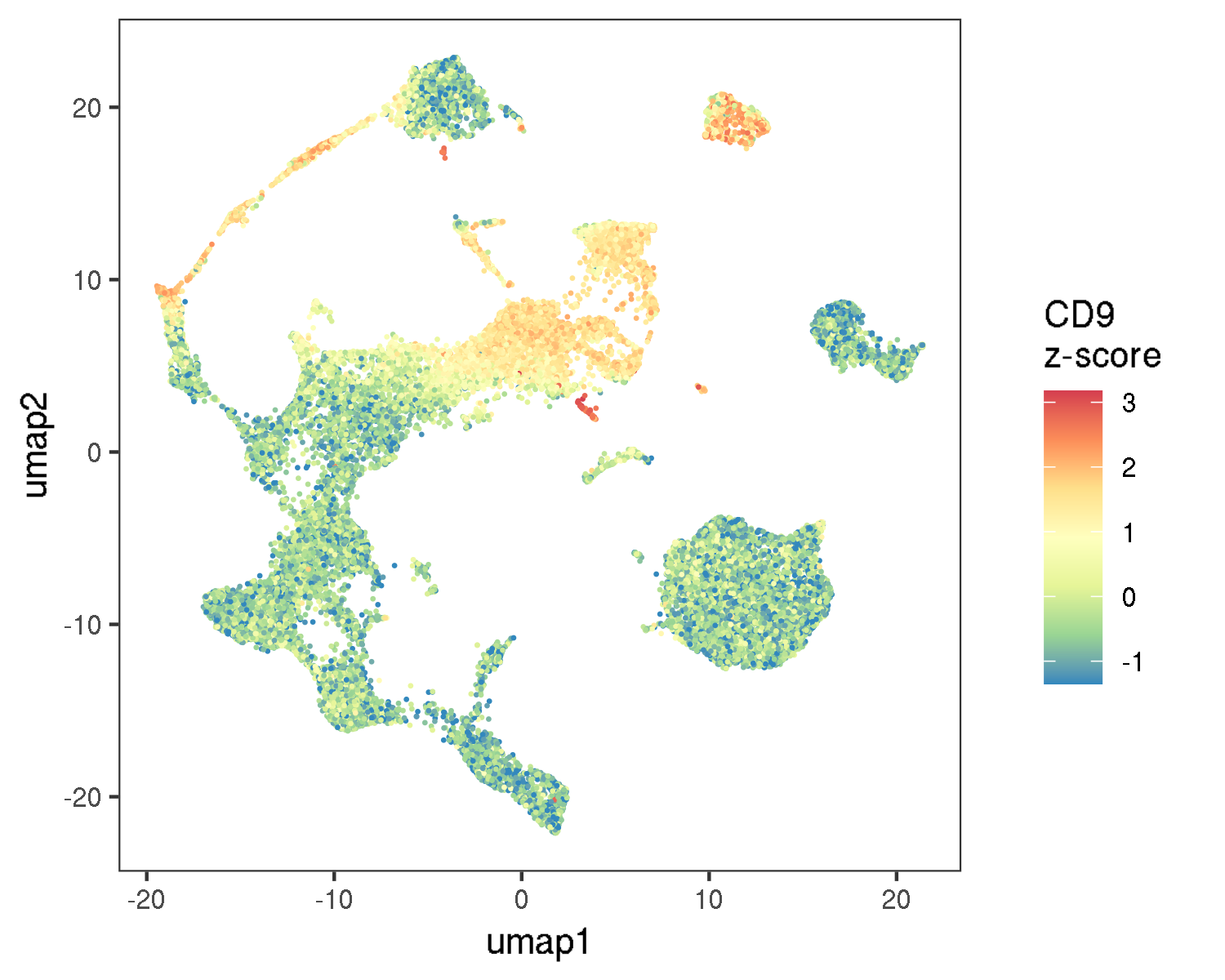

Supplement: Supplementary file 8 — Supplementary Data 5 [file 41467_2024_49883_MOESM8_ESM.zip › BMMC_final_panel_all_markers/CD9.png]

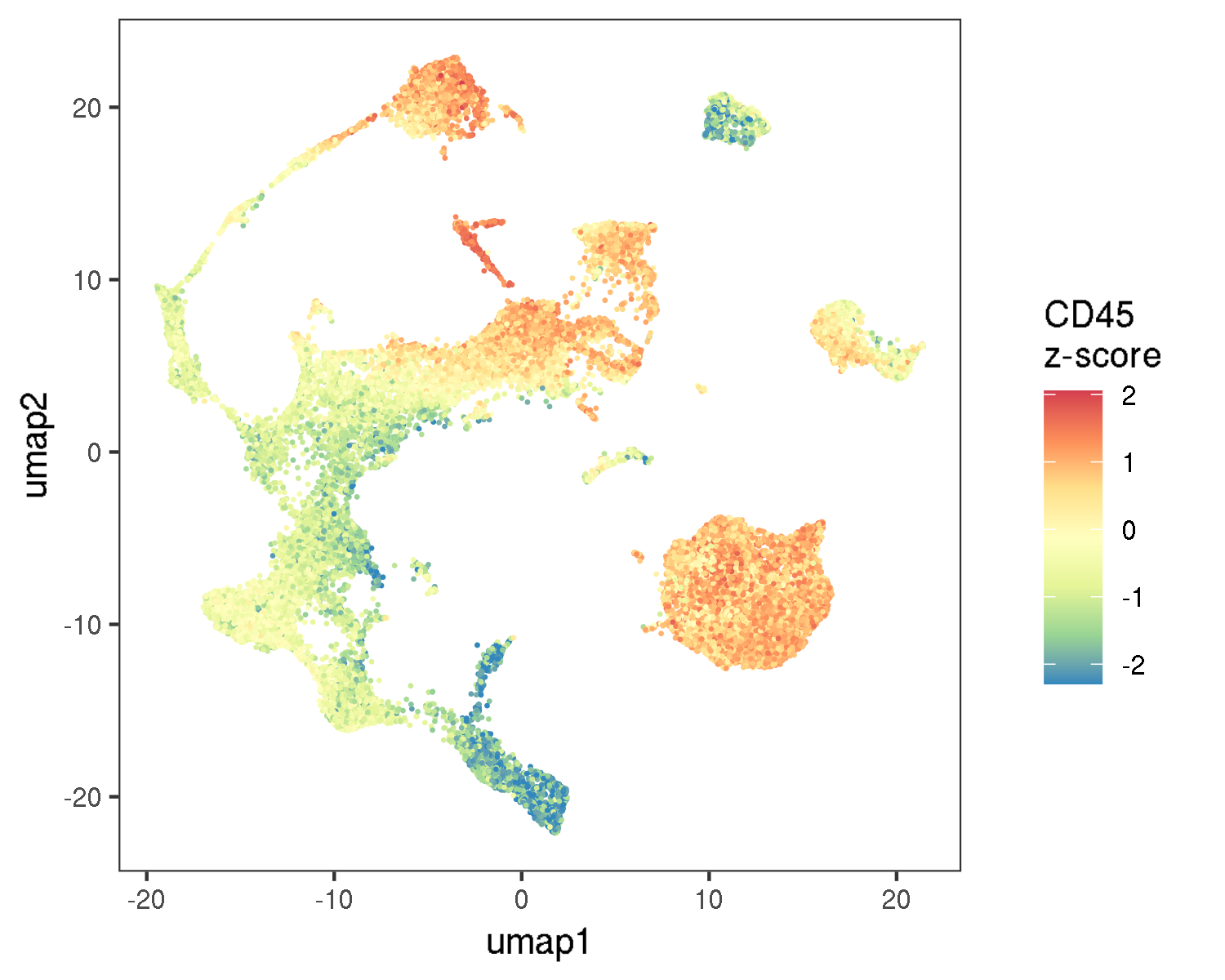

Supplement: Supplementary file 8 — Supplementary Data 5 [file 41467_2024_49883_MOESM8_ESM.zip › BMMC_final_panel_all_markers/CD45.png]

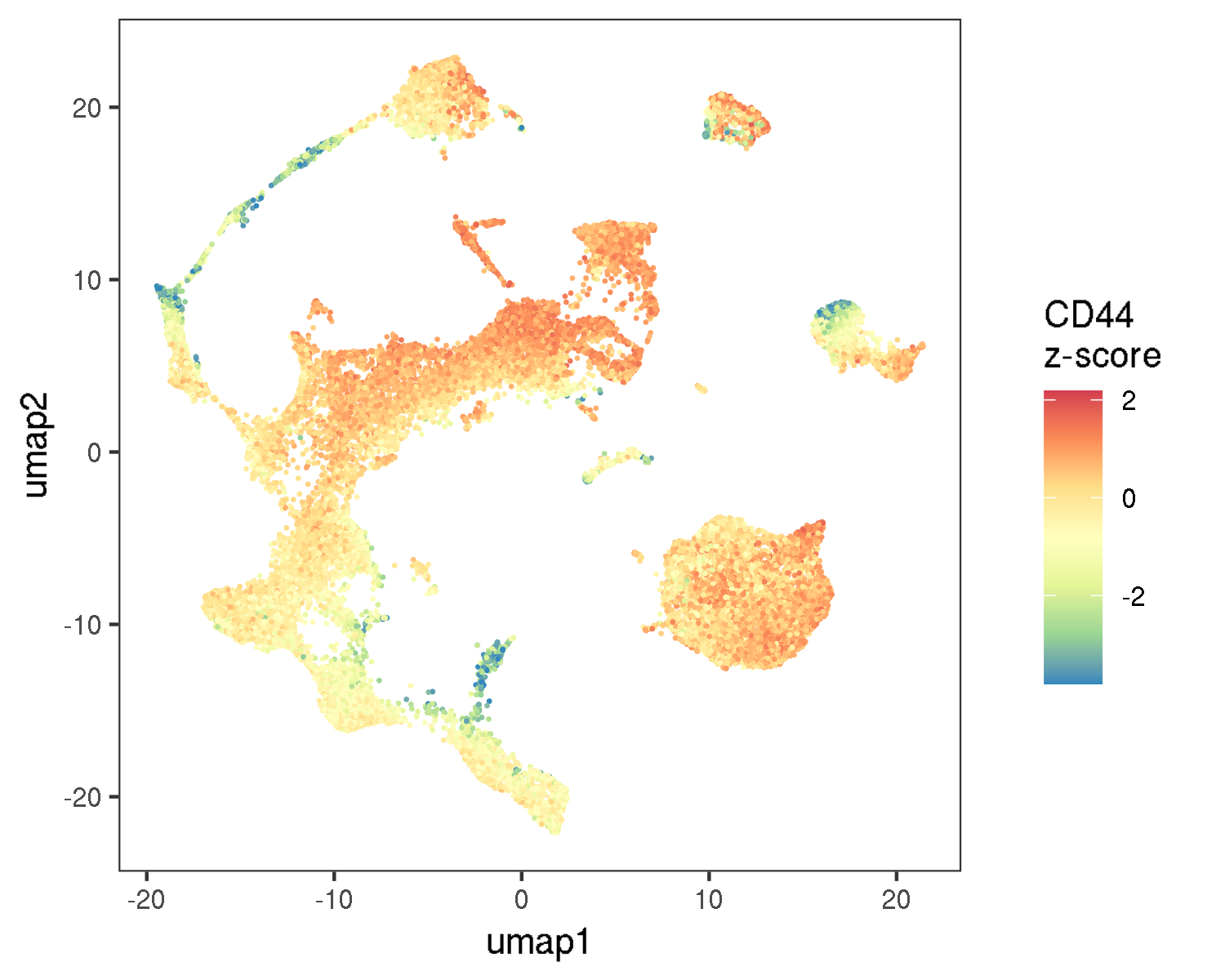

Supplement: Supplementary file 8 — Supplementary Data 5 [file 41467_2024_49883_MOESM8_ESM.zip › BMMC_final_panel_all_markers/CD44.png]

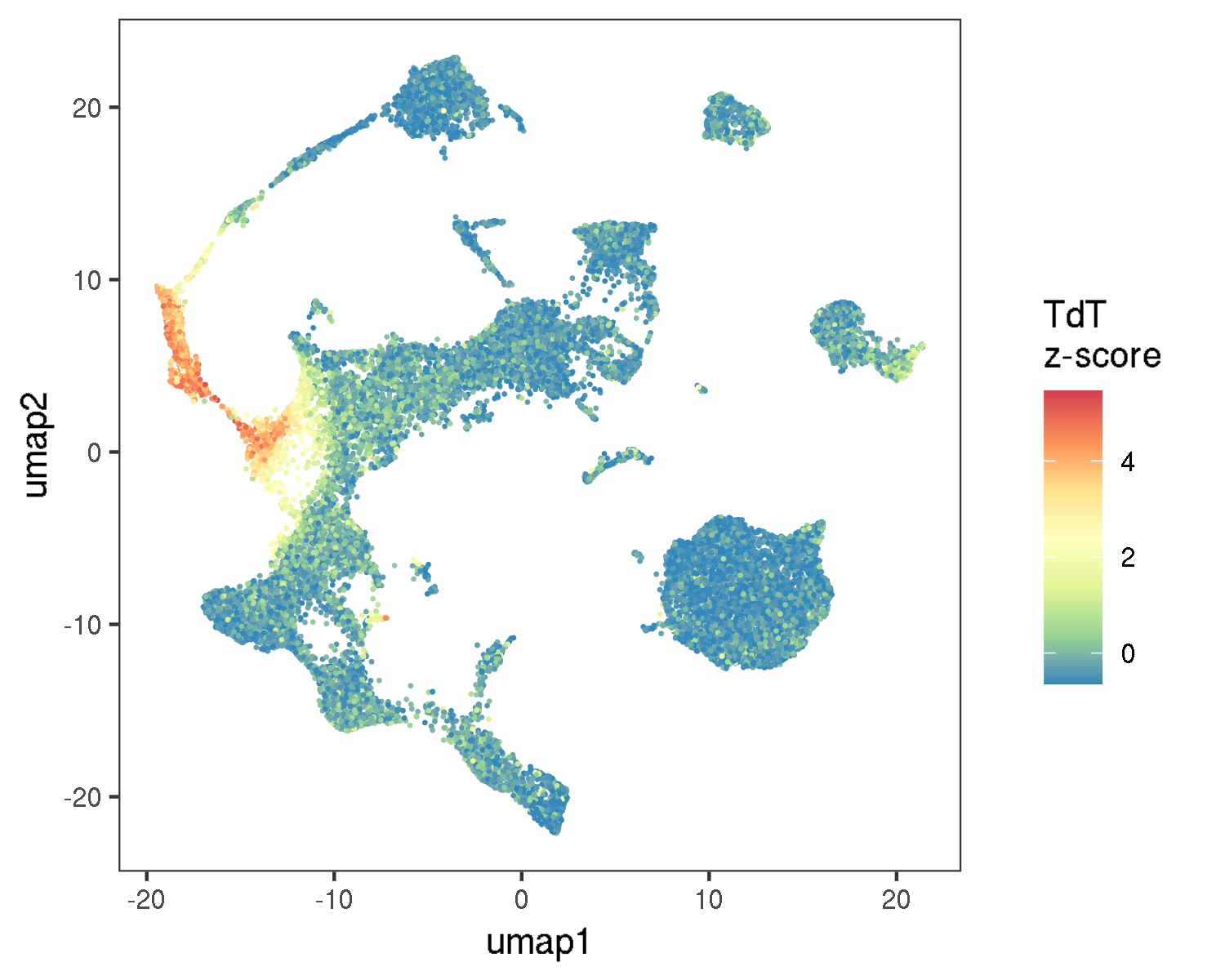

Supplement: Supplementary file 8 — Supplementary Data 5 [file 41467_2024_49883_MOESM8_ESM.zip › BMMC_final_panel_all_markers/TdT.png]

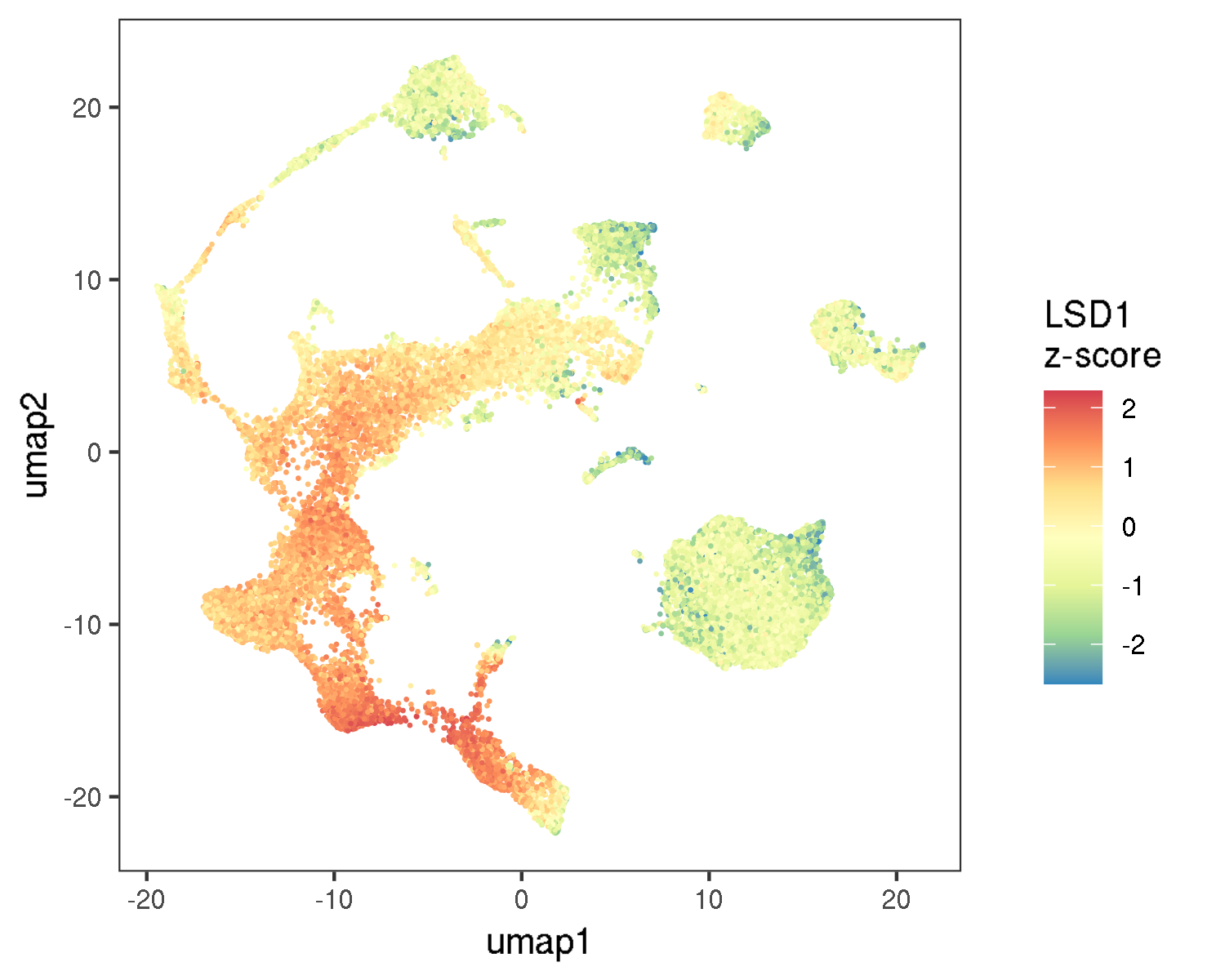

Supplement: Supplementary file 8 — Supplementary Data 5 [file 41467_2024_49883_MOESM8_ESM.zip › BMMC_final_panel_all_markers/LSD1.png]

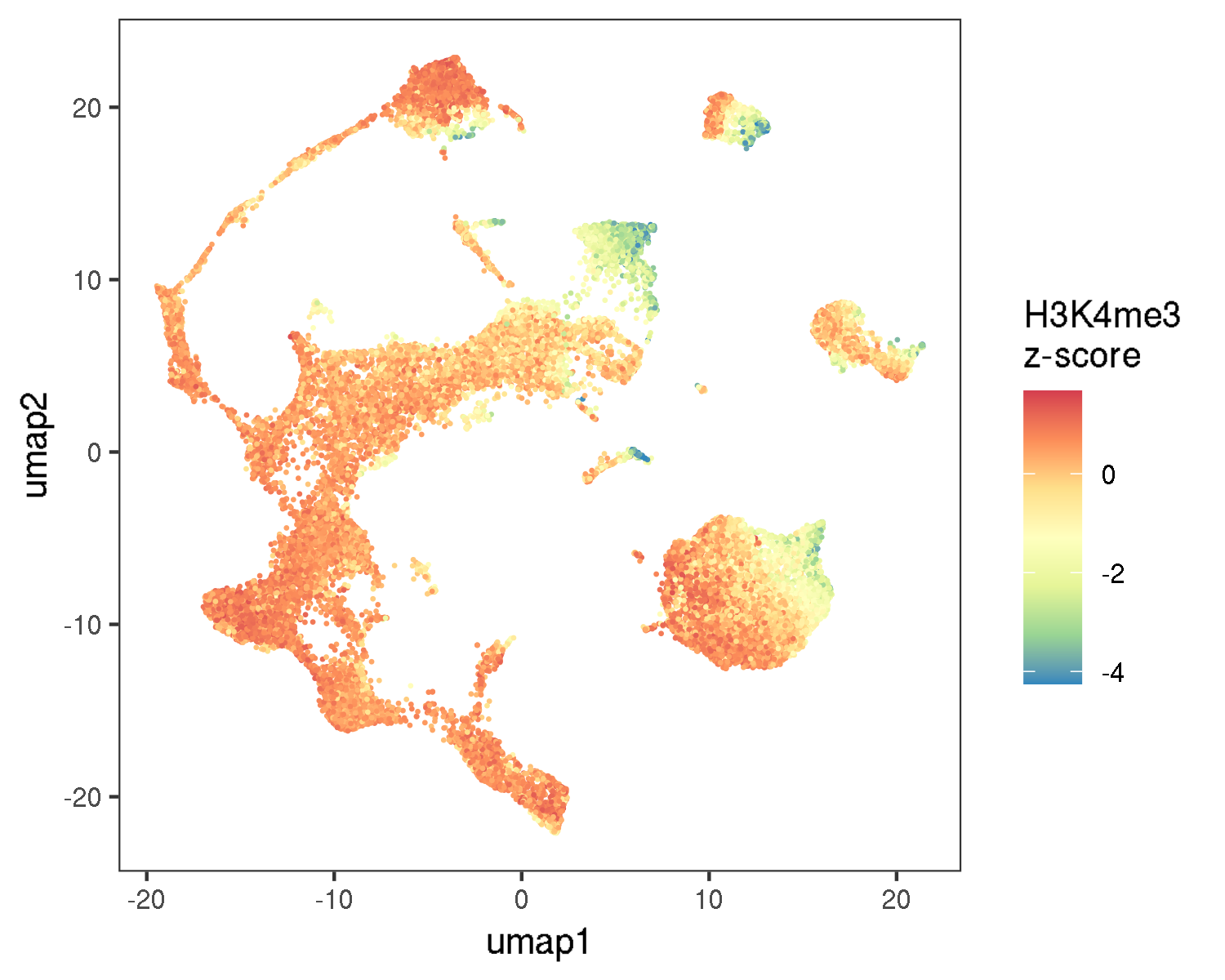

Supplement: Supplementary file 8 — Supplementary Data 5 [file 41467_2024_49883_MOESM8_ESM.zip › BMMC_final_panel_all_markers/H3K4me3.png]

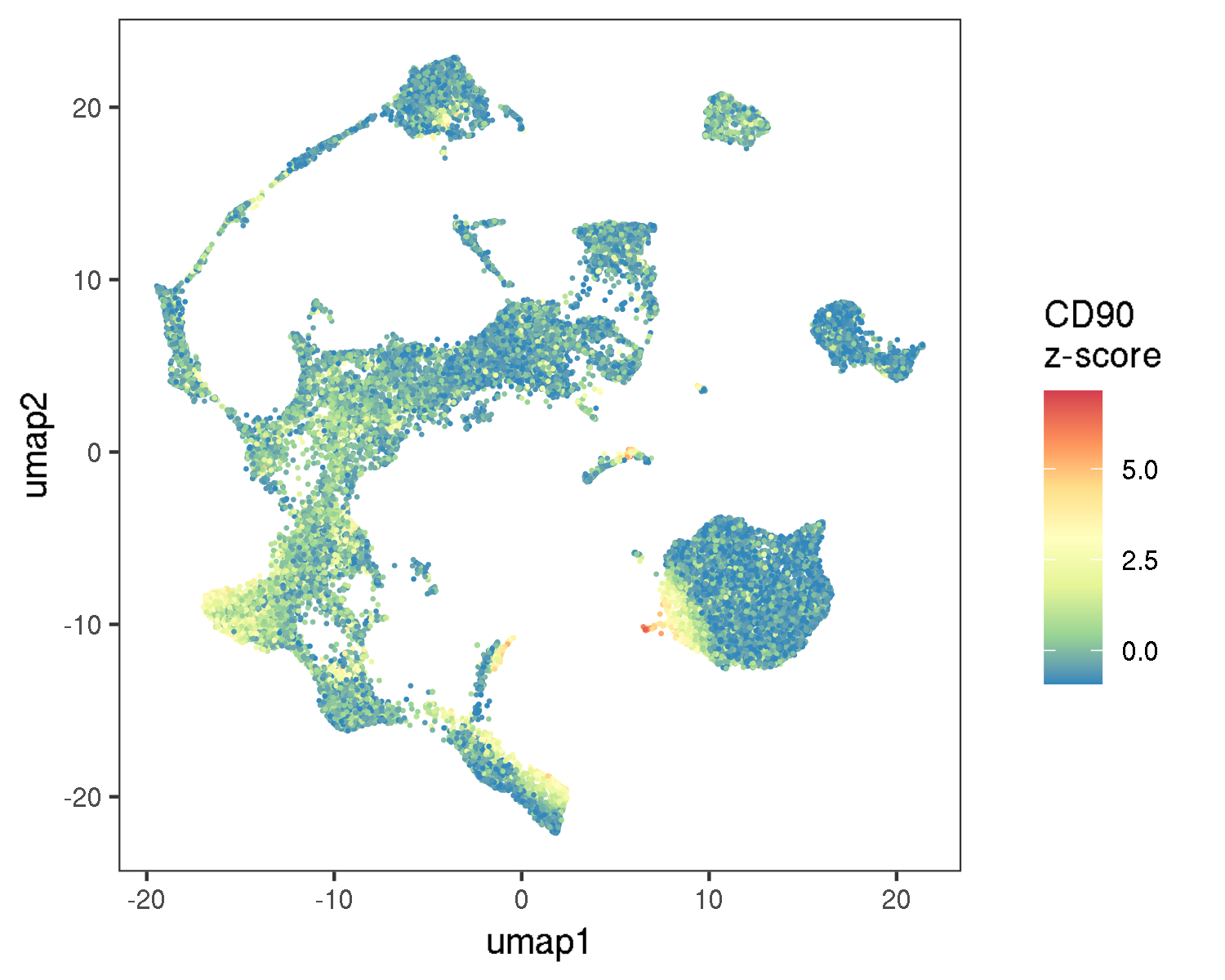

Supplement: Supplementary file 8 — Supplementary Data 5 [file 41467_2024_49883_MOESM8_ESM.zip › BMMC_final_panel_all_markers/CD90.png]

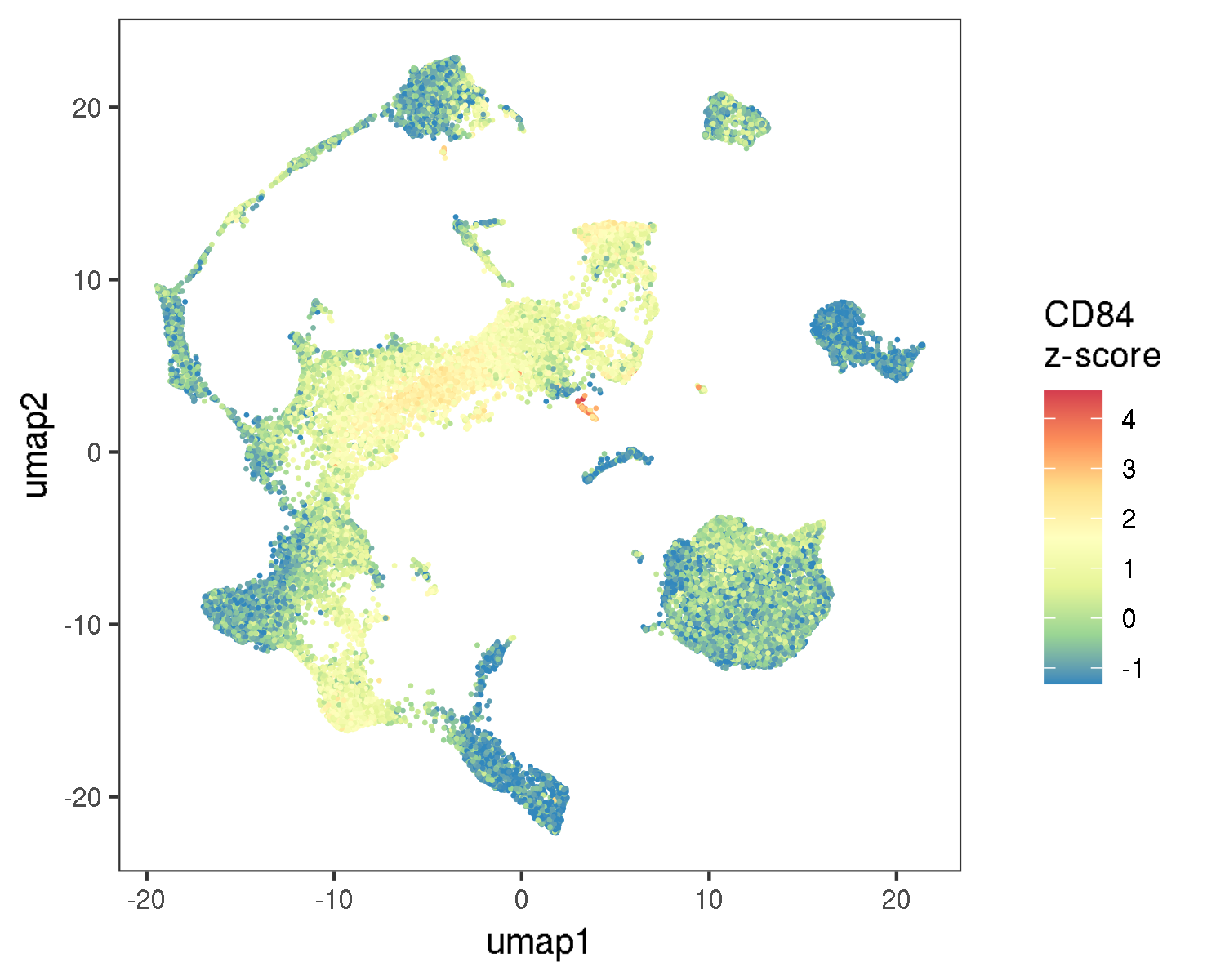

Supplement: Supplementary file 8 — Supplementary Data 5 [file 41467_2024_49883_MOESM8_ESM.zip › BMMC_final_panel_all_markers/CD84.png]

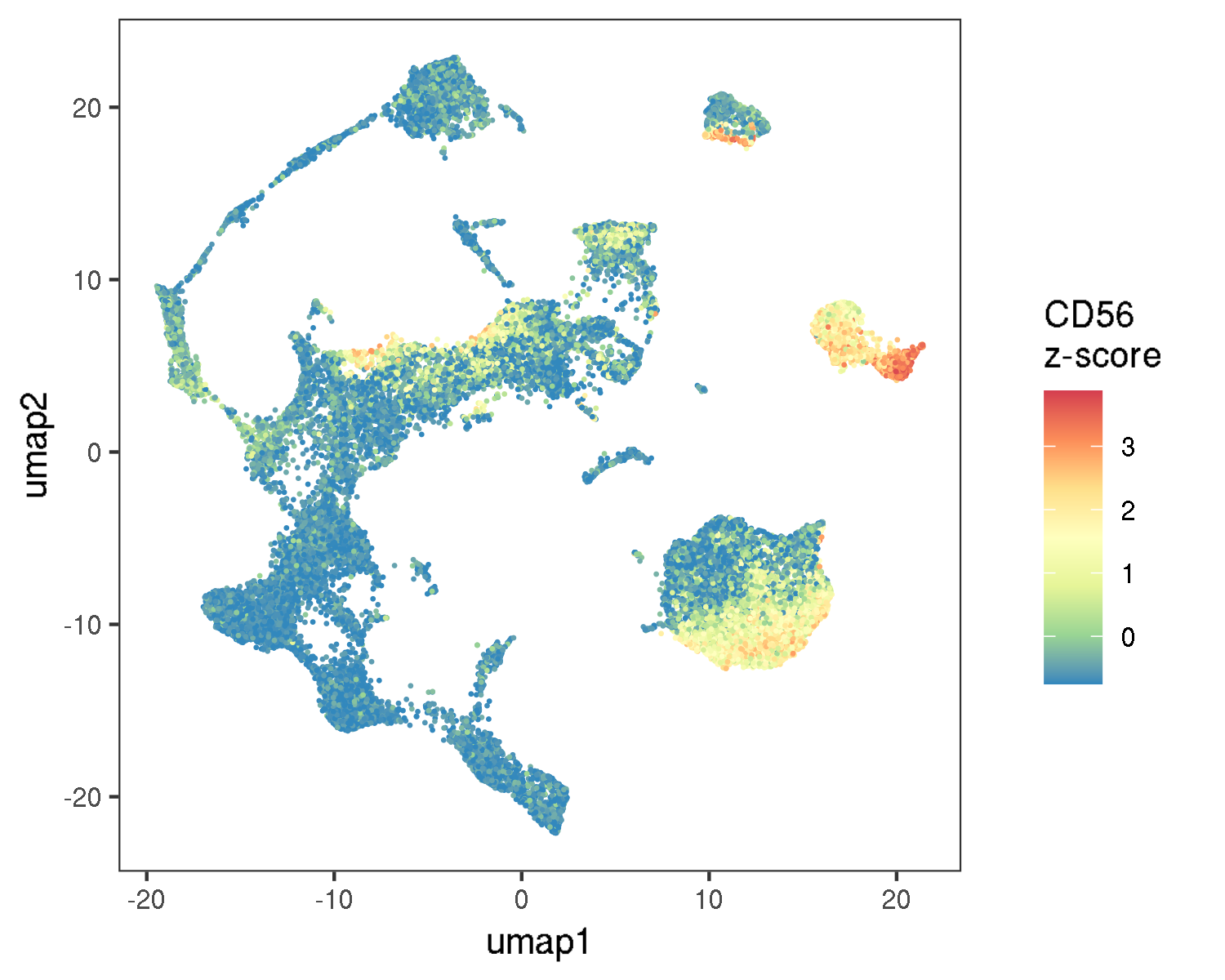

Supplement: Supplementary file 8 — Supplementary Data 5 [file 41467_2024_49883_MOESM8_ESM.zip › BMMC_final_panel_all_markers/CD56.png]
