# Supplementary material for: Terminal deoxynucleotidyl transferase and CD84 identify human multi-potent lymphoid progenitors
Source: Nat Commun. 2024 Jul 13;15:5910. doi: 10.1038/s41467-024-49883-w (PMC11246490; doi:10.1038/s41467-024-49883-w)
Supplement: Supplementary file 9 — Reporting Summary [file 41467_2024_49883_MOESM9_ESM.pdf]

nature portfolio

Corresponding author(s): Sean Bendall
Last updated by author(s): Apr 23, 2024

Reporting Summary

Nature Portfolio wishes to improve the reproducibility of the work that we publish. This form provides structure for consistency and transparency in reporting. For further information on Nature Portfolio policies, see our Editorial Policies and the Editorial Policy Checklist.

Statistics

For all statistical analyses, confirm that the following items are present in the figure legend, table legend, main text, or Methods section.

n/a	Confirmed	
☐	☒	The exact sample size (*n*) for each experimental group/condition, given as a discrete number and unit of measurement
☐	☒	A statement on whether measurements were taken from distinct samples or whether the same sample was measured repeatedly
☐	☒	The statistical test(s) used AND whether they are one- or two-sided<br>*Only common tests should be described solely by name; describe more complex techniques in the Methods section.*
☐	☒	A description of all covariates tested
☐	☒	A description of any assumptions or corrections, such as tests of normality and adjustment for multiple comparisons
☐	☒	A full description of the statistical parameters including central tendency (e.g. means) or other basic estimates (e.g. regression coefficient) AND variation (e.g. standard deviation) or associated estimates of uncertainty (e.g. confidence intervals)
☐	☒	For null hypothesis testing, the test statistic (e.g. *F*, *t*, *r*) with confidence intervals, effect sizes, degrees of freedom and *P* value noted<br>*Give P values as exact values whenever suitable.*
☒	☐	For Bayesian analysis, information on the choice of priors and Markov chain Monte Carlo settings
☒	☐	For hierarchical and complex designs, identification of the appropriate level for tests and full reporting of outcomes
☒	☐	Estimates of effect sizes (e.g. Cohen's *d*, Pearson's *r*), indicating how they were calculated

Our web collection on statistics for biologists contains articles on many of the points above.

Software and code

Policy information about availability of computer code

Data collection	Single-cell mass-cytometry data was generated using a CyTOF2.<br>FACS data was collected using BD FACSDiva.
Data analysis	premessa v0.2.4, FlowSOM v3.10, Seurat v3.2.1, ChrAccR v0.9.17, ArchR v1.0.2, CellEngine (https://cellengine.com/), ELDA (https://bioinf.wehi.edu.au/software/elda/) were used.

For manuscripts utilizing custom algorithms or software that are central to the research but not yet described in published literature, software must be made available to editors and reviewers. We strongly encourage code deposition in a community repository (e.g. GitHub). See the Nature Portfolio guidelines for submitting code & software for further information.

Data

Policy information about availability of data

All manuscripts must include a data availability statement. This statement should provide the following information, where applicable:
- Accession codes, unique identifiers, or web links for publicly available datasets
- A description of any restrictions on data availability
- For clinical datasets or third party data, please ensure that the statement adheres to our policy

The mass cytometry data for the screen and associated code for processing is available at https://doi.org/10.5061/dryad.xgxd254jp and all the other data in the manuscript is available at https://doi.org/10.5061/dryad.1c59zw3zt. All source file except for the mass cytometry screen data on Dryad is included as Source Data

file.

Research involving human participants, their data, or biological material

Policy information about studies with human participants or human data. See also policy information about sex, gender (identity/presentation), and sexual orientation and race, ethnicity and racism.

Reporting on sex and gender	Not applicable, since there were no human research participants in this study.
Reporting on race, ethnicity, or other socially relevant groupings	Not applicable, since there were no human research participants in this study.
Population characteristics	Not applicable, since there were no human research participants in this study.
Recruitment	Not applicable, since there were no human research participants in this study.
Ethics oversight	Not applicable, since there were no human research participants in this study.

Note that full information on the approval of the study protocol must also be provided in the manuscript.

Field-specific reporting

Please select the one below that is the best fit for your research. If you are not sure, read the appropriate sections before making your selection.

[x] Life sciences [ ] Behavioural & social sciences [ ] Ecological, evolutionary & environmental sciences

For a reference copy of the document with all sections, see nature.com/documents/nr-reporting-summary-flat.pdf

Life sciences study design

All studies must disclose on these points even when the disclosure is negative.

Sample size	Human bone marrow (BM) CD34+ progenitors were collected as much as possible from 1 order of 50ml bone marrow per donor. All cells collected from a donor was used for mass cytometry screen or inTAC-seq experiment, where maximum cell number was targeted. Each population was sorted from the total BM sample. For other experiments, aliquots of 10 million BM mononuclear cells were used per replicate experiment. 10 million cell aliquots were determined from prior experiences to obtain minimum 10,000 CD34+ cells per donor to allow analysis of full hematopoieitic differentiation.
Data exclusions	No data were excluded from analysis.
Replication	Bone marrow protein marker screen was conducted with three biological replicates, and validated on several new donor samples in follow-up experiments. ATAC-seq or in vitro differentiation studies were all carried out with at least two biological replicates and two technical replicates each. Both replicates for ATAC-seq experiments were successful. All in vitro differentiation experiment replicates were successful.
Randomization	Randomization was not relevant to this study as there were no clinical or in vivo studies.
Blinding	Blinding was not relevant to this study as there were no clinical or in vivo studies.

Reporting for specific materials, systems and methods

We require information from authors about some types of materials, experimental systems and methods used in many studies. Here, indicate whether each material, system or method listed is relevant to your study. If you are not sure if a list item applies to your research, read the appropriate section before selecting a response.

Materials & experimental systems

n/a	Involved in the study	
[ ]	[x]	Antibodies
[ ]	[x]	Eukaryotic cell lines
[x]	[ ]	Palaeontology and archaeology
[x]	[ ]	Animals and other organisms
[x]	[ ]	Clinical data
[x]	[ ]	Dual use research of concern
[x]	[ ]	Plants

Methods

n/a	Involved in the study	
[x]	[ ]	ChIP-seq
[ ]	[x]	Flow cytometry
[x]	[ ]	MRI-based neuroimaging

Antibodies

Antibodies used

CD45 HI30 BioLegend
CD71 CY1G4 BioLegend
CD38 HIT2 BioLegend
TdT E17-1519 BD
IRF8 7G11A45 BioLegend
CD45RA HI100 Fluidigm
CD34 581 BioLegend
SATB1 O96C6 BioLegend
CD10 HI10a BioLegend
CD90 5E10 BioLegend
CD123 6H6 BioLegend
Anti-FITC polyclonal Southern Biotech
Anti-biotin 1D4-C5 BioLegend
CD39 A1 BioLegend
CD103 Ber-ACT8 BioLegend
CD45RO UCHL1 Biolegend
CD70 113-16 BioLegend
CD165 SN2 (N6-D11) BioLegend
TCRgd 11F2 Fluidigm
CD366 F38-2E2 Fluidigm
CD5 UCHT2 BioLegend
CD199 BL/CCR9 BioLegend
CD200 OX-104 BioLegend
CD4 RPA-T4 BioLegend
CD184 12G5 BioLegend
CD357 621 Fluidigm
TCRab BW242/412 Miltenyi
CD26 BA5b Fluidigm
CD7 M-T701 BD
CD8a RPA-T8 BioLegend
CD254 MIH24 BioLegend
CD278 C398.4A BioLegend
CD52 HI186 BioLegend
CD43 CD43-10G7 BioLegend
CD154 24-31 Fluidigm
CD25 BC96 BioLegend
CD3 UCHT1 Biolegend
CD57 HCD57 BioLegend
CD365 1D12 BioLegend
CD2 TS1/8 BioLegend
CD279 EH12.2H7 DVS
CD56 NCAM16.2 Fluidigm
CD94 DX22 BioLegend
CD6 BL-CD6 Fluidigm
CD81 5A6 BioLegend
CD96 NK92.39 BioLegend
CD122 TU27 BioLegend
FcRL6 2H3 BioLegend
CD178 NOK-1 BioLegend
CD314 1D11 BioLegend
CD223 polyclonal R&D
CD100 A8 Fluidigm
CD319 162.1 BioLegend
CD158a/h HP-MA4 BioLegend
CD197 G043H7 Fluidigm
CD186 K041E5 Fluidigm
CD337 P30-15 BioLegend
CD336 P44-8 BioLegend
CD355 Cr24.1 BioLegend
CX3CR1 2A9-1 BioLegend
CD335 900 BioLegend
CD74 LN2 Fluidigm
CD158e1 DX9 Fluidigm
CD158d mAb 33 (33) BioLegend
CD158f UP-R1 BioLegend

TCR Va24-Ja18 6B11 Fluidigm
NKp80 5D12 BioLegend
CD161 HP-3G10 BioLegend
CD158b DX27 Fluidigm
CD229 HLy9.1.25 Fluidigm
TCR Vd2 B6 BioLegend
TCR Va7.2 3C10 BioLegend
CD307e 509f6 BioLegend
CD48 BJ40 BioLegend
CD179a HSL96 BioLegend
CD30 BY88 BioLegend
CD138 DL-101 BioLegend
CD79b CB3-1 Thermo Fisher
CD19 SJ25C1 BioLegend
CD267 1A1 BioLegend
CD268 11C1 Fluidigm
CD10 HI10a BioLegend
IgL MHL-38 BioLegend
CD179b HSL11 BioLegend
CD20 2H7 BioLegend
TLT-2 MIH61 BioLegend
IgK MHK-49 BioLegend
CD127 HCD127 BioLegend
CD272 MIH26 BioLegend
CD95 DX2 Fluidigm
IgM G20-127 BD
CD27 O323 BioLegend
CD22 HIB22 BioLegend
CD275 9F.8A4 BioLegend
CD290 3C10C5 BioLegend
CD185 51505 Fluidigm
CD351 TX61 BioLegend
CD352 NT-7 BioLegend
CD360 2G1-K12 BioLegend
IgD IA6-2 BioLegend
IgA 9H9H11 BioLegend
CD45RB MEM-55 BioLegend
CD55 JS11 Fluidigm
CD124 G077F6 BioLegend
CD152 L3D10 BioLegend
CD29 TS2/16 BioLegend
CD9 HI9a BioLegend
CD274 29E.2A3 BioLegend
CD84 CD84.1.21 Fluidigm
CD11b ICRF44 BD
CD18 TS1/18 BioLegend
CD172g LSB2.20 BioLegend
CD194 205410 Fluidigm
CD126 UV4 BioLegend
CD137 4B4-1 BD
CD24 ML5 BioLegend
CD69 FN50 Fluidigm
CD257 T7-241 Fluidigm
CD196 G034E3 BioLegend
CD35 E11 BioLegend
CD50 CBR-IC3/1 BioLegend
CD150 A12 (7D4) BioLegend
CD130 2E1B02 Fluidigm
CD46 TRA-2-10 BioLegend
CD40 5C3 BioLegend
CD44 IM7 Fluidigm
CD47 B6H12 BD
CD59 p282/H19 Fluidigm
CD54 HA58 BioLegend
CD134 ACT35 Fluidigm
CD321 OV-5B8 BioLegend
CD82 ASL-24 BioLegend
CD205 HD30 BioLegend

CD86 IT2.2 BioLegend
CD119 GIR-208 BioLegend
CD72 3F3 BioLegend
CD148 A3 BioLegend
CD32 FUN-2 Fluidigm
siglec 10 5G6 R&D
siglec 9 K8 Fluidigm
CD147 HIM6 Fluidigm
CD156c SHM14 BioLegend
CD137 ligand 5F4 Fluidigm
CD98 UM7F8 Fluidigm
CD28 CD28.2 BioLegend
CD166 3A6 BioLegend
integrin b7 FIB504 Fluidigm
CD180 MHR73-11 BioLegend
CD181 8F1/CXCR1 BioLegend
HLA-G 87G BioLegend
CD328 6-434 Fluidigm
CD162 KPL-1 Fluidigm
CD195 T21/8 BioLegend
CD200R OX-108 BioLegend
CD210 3F9 BioLegend
CD182 5E8/CXCR2 BioLegend
CD218a H44 BioLegend
CD244 C1.7 BioLegend
HLA-E 3D12 BioLegend
HLA-A2 BB7.2 BioLegend
CD45R/B220 RA3-6B2 DVS
CD252 11C3.1 BioLegend
CD226 11A8 BioLegend
CD307d 413D12 BioLegend
CD220 B6.220 BioLegend
CD102 CBR-IC2-2 Fluidigm
CD253 RIK-2 BioLegend
CD276 MIH42 BioLegend
CD11a HI111 BioLegend
CD277 BT3.1 BioLegend
CD294 BM16 BioLegend
CD245 DY12 BioLegend
CD101 BB27 Fluidigm
CD317 RS38E BioLegend
CD146 SHM-57 BioLegend
CD107b H4B4 Fluidigm
CD1d 51.1 Fluidigm
CD45RA HI100 BioLegend
CD371 50C1 BioLegend
CCR10 6588-5 BioLegend
CXCR7 8F11-M16 BioLegend
CD53 HI29 BD
CD73 AD2 Fluidigm
TCR Vb8 JR2 (JR.2) BioLegend
CD80 2D10 BioLegend
TCR Vg9 B3 BioLegend
HLA-A/B/C W6/32 BioLegend
HLA-DQ HLADQ1 BioLegend
TCR Vb9 MKB1 BioLegend
TCR Vb13.2 H132 BioLegend
CLA HECA-452 Fluidigm
CD109 W7C5 BioLegend
CD34 581 BioLegend
CD111 R1.302 BioLegend
CD144 BV9 Fluidigm
CD112 TX31 BioLegend
CD131 1C1 BioLegend
CD90 50000000000 BioLegend
CD164 67D2 BioLegend
CD129 AH9R7 BD
CD183 G025H7 Fluidigm

CD115 9-4D2-1E4 BioLegend
CD33 WM53 Fluidigm
CD38 HIT2 BioLegend
CD201 RCR-401 BioLegend
CD14 M5E2 BD
CD11c B-ly6 BD
CD1c L161 BioLegend
CD15 W6D3 Fluidigm
CD16 3G8 BioLegend
CD202b 33.1 (Ab33) BioLegend
CD303 201A BioLegend
CD105 43A3 BioLegend
CD135 4G8 BD
CD243 UIC2 BioLegend
CD117 104D2 BioLegend
CD318 CUB1 BioLegend
CD325 8C11 BioLegend
HLA-DR L243 Biolegend
CD193 5E8 Fluidigm
CD64 10.1 BioLegend
CD42b HIP1 BioLegend
CD51 NKI-M9 BioLegend
CD140b 18A2 BioLegend
CD61 VI-PL2 Fluidigm
CD71 CY1G4 BioLegend
CD66b 80H3 Fluidigm
CD326 9C4 BioLegend
CD45 HI30 BioLegend
CD93 VIMD2 BioLegend
CD114 LMM741 BioLegend
CD143 5-369 BioLegend
CD203c NP4D6 BioLegend
CD170 1A5 BioLegend
CD300f UP-D2 BioLegend
CD163 GHI/61 BioLegend
CD324 67A4 BioLegend
CD36 5-271 BioLegend
siglec 8 7C9 Fluidigm
CD107a H4A3 BioLegend
CD87 VIM5 BioLegend
CD106 STA BioLegend
CD13 L138 BD
CD140a 16A1 BioLegend
CD63 H5C6 BD
CD68 Y1/82A BioLegend
CDw93 R139 BD
CD169 7-239 BioLegend
CD89 A59 Fluidigm
CD41 HIP8 BioLegend
CD88 S5/1 BioLegend
CD207 1000 BioLegend
CD83 HB15e BioLegend
CD172b B4B6 Fluidigm
CD21 LT21 BioLegend
CD123 6H6 Fluidigm
CD155 SKII.4 BioLegend
FceRIa AER-37 (CRA-1) BioLegend
CD282 TL2.1 Fluidigm
CD1b SN13 Fluidigm
CD209 9E9A8 BioLegend
CD66 B1.1/CD66 BD
CD270 122 BioLegend
CD116 4H1 BioLegend
C3aR hC3aRZ8 BioLegend
galectin 9 9M1-3 BioLegend
CD286 TLR6.127 BioLegend
CD301 H037G3 BioLegend
CD23 EBVCS-5 Fluidigm

CD300e UP-H2 BioLegend
podoplanin NC-08 BioLegend
CD1a HI149 Fluidigm
CD206 15-2 DVS
CD304 12C2 Fluidigm
b2 microglobulin 2M2 Fluidigm
CD97 VIM3b Fluidigm
CD273 24F.10C12 DVS
CD370 8F9 BioLegend
CD271 ME20.4 BioLegend
CD172a SE5A5 Fluidigm
CD58 TS2/9 Fluidigm
CD51/CD61 23C6 BioLegend
CD132 TUGh4 Fluidigm
CD66a/c/e ASL-32 Fluidigm
CD85a MKT5.1 BioLegend
CD85d 42D1 BioLegend
CD235ab HIR2 BioLegend
CD85g 17G10.2 BioLegend
CD85h 24 BioLegend
CD85k ZM4.1 BioLegend
CD85j GHI/75 Fluidigm
C5L2 1D9-M12 BioLegend
GARP 7B11 BioLegend
Mac-2 Gal397 BioLegend
CD49e NKI-SAM-1 Fluidigm
CD49b P1E6-C5 Fluidigm
TIM-4 9F4 BioLegend
CD49a TS2/7 Fluidigm
CD49f GoH3 BioLegend
SSEA-3 MC-631 BD
SSEA-4 MC-813-70 BioLegend
SSEA-5 8E+11 BioLegend
integrin a9b1 Y9A2 Fluidigm
CD62L DREG-56 BioLegend
CD62P AK4 BioLegend
CD31 WM59 BioLegend
CD49d 9F10 BioLegend
CD141 1A4 Fluidigm
CD62E HAE-1f BioLegend
CD300c TX45 BioLegend
TSLPR 1B4 BioLegend
CD108 MEM-150 BioLegend
CD167a 51D6 BioLegend
CD284 HTA125 BioLegend
CD213a2 SHM38 BioLegend
CD255 CARL-1 BioLegend
CD258 T5-39 BioLegend
CD261 DJR1 BioLegend
CD262 DJR2-4 (7-8) BioLegend
CD263 DJR3 BioLegend
CD266 ITEM-1 BioLegend
CD334 4FR6D3 BioLegend
CD340 24D2 BioLegend
CD309 89106 R&D
CD344 CH3A4A7 BioLegend
CD49c ASC-1 Fluidigm
integrin b5 AST-3T BioLegend
CD231 SN1a M3-3D9 Fluidigm
lymphotoxin β receptor 31G4D8 BioLegend
EGFR AY13 BioLegend
IL-28RA MHLICR2a BioLegend
LAP TW4-6H10 BioLegend
CD305 NKTA255 Santa Cruz
MSC/NPC W4A5 BioLegend
CD99 HCD99 Fluidigm
IFNg R b chain 2HUB-159 BioLegend
CD354 TREM-26 Fluidigm

jagged 2 MHJ2-523 BioLegend
MIC A/B 6D4 Fluidigm
SUSD2 W3D5 BioLegend
NPC 57D2 BioLegend
PSMA LNI-17 BioLegend
Notch 1 MHN1-519 BioLegend
Notch 2 MHN2-25 BioLegend
Notch 3 MHN3-21 BioLegend
Notch 4 MHN4-2 BioLegend
erbB3 1B4C3 BioLegend
TRA-1-60-R TRA-1-60-R BioLegend
TRA-1-81 TRA-1-81 BioLegend
delta opioid receptor DOR7D2A4 BioLegend
DLL1 MHD1-314 BioLegend
DLL4 MHD4-46 BioLegend
CD298 LNH-94 BioLegend
SUSD2 W5C5 BioLegend
DR3 JD3 BioLegend
CD215 JM7A4 BioLegend
CD338 5D3 BioLegend
GLUT1 EPR3915 Abcam
TNAP W8B2 BioLegend
IgE MHE-18 BioLegend
GLI Poly6424 BioLegend
CD221 1H7 BD
MCT1 P14612 Thermo Fisher
CD104 58XB4 Fluidigm
BMI-1 P51-311 BD
NFkB p65 (pS536)   J144-460 BD
OCT3/4 O50-808 BD
Hes1 3A3 Abnova
AKT_p J1-223.371 Fluidigm
ID2 4E12G5 Abcam
GFI-1 3G8 Abnova
PBX1 UNLB CST
SHIP1 C40G9 CST
RUNX1 1C5B16 BioLegend
PU.1 7C6B05 BioLegend
FoxP3 259D/C7 Fluidigm
GATA1 D52H6 CST
CEBPA 16C12B70 BioLegend
NFIL3 #714401 R&D
ID3 " D16D10" CST
TOX TXRX10 eBioscience
PAX5 (total) 1H9 eBioscience
FoxP1 D35D10 CST
GATA3 TWAJ Fluidigm
TCF1 C63D9 CST
REX1 14HCLC Thermo Fisher
b catenin D13A1 CST
FoxA2 D56D6 CST
GATA2 oligoclonal Life technologies
FoxO3A D19A7 CST
LEF1 C18A7 CST
KLF4 polyclonal CST
GAPDH 65C Thermo Fisher
NRF2 EP1809Y Abcam
CytC 6H2.B4 BioLegend
OGDH polyclonal Thermo Fisher
puromycin 12D10 Sigma
ENO1 EPR10863(B) Abcam
CS EPR8067 Abcam
BrdU 3D4 BD
HIF1A 16H4L13 Thermo Fisher
PFKFB4 polyclonal Thermo Fisher
DRP1 EPR19274 Abcam
ATPA5 15H4C4 Abcam
ACADM 3B7BH7 Abcam

H3_p(S10) 11D8 Biolegend
VDAC1 20B12AF2 Abcam
Cyclin_B1 GNS-1 BD
XBP1 polyclonal Thermo Fisher
S6_p N7-548 BD
PGC1a_p polyclonal R&D
LAL 9G7F12 Abcam
Ki67 B56 BD
NRF1 EPR5554(N) Abcam
LDHA EP1566Y Abcam
IDH1 843219 Abcam
HK2 3D3 Abcam
ACADVL EPR15107(B) Abcam
CPT1A 8F6AE9 Abcam
GOT2 polyclonal Invitrogen
GLUD12 D9F7P CST
OPA1 EPR11057(B) Abcam
H3_pT3 polyclonal CST
CHD1 D8C2 CST
H3k27ac 8173BF CST
SetD8 43AT551.86 Abcam
LSD1 polyclonal CST
HP1beta 4D7B8 Santa Cruz
SmarcD3 polyclonal Milipore
H2Bk5ac D5H1S CST
CTCF D31H2 CST
Ubi-H2A D27C4 CST
DNMT3L ab3493 Abcam
Suz12 D39F6 CST
H3k4me3 " C42D8" CST
H3k18ac polyclonal CST
LaminA/C E-1 Santa Cruz
Acetyl-CBP/p300 polyclonal CST
Histone H1.2 polyclonal Novus
H3k9ac C5B11 CST
H3k27me3 C36B11 CST
Jarid2 D6M9X CST
HH3 (mono) D1H2 CST
CD34 8G12 PerCP/Cy5.5 BD
CD38 E17-1519 BV421 Biolegend
CD45RA HI100 BV605 Biolegend
cd3 UCHT1 biotin Biolegend
cd11b M1/70 biotin Biolegend
cd14 M5E2 biotin Biolegend
cd20 2H7 biotin Biolegend
cd56 5.1H11 biotin Biolegend
cd61 Y2/51 biotin Biolegend
cd66b B1.1/CD66 biotin BD
cd235a HIR2 biotin Biolegend
CD123 6H6 BUV737 BD
CD84 CD84.1.21 APC Biolegend
CD10 HI10a PE-Cy7 Biolegend
TdT E17-1519 FITC BD
SATB1 14/SATB1 Alexa 647 BD
CD14 M5E2 BV421 Biolegend
CD56 5.1H11 BV785 Biolegend
CD45 HI30 BUV395 BD
CD15 HI98 APC BD
CD19 HIB19 PE Biolegend
CD5 UCHT2 FITC Biolegend
CD7 CD7-6B7 Pac Blue Biolegend
CD3 OKT3 BV510 Biolegend
CD4 SK3 APC BD
CD1a HI149 PE Biolegend
CD8 SK1 PE-cy7 BD
CD14 M5E2 BV605 Biolegend
CD45 HI30 APC Biolegend
CD15 HI98 APC-Cy7 Biolegend

Validation	Each antibody was validated for the reactivity with human antigen and for the flow cytometry application. Furthermore, each antibody was validated and titrated using positive and negative human PBMC populations or cell lines prior to this study.

Eukaryotic cell lines

Policy information about cell lines and Sex and Gender in Research

Cell line source(s)	OP9 and OP9-DL4 mouse cell lines were gifted by Zúñiga-Pflücker lab, who developed the cell lines.
Authentication	No authentication for cell lines was performed.
Mycoplasma contamination	OP9 and OP9-DL4 cells were routinely tested for mycoplasma contatmination and were negative.
Commonly misidentified lines (See ICLAC register)	No commonly misidentified lines was used in this study.

Plants

Seed stocks	*Report on the source of all seed stocks or other plant material used. If applicable, state the seed stock centre and catalogue number. If plant specimens were collected from the field, describe the collection location, date and sampling procedures.*
Novel plant genotypes	*Describe the methods by which all novel plant genotypes were produced. This includes those generated by transgenic approaches, gene editing, chemical/radiation-based mutagenesis and hybridization. For transgenic lines, describe the transformation method, the number of independent lines analyzed and the generation upon which experiments were performed. For gene-edited lines, describe the editor used, the endogenous sequence targeted for editing, the targeting guide RNA sequence (if applicable) and how the editor was applied.*
Authentication	*Describe any authentication procedures for each seed stock used or novel genotype generated. Describe any experiments used to assess the effect of a mutation and, where applicable, how potential secondary effects (e.g. second site T-DNA insertions, mosiacism, off-target gene editing) were examined.*

Flow Cytometry

Plots

Confirm that:

- [x] The axis labels state the marker and fluorochrome used (e.g. CD4-FITC).
- [x] The axis scales are clearly visible. Include numbers along axes only for bottom left plot of group (a 'group' is an analysis of identical markers).
- [x] All plots are contour plots with outliers or pseudocolor plots.
- [x] A numerical value for number of cells or percentage (with statistics) is provided.

Methodology

Sample preparation	Bone marrow mononuclear cells were washed with PBS and stained with Live/Dead Aqua (Thermo Fisher) as instructed by the manufacturer in dark for 20 minutes. Cells were washed with FACS buffer with benzonase and spun down at 300g, 5 min, 4°C. Cell surface antibodies was added to samples in FACS buffer in the dark on ice for 30 minutes, followed by a wash with FACS buffer and a spin down at 300g, 5 min, 4°C. For fixation, each sample was fixed with 1ml of 16% PFA for 1 minute before washing with FOXP3 Fix/Perm Buffer (eBiosciences) and centrifugation at 600g, 5 min, 4°C. Intracellular (I/C) panel was prepared in Fix/Perm Buffer and added to samples with a brief vortex. Intracellular staining was in the dark on ice for 30 minute.
Instrument	BD FACSAria Fusion was used for all cell sorting
Software	BD FACSDiva was used for collection and CellEngine (https://cellengine.com/) was used for gating
Cell population abundance	For inTAC-seq, minimum 5,000 cells were collected per population. For limiting dilution assay, specific numbers of cells were sorted into each well using single-cell option on BD FACSDiva.
Gating strategy	Gating schemes for HSPC population is shown in Fig 5c. Positive and negative gates for TdT or CD84 were drawn based on distinct biomodal peaks observed in total CD34+ population.

- [x] Tick this box to confirm that a figure exemplifying the gating strategy is provided in the Supplementary Information.
